# Diastereoselective Three-Component 1,3-Dipolar Cycloaddition to Access Functionalized *β*-Tetrahydrocarboline- and Tetrahydroisoquinoline-Fused Spirooxindoles

**DOI:** 10.3390/molecules29081790

**Published:** 2024-04-15

**Authors:** Yongchao Wang, Yu Chen, Shengli Duan, Yiyang Cao, Wenjin Sun, Mei Zhang, Delin Zhao, Donghua Hu, Jianwei Dong

**Affiliations:** 1 College of Vocational and Technical Education, Yunnan Normal University, Kunming 650092, Chinasunwenjin@stu.ynu.edu.cn (W.S.);; 2 College of Chemistry and Environmental Science, Qujing Normal University, Qujing 655011, China

**Keywords:** three component, 1,3-dipolar cycloaddition, dipolarophiles, fused spirooxindoles, chemoselectivity

## Abstract

A chemselective catalyst-free three-component 1,3-dipolar cycloaddition has been described. The unique polycyclic THPI and THIQs were creatively employed as dipolarophiles, which led to the formation of functionalized *β*-tetrahydrocarboline- and tetrahydroisoquinoline-fused spirooxindoles in 60–94% of yields with excellent diastereoselectivities (10: 1−>99: 1 dr). This reaction not only realizes a concise THPI- or THIQs-based 1,3-dipolar cycloaddition, but also provides a practical strategy for the construction of two distinctive spirooxindole skeletons.

## 1. Introduction

**Scheme 1:**
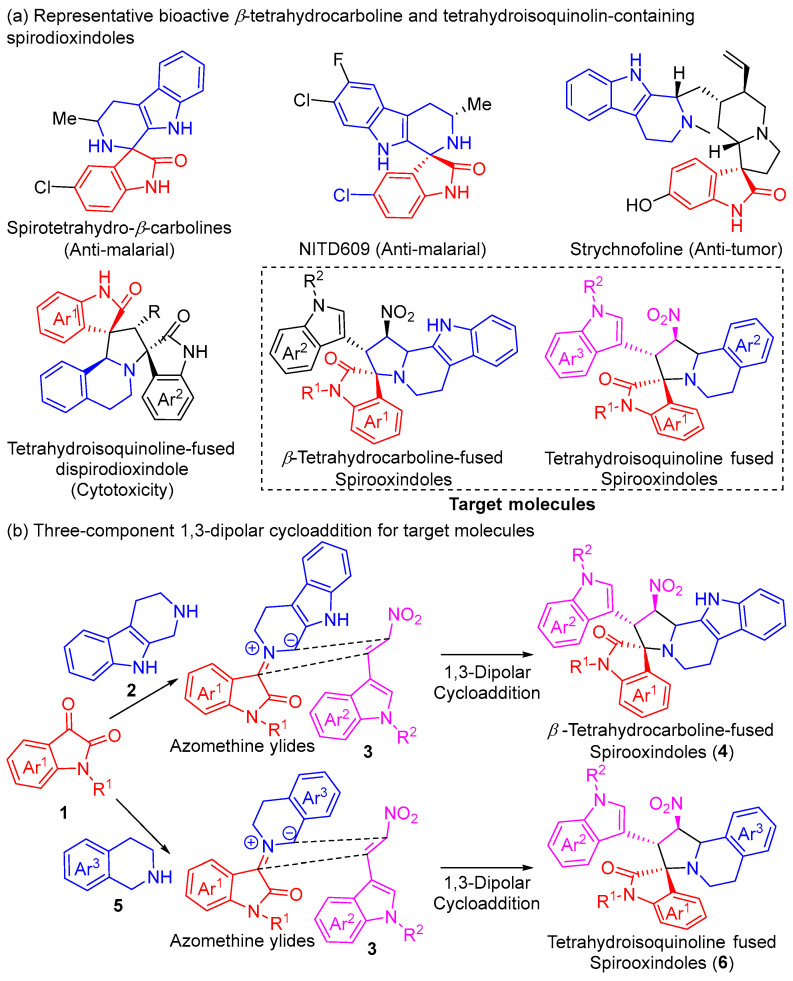
Representative examples of *β*-tetrahydrocarboline, tetrahydroisoquinoline-fused spirooxindoles, and the design synthesis strategy for target molecules.

The polycyclic indole skeleton belongs to a prominent heterocycle, containing a spiro- or fused- indole motif, and plays an important role in organic synthesis, phytochemistry, and drug discovery [1,2,3,4,5]. As two representative subgroups of polycyclic indoles, *β*-tetrahydrocarboline and tetrahydroisoquinoline containing spirooxindoles belong to representative heterocyclic scaffolds. Respectively, *β*-tetrahydrocarboline features a fusion of indole and piperidine ring [6,7,8], and spirooxindole bears a spiro-framework at the 3-position of the oxindole core [9,10,11,12], accompanied by versatile tetrahydroisoquinoline [13,14,15], which are considered the important pharmacophores related to anti-malarial, anti-tumor, and anti-bacterial activities, among others [16,17,18,19]. Constantly, two or more skeleton-units are accommodated in a unique natural or synthetic pharmacologically active compound, which recognized as a drug design strategy via combining multiple molecule-cores to potentialize its biological activities [20,21,22]. *β*-Tetrahydrocarboline- and tetrahydroisoquinoline-fused spirooxindoles, all of which integrate the characteristic unit, are prevalent in natural alkaloids and synthetic pharmaceutical molecules (Scheme 1a). Bifunctionalized spirotetrahydro-*β*-carboline has been identified as providing promising therapeutic leads with antimalarial activity [23]. NITD609 is a promising candidate for the treatment of malaria and human cancer [24,25]. Strychnofoline, isolated from the leaves of Strychnos usambarensis, displays high antimitotic activity against cultures of mice melanoma and Ehrlich tumor cells [26]. Tetrahydroisoquinoline-fused dispirodioxindoles have been proven to be related to cytotoxicity [27]. Hence, there is an enormous demand to achieve functionalized polycyclic indoles, particularly those *β*-tetrahydrocarboline- and tetrahydroisoquinoline-fused spirooxindoles by combining bioactive *β*-tetrahydrocarboline and tetrahydroisoquinoline with spirooxindole, which could be valuable for discovering new bioactive compounds.

Due to the diverse biological activities and the challenge of the simultaneous creation of polycyclic-unit, the development of efficient methods for the construction of *β*-tetrahydrocarbolines, tetrahydroisoquinolines, and spirooxindoles has continuously attracted the attention of synthetic chemists. Eventually, the synthesis of functionalized *β*-tetrahydrocarboline derivatives has been an intense subject in chemistry [28,29,30,31], while the available methods severely rely on the use of transition metals. Additionally, abundant of methodologies also have been developed to access functionalized spirooxindoles [32,33,34,35,36,37]. Herein, the multicomponent 1,3-dipolar cycloaddition has been proved to be one of the most efficient strategie [38,39], while limited on the use of α-amino acids [40,41,42] and benzylamines [43,44,45] as dipolarophiles. Accordingly, the development of efficient synthesis strategies for preparing polycyclic indoles with potential biological activity is still highly desirable. Fortunately, 1,2,3,4-tetrahydro-9H-pyrido [3,4-b]indole (THPI) **2** [46,47,48] and 1,2,3,4-tetrahydroisoquinolines (THIQs) **5** [49,50] have been proven to be valuable synthons, while still undeveloped in the 1,3-dipolar cycloadditions. Here, we present a diastereoselective three-component 1,3-dipolar cycloaddition of isatins (**1**), THPI **2** (or THIQs **5**), and (*E*)-3-(2-nitrovinyl)-indoles (**3**) under green conditions, allowing for rapid access to functionalized *β*-tetrahydrocarboline- and tetrahydroisoquinoline-fused spirooxindoles (**4** and **6**) in excellent yields and diastereoselectivities (Scheme 1b).

## 2. Results and Discussion

To establish the feasibility of this cascade cyclization as well as to optimize the reaction conditions, the three-component 1,3-dipolar cycloaddition of isatin (**1a**), THPI (**2**), and (*E*)-1-methyl-3-(2-nitrovinyl)-1H-indole (**3a**) was selected as the model reaction. As shown in Table 1, the solvent, temperature, and time were screened successively. Initially, the model reaction was performed in EtOH at room temperature for 8 h, the desired *β*-tetrahydrocarboline-fused spirooxindole **4a** was isolated in a 53% yield with excellent diastereoselectivity (>20: 1 dr). Then, diverse solvents (including proton solvents and aprotic solvents) were screened systematically to further optimize the reaction (entries 1–12). The model reaction proceeded smoothly in the all screened solvents except water, which may attribute to the poor dissolution of substrates. Pleasingly, the best reactivity and selectivity were obtained when MeOH was employed (entry 2). To optimize the reactivity, we then investigated the effect of temperature on this cyclization process (entries 2, 13–14). Significantly, the reactivity increased with increasing temperature. Furthermore, appropriately extending the time (to 12 h) is beneficial for the reaction (entries 14–15) and maintained excellent diastereoselectivity, while there was no significantly increase in yield when further extending the time to 24 h (entries 15–16). Overall, the best reaction conditions for synthesizing functionalized *β*-tetrahydrocarboline-fused spirooxindoles are achieved by employing isatins (**1**), 1,2,3,4-tetrahydro-9H-pyrido[3,4-b]indole (**2**) and 3-(2-nitrovinyl)-1H-indoles (**3**) in MeOH under reflux conditions for 12 h.

With the optimal reaction conditions in hand, we then explored the scope of this three-component 1,3-dipolar cycloaddition and the results are summarized in Table 2. Initially, THPI (**2**) and (*E*)-1-methyl-3-(2-nitrovinyl)-1H-indole (**3a**) were fixed as the substrate to test a variety of isatins (**1**). We were pleased to find that this 1,3-dipolar cycloaddition was tolerated for the all tested isatins, bearing various different electron properties at an aromatic ring or *N*-position, affording the corresponding *β*-tetrahydrocarboline-fused spirooxindoles **4a**–**4h** in 78–91% yields. It was worth noting that the R^1^ group of isatins (**1**) has slight influence on diastereoselectivity. Particularly, the H, F, and Me substituents seem more advantageous for the diastereoselectivity (25: 1->99: 1 dr) compared to Br and Cl (10: 1–13: 1 dr, **4d**–**4f**). Furthermore, the *N*-substituted effect of 3-(2-nitrovinyl)-1H-indoles (**3**) was evaluated. All of the examined *N*-substituted groups (H, Me, and Ph) were beneficial for the diastereoselectivity (13: 1–33: 1 dr, **4h**–**4j**), and *N*-protected substrates have better reactivity compared to NH-containing substrate (81% vs. 89–91% yields). Subsequently, various 3-(2-nitrovinyl)-1H-indoles **3** were examined to further evaluate the substrates. Series of 3-(2-nitrovinyl)-1H-indoles **3** bearing different electron properties and substitution patterns were compatible for this 1,3-dipolar cycloaddition, affording the corresponding products **4i**–**4p** in 71–89% yields with 13: 1−>99: 1 dr value. It is worth noting that the three-component 1,3-dipolar cycloaddition of isatins (**1**), substrate **2,** and *N*-unprotected 3-(2-nitrovinyl)-1H-indole were proceeded smoothly and led to the desired products **4p**–**4t**. However, nitro-containing isatin has been shown to be unsuitable in this 1,3-dipolar cycloaddition, and we did not obtain the *β*-tetrahydrocarboline-fused dispirooxindoles **4v**, which may be attributed to spatial hindrance. The structure of product **4c** has been unequivocally established by X-ray crystallographic analysis (CCDC: 2339409). In addition, the practicability of this cascade cyclization was displayed by a 5 mmol scale reaction and afforded the product **4a** in 81% yield with >99: 1 dr value (2.04 g).

To further explore the scope of this 1,3-dipolar cycloaddition, and develop an effective THIQs-based method via catalyst-free strategy, the three-component cycloaddition of isatins (**1**), 3-(2-nitrovinyl)-1*H*-indoles (**3**), and (THIQs) **5** was described under the optimized conditions. As shown in Table 3, this three-component 1,3-dipolar cycloaddition was tolerated for all tested substrates, affording desired functionalized spirooxindoles **6** in 61–94% yields with excellent diastereoselectivities (all >99: 1 dr). Diverse isatins (**1**), 3-(2-nitrovinyl)-1*H*-indoles (**3**) and (THIQs) **5** bearing various different electron properties and substitution patterns, have been systematically evaluated, and the structure and practicability of this cascade cyclization were also clarified by the X-ray crystallographic analysis (CCDC: 2339410) and gram-level experiment of **6b** separately. It is worth noting that the high-purity products **6** were obtained by the simple filtration and recrystallization for the most examples. This 1,3-dipolar cycloaddition not only establishes a THIQ-based catalyst-free synthesis strategy, but also provides a concise, easy-operation synthetic pathway for functionalized spirooxindoles.

Additionally, the 1,3-dipolar cycloaddition of isatins (**1**), (THIQs) **5** and ethyl (*Z*)-2-(2-oxoindolin-3-ylidene)acetates (**7**) was also achieved (Table 4). Under the optimal reactions, various isatins (**1**), (THIQs) **5,** and ethyl (*Z*)-2-(2-oxoindolin-3-ylidene) acetates (**7**) with diverse substitutions were proven to be applicable for this cycloaddition. Via the three-component cascade cycloaddition, the desired tetrahydroisoquinoline-fused dispirooxindoles **8a–8j** were obtained in 60–85% yields with excellent diastereoselectivities (25: 1−>99: 1 dr).

**Scheme 2:**
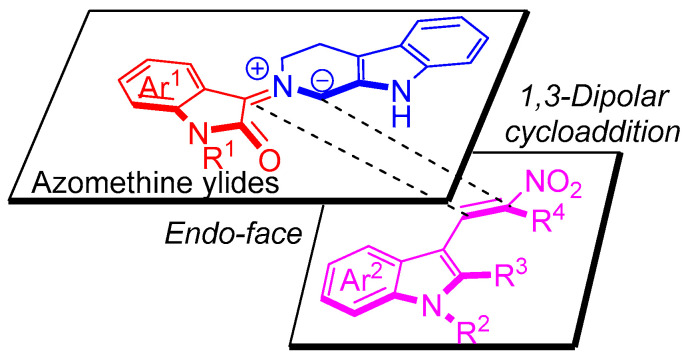
Proposed transition state for the 1,3-dipolar cycloaddition.

A proposed transition state for the stereochemistry was proposed based on the experimental results and previous reports [51,52,53] (Scheme 2). Initially, the isatin-derived azomethine ylides were generated in situ via the condensation of isatins (**1**) and THPI (**2**). Then, the intermolecular 1,3-dipolar cycloaddition subsequently executed via the endo-approach pathway and realized the stereochemistry control of desired products. The steric hindrance between the bulky indole moiety of 3-(2-nitrovinyl)-1*H*-indoles (**3**) and isatin proved plays the key role in the stereochemistry and the excellent diastereoselectivities.

## 3. Conclusions

In conclusion, we described a chemically sustainable three-component 1,3-dipolar cycloaddition of isatins, 3-(2-nitrovinyl)-indoles, and THPI or THIQs, allowing for rapid access to functionalized *β*-tetrahydrocarboline- and tetrahydroisoquinoline-fused spirooxindoles in 60–94% yields with excellent diastereoselectivities (10: 1−>99: 1 dr). This reaction not only realizes a catalyst-free 1,3-dipolar cycloaddition based on unique THPI or THIQs, but also provides a practical strategy for the construction of novel *β*-tetrahydrocarboline- and tetrahydroisoquinoline-fused spirooxindoles. Further investigations of the synthesized *N*-fused polycyclic spirooxindoles are ongoing in our laboratory.

## 4. Experimental Section

### 4.1. General Information

Reagents were of the highest commercial grade (Adamas), the solvents were AR, and were used without further purification. NMR spectra were recorded on a Bruker DRX 400 (^1^H: 400 MHz, ^13^C: 100 MHz) with TMS as the internal standard. Chemical shifts (*δ*) were expressed in ppm, *J* values were given in Hz, and deuterated DMSO-*d_6_* was used as a solvent. IR spectra were recorded on an FT-IR Thermo Nicolet Avatar 360 using KBr pellet. The mass spectroscopic data were obtained from an Agilient 6550 Q-TOF & Thermo Fisher-QE spectrometer. Melting points were determined with an SGWX-4 melting-point apparatus. The reactions were monitored by thin-layer chromatography (TLC) with silica gel GF_254_, and all compounds were visualized by UV and sprayed with H_2_SO_4_ (10%) in ethanol, followed by heating. A suitable single crystal was selected and analyzed with Rigaku XtaLab Synergy. The ^1^H and ^13^C NMR spectras for compounds **4**, **6**, **8**, and single crystal X-ray diffraction study data of compound **4c**, **6b** were included in Supplementary Materials.

### 4.2. General Procedure for the Synthesis of Compounds ***4***

A mixture of isatins (**1**, 0.5 mmol), THPI (**2**, 0.5 mmol), 3-(2-nitrovinyl)-indoles (**3**, 0.5 mmol) in MeOH was stirred under reflux conditions for 12 h and indicated by TLC. Then, the reaction mixture was allowed to cool to room temperature and extracted with dichloromethane (3 × 50 mL). The combined organic layers were washed with saturated brine solution (20 mL), followed by drying over MgSO_4_ and evaporating in vacuo. The crude product was purified by column chromatography to give the pure title compounds.

*2′-(1-Methyl-1H-indol-3-yl)-1′-nitro-1′,2′,5′,6′,11′,11b’-hexahydrospiro[indoline-3,3′-indolizino[8, 7-b]indol]-2-one* **(4a).** Yellow solid; 88% yield; >99: 1 dr; mp 258.7–258.9 °C; IR(KBr) 741, 1011, 1192, 1315, 1335, 1450, 1472, 1553, 1711, 2818, 3057, 3337 cm^−1^; HRMS(ESI) calcd for C_30_H_25_N_5_O_3_ [M+H]^+^ 504.2030, found 504.2024; ^1^H-NMR (400 MHz, DMSO-*d*_6_): *δ* 10.85 (s, 1H, NH), 10.17 (s, 1H, NH), 7.72 (d, *J* = 7.2 Hz, 1H, ArH), 7.44–7.39 (m, 3H, ArH), 7.35–7.31 (m, 2H, ArH), 7.30–7.18 (m, 1H, ArH), 7.23–7.06 (m, 2H, ArH), 7.04–6.98 (m, 1H, ArH), 6.81–6.75 (m, 2H, ArH), 6.66 (d, *J* = 7.6 Hz, 1H, ArH), 6.09–6.07 (m, 1H, CH), 5.97 (d, *J* = 6.8 Hz, 1H, CH), 4.84 (d, *J* = 4.0 Hz, 1H, CH), 3.76 (s, 3H, CH_2_), 2.80–2.73 (m, 2H, CH_2_), 2.68–2.64 (m, 2H, CH_2_); ^13^C-NMR (100 MHz, DMSO-*d*_6_): *δ* 177.2, 143.6, 136.6, 136.5, 130.3, 128.6, 128.5, 127.4, 126.9, 124.9, 122.9, 121.8, 121.5, 119.3, 119.1, 118.4, 118.3, 112.0, 110.3, 110.1, 109.5, 108.9, 92.2, 74.8, 60.6, 51.7, 43.3, 33.0, 22.4.

*5-Fluoro-2′-(1-methyl-1H-indol-3-yl)-1′-nitro-1′,2′,5′,6′,11′,11b’-hexahydrospiro[indoline-3,3′-indolizino[8, 7-b]indol]-2-one* **(4b).** Light yellow solid; 84% yield; 33: 1 dr; mp 269–270 °C; IR(KBr) 741, 814, 1121, 1169, 1321, 1458, 1487, 1557, 1717, 2887, 3308, 3368 cm^−1^; HRMS(ESI) calcd for C_30_H_24_FN_5_O_3_ [M+H]^+^ 522.1936, found 522.1940; ^1^H-NMR (400 MHz, DMSO-*d*_6_+CDCl_3_): *δ* 10.88 (s, 1H, NH), 10.21 (s, 1H, NH), 7.56–7.54 (m, 1H, ArH), 7.43 (d, *J* = 8.0 Hz, 3H, ArH), 7.40–7.34 (m, 1H, ArH), 7.17–7.06 (m, 3H, ArH), 7.00 (d, *J* = 14.8 Hz, 1H, ArH), 6.88–6.82 (m, 2H, ArH), 6.68–6.64 (m, 1H, ArH), 6.10–6.07 (m, 1H, CH), 5.95 (d, *J* = 6.4 Hz, 1H, CH), 4.84 (d, *J* = 4.0 Hz, 1H, CH), 3.76 (s, 3H, CH_3_), 2.81–2.65 (m, 4H, CH_2_); ^13^C-NMR (100 MHz, DMSO-*d*_6_+CDCl_3_): *δ* 177.1, 160.1, 157.7, 139.8, 136.6, 136.5, 130.5, 130.4, 130.1, 128.8, 127.3, 126.9, 121.8, 121.5, 119.4, 119.1, 118.4, 118.3, 116.9, 112.0, 111.1, 110.3, 109.4, 107.8, 91.9, 75.1, 60.7, 51.6, 43.4, 33.0, 22.4.

*6-Chloro-2′-(1-methyl-1H-indol-3-yl)-1′-nitro-1′,2′,5′,6′,11′,11b’-hexahydrospiro[indoline-3,3′-indolizino[8, 7-b]indol]-2-one* **(4c).** Chartreuse solid; 89% yield; >99: 1 dr; mp 268–269 °C; IR(KBr) 568, 733, 918, 1128, 1177, 1319, 1483, 1545, 1618, 1699, 1719, 2893, 3312, 3377 cm^−1^; HRMS(ESI) calcd for C_30_H_24_ClN_5_O_3_ [M+H]^+^ 538.1640, found 538.1644; ^1^H-NMR (400 MHz, DMSO-*d*_6_): *δ* 10.86 (s, 1H, NH), 10.32 (s, 1H, NH), 7.73 (d, *J* = 8.0 Hz, 1H, ArH), 7.44–7.35 (m, 4H, ArH), 7.27 (d, *J* = 8.0 Hz, 1H, ArH), 7.09 (t, *J* = 12.4 Hz, 2H, ArH), 7.00 (t, *J* = 14.8 Hz, 1H, ArH), 6.85 (d, *J* = 3.6 Hz, 2H, ArH), 6.68 (s, 1H, ArH), 6.08 (t, *J* = 10.8 Hz, 1H, CH), 5.93 (d, *J* = 6.4 Hz, 1H, CH), 4.83 (d, *J* = 4.0 Hz, 1H, CH), 3.77 (s, 3H, CH_3_), 2.79–2.65 (m, 4H, CH_2_); ^13^C-NMR (100 MHz, DMSO-*d*_6_): *δ* 177.1, 145.0, 136.6, 136.5, 134.5, 130.1, 128.8, 127.5, 127.3, 126.8, 126.5, 122.8, 121.9, 121.5, 119.4, 119.1, 118.3, 112.0, 110.4, 110.2, 109.4, 107.7, 91.9, 74.5, 60.7, 51.6, 43.3, 33.0, 22.4.

*5-Chloro-2′-(1-methyl-1H-indol-3-yl)-1′-nitro-1′,2′,5′,6′,11′,11b’-hexahydrospiro[indoline-3,3′-indolizino[8, 7-b]indol]-2-one* **(4d).** Chartreuse solid; 81% yield; 11: 1 dr; mp 242–243 °C; IR(KBr) 733, 741, 1045, 1194, 1238, 1323, 1476, 1545, 1618, 1697, 1740, 3300, 3366 cm^-1^; HRMS(ESI) calcd for C_30_H_24_ClN_5_O_3_ [M+H]^+^ 538.1640, found 538.1644; ^1^H-NMR (400 MHz, DMSO-*d*_6_): *δ* 10.87 (s, 1H, NH), 10.29 (s, 1H, NH), 7.71 (s, 1H, ArH), 7.43 (d, *J* = 8.0 Hz, 5H, ArH), 7.01–7.00 (m, 3H, ArH), 6.85 (s, 2H, ArH), 6.68 (s, 1H, ArH), 6.09 (s, 1H, CH), 5.93 (s, 1H, CH), 4.85 (s, 1H, CH), 3.77 (s, 3H, CH_3_), 2.77–2.66 (m, 4H, CH_2_); ^13^C-NMR (100 MHz, DMSO-*d*_6_): *δ* 176.8, 142.5, 136.7, 136.5, 130.9, 130.2, 130.0, 128.8, 127.2, 126.9, 124.8, 121.9, 121.5, 119.4, 119.1, 118.4, 118.3, 112.0, 111.7, 110.3, 109.5, 107.8, 91.8, 74.8, 60.2, 51.7, 43.3, 33.0, 22.4.

*5-Bromo-2′-(1-methyl-1H-indol-3-yl)-1′-nitro-1′,2′,5′,6′,11′,11b’-hexahydrospiro[indoline-3,3′-indolizino[8, 7-b]indol]-2-one* **(4e).** Light yellow solid; 79% yield; 13: 1 dr; mp 235–236 °C; IR(KBr) 428, 733, 820, 1045, 1173, 1194, 1238, 1323, 1474, 1545, 1618, 1697, 1740, 1816, 3300, 3362 cm^−1^; HRMS(ESI) calcd for C_30_H_24_BrN_5_O_3_ [M]^+^ 581.1063, found 581.1070; ^1^H-NMR (400 MHz, DMSO-*d*_6_): *δ* 10.87 (s, 1H, NH), 10.29 (s, 1H, NH), 7.83 (d, *J* = 2.0 Hz, 1H, ArH), 7.51 (d, *J* = 2.0 Hz, 1H, ArH), 7.49–7.42 (m, 2H, ArH), 7.40–7.35 (m, 2H, ArH), 7.10–7.04 (m, 2H, ArH), 7.00 (t, *J* = 14.8 Hz, 1H, ArH), 6.84 (d, *J* = 3.6 Hz, 2H, ArH), 6.64 (d, *J* = 8.4 Hz, 1H, ArH), 6.10–6.08 (m, 1H, CH), 5.92 (d, *J* = 6.4 Hz, 1H, CH), 4.84 (d, *J* = 3.6 Hz, 1H, CH), 3.76 (s, 3H, CH_3_), 2.79–2.65 (m, 4H, CH_2_); ^13^C-NMR (100 MHz, DMSO-*d*_6_): *δ* 176.6, 143.0, 136.7, 136.5, 133.1, 131.3, 130.0, 128.8, 127.6, 127.2, 126.9, 121.9, 121.6, 119.4, 119.1, 118.4, 118.3, 114.5, 112.2, 110.1, 110.3, 109.5, 107.9, 91.7, 74.8, 60.2, 51.7, 43.4, 33.9, 22.4.

*6-Bromo-2′-(1-methyl-1H-indol-3-yl)-1′-nitro-1′,2′,5′,6′,11′,11b’-hexahydrospiro[indoline-3,3′-indolizino[8, 7-b]indol]-2-one* **(4f).** Yellow solid; 83% yield; 10: 1 dr; mp 242–243 °C; IR(KBr) 743, 912, 1329, 1483, 1551, 1611, 1676, 1709, 2820, 3383, 3464 cm^−1^; HRMS(ESI) calcd for C_30_H_24_BrN_5_O_3_ [M+H]^+^ 582.1135, found 582.1139; ^1^H-NMR (400 MHz, DMSO-*d*_6_): *δ* 10.87 (s, 1H, NH), 10.32 (s, 1H, NH), 7.66 (d, *J* = 8.0 Hz, 7H, ArH), 7.43–7.35 (m, 5H, ArH), 7.09 (t, *J* = 13.2 Hz, 2H, ArH), 7.00 (t, *J* = 15.2 Hz, 1H, ArH), 6.84 (t, *J* = 17.6 Hz, 3H, ArH), 6.08–6.06 (m, 1H, CH), 5.91 (d, *J* = 6.4 Hz, 1H, CH), 4.82 (d, *J* = 4.0 Hz, 1H, CH), 3.77 (s, 3H, CH_3_), 2.78–2.64 (m, 4H, CH_2_); ^13^C-NMR (100 MHz, DMSO-*d*_6_): *δ* 177.0, 145.2, 136.6, 136.5, 130.1, 128.8, 127.2, 126.9, 126.7, 122.9, 121.9, 121.5, 119.4, 119.1, 118.3, 112.9, 112.0, 110.4, 109.4, 107.7, 91.9, 74.6, 60.7, 51.5, 33.1, 21.3.

*5, 7-Dimethyl-2′-(1-methyl-1H-indol-3-yl)-1′-nitro-1′,2′,5′,6′,11′,11b’-hexahydrospiro[indoline-3,3′-indolizino[8, 7-b]indol]-2-one* **(4g).** Light yellow solid; 78% yield; >99: 1 dr; mp 274–275 °C; IR(KBr) 737, 1178, 1338, 1489, 1553, 1689, 2910, 3057, 3336, 3385, 3423, 3649, 3711, 3734, 3820 cm^−1^; HRMS(ESI) calcd for C_32_H_29_N_5_O_3_ [M+H]^+^ 532.2343, found 532.2353; ^1^H-NMR (400 MHz, DMSO-*d*_6_): *δ* 10.86 (s, 1H, NH), 10.16 (s, 1H, NH), 7.42 (s, 2H, ArH), 7.36 (s, 3H, ArH), 7.09 (s, 2H, ArH), 7.00 (s, 1H, ArH), 6.93 (s, 1H, ArH), 6.82 (s, 2H, ArH), 6.07 (s, 1H, CH), 5.97 (s, 1H, CH), 4.81 (s, 1H, CH), 3.76 (s, 3H, CH_3_), 2.77 (s, 1H, CH_2_), 2.67 (s, 2H, CH_2_), 2.51 (s, 1H, CH_2_), 2.38 (s, 3H, CH_3_), 1.99 (s, 3H, CH_3_); ^13^C-NMR (100 MHz, DMSO-*d*_6_): *δ* 177.7, 139.8, 136.6, 132.0, 131.7, 130.3, 128.6, 128.3, 127.4, 127.0, 122.6, 121.8, 121.5, 119.3, 119.1, 118.7, 118.3, 110.0, 110.2, 109.4, 108.2, 92.2, 75.0, 60.5, 51.7, 43.3, 33.0, 22.4, 21.3, 16.5.

*Methyl-2′-(1-methyl-1H-indol-3-yl)-1′-nitro-1′,2′,5′,6′,11′,11b’-hexahydrospiro[indoline-3,3′-indolizino[8, 7-b]indol]-2-one* **(4h).** Yellow solid; 91% yield; 25: 1 dr; mp 259.4–262.2 °C; IR(KBr) 1190, 1334, 1493, 1687, 2916, 3066, 3341, 3387, 3433, 3650 cm^−1^; HRMS(ESI) calcd for C_31_H_27_N_5_O_3_ [M+H]^+^ 517.2114, found 517.2121; ^1^H-NMR (400 MHz, DMSO-*d*_6_): *δ* 10.85 (s, 1H, NH), 10.17 (s, 1H, NH), 7.72 (d, *J* = 7.2 Hz, 1H, ArH), 7.43–7.31 (m, 3H, ArH), 7.29–7.27 (m, 2H, ArH), 7.22 (d, *J* = 7.6 Hz, 1H, ArH), 7.11 (s, 2H, ArH), 7.09–6.64 (m, 1H, ArH), 6.08 (d, *J* = 4.4 Hz, 1H, ArH), 6.06 (d, *J* = 4.4 Hz, 1H, ArH), 4.83 (d, *J* = 4.4 Hz, 1H, CH), 3.76 (s, 3H, CH_2_), 2.79–2.64 (m, 4H, CH_3_); ^13^C-NMR (100 MHz, DMSO-*d*_6_): *δ* 177.2, 143.6, 136.6, 130.3, 128.5, 127.4, 126.8, 124.8, 122.9, 121.7, 119.3, 118.3, 111.9, 110.2, 109.5, 107.9, 92.2, 74.8, 60.6, 51.6, 43.3, 33.0, 22.4.

*2′-(1H-Indol-3-yl)-1-methyl-1′-nitro-1′,2′,5′,6′,11′,11b’-hexahydrospiro[indoline-3,3′-indolizino[8, 7-b]indol]-2-one* **(4i).** Yellow solid; 87% yield; 13: 1 dr; mp 267.4–268.2 °C; IR(KBr) 1198, 1365, 1478, 1689, 2962, 3040, 3348, 3390, 3456, 3647 cm^−1^; HRMS(ESI) calcd for C_30_H_25_N_5_O_3_ [M+H]^+^ 504.2030, found 504.2037; ^1^H-NMR (400 MHz, DMSO-*d*_6_): *δ* 10.87 (s, 1H, NH), 10.18 (s, 1H, NH), 7.72 (d, *J* = 7.2 Hz, 1H, ArH), 7.30 (d, *J* = 8.0 Hz, 3H, ArH), 7.27 (s, 2H, ArH), 7.22 (t, *J* = 14.8 Hz, 1H, ArH), 7.11 (t, *J* = 15.2 Hz, 2H, ArH), 7.05 (d, *J* = 6.8 Hz, 1H, ArH), 6.80–6.74 (m, 2H, ArH), 6.65 (d, *J* = 7.6 Hz, 1H, ArH), 6.09–6.07 (m, 1H, ArH), 5.96 (d, *J* = 6.4 Hz, 1H, ArH), 4.84 (d, *J* = 4.4 Hz, 1H, CH), 3.76 (s, 3H, CH_2_), 2.53 (d, *J* = 6.0 Hz, 2H, CH_2_), 2.73 (s, 2H, CH_3_); ^13^C-NMR (100 MHz, DMSO-*d*_6_): *δ* 177.2, 143.6, 136.6, 130.3, 128.6, 127.4, 126.8, 124.8, 122.9, 121.8, 119.3, 118.4, 111.9, 110.3, 109.5, 107.9, 92.9, 74.8, 60.6, 51.7, 43.3, 33.0, 22.4.

*Methyl-1′-nitro-2′-(1-phenyl-1H-indol-3-yl)-1′,2′,5′,6′,11′,11b’-hexahydrospiro[indoline-3, 3′-indolizino[8, 7-b]indol]-2-one* **(4j).** Yellow solid; 89% yield; 33: 1 dr; mp 237.8–242.2 °C; IR(KBr) 1188, 1340, 1355, 1457, 1491, 1545, 1629, 2967, 3337, 3443 cm^−1^; HRMS(ESI) calcd for C_36_H_29_N_5_O_3_ [M+H]^+^ 580.2343, found 580.2351; ^1^H-NMR (400 MHz, DMSO-*d*_6_): *δ* 10.87 (s, 1H, NH), 7.87–7.85 (m, 1H, ArH), 7.47–7.44 (m, 3H, ArH), 7.40–7.28 (m, 6H, ArH), 7.12–7.06 (m, 2H, ArH), 7.00 (t, *J* = 8.0 Hz, 1H, ArH), 6.74 (t, *J* = 12.0 Hz, 1H, ArH), 6.56–6.51 (m, 4H, ArH), 6.19–6.16 (m, 1H, ArH), 5.82 (d, *J* = 6.4 Hz, 1H, ArH), 4.95 (d, *J* = 3.6 Hz, 1H, CH), 3.77 (s, 3H, CH_2_), 2.93 (t, *J* = 8.0 Hz, 1H, CH_2_), 2.78–2.74 (m, 3H, CH_3_); ^13^C-NMR (100 MHz, DMSO-*d*_6_): *δ* 174.7, 144.8, 136.7, 133.8, 130.3, 129.8, 128.5, 127.2, 126.6, 124.9, 121.9, 119.3, 118.4, 112.0, 110.2, 109.6, 108.1, 91.3, 74.8, 61.3, 52.8, 43.3, 33.1, 22.5.

*2′-(5-Methyl-1H-indol-3-yl)-1′-nitro-1′,2′,5′,6′,11′,11b’-hexahydrospiro[indoline-3, 3′-indolizino[8,7-b]indol]-2-one* **(4k).** Yellow solid; 82% yield; 33: 1 dr; mp 233.7–235.6 °C; IR(KBr) 588, 746, 1179, 1192, 1323, 1362, 1450, 1472, 1551, 1622, 1701, 2900, 3331, 3431 cm^−1^; HRMS(ESI) calcd for C_30_H_25_N_5_O_3_ [M+H]^+^ 504.2030, found 504.2037; ^1^H-NMR (400 MHz, DMSO-*d*_6_): *δ* 11.00 (d, *J* = 2.0 Hz, 1H, NH), 10.82 (s, 1H, NH), 10.12 (s, 1H, NH), 7.73 (d, *J* = 7.2 Hz, 1H, ArH), 7.44–7.40 (m, 2H, ArH), 7.33–7.30 (m, 2H, ArH), 7.26–7.17 (m, 1H, ArH), 7.09 (t, *J* = 14.8 Hz, 1H, ArH), 7.00 (t, *J* = 14.8 Hz, 1H, ArH), 6.81 (d, *J* = 7.6 Hz, 1H, ArH), 6.66 (d, *J* = 7.6 Hz, 1H, ArH), 6.37 (s, 1H, ArH), 6.13–6.10 (m, 1H, CH), 5.95 (d, *J* = 6.8 Hz, 1H, CH), 4.55 (d, *J* =4.0 Hz, 1H, CH), 2.81–2.78 (m, 2H, CH_2_), 2.73 (s, 1H, CH_2_), 2.66 (t, *J* = 11.2 Hz, 2H, CH_2_), 2.12 (s, 3H, CH_3_); ^13^C-NMR (100 MHz, DMSO-*d*_6_): *δ* 177.1, 143.8, 136.5, 134.6, 130.3, 130.1, 128.9, 127.5, 127.3, 126.9, 124.9, 124.2, 123.3, 122.8, 121.5, 119.0, 118.3, 118.0, 112.0, 111.5, 110.1, 109.5, 108.4, 91.9, 74.7, 60.7, 52.1, 43.3, 22.4, 21.6.

*2′-(6-Methyl-1H-indol-3-yl)-1′-nitro-1′,2′,5′,6′,11′,11b’-hexahydrospiro[indoline-3,3′-indolizino[8, 7-b]indol]-2-one* **(4l).** Tangerine solid; 79% yield; >99: 1 dr; mp 224.1–225.7 °C; IR(KBr) 451, 737, 804, 1186, 1271, 1329, 1357, 1471, 1541, 1701, 2818, 3350, 3419, 3437 cm^−1^; HRMS(ESI) calcd for C_30_H_25_N_5_O_3_ [M+H]^+^ 504.2030, found 504.2024; ^1^H-NMR (400 MHz, DMSO-*d*_6_): *δ* 10.98 (s, 1H, NH), 10.97 (s, 1H, NH), 10.81 (s, 1H, NH), 7.71 (s, 1H, ArH), 7.69 (s, 2H, ArH), 7.42 (t, *J* = 16.8 Hz, 2H, ArH), 7.30–7.27 (m, 1H, ArH), 7.22–7.07 (m, 2H, ArH), 7.02–6.98 (m, 1H, CH), 5.95 (d, *J* = 6.4 Hz, 1H, CH), 4.82 (d, *J* = 4.0 Hz, 1H, CH), 2.79–2.51 (m, 4H, CH_2_), 2.31 (s, 3H, CH_3_); ^13^C-NMR (100 MHz, DMSO-*d*_6_): *δ* 177.2, 143.7, 136.6, 136.5, 130.8, 130.3, 130.2, 128.7, 126.9, 125.0, 124.8, 123.6, 122.9, 121.5, 120.9, 119.0, 118.3, 117.9, 112.0, 111.7, 110.1, 109.4, 108.7, 92.2, 74.8, 60.7, 51.9, 43.3, 22.4, 21.7.

*2′-(6-Methoxy-1H-indol-3-yl)-1′-nitro-1′,2′,5′,6′,11′,11b’-hexahydrospiro[indoline-3,3′-indolizino[8,7-b]indol]-2-one* **(4m).** Yellow solid; 81% yield; >99: 1 dr; mp 246.7–248.0 °C; IR(KBr) 742, 750, 1175, 1281, 1359, 1452, 1487, 1551, 1618, 1699, 2829, 3354, 3435 cm^−1^; HRMS(ESI) calcd for C_30_H_25_N_5_O_4_ [M+H]^+^ 520.1979, found 520.1986; ^1^H-NMR (400 MHz, DMSO-*d*_6_): *δ* 11.00 (s, 1H, NH), 10.99 (s, 1H, NH), 10.81 (s, 1H, NH), 7.78 (d, *J* = 7.2 Hz, 1H, ArH), 7.45–7.37 (m, 3H, ArH), 7.32–7.23 (m, 1H, ArH), 7.20 (t, *J* = 14.8 Hz, 2H, ArH), 7.08 (t, *J* = 14.8 Hz, 1H, ArH), 7.00 (t, *J* = 14.8 Hz, 1H, ArH), 6.68 (d, *J* = 7.6 Hz, 1H, ArH), 6.62–6.60 (m, 1H, ArH), 6.15–6.12 (m, 1H, ArH), 6.09 (d, *J* = 2.4 Hz, 1H, CH), 5.94 (d, *J* = 6.8 Hz, 1H, CH), 4.83 (d, *J* =4.0 Hz, 1H, CH), 3.42 (d, *J* = 8.0 Hz, 3H, CH_3_), 2.78–2.63 (m, 2H, CH_2_), 2.51 (t, *J* = 3.2 Hz, 2H, CH_2_); ^13^C-NMR (100 MHz, DMSO-*d*_6_): *δ* 177.0, 153.5, 143.8 136.6, 132.2, 130.3, 130.2, 129.0, 127.3, 126.9, 124.9, 124.8, 121.9, 121.5, 119.0, 118.3, 112.7, 112.3, 112.0, 110.1, 109.5, 108.8, 99.3, 91.9, 74.6, 60.7, 55.2, 51.8, 43.3, 22.4.

*2′-(5-Fluoro-1H-indol-3-yl)-1′-nitro-1′,2′,5′,6′,11′,11b’-hexahydrospiro[indoline-3,3′-indolizino[8, 7-b]indol]-2-one* **(4n).** Light yellow solid; 77% yield; >99:1 dr; mp 228.9–231.2 °C; IR(KBr) 754, 939, 1041, 1165, 1321, 1364, 1472, 1558, 1618, 1701, 2916, 3057, 3309, 3453 cm^−1^; HRMS(ESI) calcd for C_29_H_22_FN_5_O_3_ [M+H]^+^ 508.1779, found 508.1786; ^1^H-NMR (400 MHz, DMSO-*d*_6_): *δ* 11.26 (s, 1H, NH), 10.81 (s, 1H, NH), 10.14 (s, 1H, NH), 7.74 (d, *J* = 7.2 Hz, 1H, ArH), 7.40 (d, *J* = 11.2 Hz, 3H, ArH), 7.39–7.31 (m, 2H, ArH), 7.29–7.21 (m, 1H, ArH), 7.09 (t, *J* = 14.8 Hz, 1H, ArH), 6.99 (t, *J* = 14.8 Hz, 1H, ArH), 6.86–6.82 (m, 1H, ArH), 6.67 (d, *J* = 7.6 Hz, 1H, ArH), 6.39 (d, *J* = 10.4 Hz, 1H, ArH), 6.12 (t, *J* = 10.8 Hz, 1H, CH), 5.95 (d, *J* = 6.4 Hz, 1H, CH), 4.78 (d, *J* =4.0 Hz, 1H, CH), 2.79–2.77 (m, 1H, CH_2_), 2.72 (s, 1H, CH_2_), 2.67 (s, 1H, CH_2_), 2.64 (s, 1H, CH_2_); ^13^C-NMR (100 MHz, DMSO-*d*_6_): *δ* 177.1, 143.6, 136.5, 132.9, 130.3, 130.2, 128.6, 127.2, 126.9, 126.6, 125.0, 122.9, 121.5, 119.1, 118.3, 113.0, 112.0, 110.1, 109.8, 109.4, 109.1, 103.2, 103.0, 91.7, 74.7, 60.7, 51.9, 43.3, 22.4.

*2′-(1H-Indol-3-yl)-1′-methyl-1′-nitro-1′,2′,5′,6′,11′,11b’-hexahydrospiro[indoline-3,3′-indolizino[8,7-b]indol]-2-one* **(4o).** Light yellow solid; 74% yield; >99: 1 dr; mp 273–274 °C; IR(KBr) 746, 1180, 1192, 1250, 1321, 1335, 1369, 1452, 1472, 1549, 1618, 1707, 1730, 3213, 3418 cm^−1^; HRMS(ESI) calcd for C_29_H_22_ClN_5_O_3_ [M+H]^+^ 524.1484, found 524.1489; ^1^H-NMR (400 MHz, DMSO-*d*_6_+CDCl_3_): *δ* 11.31 (d, *J* = 2.0 Hz, 1H, NH), 10.83 (s, 1H, NH), 10.15 (s, 1H, NH), 7.72 (d, *J* = 7.2 Hz, 1H, ArH), 7.44–7.40 (m, 4H, ArH), 7.37 (d, *J* = 2.0 Hz, 1H, ArH), 7.21 (t, *J* = 14.8 Hz, 1H, ArH), 7.09 (t, *J* = 14.8 Hz, 1H, ArH), 6.99 (t, *J* = 14.8 Hz, 1H, ArH), 7.76–7.73 (m, 1H, ArH_2_), 6.69–6.65 (m, 2H, ArH), 6.13–6.11 (m, 1H, CH), 5.95 (d, *J* = 6.8 Hz, 1H, CH), 4.82 (d, *J* = 4.0 Hz, 1H, CH), 7.79–7.72 (m, 4H, CH_2_); ^13^C-NMR (100 MHz, DMSO-*d*_6_+CDCl_3_): *δ* 177.1, 143.6, 136.6, 136.5, 130.3, 130.2, 128.5, 126.9, 126.4, 125.8, 125.7, 124.9, 123.0, 121.5, 119.6, 119.4, 119.0, 118.3, 112.0, 111.6, 110.1, 109.4, 109.2, 91.8, 74.7, 60.3, 51.6, 43.3, 22.4.

*2′-(5-Bromo-1H-indol-3-yl)-1′-nitro-1′,2′,5′,6′,11′,11b’-hexahydrospiro[indoline-3,3′-indolizino[8, 7-b]indol]-2-one* **(4p).** Brown solid; 71% yield; >99: 1 dr; mp 254–255 °C; IR(KBr) 457, 746, 1190, 1327, 1471, 1553, 1622, 1697, 3057, 3647, 3724, 3799, 3819, 3838 cm^−1^; HRMS(ESI) calcd for C_29_H_22_BrN_5_O_3_ [M+H]^+^ 568.0979, found 568.0970; ^1^H-NMR (400 MHz, DMSO-*d*_6_): *δ* 11.35 (s, 1H, NH), 10.81 (s, 1H, NH), 10.13 (s, 1H, NH), 7.74 (d, *J* = 7.2 Hz, 1H, ArH), 7.45–7.40 (m, 3H, ArH), 7.33–7.29 (m, 1H, ArH), 7.27–7.22 (m, 2H, ArH), 7.11–7.07 (m, 2H, ArH), 6.99 (t, *J* = 7.6 Hz, 1H, ArH), 6.75 (s, 1H, ArH), 6.68 (d, *J* = 7.6 Hz, 1H, ArH), 6.16–6.13 (m, 1H, CH), 5.94 (d, *J* = 6.4 Hz, 1H, CH), 4.80 (d, *J* = 4.0 Hz, 1H, CH), 2.80 (d, *J* = 4.8 Hz, 1H, CH_2_), 2.78 (d, *J* = 3.6 Hz, 1H, CH_2_), 2.67 (d, *J* = 11.2 Hz, 1H, CH_2_); ^13^C-NMR (100 MHz, DMSO-*d*_6_): *δ* 177.1, 143.6, 136.6, 136.5, 135.3, 130.4, 130.2, 128.6, 126.9, 126.1, 125.0, 124.2, 122.9, 121.5, 120.9, 119.0, 118.3, 113.9, 112.0, 111.9, 110.1, 109.5, 108.7, 91.4, 74.7, 60.7, 51.9, 42.3, 22.4.

*5-Methyl-2′-(1-methyl-1H-indol-3-yl)-1′-nitro-1′,2′,5′,6′,11′,11b’-hexahydrospiro[indoline-3,3′-indolizino[8,7-b]indol]-2-one* (**4q**). Yellow solid; 78% yield; 25:1 dr; mp 242.1–242.3 °C; IR(KBr) 733, 814, 1045, 1171, 1198, 1240, 1323, 1358, 1495, 1543, 1694, 1738, 2814, 3335, 3354 cm^−1^; HRMS(ESI) calcd for C_31_H_27_N_5_O_3_ [M+H]^+^ 518.2187, found 518.2192; ^1^H-NMR (400 MHz, DMSO-*d*_6_): *δ* 10.85 (s, 1H, NH), 10.07 (s, 1H, NH), 7.54 (s, 1H, ArH), 7.43–7.33 (m, 4H, ArH), 7.00 (t, *J* = 14.4 Hz, 4H, ArH), 6.82 (s, 2H, ArH), 6.55 (d, *J* = 8.0 Hz, 1H, ArH), 6.06 (d, *J* = 4.8 Hz, 1H, CH), 5.96 (d, *J* = 5.6 Hz, 1H, CH), 4.81 (d, *J* = 3.2 Hz, 1H, CH), 3.76 (s, 3H, CH_3_), 2.78–2.65 (m, 4H, CH_2_), 2.41 (s, 3H, CH_3_); ^13^C-NMR (100 MHz, DMSO-*d*_6_): *δ* 177.2, 141.1, 136.6, 136.5, 131.8, 130.5, 130.3, 128.6, 128.5, 127.4, 126.9, 125.3, 121.8, 121.5, 119.3, 119.0, 118.5, 118.3, 112.0, 110.2, 109.9, 109.4, 108.1, 92.2, 74.9, 60.6, 51.8, 43.3, 33.0, 22.4, 21.4.

*5-Chloro-2′-(1H-indol-3-yl)-1′-nitro-1′,2′,5′,6′,11′,11b’-hexahydrospiro[indoline-3,3′-indolizino[8,7-b]indol]-2-one* **(4r).** Brown solid; 76% yield; >99:1 dr; mp 257–258 °C; IR(KBr) 584, 744, 1176, 1319, 1436, 1473, 1686, 1734, 2360, 3178, 3246, 3277, 3566, 3689, 3726, 3840 cm^−1^; HRMS(ESI) calcd for C_29_H_22_ClN_5_O_3_ [M+H]^+^ 524.1484, found 524.1494; ^1^H-NMR (400 MHz, DMSO-*d*_6_+CDCl_3_): *δ* 11.19 (s, 1H, NH), 10.85 (s, 1H, NH), 10.28 (s, 1H, NH), 7.71 (s, 1H, ArH), 7.45–7.32 (m, 5H, ArH), 7.10 (t, *J* = 14.8 Hz, 1H, ArH), 7.00 (t, *J* = 14.4 Hz, 2H, ArH), 6.80 (d, *J* = 6.0 Hz, 2H, ArH), 6.69 (d, *J* = 8.4 Hz, 1H, ArH), 6.14–6.11 (m, 1H, CH), 5.76 (d, *J* = 6.0 Hz, 1H, CH), 4.86 (d, *J* = 3.6 Hz, 1H, CH), 2.77–2.65 (m, 4H, CH_2_); ^13^C-NMR (100 MHz, DMSO-*d*_6_+CDCl_3_): *δ* 176.8, 142.6, 136.5, 136.2, 131.1, 130.2, 130.1, 126.9, 126.8, 126.7, 124.9, 124.6, 121.7, 121.5, 119.2, 119.1, 118.3, 118.1, 112.1, 112.0, 111.6, 109.4, 108.7, 91.8, 74.8, 60.9, 51.4, 43.3, 22.4.

*5-Bromo-2′-(1H-indol-3-yl)-1′-nitro-1′,2′,5′,6′,11′,11b’-hexahydrospiro[indoline-3,3′-indolizino[8,7-b]indol]-2-one* **(4s).** Brown solid; 79% yield; 11:1 dr; mp 261–263 °C; IR(KBr) 743, 822, 1098, 1177, 1184, 1474, 1547, 1701, 2369, 2819, 3334, 3406 cm^−1^; HRMS(ESI) calcd for C_29_H_22_BrN_5_O_3_ [M+H]^+^ 568.0979, found 568.0987; ^1^H-NMR (400 MHz, DMSO-*d*_6_+CDCl_3_): *δ* 11.19 (s, 1H, NH), 10.85 (s, 1H, NH), 10.28 (s, 1H, NH), 7.84 (s, 1H, ArH), 7.50 (d, *J* = 8.0 Hz, 1H, ArH), 7.45–7.38 (m, 3H, ArH), 7.33 (d, *J* = 8.0 Hz, 1H, ArH), 7.00 (t, *J* = 14.4 Hz, 1H, ArH), 7.01 (t, *J* = 14.0 Hz, 2H, ArH), 6.78 (t, *J* = 13.2 Hz, 2H, ArH), 6.65 (d, *J* = 8.0 Hz, 1H, ArH), 6.13 (t, *J* = 8.8 Hz, 1H, CH), 5.93 (d, *J* = 5.2 Hz, 1H, CH), 4.87 (d, *J* = 2.8 Hz, 1H, CH), 2.76 (t, *J* = 11.2 Hz, 2H, CH_2_), 2.72 (d, *J* = 4.8 Hz, 1H, CH_2_), 2.67 (d, *J* = 11.2 Hz, 1H, CH_2_); ^13^C-NMR (100 MHz, DMSO-*d*_6_+CDCl_3_): *δ* 176.6, 143.0, 136.5, 136.2, 133.0, 131.5, 130.1, 127.6, 126.9, 125.6, 121.7, 121.5, 119.2, 119.1, 118.3, 118.1, 114.4, 112.1, 112.0, 109.5, 108.7, 71.8, 74.7, 60.9, 51.8, 43.3, 22.4.

*6-Bromo-2′-(1H-indol-3-yl)-1′-nitro-1′,2′,5′,6′,11′,11b’-hexahydrospiro[indoline-3,3′-indolizino[8,7-b]indol]-2-one* **(4t).** Yellow solid; 77% yield; 33: 1 dr; mp 266–267 °C; IR(KBr) 484, 910, 1126, 1182, 1273, 1379, 1445, 1553, 1608, 1699, 3396, 3421, 3437 cm^−1^; HRMS(ESI) calcd for C_29_H_22_BrN_5_O_3_ [M+H]^+^ 568.0979, found 568.0971; ^1^H-NMR (400 MHz, DMSO-*d*_6_): *δ* 11.19 (s, 1H, NH), 11.10 (s, 1H, NH), 10.53 (s, 1H, NH), 7.67 (d, *J* = 7.6 Hz, 1H, ArH), 7.42 (t, *J* = 11.6 Hz, 4H, ArH), 7.40–7.31 (m, 1H, ArH), 7.33 (d, *J* = 8.4 Hz, 1H, ArH), 7.11–6.98 (m, 2H, ArH), 6.86–6.79 (m, 3H, ArH), 6.13–6.10 (m, 1H, CH_3_), 5.94 (d, *J* = 6.4 Hz, 1H, CH), 4.85 (d, *J* = 4.0 Hz, 1H, CH), 2.80–2.77 (m, 2H, CH_2_), 2.73 (t, *J* = 13.6 Hz, 1H, CH_2_), 2.68–2.63 (m, 1H, CH_2_); ^13^C-NMR (100 MHz, DMSO-*d*_6_): *δ* 177.0, 145.2, 136.5, 136.2, 130.1, 128.1, 126.9, 126.8, 125.6, 124.6, 122.8, 121.8, 121.5, 119.2, 119.1, 118.3, 118.1, 113.0, 112.1, 112.0, 109.4, 108.6, 92.0, 74.6, 60.8, 51.3, 43.3, 22.4.

### 4.3. General Procedure for the Synthesis of Compounds ***6***

A mixture of isatins (**1**, 0.5 mmol), THIQs (**5**, 0.5 mmol), and 3-(2-nitrovinyl)-indoles (**3**, 0.5 mmol) in MeOH was stirred under reflux conditions for 12 h. The resulting precipitate was collected by filtrating and washing with cold MeOH 2–3 times. The crude product was further purified by recrystallization (for products **6a**, **6b**, **6e**, **6l**, **6m**, **6o**, **6p**, **6q**, **6r**, **6t**, **6v**, **6w**, and **6y**) in MeOH or column chromatography (for products **6c**, **6d**, **6f**, **6g**, **6h**, **6i**, **6j**, **6k**, **6n**, **6s**, **6u**, **6x**, **6z**, the mixtures of petroleum ether and ethyl acetate were used as eluents) to afford the pure corresponding compound.

*2′-(Indolin-3-yl)-1′-nitro-1′,5′,6′,10b’-tetrahydro-2′H-spiro[indoline-3,3′-pyrrolo[2,1-a]isoquinolin]-2-one* **(6a).** Yellow solid; 92% yield; >99: 1 dr; mp 288–289 °C; IR (KBr) 908, 1057, 1188, 1339, 1472, 1547, 1620, 1705, 2810, 2945, 3337 cm^−1^; HRMS(ESI) calcd for C_27_H_22_N_4_O_3_ [M+H]^+^ 451.1765, found 451.1769; ^1^H-NMR (400 MHz, DMSO-*d*_6_): *δ* 10.82 (s, 1H, NH), 9.56 (s, 1H, NH), 8.25–7.75 (m, 1H, ArH), 7.74 (d, *J* = 7.6 Hz, 1H, ArH), 7.72 (s, 1H, ArH), 7.72–7.68 (m, 3H, ArH), 7.67 (s, 1H, ArH), 7.66–7.63 (m, 2H, ArH), 7.46–7.42 (m, 1H, ArH), 7.29 (s, 1H, ArH), 7.27 (s, 1H, ArH), 7.22 (d, *J* = 0.8 Hz, 1H, CH), 7.21–7.15 (m, 1H, CH), 7.14 (d, *J* = 6.8 Hz, 1H, CH), 6.78–6.75 (m, 1H, CH), 3.53 (t, *J* = 17.6 Hz, 1H, CH_2_), 3.39 (t, *J* = 12.8 Hz, 1H, CH_2_), 3.30–3.17 (m, 1H, CH_2_); ^13^C-NMR (100 MHz, DMSO-*d*_6_): *δ* 177.1, 143.8, 136.6, 135.8, 134.1, 130.2, 129.6, 128.7, 127.7, 127.2, 126.2, 125.1, 124.9, 124.6, 123.0, 121.9, 119.4, 118.4, 111.7, 111.6, 110.1, 109.5, 94.1, 63.6, 52.9, 43.1, 29.6.

*2′-(1H-Indol-3-yl)-5-methyl-1′-nitro-1′,5′,6′,10b’-tetrahydro-2′H-spiro[indoline-3,3′-pyrrolo[2,1-a]isoquinolin]-2-one* **(6b).** Yellow solid; 87% yield; >99: 1 dr; mp 263–264 °C; IR(KBr) 419, 739, 1458, 1491, 1555, 1705, 3385, 3421, 3523, 3736, 3821, 3838 cm^−1^; HRMS(ESI) calcd for C_28_H_24_N_4_O_3_ [M+H]^+^ 465.1921, found 465.1930; ^1^H-NMR (400 MHz, DMSO-*d*_6_): *δ* 11.16 (d, *J* = 1.6 Hz, 1H, NH), 10.01 (s, 1H, NH), 7.55 (d, *J* = 2.4 Hz, 1H, ArH), 7.48 (s, 1H, ArH), 7.28 (d, *J* = 8 Hz, 2H, ArH), 7.21–7.15 (m, 3H, ArH), 7.07 (d, *J* = 7.6 Hz, 1H, ArH), 6.96 (t, *J* = 15.2 Hz, 1H, ArH), 6.68 (t, *J* = 14.8 Hz, 1H, ArH), 6.61 (d, *J* = 8 Hz, 1H, ArH), 6.50 (d, *J* = 8 Hz, 1H, ArH), 6.41–6.38 (m, 1H, ArH), 5.80 (d, *J* = 6.8 Hz, 1H, CH), 4.66 (d, *J* = 4.4 Hz, 1H, CH), 4.18–4.14 (m, 1H, CH), 4.69 (d, *J* = 4.4 Hz, 1H, CH), 2.98–2.93 (m, 1H, CH_2_), 2.71–2.67 (m, 1H, CH_2_), 2.62 (t, *J* = 11.2 Hz, 2H, CH_2_), 2.40 (s, 3H, CH_3_); ^13^C-NMR (100 MHz, DMSO-*d*_6_): *δ* 176.9, 141.2, 136.1, 135.4, 133.8, 131.7, 130.4, 129.5, 128.4, 127.2, 127.1, 126.1, 125.2, 125.1, 125.0, 121.5, 119.1, 118.1, 111.9, 109.8, 108.8, 93.3, 75.0, 63.3, 52.6, 49.1, 42.8, 21.3.

*5-Fluoro-2′-(1H-indol-3-yl)-1′-nitro-1′,5′,6′,10b’-tetrahydro-2′H-spiro[indoline-3,3′-pyrrolo[2,1-a]isoquinolin]-2-one* **(6c).** Brown solid; 82% yield; >99: 1 dr; mp 225–229 °C; IR(KBr) 727, 748, 1186, 1204, 1477, 1544, 1616, 1697, 2857, 2926, 3235 cm^−1^; HRMS(ESI) calcd for C_27_H_21_FN_4_O_3_ [M+H]^+^ 469.1670, found 469.1679; ^1^H-NMR (400 MHz, DMSO-*d*_6_): *δ* 11.29 (s, 1H, NH), 10.23 (s, 1H, NH), 7.66 (d, *J* = 2.0 Hz, 1H, ArH), 7.59 (d, *J* = 2.4 Hz, 1H, ArH), 7.36–7.31 (m, 3H, ArH), 7.29–7.15 (m, 3H, ArH), 6.98 (t, *J* = 14.8 Hz, 1H, ArH), 6.73 (t, *J* = 15.2 Hz, 1H, ArH), 6.65 -6.62(m, 1H, ArH), 6.44–6.41 (m, 1H, ArH), 5.77 (d, *J* = 6.8 Hz, 1H, CH), 4.70 (d, *J* = 4.4 Hz, 1H, CH), 4.18–4.15 (m, 1H, CH), 3.18 (d, *J* = 5.2 Hz, 2H, CH_2_), 3.02–2.94 (m, 1H, CH_2_), 2.69 (t, *J* = 14.4 Hz, 1H, CH_2_), 2.64–2.61 (m, 2H, CH_2_); ^13^C-NMR (100 MHz, DMSO-*d*_6_): *δ* 177.7, 143.5, 135.5, 135.4, 134.9, 133.7, 130.2, 129.6, 128.3, 127.3, 126.2, 125.3, 124.7, 122.7, 120.7, 119.0, 111.0, 110.0, 103.1, 91.0, 63.7, 60.3, 42.8, 40.1, 29.9, 21.2, 14.6, 11.8.

*5-Chloro-2′-(1H-indol-3-yl)-1′-nitro-1′,5′,6′,10b’-tetrahydro-2′H-spiro[indoline-3,3′-pyrrolo[2,1-a]isoquinolin]-2-one* **(6d).** Yellow solid; 80% yield; >99: 1 dr; mp 256–258 °C; IR(KBr) 743, 1175, 1139, 1373, 1458, 1489, 1553, 1695, 3198, 3309, 3368, 3649 cm^−1^; HRMS(ESI) calcd for C_27_H_21_ClN_4_O_3_ [M+H]^+^ 485.1375, found 485.1368; ^1^H-NMR (400 MHz, DMSO-*d*_6_+CDCl_3_): *δ* 10.96 (s, 1H, NH), 9.98 (s, 1H, NH), 7.43 (d, *J* = 2.4 Hz, 1H, ArH), 7.29–7.22 (m, 1H, ArH), 7.18–7.13 (m, 3H, ArH), 7.00–6.95 (m, 2H, ArH), 6.76–6.72 (m, 2H, ArH), 6.59–6.55 (m, 1H, ArH), 6.22–6.18 (m, 1H, CH), 5.85 (d, *J* = 6.8 Hz, 1H, CH), 4.70 (t, *J* = 4.8 Hz, 1H, CH), 4.01–3.97 (m, 1H, CH), 3.26 (m, 2H, CH_2_), 3.07 (s, 1H, CH_2_), 2.76(t, *J* = 14.4 Hz, 1H, CH_2_), 2.76 (t, *J* = 14.4 Hz, 1H, CH_2_), 2.68 (s, 2H, CH_2_); ^13^C-NMR (100 MHz, DMSO-*d*_6_+CDCl_3_): *δ* 177.1, 160.2, 157.8, 139.6, 136.1, 135.3, 133.3, 130.2, 130.1, 129.3, 127.0, 126.9, 125.9, 124.9, 124.5, 121.5, 119.1, 117.9, 116.3, 116.1, 112.1, 111.8, 111.7, 110.8, 110.7, 108.4, 93.2, 75.2, 63.3, 52.6, 49.4, 42.7, 29.9.

*5-Bromo-2′-(1H-indol-3-yl)-1′-nitro-1′,5′,6′,10b’-tetrahydro-2′H-spiro[indoline-3,3′-pyrrolo[2,1-a]isoquinolin]-2-one* **(6e).** Yellow solid; 93% yield; >99: 1 dr; mp 280–282 °C; IR(KBr) 733, 1132, 1190, 1319, 1338, 1375, 1418, 1545, 1614, 1711, 3323, 3566 cm^−1^; HRMS(ESI) calcd for C_27_H_21_BrN_4_O_3_ [M+H]^+^ 529.0870, found 529.0877; ^1^H-NMR (400 MHz, DMSO-*d*_6_+CDCl_3_): *δ* 11.14 (d, *J* = 2.4 Hz, 1H, NH), 10.20 (s, 1H, ArH), 7.60 (d, *J* = 8.0 Hz, 1H, ArH), 7.53 (d, *J* = 2.4 Hz, 1H, ArH), 7.37–7.34 (m, 1H, ArH), 7.30–7.25 (m, 2H, ArH), 7.19–7.13 (m, 3H, ArH), 6.99–6.95 (m, 1H, ArH), 6.75–6.71 (m, 2H, ArH), 6.66 (d, *J* = 8.0 Hz, 1H, ArH), 6.36–6.33 (m, 1H, CH), 5.78 (d, *J* = 6.8 Hz, 1H, CH), 4.67 (d, *J* = 4.4 Hz, 1H, CH), 3.02–2.93 (m, 1H, CH_2_), 2.72 (t, *J* = 11.6 Hz, 1H, CH_2_), 2.67–2.61(m, 2H, CH_2_); ^13^C-NMR (100 MHz, DMSO-*d*_6_+CDCl_3_): *δ* 176.7, 145.2, 136.1, 135.3, 133.5, 129.4, 127.8, 127.1, 126.9, 126.5, 126.1, 125.4, 125.1, 125.0, 122.8, 121.5, 119.1, 117.9, 112.9, 111.9, 108.4, 93.1, 74.7, 63.3, 52.4, 42.8, 29.9.

*2′-(1H-Indol-3-yl)-5-methoxy-1′-nitro-1′,5′,6′,10b’-tetrahydro-2′H-spiro[indoline-3,3′-pyrrolo[2,1-a]isoquinolin]-2-one* **(6f).** Light yellow solid; 77% yield; >99: 1 dr; mp 233–236 °C; IR(KBr) 743, 762, 1030, 1205, 1300, 1335, 1490, 1545, 1690, 2829, 2935, 3442 cm^−1^; HRMS(ESI) calcd for C_28_H_24_N_4_O_4_ [M+H]^+^ 481.1870, found 481.1861; ^1^H-NMR (400 MHz, DMSO-*d*_6_+CDCl_3_): *δ* 10.92 (s, 1H, NH), 9.81 (s, 1H, NH), 7.42 (s, 1H, ArH), 7.26 (t, *J* = 15.6 Hz, 2H, ArH), 7.16 (t, *J* = 7.2 Hz, 4H, ArH), 6.98–6.95 (m, 1H, ArH), 7.77 (t, *J* = 8.4 Hz, 3H, ArH), 6.53 (s, 1H, CH), 6.19–6.16 (m, 1H, CH), 5.85 (d, *J* = 6.8 Hz, 1H, CH), 4.70 (d, *J* = 4.8 Hz, 1H, CH), 4.00 (d, *J* = 4.8 Hz, 1H, CH_2_), 3.85 (d, *J* = 1.6 Hz, 3H, CH_2_), 3.10 (s, 1H, CH_2_), 2.77 (t, *J* = 18.8 Hz, 1H, CH_2_), 2.71 (s, 2H, CH_2_); ^13^C-NMR (100 MHz, DMSO-*d*_6_+CDCl_3_): *δ* 177.1, 156.0, 136.7, 136.1, 135.3, 133.5, 129.5, 129.4, 127.0, 126.9, 125.9, 124.7, 124.4, 121.5, 119.1, 118.1, 114.5, 111.7, 111.0, 110.4, 108.6, 93.5, 75.3, 63.2, 55.9, 52.7, 49.4, 42.7, 29.9.

*6-Bromo-2′-(1H-indol-3-yl)-1′-nitro-1′,5′,6′,10b’-tetrahydro-2′H-spiro[indoline-3, 3′-pyrrolo[2, 1-a]isoquinolin]-2-one* **(6g).** Yellow solid; 74% yield; >99: 1 dr; mp 282–284 °C; IR(KBr) 1543, 1616, 1715, 1734, 2343, 2359, 3337, 3628, 3714, 3820 cm^−1^; HRMS(ESI) calcd for C_27_H_21_BrN_4_O_3_ [M+H]^+^ 529.0870, found 529.0878; ^1^H-NMR (400 MHz, DMSO-*d*_6_+CDCl_3_): *δ* 11.08 (s, 1H, NH), 10.15 (s, 1H, NH), 7.58 (t, *J* = 8.0 Hz, 1H, ArH), 7.49 (s, 1H, ArH), 7.40 (d, *J* = 8.4 Hz, 1H, ArH), 7.36–7.21 (m, 2H, ArH), 7.20–7.14 (m, 3H, ArH), 7.07 (s, 1H, ArH), 6.97–6.67 (m, 3H, ArH), 6.31–6.28 (m, 1H, CH), 5.78 (d, *J* = 6.0 Hz, 1H, CH), 4.67 (d, *J* = 4.0 Hz, 1H, CH), 2.98 (s, 1H, CH_2_), 2.70 (s, 1H, CH_2_), 2.65 (d, *J* = 10.0 Hz, 2H, CH_2_); ^13^C-NMR (100 MHz, DMSO-*d*_6_+CDCl_3_): *δ* 176.8, 145.2, 136.1, 135.3, 133.5, 129.4, 127.7, 127.1, 126.9, 126.3, 126.0, 125.4, 125.0, 124.9, 122.8, 121.5, 119.1, 117.9, 112.9, 111.8, 108.4, 93.1, 74.7, 62.3, 52.4, 42.8, 29.9.

*7-Chloro-2′-(1H-indol-3-yl)-1′-nitro-1′,5′,6′,10b’-tetrahydro-2′H-spiro[indoline-3,3′-pyrrolo[2,1-a]isoquinolin]-2-one* **(6h).** Yellow solid; 76% yield; >99: 1 dr; mp 262–264 °C; IR(KBr) 739, 1177, 1339, 1458, 1545, 1556, 1622, 1714, 3421, 3566, 3751, 3853 cm^−1^; HRMS(ESI) calcd for C_27_H_21_ClN_4_O_3_ [M+H]^+^ 485.1375, found 485.1384; ^1^H-NMR (400 MHz, DMSO-*d*_6_): *δ* 11.22 (d, *J* = 2.0 Hz, 1H, NH), 10.58 (s, 1H, NH), 7.65 (d, *J* = 7.2 Hz, 1H, ArH), 7.60 (d, *J* = 2.4 Hz, 1H, ArH), 7.37 (d, *J* = 7.6 Hz, 1H, ArH), 7.25 (d, *J* = 7.6 Hz, 2H, ArH), 7.21 (d, *J* = 3.2 Hz, 4H, ArH), 6.98 (t, *J* =14.8 Hz, 1H, ArH), 6.69 (t, *J* = 14.8 Hz, 1H, ArH), 6.63 (d, *J* = 8 Hz, 1H, ArH), 6.44–6.43 (m, 1H, ArH), 5.77 (d, *J* = 6.8 Hz, 1H, CH), 4.69 (d, *J* = 4.4 Hz, 1H, CH), 4.19–4.15 (m, 1H, CH), 3.18 (d, *J* = 5.2 Hz, 2H, CH_2_), 2.96 (t, *J* = 16.8 Hz, 1H, CH_2_), 2.92 (s, 1H, CH_2_), 2.90 (s, 2H, CH_2_); ^13^C-NMR (100 MHz, DMSO-*d*_6_): *δ* 176.9, 141.4, 136.2, 135.3, 133.5, 130.5, 130.2, 129.5, 127.3, 126.9, 126.2, 125.2, 125.1, 124.3, 123.4, 121.7, 119.1, 117.9, 114.2, 112.0, 108.5, 92.9, 75.5, 63.4, 52.8, 49.1, 42.9.

*7-Bromo-2′-(1H-indol-3-yl)-1′-nitro-1′,5′,6′,10b’-tetrahydro-2′H-spiro[indoline-3,3′-pyrrolo[2,1-a]isoquinolin]-2-one* **(6i).** Light yellow solid; 79% yield; >99: 1 dr; mp 288–290 °C; IR(KBr) 419, 745, 1338, 1473, 1549, 1684, 1715, 3244, 3309, 3503, 3628, 3734, 3819 cm^−1^; HRMS(ESI) calcd for C_27_H_21_BrN_4_O_3_ [M+H]^+^ 529.0870, found 529.0878; ^1^H-NMR (400 MHz, DMSO-*d*_6_+CDCl_3_): *δ* 11.02 (s, 1H, NH), 10.29 (s, 1H, NH), 7.67 (d, *J* = 7.6 Hz, 1H, ArH), 7.46 (d, *J* = 2.0 Hz, 1H, ArH), 7.40 (d, *J* = 8.0 Hz, 1H, ArH), 7.29–7.25 (m, 2H, ArH), 7.19–7.10 (m, 4H, ArH), 6.98 (t, *J* = 29.6 Hz, 1H, ArH), 6.74 (t, *J* = 14.8 Hz, 1H, ArH), 6.66 (d, *J* = 8.0 Hz, 1H, ArH), 6.27–6.24 (m, 1H, CH), 5.83 (d, *J* = 6.8 Hz, 2H, CH), 4.71 (t, *J* = 4.8 Hz, 1H, CH), 3.07–3.01 (m, 1H, CH_2_), 2.76 (t, *J* = 16.4 Hz, 2H, CH_2_), 2.70 (d, *J* = 14.8 Hz, 1H, CH_2_); ^13^C-NMR (100 MHz, DMSO-*d*_6_+CDCl_3_): *δ* 177.0, 123.0, 136.2, 135.3, 133.3, 132.8, 130.3, 129.3, 127.0, 126.8, 126.0, 124.9, 124.6, 124.2, 123.5, 121.6, 119.1, 118.0, 111.8, 108.3, 102.5, 93.1, 75.7, 63.2, 52.8, 42.7, 29.9.

*7-Fluoro-2′-(1H-indol-3-yl)-1′-nitro-1′,5′,6′,10b’-tetrahydro-2′H-spiro[indoline-3,3′-pyrrolo[2,1-a]isoquinolin]-2-one* **(6j).** Yellow solid; 71% yield; >99: 1 dr; mp 279–281 °C; IR(KBr) 583, 735, 1196, 1339, 1474, 1556, 1645, 1713, 3176, 3367, 3385, 3566, 3736 cm^−1^; HRMS(ESI) calcd for C_27_H_21_FN_4_O_3_ [M+H]^+^ 469.1670, found 469.1673; ^1^H-NMR (400 MHz, DMSO-*d*_6_+CDCl_3_): δ 11.11 (d, *J* = 2.0 Hz, 1H, NH), 10.54 (s, 1H, NH), 7.54 (t, *J* = 10.4 Hz, 2H, ArH), 7.27 (t, *J* = 14.4 Hz, 2H, ArH), 7.22–7.19 (m, 5H, ArH), 7.18 (t, *J* = 5.2 Hz, 1H, ArH), 6.98–6.94 (m, 1H, ArH), 6.71–6.67 (m, 1H, ArH), 6.54 (d, *J* = 8.0 Hz, 1H, ArH), 5.82 (d, *J* = 6.8 Hz, 1H, CH), 4.70 (d, *J* = 4.8 Hz, 1H, CH), 4.08–4.04 (m, 1H, CH), 3.21 (d, *J* = 5.2 Hz, 2H, CH_2_), 3.04 (d, *J* = 5.2 Hz, 1H, CH_2_), 2.97 (d, *J* = 6.8 Hz, 1H, CH_2_), 2.75 (d, *J* = 10.4 Hz, 2H, CH_2_); ^13^C-NMR (100 MHz, DMSO-*d*_6_+CDCl_3_): *δ* 176.7, 147.7, 145.2, 136.2, 135.3, 133.5, 131.6, 131.5, 130.8, 130.6, 129.4, 127.1, 126.9, 126.0, 125.1, 124.9, 123.6, 123.5, 121.5, 120.5, 119.0, 117.8, 117.1, 117.0, 111.8, 108.4, 93.0, 75.1, 75.0, 63.2, 52.9, 49.2, 42.8, 29.9.

*2′-(1H-Indol-3-yl)-1′-nitro-7-(trifluoromethyl)-1′,5′,6′,10b’-tetrahydro-2′H-spiro[indoline-3,3′-pyrrolo[2,1-a]isoquinolin]-2-one* **(6k).** White solid; 61% yield; >99: 1 dr; mp >320 °C; IR (KBr) 737, 1099, 1119, 1171, 1341, 1458, 1558, 1628, 1726, 2835, 2918, 3397 cm^−1^; HRMS(ESI) calcd for C_28_H_21_F_3_N_4_O_3_ [M+H]^+^ 519.1639, found 519.1647; ^1^H-NMR (400 MHz, DMSO-*d*_6_): *δ* 11.21 (d, *J* = 2 Hz, 1H, NH), 10.58 (s, 1H, NH), 7.97 (s, *J* = 7.2 Hz, 1H, ArH), 7.64 (d, *J* = 2.4 Hz, 1H, ArH), 7.62 (s, 1H, ArH), 7.60 (s, 1H, ArH), 7.42 (d, *J* = 7.6 Hz, 1H, ArH), 7.36 (t, *J* = 15.2 Hz, 1H, ArH), 7.19–7.15 (m, 2H, ArH), 6.98–6.94 (m, 1H, ArH), 6.66–6.62 (m, 1H, ArH), 6.49 (t, *J* = 6.4 Hz, 1H, ArH), 6.41 (s, 1H, CH), 5.76 (d, *J* = 6.8 Hz, 1H, CH), 5.85 (d, *J* = 6.8 Hz, 1H, CH), 4.72 (d, *J* = 4 Hz, 1H, CH), 2.98 (s, 1H, CH_2_), 2.71 (s, 1H, CH_2_), 2.66–2.61 (m, 2H, CH_2_); ^13^C-NMR (100 MHz, DMSO-*d*_6_): *δ* 177.2, 141.2, 136.3, 135.4, 133.4, 130.8, 129.5, 128.8, 127.3, 126.8, 126.2, 125.4, 125.1, 123.2, 122.3, 121.6, 119.0, 117.8, 111.9, 111.3, 111.0, 108.5, 92.5, 63.6, 52.9, 40.1, 39.9, 39.3.

*2′-(1H-Indol-3-yl)-1′,9′-dinitro-1′,5′,6′,10b’-tetrahydro-2′H-spiro[indoline-3,3′-pyrrolo[2,1-a]isoquinolin]-2-one* **(6l).** White solid; 91% yield; >99: 1 dr; mp 290–292 °C; IR (KBr) 743, 1302, 1371, 1497, 1553, 1595, 1612, 1686, 2830, 2928, 3065, 3291 cm^−1^; HRMS(ESI) calcd for C_33_H_26_N_4_O_3_ [M+H]^+^ 527.2078, found 527.2084; ^1^H-NMR (400 MHz, DMSO-*d*_6_): *δ* 11.01 (d, *J* = 2 Hz, 1H, NH), 7.83 (d, *J* = 1.6 Hz, 1H, ArH), 7.81 (s, 1H, ArH), 7.52–7.30 (m, 2H, ArH), 7.29–7.21 (m, 5H, ArH), 7.20–7.15 (m, 3H, ArH), 6.97 (t, *J* = 14.8 Hz, 1H, ArH), 6.66 (d, *J* = 7.6 Hz, 1H, ArH), 6.43 (t, *J* = 8 Hz, 3H, ArH), 6.39–6.36 (m, 2H, CH), 5.85 (d, *J* = 6.8 Hz, 1H, CH), 4.80 (d, *J* = 4.4 Hz, 1H, CH), 3.13–3.06 (m, 1H, CH_2_), 3.93 (s, 1H, CH_2_), 2.80 (d, *J* = 3.2 Hz, 1H, CH_2_), 2.77(s, 1H, CH_2_); ^13^C-NMR (100 MHz, DMSO-*d*_6_): *δ* 174.6, 145.0, 136.3, 135.4, 133.9, 133.4, 130.0, 129.5, 129.4, 128.1, 128.0, 127.1, 126.8, 126.5, 126.0, 125.3, 124.6, 124.5, 124.0, 121.5, 118.9, 118.0, 111.6, 109.3, 108.6, 92.5, 79.1, 63.9, 53.9, 40.3, 40.1, 39.5, 30.0.

*2′-(5-Bromo-1H-indol-3-yl)-1′-nitro-1′,5′,6′,10b’-tetrahydro-2′H-spiro[indoline-3,3′-pyrrolo[2,1-a]isoquinolin]-2-one* **(6m).** Brown solid; 90% yield; >99: 1 dr; mp 264–266 °C; IR(KBr) 752, 1184, 1321, 1339, 1458, 1471, 1549, 1618, 1705, 2810, 2947, 3298 cm^−1^; HRMS(ESI) calcd for C_27_H_21_BrN_4_O_3_ [M+H]^+^ 529.0870, found 529.0863; ^1^H-NMR (400 MHz, DMSO-*d*_6_+CDCl_3_): *δ* 11.22 (d, *J* = 1.2 Hz, 1H, NH), 9.98 (s, 1H, NH), 7.69 (d, *J* = 7.2 Hz, 1H, ArH), 7.56 (d, *J* = 2.0 Hz, 1H, ArH), 7.29 (d, *J* = 0.8 Hz, 2H, ArH), 7.14 (t, *J* = 8.8 Hz, 5H, ArH), 7.03–7.01 (m, 1H, ArH), 6.62 (d, *J* = 7.6 Hz, 1H, ArH), 6.57 (d, *J* = 1.2 Hz, 1H, ArH), 6.34–6.31 (m, 1H, CH), 5.82 (d, *J* = 7.2 Hz, 1H, CH), 4.63 (d, *J* = 4.8 Hz, 1H, CH), 3.06 (d, *J* = 5.2 Hz, 1H, CH_2_), 2.77 (t, *J* = 16 Hz, 1H, CH_2_); ^13^C-NMR (100 MHz, DMSO-*d*_6_+CDCl_3_*)*: *δ* 176.9, 143.6, 135.3, 134.9, 133.5, 130.2, 129.4, 128.6, 128.2, 127.0, 126.1, 125.9, 125.0, 124.5, 124.0, 122.7, 120.9, 113.4, 111.9, 110.0, 108.5, 92.5, 74.8, 63.3, 52.8, 42.7, 29.9.

*2′-(5-Methoxy-1H-indol-3-yl)-1′-nitro-1′,5′,6′,10b’-tetrahydro-2′H-spiro[indoline-3,3′-pyrrolo[2,1-a]isoquinolin]-2-one* **(6n).** Brown solid; 81% yield; >99: 1 dr; mp 283–285 °C; IR(KBr) 1173, 1217, 1472, 1549, 1622, 1699, 2995, 3178, 3230, 3462, 3566, 3726, 3840 cm^−1^; HRMS(ESI) calcd for C_28_H_24_N_4_O_4_ [M+H]^+^ 481.1870, found 481.1878; ^1^H-NMR (400 MHz, DMSO-*d*_6_+CDCl_3_): *δ* 10.98 (d, *J* = 2.0 Hz, 1H, NH), 10.03 (s, 1H, NH), 7.71 (d, *J* = 6.8 Hz, 1H, ArH), 7.57 (d, *J* = 2.4 Hz, 1H, ArH), 7.31–7.25 (m, 2H, ArH), 7.21–7.13 (m, 5H, ArH), 6.63 (d, *J* = 7.6 Hz, 1H, ArH), 6.57–6.54 (m, 1H, ArH), 6.43–6.40 (m, 1H, ArH), 5.92 (d, *J* = 2.4 Hz, 1H, CH), 5.78 (d, *J =* 6.8 Hz, 1H, CH), 4.65 (d, *J* = 4.4 Hz, 1H, CH), 3.38 (d, *J* = 6.4 Hz, 3H, CH_2_), 3.00–2.92 (m, 1H, CH_2_), 2.70–2.60 (m, 3H, CH_3_); ^13^C-NMR (100 MHz, DMSO-*d*_6_+CDCl_3_): *δ* 176.7, 153.5, 143.8, 135.4, 133.7, 131.2, 130.1, 129.5, 128.8, 127.3, 127.1, 126.1, 125.4, 125.2, 124.7, 122.7, 112.5, 112.2, 110.1, 108.7, 99.1, 92.9, 79.4, 74.8, 63.3, 55.2, 52.7, 42.8.

*2′-(6-Chloro-1H-indol-3-yl)-1′-nitro-1′,5′,6′,10b’-tetrahydro-2′H-spiro[indoline-3,3′-pyrrolo[2,1-a]isoquinolin]-2-one* **(6o).** Yellow solid; 88% yield; >99: 1 dr; mp 258–260 °C; IR(KBr) 418, 752, 1190, 1339, 1456, 1544, 1695, 3209, 3354, 3523, 3649, 3749, 3802 cm^−1^; HRMS(ESI) calcd for C_27_H_21_ClN_4_O_3_ [M+H]^+^ 485.1375, found 485.1369; ^1^H-NMR (400 MHz, DMSO-*d*_6_): *δ* 10.82 (s, 1H, NH), 9.56 (s, 1H, NH), 8.24 (d, *J* = 7.2 Hz, 1H, ArH), 8.02 (d, *J* = 3.2 Hz, 1H, ArH), 7.79–7.71 (m, 4H, ArH), 7.70–7.63 (m, 3H, ArH), 7.46–7.42 (m, 1H, ArH), 7.28 (d, *J* = 8.0 Hz, 1H, ArH), 7.22 (t, *J* = 7.6 Hz, 1H, ArH), 7.19–7.13 (m, 1H, ArH), 6.78–6.75 (m, 1H, CH), 6.38 (d, *J* = 7.2 Hz, 1H, CH), 5.34 (d, *J* = 4.8 Hz, 1H, ArH), 3.53 (d, *J* = 6.0 Hz, 1H, CH_2_), 3.30 (d, *J* = 1.2 Hz, 1H, CH_2_), 3.27–3.25 (m, 1H, CH_2_), 3.17 (d, *J* = 3.2 Hz, 2H, CH_2_); ^13^C-NMR (100 MHz, DMSO-*d*_6_): *δ* 176.8, 143.6, 136.6, 135.4, 133.7, 130.3, 129.5, 128.3, 127.2, 126.2, 126.1, 125.8, 125.2, 124.7, 122.9, 119.5, 119.3, 111.5, 110.1, 109.1, 92.8, 74.9, 63.3, 52.5, 49.1, 42.8, 29.8.

*2′-(1-Methyl-1H-indol-3-yl)-1′-nitro-1′,5′,6′,10b’-tetrahydro-2′H-spiro[indoline-3,3′-pyrrolo[2,1-a]isoquinolin]-2-one* **(6p).** Yellow solid; 94% yield; >99: 1 dr; mp 265–267 °C; IR (KBr) 741, 1125, 1190, 1331, 1458, 1551, 1628, 1699, 2835, 2918, 3200, 3221 cm^−1^; HRMS(ESI) calcd for C_28_H_24_N_4_O_3_ [M+H]^+^ 465.1921, found 465.1927; ^1^H-NMR (400 MHz, DMSO-*d*_6_): *δ* 10.10 (s, 1H, NH), 7.66 (d, *J* = 6.8 Hz, 1H, ArH), 7.56 (s, 1H, ArH), 7.33 (s, 1H, ArH), 7.29 (s, 1H, ArH), 7.28–7.20 (m, 1H, ArH), 7.19–7.15 (m, 3H, ArH), 7.01 (t, *J* = 14.8 Hz, 1H, ArH), 6.70 (s, *J* = 14.8 Hz, 1H, ArH), 6.59 (s, 1H, ArH), 6.53 (s, 1H, ArH), 6.37–6.34 (m, 1H, CH), 5.78 (d, *J* = 6.8 Hz, 1H, CH), 4.65 (d, *J* = 4.8 Hz, 1H, CH), 4.06 (s, 1H, CH), 3.75 (s, 3H, CH_2_), 2.95 (d, *J* = 6 Hz, 1H, CH_2_), 2.67 (s, 1H, CH_3_), 2.65 (s, 1H, CH_3_), 2.59 (t, *J* = 36.4 Hz, 1H, CH_3_); ^13^C-NMR (100 MHz, DMSO-*d*_6_): *δ* 176.9, 143.6, 136.6, 135.4, 133.7, 130.3, 129.6, 129.1, 128.3, 127.4, 127.2, 126.2, 125.0, 124.7, 122.9, 121.7, 119.2, 118.2, 110.2, 110.1, 108.0, 93.2, 63.2, 52.4, 40.2, 40.0, 39.5, 39.3.

*1′-Ethyl-2′-(1-methyl-1H-indol-3-yl)-1′-nitro-1′,5′,6′,10b’-tetrahydro-2′H-spiro[indoline-3,3′-pyrrolo[2,1-a]isoquinolin]-2-one* **(6q).** Yellow solid; 93% yield; >99: 1 dr; mp 220–224 °C; IR(KBr) 737, 1339, 1474, 1535, 1622, 1697, 3447, 3481, 3566, 3587, 3614 cm^−1^; HRMS(ESI) calcd for C_30_H_28_N_4_O_3_ [M+H]^+^ 493.2234, found 493.2240; ^1^H-NMR (400 MHz, DMSO-*d*_6_+CDCl_3_): *δ* 10.58 (s, 1H, NH), 7.44 (d, *J* = 7.2 Hz, 1H, ArH), 7.29 (d, *J* = 8.0 Hz, 1H, ArH), 7.22 (s, 1H, ArH), 7.19–7.07 (m, 7H, ArH), 6.99–6.93 (m, 2H, ArH), 6.72 (d, *J* = 7.6 Hz, 1H, ArH), 5.27 (s, 1H, CH), 5.25 (s, 1H, CH), 3.74 (s, 3H, CH_2_), 3.21 (s, 3H, CH_2_), 3.04–2.91 (m, 1H, CH_2_), 2.73–2.52 (m, 4H, CH_2_), 2.13–2.04 (m, 1H, CH_3_), 0.81 (t, *J* = 14.0 Hz, 3H, CH_3_); ^13^C-NMR (100 MHz, DMSO-*d*_6_+CDCl_3_): *δ* 178.4, 143.5, 135.9, 135.6, 135.2, 129.9, 129.7, 129.2, 128.7, 128.4, 127.0, 125.8, 124.5, 123.9, 122.8, 122.1, 119.8, 117.4, 110.1, 110.0, 105.6, 100.6, 73.9, 67.9, 55.0, 49.2, 33.2, 30.1, 29.5, 9.9.

*1′-Ethyl-2′-(1H-indol-3-yl)-1′-nitro-1′,5′,6′,10b’-tetrahydro-2′H-spiro[indoline-3,3′-pyrrolo[2,1-a]isoquinolin]-2-one* **(6r).** Brown solid; 86% yield; >99: 1 dr; mp 230–234 °C; IR(KBr) 735, 746, 1339, 1473, 1533, 1697, 1734, 3197, 3275, 3523, 3712, 3853 cm^−1^; HRMS(ESI) calcd for C_29_H_26_N_4_O_3_ [M+H]^+^ 479.2078, found 479.2084; ^1^H-NMR (400 MHz, DMSO-*d*_6_+CDCl_3_): *δ* 10.88 (s, 1H, NH), 10.42 (s, 1H, NH), 7.43 (d, *J* = 7.2 Hz, 1H, ArH), 7.25 (t, *J* = 7.6 Hz, 2H, ArH), 7.13–7.10 (m, 6H, ArH), 7.00–6.01 (m, 2H, ArH), 6.87 (t, *J* = 14.8 Hz, 1H, ArH), 6.67 (d, *J* = 7.6 Hz, 1H, ArH), 5.27 (d, *J* = 9.2 Hz, 2H, CH), 3.03–2.94 (m, 1H, CH_2_), 2.69–2.62 (m, 4H, CH_2_), 2.09–2.04 (m, 1H, CH_2_), 0.79 (t, *J* = 14.4 Hz, 3H, CH_3_); ^13^C-NMR (100 MHz, DMSO-*d*_6_+CDCl_3_): *δ* 178.6, 143.3, 135.5, 135.4, 135.2, 129.7, 129.5, 128.7, 128.4, 126.8, 125.6, 124.4, 123.8, 122.7, 121.8, 119.5, 117.2, 111.7, 109.9, 106.4, 100.6, 74.0, 68.0, 60.1, 55.4, 42.7, 30.1, 29.5, 21.1, 14.4, 9.8.

*2′-(2-Methyl-1H-indol-3-yl)-1′-nitro-1′,5′,6′,10b’-tetrahydro-2′H-spiro[indoline-3,3′-pyrrolo[2,1-a]isoquinolin]-2-one* **(6s).** Brown solid; 78% yield; >99: 1 dr; mp 215–224 °C; IR(KBr) 741, 1339, 1458, 1554, 1683, 1705, 3367, 3524, 3711, 3751, 3820 cm-1; HRMS(ESI) calcd for C_28_H_24_N_4_O_3_ [M+H]^+^ 465.1921, found 465.1926; ^1^H-NMR (400 MHz, DMSO-*d*_6_): *δ* 10.95 (s, 1H, NH), 10.06 (s, 1H, NH), 7.59 (d, *J* = 7.2 Hz, 1H, ArH), 7.51 (s, 2H, ArH), 7.32–7.14 (m, 5H, ArH), 6.98 (t, *J* = 14.4 Hz, 1H, ArH), 6.90 (d, *J* = 6.8 Hz, 1H, ArH), 6.66 (d, *J* = 7.6 Hz, 1H, ArH), 6.46 (t, *J* = 11.2 Hz, 1H, ArH), 5.99 (d, *J* = 6.4 Hz, 1H, CH), 4.54 (d, *J* = 4.4 Hz, 1H, CH), 4.07–4.00 (m, 1H, CH), 3.00–2.94 (m, 1H, CH_2_), 2.71 (d, *J* = 6.8 Hz, 3H, CH_2_), 2.51 (s, 1H, CH_3_), 1.83 (s, 3H, CH_3_); ^13^C-NMR (100 MHz, DMSO-*d*_6_): *δ* 177.7, 143.6, 135.6, 135.5, 134.9, 133.7, 130.2, 129.6, 128.3, 127.3, 126.2, 125.3, 124.7, 122.7, 120.7, 119.0, 111.0, 110.0, 103.1, 91.0, 74.9, 63.7, 60.3, 54.3, 42.8, 21.2, 14.6, 11.8.

*8′-Bromo-2′-(1H-indol-3-yl)-1′-nitro-1′,5′,6′,10b’-tetrahydro-2′H-spiro[indoline-3,3′-pyrrolo[2,1-a]isoquinolin]-2-one* **(6t).** Yellow solid; 93% yield; >99: 1 dr; mp >320 °C; IR(KBr) 754, 1338, 1489, 1552, 1683, 2827, 2927, 3047, 3197, 3286, 3348, 3462, 3566, 3711, 3801 cm^−1^; HRMS(ESI) calcd for C_27_H_21_BrN_4_O_3_ [M+H]^+^ 529.0870, found 529.0862; ^1^H-NMR (400 MHz, DMSO-*d*_6_+CDCl_3_): *δ* 11.04 (s, 1H, NH), 11.02 (s, 1H, NH), 7.66 (d, *J* = 7.2 Hz, 1H, ArH), 7.49 (d, *J* = 2.4 Hz, 1H, ArH), 7.34 (d, *J* = 6.4 Hz, 2H, ArH), 7.27–7.21 (m, 3H, ArH), 7.18–7.15 (m, 1H, ArH), 6.94 (t, *J* = 14.8 Hz, 1H, ArH), 6.67 (t, *J* = 15.2 Hz, 1H, ArH), 6.60 (d, *J* = 7.6 Hz, 2H, ArH), 6.32–6.30 (m, 1H, CH), 5.77 (d, *J* = 6.8 Hz, 1H, CH), 4.69 (d, *J* = 4.4 Hz, 1H, CH), 3.04–2.95 (m, 1H, CH_2_), 2.72 (s, 1H, CH_2_), 2.70–2.64 (m, 2H, CH_2_); ^13^C-NMR (100 MHz, DMSO-*d*_6_+CDCl_3_): *δ* 176.9, 143.6, 138.2, 136.1, 133.0, 132.0, 130.0, 128.9, 128.2, 127.2, 127.0, 124.7, 124.5, 122.7, 121.4, 120.3, 119.0, 118.1, 111.7, 110.0, 108.6, 93.1, 74.8, 62.8, 52.5, 42.3, 29.8.

*2′-(1H-Indol-3-yl)-1′, 9′-dinitro-1′,5′,6′,10b’-tetrahydro-2′H-spiro[indoline-3,3′-pyrrolo[2,1-a]isoquinolin]-2-one* **(6u).** Yellow solid; 85% yield; >99: 1 dr; mp 287–289 °C; IR(KBr) 1350, 1472, 1519, 1683, 1717, 2320, 3365, 3444, 3566, 3674, 3734, 3799, 3838 cm^−1^; HRMS(ESI) calcd for C_27_H_21_N_5_O_5_ [M+H]^+^ 496.1615, found 496.1621; ^1^H-NMR (400 MHz, DMSO-*d*_6_+CDCl_3_): *δ* 10.83 (s, 1H, NH), 9.93 (s, 1H, NH), 8.22 (s, 1H, ArH), 8.03 (t, *J* = 8.4 Hz, 1H, ArH), 8.02 (s, 1H, ArH), 7.86 (s, 1H, ArH), 7.70–7.16 (m, 4H, ArH), 6.97 (t, *J* = 14.8 Hz, 1H, ArH), 6.74–6.60 (m, 3H, ArH), 6.41–6.38 (m, 1H, ArH), 5.95 (d, *J* = 6.4 Hz, 1H, CH), 4.79 (d, *J* = 4.4 Hz, 1H, CH), 3.18–3.13 (m, 1H, CH_2_), 2.83 (d, *J* = 10.0 Hz, 1H, CH_2_), 2.78 (d, *J* = 8.6 Hz, 2H, CH_2_); ^13^C-NMR (100 MHz, DMSO-*d*_6_+CDCl_3_): *δ* 177.0, 145.9, 143.8, 143.5, 136.1, 135.0, 130.5, 130.0, 127.8, 126.9, 124.4, 122.7, 121.8, 121.5, 120.4, 119.1, 118.1, 111.6, 110.1, 108.4, 92.9, 74.7, 62.8, 52.4, 41.9, 30.3.

*1′-Ethyl-2′-(1-methyl-1H-indol-3-yl)-1′-nitro-1′,5′,6′,10b’-tetrahydro-2′H-spiro[indoline-3,3′-pyrrolo[2,1-a]isoquinolin]-2-one* **(6v).** White solid; 94% yield; >99: 1 dr; mp 271–273 °C; IR (KBr) 741, 1030, 1204, 1302, 1375, 1491, 1551, 1699, 2839, 2920, 3347, 3431 cm^−1^; HRMS(ESI) calcd for C_29_H_26_N_4_O_4_ [M+H]^+^ 495.2027, found 495.2033; ^1^H-NMR (400 MHz, DMSO-*d*_6_): *δ* 9.96 (s, 1H, NH), 7.55 (s, 1H, ArH), 7.33 (d, *J* = 8.4 Hz, 1H, ArH), 7.26 (s, 1H, ArH), 7.24–7.20 (m, 3H, ArH), 7.18 (t, *J* = 9.2 Hz, 1H, ArH), 7.02–7.01 (m, 1H, ArH), 6.86–6.83 (m, 1H, ArH), 6.77 (t, *J* = 8.0 Hz, 1H, ArH), 6.67 (s, 1H, ArH), 6.54 (s, 1H, CH), 6.36–6.33 (m, 1H, CH), 5.77 (d, *J* = 6.8 Hz, 1H, CH), 4.65 (d, *J* = 4.8 Hz, 1H, CH), 3.83 (s, 3H, CH_3_), 3.75 (s, 3H, CH_2_), 3.01 (s, 1H, CH_2_), 2.70 (s, *J* = 5.2 Hz, 1H, CH_3_), 2.67 (s, 1H, CH_3_), 2.63 (s, 1H, CH_3_); ^13^C-NMR (100 MHz, DMSO-*d*_6_): *δ* 176.8, 155.9, 136.8, 136.6, 135.4, 133.7, 129.6, 129.1, 127.4, 127.2, 126.2, 125.0, 121.7, 119.3, 118.4, 115.0, 111.1, 110.6, 110.2, 108.0, 93.2, 63.2, 60.3, 56.1, 52.4, 40.1, 40.0, 39.5, 33.1.

*5-Bromo-2′-(1-methyl-1H-indol-3-yl)-1′-nitro-1′,5′,6′,10b’-tetrahydro-2′H-spiro[indoline-3,3′-pyrrolo[2,1-a]isoquinolin]-2-one* **(6w).** Orange solid; 92% yield; >99: 1 dr; mp 264–266 °C; IR (KBr) 741, 908, 1204, 1375, 1481, 1558, 1612, 1718, 2839, 2920, 3246, 3358 cm^−1^; HRMS(ESI) calcd for C_28_H_23_BrN_4_O_3_ [M+H]^+^ 543.1026, found 543.1020; ^1^H-NMR (400 MHz, DMSO-*d*_6_): *δ* 10.29 (s, 1H, NH), 7.62 (d, *J* = 8 Hz, 1H, ArH), 7.56 (s, 1H, ArH), 7.41–7.36 (m, 1H, ArH), 7.27 (s, 1H, ArH), 7.26–7.21 (m, 1H, ArH), 7.20–7.15 (m, 3H, ArH), 7.05 (t, *J* = 15.2 Hz, 1H, ArH), 6.79 (s, 1H, ArH), 6.76 (t, *J* = 6.8 Hz, 1H, ArH), 6.65 (s, 1H, CH), 6.38–6.35 (m, 1H, CH), 5.76 (d, *J* = 6.8 Hz, 1H, CH), 4.67 (d, *J* = 4.8 Hz, 1H, CH), 3.76 (s, 3H, CH_2_), 2.97–2.90 (m, 1H, CH_2_), 2.71 (d, *J* = 4.4 Hz, 1H, CH_3_), 2.65 (s, 1H, CH_3_), 2.62 (t, *J* = 3.2 Hz, 1H, CH_3_); ^13^C-NMR (100 MHz, DMSO-*d*_6_): *δ* 176.6, 145.3, 136.6, 135.3, 133.5, 129.6, 129.3, 127.7, 126.7, 126.2, 125.6, 125.1, 122.9, 121.8, 119.3, 118.1, 112.9, 110.3, 107.7, 93.0, 63.3, 60.2, 52.2, 42.8, 40.2, 40.0, 39.3, 33.1.

*7-Bromo-2′-(1-methyl-1H-indol-3-yl)-1′-nitro-1′,5′,6′,10b’-tetrahydro-2′H-spiro[indoline-3, 3′-pyrrolo[2, 1-a]isoquinolin]-2-one* **(6x).** Orange solid; 86% yield; >99: 1 dr; mp 285–287 °C; IR (KBr) 741, 1125, 1190, 1331, 1458, 1549, 1628, 1699, 2835, 2918, 3200, 3221 cm^−1^; HRMS(ESI) calcd for C_28_H_23_BrN_4_O_3_ [M+H]^+^ 543.1026, found 543.1031; ^1^H-NMR (400 MHz, DMSO-*d*_6_): *δ* 10.12 (s, 1H, NH), 7.66 (s, 1H, ArH), 7.54 (s, 1H, ArH), 7.43 (s, 1H, ArH), 7.41 (d, *J* = 5.2 Hz, 1H, ArH), 7.31 (s, 1H, ArH), 7.29 (s, *J* = 0.8 Hz, 1H, ArH), 7.26 (s, 1H, ArH), 7.23–7.18 (m, 1H, ArH), 7.02 (t, *J* = 15.2 Hz, 1H, ArH), 6.72 (s, 1H, ArH), 6.60 (s, *J* = 7.6 Hz, 1H, ArH), 6.54 (d, *J* = 8 Hz, 1H, CH), 6.40–6.37 (m, 1H, CH), 5.73 (d, *J* = 6.8 Hz, 1H, CH), 4.65 (d, *J* = 4.4 Hz, 1H, CH), 3.74 (s, 3H, CH_2_), 2.97–2.89 (m, 1H, CH_2_), 2.73 (s, 1H, CH_3_), 2.67 (t, *J* = 10.4 Hz, 1H, CH_3_), 2.63 (t, *J* = 7.6 Hz, 1H, CH_3_); ^13^C-NMR (100 MHz, DMSO-*d*_6_): *δ* 176.8, 143.6, 138.4, 136.6, 133.1, 132.1, 130.3, 129.1, 128.1, 127.4, 124.7, 122.9, 121.7, 120.3, 119.2, 118.2, 110.2, 110.1, 107.9, 93.0, 62.9, 52.4, 40.2, 40.0, 39.6, 33.1, 29.6, 14.6.

*2′-(1-Methyl-1H-indol-3-yl)-1′-nitro-1-phenyl-1′,5′,6′,10b’-tetrahydro-2′H-spiro[indoline-3, 3′-pyrrolo[2, 1-a]isoquinolin]-2-one* **(6y).** Orange solid; 87% yield; >99: 1 dr; mp 277–279 °C; IR (KBr) 741, 908, 1203, 1302, 1375, 1551, 1612, 1701, 2810, 2920, 3246, 3358 cm^−1^; HRMS(ESI) calcd for C_34_H_28_N_4_O_3_ [M+H]^+^ 541.2234, found 541.2240; ^1^H-NMR (400 MHz, DMSO-*d*_6_): *δ* 7.81–7.79 (m, 1H, ArH), 7.63 (s, 1H, ArH), 7.39 (s, Hz, 1H, ArH), 7.38–7.33 (m, 4H, ArH), 7.31 (s, 1H, ArH), 7.30 (m, 1H, ArH), 7.25 (s, 1H, ArH), 7.24–7.18 (m, 1H, ArH), 7.06 (s, 1H, ArH), 6.68 (s, 1H, ArH), 6.64 (s, 1H, ArH), 6.48 (s, 1H, ArH), 6.47 (t, *J* = 6.4 Hz, 1H, ArH), 6.45 (t, *J* = 2.8 Hz, 1H, ArH), 6.43–6.41 (m, 1H, CH), 6.34 (s, 1H, CH), 5.79 (d, *J* = 6.8 Hz, 1H, CH), 4.76 (d, *J* = 4 Hz, 1H, CH), 3.76 (s, 3H, CH_2_), 2.99 (s, 1H, CH_2_), 2.85 (s, 1H, CH_3_), 2.75 (s, 1H, CH_3_), 2.73 (s, 1H, CH_3_); ^13^C-NMR (100 MHz, DMSO-*d*_6_): *δ* 174.5, 145.0, 136.8, 135.4, 133.9, 133.4, 130.4, 129.9, 129.6, 129.0, 128.5, 127.9, 127.3, 127.2, 126.6, 126.2, 125.4, 124.8, 124.3, 121.8, 119.1, 118.1, 110.1, 109.4, 108.0, 92.3, 63.9, 53.6, 42.8, 40.1, 39.9, 39.5, 33.1, 29.9.

*2′-(1-Methyl-1H-indol-3-yl)-1′,9′-dinitro-1′,5′,6′,10b’-tetrahydro-2′H-spiro[indoline-3,3′-pyrrolo[2,1-a]isoquinolin]-2-one* **(6z).** Orange solid; 85% yield; >99: 1 dr; mp >320 °C; IR (KBr) 741, 1125, 1190, 1341, 1458, 1518, 1557, 1628, 1699, 2835, 2918, 3110 cm^−1^; HRMS(ESI) calcd for C_28_H_23_N_5_O_5_ [M+H]^+^ 510.1772, found 510.1779; ^1^H-NMR (400 MHz, DMSO-*d*_6_): *δ* 10.11 (s, 1H, NH), 8.32 (s, 1H, ArH), 8.21 (d, *J* = 86 Hz, 1H, ArH), 8.08 (s, 1H, ArH), 8.07 (s, 1H, ArH), 7.67 (d, *J* = 7.2 Hz, 1H, ArH), 7.28 (s, 1H, ArH), 7.23 (s, 1H, ArH), 7.19 (s, 1H, ArH), 7.04–7.00 (m, 1H, ArH), 6.72–6.78 (m, 1H, ArH), 6.62 (s, 1H, ArH), 6.61–6.59 (m, 1H, CH), 6.50 (s, 1H, CH), 5.85 (d, *J* = 6.8 Hz, 1H, CH), 4.69 (d, *J* = 4.4 Hz, 1H, CH), 3.76 (s, 3H, CH_2_), 3.07–3.04 (m, 1H, CH_2_), 2.84 (s, 1H, CH_3_), 2.73 (s, 1H, CH_3_), 2.66 (t, *J* = 11.2 Hz, 1H, CH_3_); ^13^C-NMR (100 MHz, DMSO-*d*_6_): *δ* 176.6, 145.9, 144.2, 143.7, 136.6, 135.2, 131.1, 130.4, 129.2, 128.2, 127.3, 124.7, 123.0, 122.1, 121.7, 120.9, 119.2, 118.3, 110.2, 110.1, 108.0, 92.6, 62.9, 52.3, 40.2, 40.0, 39.6, 39.4.

### 4.4. General Procedure for the Synthesis of Compounds ***8***

A mixture of isatins (**1**, 0.5 mmol), THIQs (**5**, 0.5 mmol), and ethyl (*Z*)-2-(2-oxoindolin-3-ylidene) acetates (**7**, 0.5 mmol) in MeOH was stirred under reflux conditions for 12 h and indicated by TLC. Then, the reaction mixture was allowed to cool to room temperature and extracted with dichloromethane (3×50 mL). The combined organic layers were washed with saturated brine solution (20 mL), followed by drying over MgSO_4_ and evaporating in vacuo. The crude product was purified by column chromatography to give the pure title compounds.

*Ethyl 2,2″-dioxo-1′,5′,6′,10b’-tetrahydrodispiro[indoline-3,2′-pyrrolo[2,1-a]isoquinoline-3′,3″-indoline]-1′-carboxylate* **(8a).** Orange solid; 66% yield; 25: 1 dr; mp > 320 °C; IR (KBr) 1190, 1292, 1341, 1474, 1618, 1709, 2345, 2372, 3210 cm^−1^; HRMS(ESI) calcd for C_29_H_25_N_3_O_4_ [M+H]^+^ 480.1918, found 480.1914; ^1^H-NMR (400 MHz, DMSO-*d*_6_): *δ* 10.77 (s, 1H, NH), 10.60 (s, 1H, NH), 7.95 (d, *J* = 7.2 Hz, 1H, ArH), 7.32 (s, 1H, ArH), 7.30 (t, *J* = 15.2 Hz, 3H, ArH), 7.27 (s, 1H, ArH), 7.08–7.03 (m, 3H, ArH), 6.97–6.92 (m, 2H, ArH), 6.89 (d, *J* = 8.0 Hz, 1H, ArH), 6.34 (d, *J* = 7.6 Hz, 1H, ArH), 5.51 (s, 1H, CH_2_), 4.06 (s, 1H, CH), 3.50–3.45 (m, 2H, CH_2_), 2.83 (d, *J* = 6.4 Hz, 1H, CH), 2.65 (t, *J* = 16.0 Hz, 2H, CH_2_), 1.21 (s, 1H, CH_3_), 0.44 (t, *J* = 14.4 Hz, 2H, CH_3_); ^13^C-NMR (100 MHz, DMSO-*d*_6_): *δ* 179.4, 177.3, 167.9, 144.0, 142.9, 135.7, 135.0, 132.0, 130.3, 129.4, 129.2, 127.5, 126.8, 126.1, 125.8, 124.6, 123.3, 122.4, 110.0, 109.8, 70.5, 68.6, 62.7, 60.5, 57.9, 42.2, 40.5, 29.7, 13.3.

*1″-Methyl-2, 2″-dioxo-1′,5′,6′,10b’-tetrahydrodispiro[indoline-3, 2′-pyrrolo[2,1-a]isoquinoline-3′,3″-indoline]-1′-carboxylate* **(8b).** Pale yellow solid; 85% yield; >99: 1 dr; mp 294–296 °C; IR (KBr) 1211, 1352, 1373, 1471, 1616, 1653, 1699, 1717, 1869, 2345 cm^−1^; HRMS(ESI) calcd for C_30_H_27_N_3_O_4_ [M+H]^+^ 494.2074, found 494.2083; ^1^H-NMR (400 MHz, DMSO-*d*_6_): *δ* 10.78 (s, 1H, NH), 7.99 (s, 1H, ArH), 7.97–7.39 (m, 1H, ArH), 7.35–7.28 (m, 2H, ArH), 2.28–7.13 (m, 1H, ArH), 7.10–7.04 (m, 4H, ArH), 6.95 (t, *J* = 15.6 Hz, 2H, ArH), 6.35 (d, *J* = 7.6 Hz, 1H, ArH), 5.54 (s, 1H, CH), 4.09 (s, 1H, CH), 3.47–3.39 (m, 2H, CH_2_), 3.22 (s, 3H, CH_3_), 2.87–2.80 (m, 1H, CH_2_), 2.78–2.50 (m, 3H, CH_2_), 0.43 (t, *J* = 14.4 Hz, 3H, CH_3_); ^13^C-NMR (100 MHz, DMSO-*d*_6_): *δ* 179.3, 175.5, 167.9, 145.3, 142.9, 135.6, 134.9, 131.9, 130.5, 129.5, 129.3, 126.9, 126.7, 126.2, 125.8, 124.3, 123.4, 123.2, 122.5, 109.9, 109.1, 70.4, 68.8, 63.0, 60.6, 58.0, 42.2, 29.6, 26.4, 13.3.

*2, 2″-Dioxo-1″-phenyl-1′,5′,6′,10b’-tetrahydrodispiro[indoline-3,2′-pyrrolo[2,1-a]isoquinoline-3′,3″-indoline]-1′-carboxylate* **(8c).** Pale yellow solid; 74% yield; >99: 1 dr; mp > 300 °C; IR (KBr) 1200, 1369, 1458, 1508, 1616, 1647, 1707, 1734, 2345, 3736 cm^−1^; HRMS(ESI) calcd for C_35_H_29_N_3_O_4_ [M+H]^+^ 556.2231, found 556.2234; ^1^H-NMR (400 MHz, DMSO-*d*_6_): *δ* 10.84 (s, 1H, NH), 7.88 (s, 1H, ArH), 7.66 (t, *J* = 16.0 Hz, 1H, ArH), 7.53 (t, *J* = 14.0 Hz, 3H, ArH), 7.49–7.44 (m, 1H, ArH), 7.38–7.32 (m, 2H, ArH), 7.30–7.20 (m, 1H, ArH), 7.09–7.04 (m, 3H, ArH), 7.02–6.95 (m, 2H, ArH), 6.81 (m, 1H, ArH), 6.37 (d, *J* = 8.0 Hz, 1H, ArH), 5.53 (s, 1H, CH), 4.18 (d, *J* = 6.4 Hz, 1H, CH), 3.54–3.41 (m, 2H, CH_2_), 2.93–2.83 (m, 2H, CH_2_), 2.80–2.51 (m, 2H, CH_2_), 0.45 (t, *J* = 14.0 Hz, 3H, CH_3_); ^13^C-NMR (100 MHz, DMSO-*d*_6_): *δ* 179.3, 175.0, 168.2, 145.0, 142.9, 135.6, 135.0, 134.7, 131.8, 130.6, 130.2, 129.5, 129.3, 128.6, 127.2, 126.9, 126.6, 126.2, 125.6, 124.9, 123.9, 123.3, 122.5, 110.0, 109.5, 70.3, 69.0, 63.7, 60.8, 57.8, 42.2, 40.5, 40.1, 29.7, 13.4.

*5″-Methyl-2,2″-dioxo-1′,5′,6′,10b’-tetrahydrodispiro[indoline-3,2′-pyrrolo[2,1-a]isoquinoline-3′,3″-indoline]-1′-carboxylate* **(8d).** Pale yellow solid; 64% yield; 34: 1 dr; mp > 300 °C; IR (KBr) 1196, 1474, 1497, 1616, 1707, 1736, 2345, 2371, 3198, 3566 cm^−1^; HRMS(ESI) calcd for C_30_H_27_N_3_O_4_ [M+H]^+^ 494.2074, found 494.2081; ^1^H-NMR (400 MHz, DMSO-*d*_6_): *δ* 10.77 (s, 1H, NH), 10.49 (d, *J* = 8.0 Hz, 1H, NH), 7.95 (d, *J* = 7.2 Hz, 1H, ArH), 7.29 (t, *J* = 15.2 Hz, 2H, ArH), 7.10 (s, 3H, ArH), 7.09 (s, 2H, ArH), 7.06–7.02 (m, 1H, ArH), 6.94 (t, *J* = 16.0 Hz, 1H, ArH), 7.77 (d, *J* = 8.0 Hz, 1H, ArH), 5.50 (s, 1H, ArH), 4.03 (t, *J* = 12.4 Hz, 1H, CH_2_), 2.88–2.80 (m, 1H, CH), 2.65 (d, *J* = 10.8 Hz, 2H, CH_3_), 2.63–2.56 (m, 2H, CH_2_), 2.55–2.51 (m, 2H, CH_3_), 2.30 (s, 3H, CH_2_), 0.44 (t, *J* = 14.0 Hz, 2H, CH_3_); ^13^C-NMR (100 MHz, DMSO-*d*_6_): *δ* 179.5, 177.2, 167.9, 142.9, 141.6, 135.8, 135.0, 132.0, 131.2, 130.6, 129.4, 129.2, 127.6, 127.5, 126.8, 126.1, 125.9, 125.1, 123.3, 122.4, 109.8, 70.5, 68.6, 62.7, 60.4, 57.8, 42.2, 29.7, 21.1, 13.3.

*5″-Methoxy-2,2″-dioxo-1′,5′,6′,10b’-tetrahydrodispiro[indoline-3,2′-pyrrolo[2,1-a]isoquinoline-3′,3″-indoline]-1′-carboxylate* **(8e).** Brown solid; 71% yield; 40: 1 dr; mp 297–299 °C; IR (KBr) 1190, 1288, 1491, 1616, 1701, 1734, 2345, 3167, 3190, 3736 cm^−1^; HRMS(ESI) calcd for C_30_H_27_N_3_O_5_ [M+H]^+^ 510.2023, found 510.2018; ^1^H-NMR (400 MHz, DMSO-*d*_6_): *δ* 10.78 (s, 1H, NH), 10.42 (s, 1H, NH), 7.96 (d, *J* = 7.2 Hz, 1H, ArH), 7.31–7.02 (m, 3H, ArH), 6.96–6.89 (m, 2H, ArH), 6.87–6.82 (m, 1H, ArH), 6.80 (s, 2H, ArH), 6.35 (d, *J* = 7.6 Hz, 1H, ArH), 5.49 (s, 1H, ArH), 3.74 (s, 3H, CH_3_), 3.49–3.46 (m, 2H, CH), 3.42 (d, *J* = 9.6 Hz, 2H, CH_2_), 2.90–2.81 (m, 1H, CH_2_), 2.66–2.51 (m, 3H, CH_2_), 0.45 (t, *J* = 14.4 Hz, 3H, CH_3_); ^13^C-NMR (100 MHz, DMSO-*d*_6_): *δ* 179.4, 177.1, 167.8, 155.4, 142.9, 137.2, 135.8, 135.0, 132.0, 129.5, 129.2, 138.9, 126.8, 126.1, 125.9, 123.3, 122.4, 115.1, 110.9, 110.6, 109.8, 70.8, 68.6, 62.7, 60.4, 57.7, 55.9, 40.5, 29.7, 13.3.

*5″-Fluoro-2,2″-dioxo-1′,5′,6′,10b′-tetrahydrodispiro[indoline-3,2′-pyrrolo[2,1-a]isoquinoline-3′,3″-indoline]-1′-carboxylate* **(8f).** Pale yellow solid; 77% yield; 25: 1 dr; mp > 300 °C; IR (KBr) 1192, 1211, 1296, 1474, 1491, 1616, 1705, 1734, 3210, 3566 cm^−1^; HRMS(ESI) calcd for C_29_H_24_FN_3_O_4_ [M+H]^+^ 498.1824, found 498.1828; ^1^H-NMR (400 MHz, DMSO-*d*_6_): *δ* 10.79 (s, 1H, NH), 10.64 (s, 1H, NH), 7.94–7.28 (m, 1H, ArH), 7.18–7.13 (m, 1H, ArH), 7.08–7.03 (m, 2H, ArH), 6.97–6.93 (m, 3H, ArH), 6.91–6.87 (m, 2H, ArH), 6.34 (d, *J* = 8.0 Hz, 1H, ArH), 5.48 (s, 1H, ArH), 4.04 (s, 2H, CH), 3.50–3.42 (m, 2H, CH_2_), 2.90–2.82 (m, 1H, CH_2_), 2.67–2.51 (m, 3H, CH_2_), 0.45 (t, *J* = 14.0 Hz, 3H, CH_3_); ^13^C-NMR (100 MHz, DMSO-*d*_6_): *δ* 179.3, 177.2, 167.8, 160.0, 157.3, 142.9, 140.2, 135.6, 134.9, 131.9, 129.5, 129.3, 129.2, 126.8, 123.3, 122.4, 116.9, 116.7, 112.4, 112.1, 111.0, 110.9, 109.9, 70.8, 68.5, 62.6, 60.5, 57.7, 13.3.

*6″-Bromo-2,2″-dioxo-1′,5′,6′,10b′-tetrahydrodispiro[indoline-3,2′-pyrrolo[2,1-a]isoquinoline-3′,3″-indoline]-1′-carboxylate* **(8g).** Pale yellow solid; 61% yield; >99: 1 dr; mp > 300 °C; IR (KBr) 1190, 1341, 1474, 1616, 1709, 1717, 1734, 2345, 3370, 3566 cm^−1^; HRMS(ESI) calcd for C_29_H_24_BrN_3_O_4_ [M+H]+ 557.0950, found 557.0955; ^1^H-NMR (400 MHz, DMSO-*d*_6_): *δ* 10.77 (d, *J* =8.8 Hz, 2H, NH), 7.91 (s, 1H, ArH), 7.89–7.28 (m, 2H, ArH), 7.27–7.24 (m, 3H, ArH), 7.22–7.03 (m, 1H, ArH), 6.97–6.94 (m, 1H, ArH), 6.34 (d, *J* =7.6 Hz, 1H, ArH), 5.46 (s, 1H, ArH), 4.04 (s, 1H, ArH), 3.51–3.41 (m, 2H, ArH), 2.85 (s, 2H, CH), 2.83 (t, *J* =15.6 Hz, 1H, CH_2_), 2.68 (m, 1H, CH_2_), 2.64–2.56 (m, 2H, CH_2_), 2.54–2.50 (m, 2H, CH_2_), 0.45 (t, *J* =14.4 Hz, 1H, CH_3_); ^13^C-NMR (100 MHz, DMSO-*d*_6_): *δ* 179.3, 177.1, 167.8, 145.6, 142.9, 135.6, 134.9, 131.8, 129.5, 129.3, 126.9, 126.6, 126.2, 125.7, 125.2, 123.3, 123.0, 122.5, 112.8, 110.0, 70.3, 68.6, 62.6, 60.6, 57.7, 42.2, 29.7, 21.1, 13.3.

*10b′-Methyl-2,2″-dioxo-1′,5′,6′,10b′-tetrahydrodispiro[indoline-3,2′-pyrrolo[2,1-a]isoquinoline-3′,3″-indoline]-1′-carboxylate* **(8h).** Purple solid; 60% yield; >99: 1 dr; mp > 300 °C; IR (KBr) 1213, 1323, 1458, 1474, 1616, 1653, 1676, 1719, 2345, 3566 cm^−1^; HRMS(ESI) calcd for C_30_H_27_N_3_O_4_ [M+H]^+^ 494.2074, found 494.2069; ^1^H-NMR (400 MHz, DMSO-*d*_6_): *δ* 11.02 (s, 1H, NH), 10.29 (s, 1H, NH), 8.07 (s, 1H, ArH), 7.68 (t, *J* = 16.3 Hz, 1H, ArH), 7.46–7.39 (m, 1H, ArH), 7.29–7.19 (m, 1H, ArH), 7.18–7.10 (m, 4H, ArH), 6.98 (t, *J* = 15.2 Hz, 1H, ArH), 6.76 (s, 1H, ArH), 7.74–6.65 (m, 1H, ArH), 6.27–6.24 (m, 1H, ArH), 5.83 (d, *J* = 6.8 Hz, 1H, ArH), 4.71 (t, *J* = 4.8 Hz, 1H, CH), 3.36 (d, *J* = 1.6 Hz, 3H, CH_2_), 3.07–3.01 (m, 1H, CH_2_), 2.76 (t, *J* = 16.4 Hz, 2H, CH_2_), 2.72–2.54 (m, 6H, CH_3_); ^13^C-NMR (100 MHz, DMSO-*d*_6_): *δ* 176.6, 142.6, 135.8, 134.9, 132.9, 132.5, 130.0, 129.0, 126.7, 126.5, 125.6, 124.6, 124.2, 123.8, 123.1, 121.2, 118.8, 117.6, 111.4, 108.0, 102.2, 92.7, 79.0, 78.6, 78.3, 75.3, 62.9, 52.5, 42.4, 29.6.

*8′,9′-Dimethoxy-2,2″-dioxo-1′,5′,6′,10b′-tetrahydrodispiro[indoline-3,2′-pyrrolo[2,1-a]isoquinoline-3′,3″-indoline]-1′-carboxylate* **(8i).** Yellow solid; 71% yield; >99: 1 dr; mp 280–282 °C; IR (KBr) 1105, 1142, 1215, 1341, 1373, 1474, 1522, 1618, 1701, 2345 cm^−1^; HRMS(ESI) calcd for C_31_H_29_N_3_O_6_ [M+H]^+^ 540.2129, found 540.2133; ^1^H-NMR (400 MHz, DMSO-*d*_6_): *δ* 10.72 (s, 1H, NH), 10.58 (s, 1H, NH), 7.93 (s, 1H, ArH), 7.91–7.08, (m, 3H, ArH), 7.08–7.04 (m, 2H, ArH), 6.94 (d, *J* = 7.6 Hz, 2H, ArH), 6.89 (d, *J* = 7.6 Hz, 1H, ArH), 6.62 (s, 1H, ArH), 5.86 (s, 1H, ArH), 5.44 (s, 1H, ArH), 4.07 (s, 1H, CH), 3.65–3.51 (m, 3H, CH_3_), 3.50–3.46 (m, 1H, CH_2_), 3.45 (t, *J* = 18.4 Hz, 1H, CH), 3.36 (s, 3H, CH_2_), 2.75–2.67 (m, 1H, CH_2_), 2.65–2.47 (m, 2H, CH_2_), 0.43 (t, *J* = 14.0 Hz, 3H, CH_3_); ^13^C-NMR (100 MHz, DMSO-*d*_6_): *δ* 179.6, 177.4, 168.0, 147.5, 146.8, 144.0, 142.9, 132.1, 130.3, 129.2, 127.6, 127.1, 126.9, 125.8, 124.8, 122.5, 122.3, 112.4, 110.0, 110.0, 106.7, 70.7, 68.8, 62.1, 60.4, 58.3, 55.7, 54.9, 42.2, 29.2, 13.3.

*5,7-Dimethyl-2,2″-dioxo-1′,5′,6′,10b′-tetrahydrodispiro[indoline-3, 2′-pyrrolo[2,1-a]isoquinoline-3′,3”-indoline]-1′-carboxylate* **(8j).** Pale yellow solid; 83% yield; >99: 1 dr; mp > 300 °C; IR (KBr) 752, 1032, 1159, 1184, 1204, 1285, 1346, 1474, 1622, 1716 cm^−1^; HRMS(ESI) calcd for C_31_H_29_N_3_O_4_ [M+H]^+^ 508.2231, found 508.2236; ^1^H-NMR (400 MHz, DMSO-*d*_6_): *δ* 10.70 (s, 1H, NH), 10.52 (s, 1H, NH), 7.61 (s, 1H, ArH), 7.30 (d, *J* = 8.0 Hz, 2H, ArH), 7.18 (d, *J* = 7.6 Hz, 2H, ArH), 7.05–6.94 (m, 2H, ArH), 6.93–6.88 (m, 1H, ArH), 6.36 (d, *J* = 8.0 Hz, 1H, ArH), 5.47 (s, 1H, ArH), 4.17–4.13 (m, 1H, CH_2_), 4.05 (d, *J* = 9.2 Hz, 1H, CH_2_), 3.51–3.43 (m, 2H, CH), 3.41 (d, 1H, CH_2_), 3.17 (d, *J* = 5.2 Hz, CH_2_), 2.87 (s, 2H, CH_2_), 2.84–2.63 (m, 3H, CH_3_), 2.58–2.24 (m, 3H, CH_3_), 0.43 (t, *J* = 14.0 Hz, 3H, CH_3_); ^13^C-NMR (100 MHz, DMSO-*d*_6_): *δ* 179.9, 177.2, 167.9, 144.0, 139.1, 135.9, 134.9, 131.7, 131.0, 130.6, 130.3, 129.4, 127.7, 126.7, 126.1, 124.6, 123.5, 122.4, 118.7, 110.0, 70.4, 68.6, 62.9, 60.3, 58.1, 49.1, 42.2, 29.7, 21.4, 16.9, 13.2.

## Figures and Tables

**Table 1:** Optimized reaction conditions *^a,b^*.

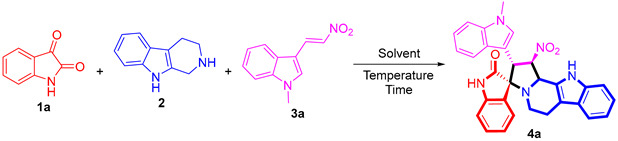				
Entry	Solvent	Temperature (°C)	Time (h)	Yield (%) *^b^*
1	EtOH	r.t. *^c^*	8	53
2	MeOH	r.t.	8	66
3	*^i^*PrOH	r.t.	8	47
4	Glycerol	r.t.	8	38
5	H_2_O	r.t.	8	<5
6	THF	r.t.	8	24
7	1,4-Dioxane	r.t.	8	49
8	Toluene	r.t.	8	47
9	CHCl_3_	r.t.	8	49
10	DCM	r.t.	8	42
11	DMSO	r.t.	8	51
12	DMF	r.t.	8	39
13	MeOH	40	8	74
14	MeOH	Reflux	8	84
**15**	**MeOH**	**Reflux**	**12**	**88**
16	MeOH	Reflux	24	89

*^a^* All reactions were carried out with **1a** (0.5 mmol), **2** (0.5 mmol), and **3a** (0.5 mmol) in the corresponding solvents (5 mL) at corresponding temperatures; *^b^* Isolated yields, >20: 1 dr by ^1^H NMR analysis via the signal of NH; *^c^* room temperature.

**Table 2:** The synthesis of functionalized *β*-tetrahydrocarboline-fused spirooxindoles **4** *^a,b,c^*.

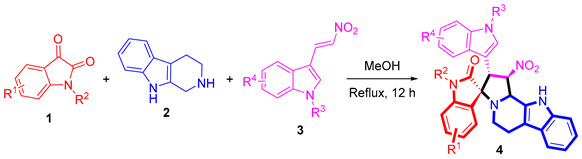
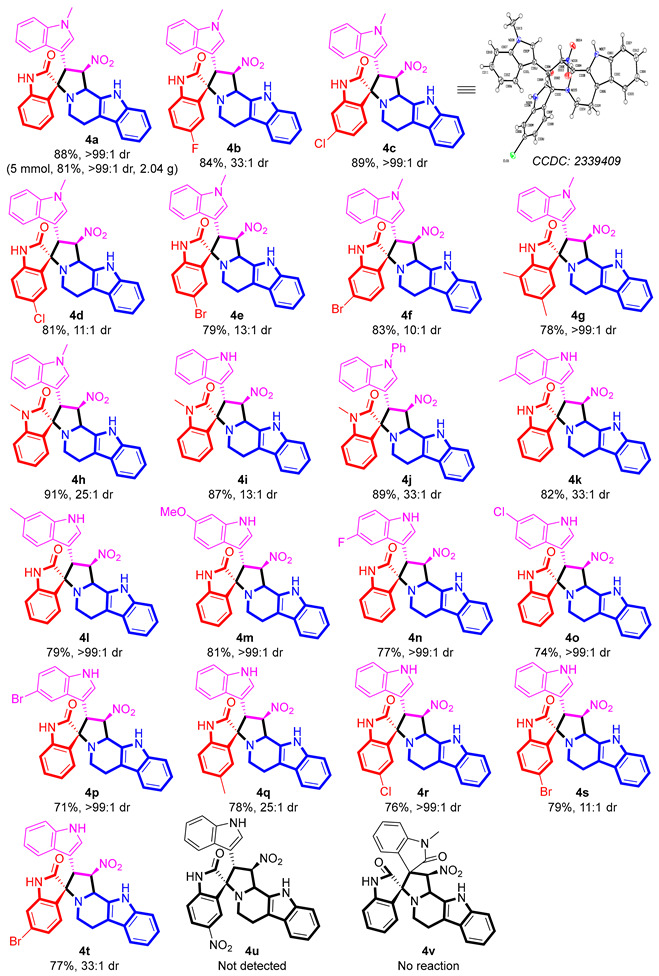

*^a^* All reactions were carried out with **1** (0.5 mmol), **2** (0.5 mmol), and **3** (0.5 mmol) in MeOH (5 mL) under reflux for 15 h; *^b^* isolated yields; *^c^* the dr values were determined by ^1^H NMR analysis via the signal of NH.

**Table 3:** The synthesis of functionalized spirooxindoles **6** *^a,b,^^c^*.

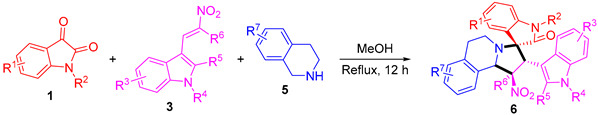
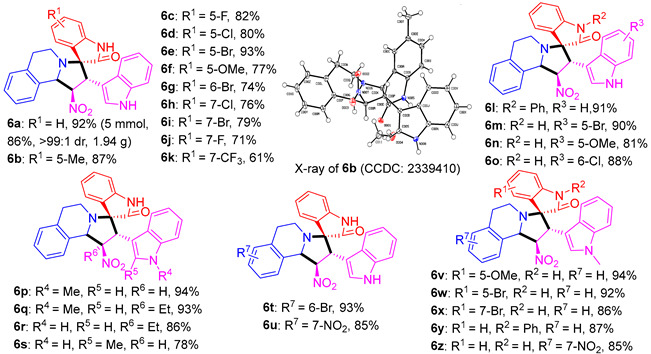

*^a^* All reactions were carried out with **1** (0.5 mmol), **3** (0.5 mmol), and **5** (0.5 mmol) in MeOH (5 mL) under reflux for 12 h; *^b^* Isolation of recrystallized yields; *^c^* All > 99: 1 dr, determined by ^1^H NMR analysis via the signal of NH.

**Table 4:** The synthesis of functionalized spirooxindoles **8** *^a,b,^^c^*.

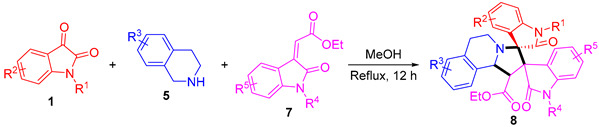
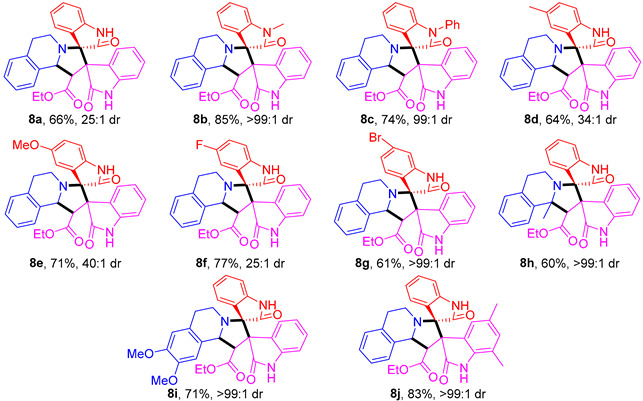

*^a^* All reactions were carried out with **1** (0.5 mmol), **5** (0.5 mmol), and **7** (0.5 mmol) in MeOH (5 mL) under reflux for 12 h; *^b^* Isolated yields; *^c^* The dr values was determined by ^1^H NMR analysis via the signal of NH.

## Data Availability

Data are contained within the article and Supplementary Materials.

## Supplementary Materials

The following supporting information can be downloaded at: https://www.mdpi.com/article/10.3390/molecules29081790/s1.
