# Supplementary material for: Diastereoselective Three-Component 1,3-Dipolar Cycloaddition to Access Functionalized β-Tetrahydrocarboline- and Tetrahydroisoquinoline-Fused Spirooxindoles
Source: Molecules. 2024 Apr 15;29(8):1790. doi: 10.3390/molecules29081790 (PMC11052326; doi:10.3390/molecules29081790)

Supplementary Materials

Diastereoselective Three-component 1,3-Dipolar Cycloaddition to Access Functionalized β-Tetrahydrocarboline and Tetrahydroisoquinoline fused Spirooxindoles

Yongchao Wang [1,*] , Yu Chen [1] , Shengli Duan [1] , Yiyang Cao[1] , Wenjin Sun [1],
Mei Zhang[1] , Delin Zhao [1] , Donghua Hu [1,*] and Jian-Wei Dong [2,*]

[1] Colleage of Vocational and Technical Education, Yunnan Normal University, Kunming 650092, China

[2] College of Chemistry and Environmental Science, Qujing Normal University, Qujing, 655011, China

[*] Correspondence:

Yongchao Wang (ycwang@ynnu.edu.cn; yongchaowang126@126.com)

Donghua Hu (hudonghua8888@126.com)

Jianwei Dong (jwdongyn@mail.qjnu.edu.cn)

Table of contents

1. ^{1}H and ^{13}C NMR spectras for compounds 4 ....S3

2. ^{1}H and ^{13}C NMR spectras for compounds 6 ....S23

3. ^{1}H and ^{13}C NMR spectras for compounds 8 ....S49

4. Single crystal X-ray diffraction study data of compound 4c S59

5. Single crystal X-ray diffraction study data of compound 6b S63

1. ^{1}H and ^{13}C NMR spectras for compounds 4

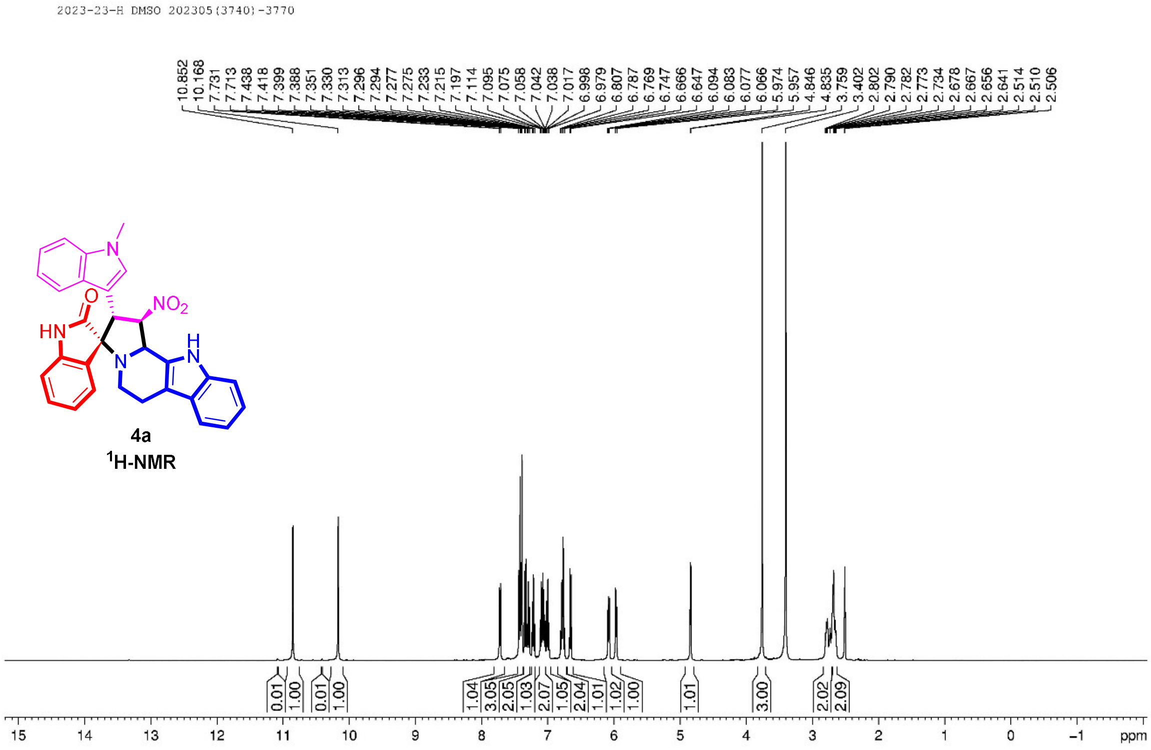

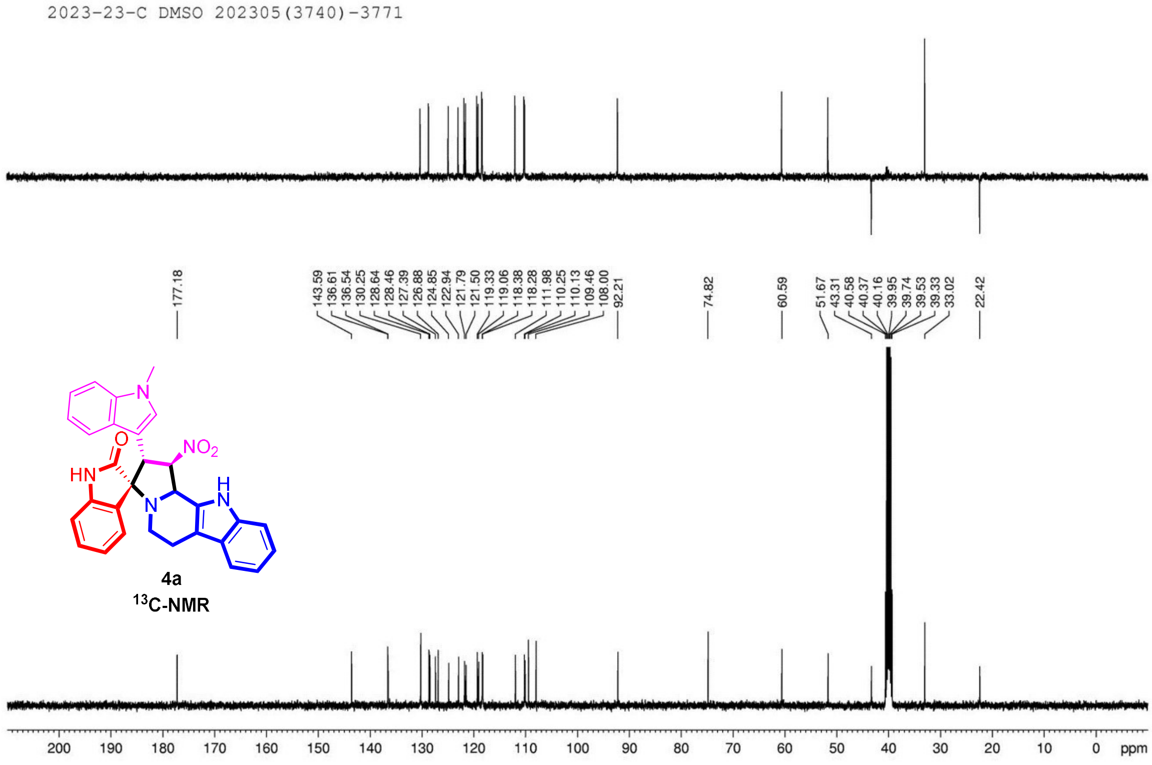

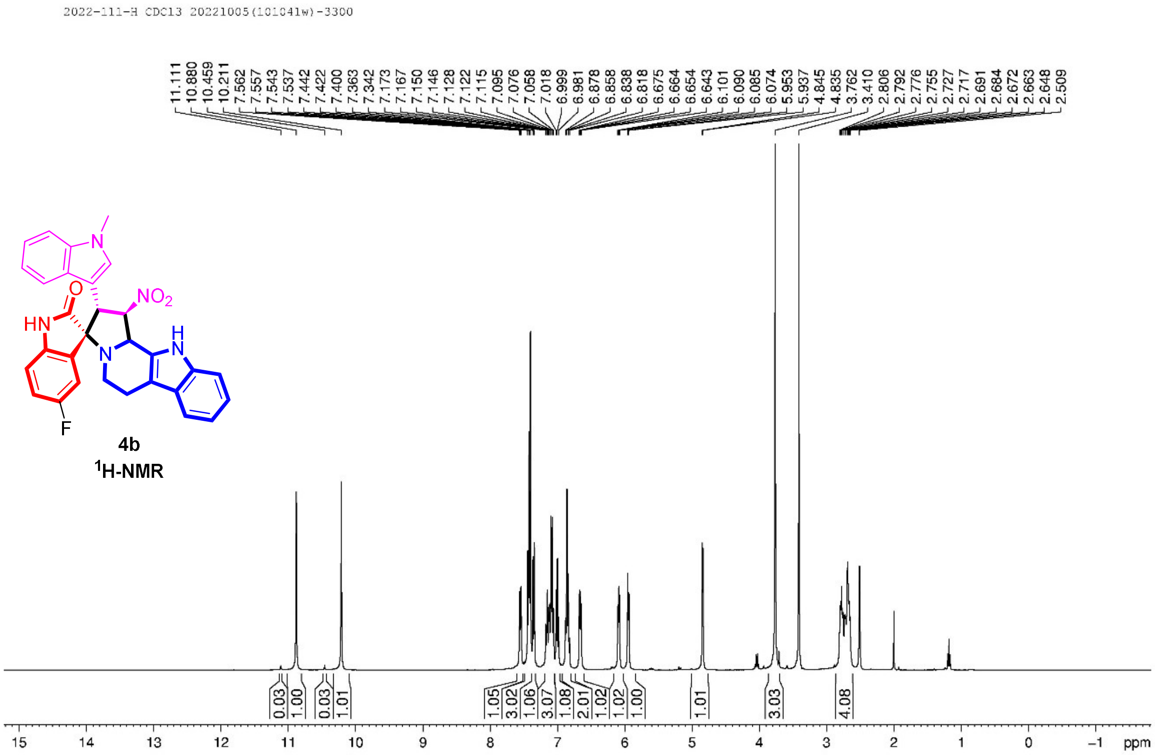
2022-111-H CDCl3 20221005(101041w)-3300
11.111
10.880
10.459
10.211
7.562
7.557
7.543
7.537
7.442
7.422
7.400
7.363
7.342
7.173
7.167
7.150
7.146
7.128
7.122
7.115
7.095
7.076
7.058
7.018
6.999
6.981
6.878
6.858
6.838
6.818
6.675
6.664
6.654
6.643
6.101
6.090
6.085
6.074
5.953
5.937
4.845
4.835
3.762
3.410
2.806
2.792
2.776
2.755
2.727
2.717
2.691
2.684
2.672
2.663
2.648
2.509
NO2
HN
O
N
H
N
F
4b
1H-NMR
0.03
1.00
0.03
1.01
1.05
3.02
1.06
3.07
1.08
2.01
1.02
1.00
1.01
3.03
4.08
15
14
13
12
11
10
9
8
7
6
5
4
3
2
1
0
−1
ppm

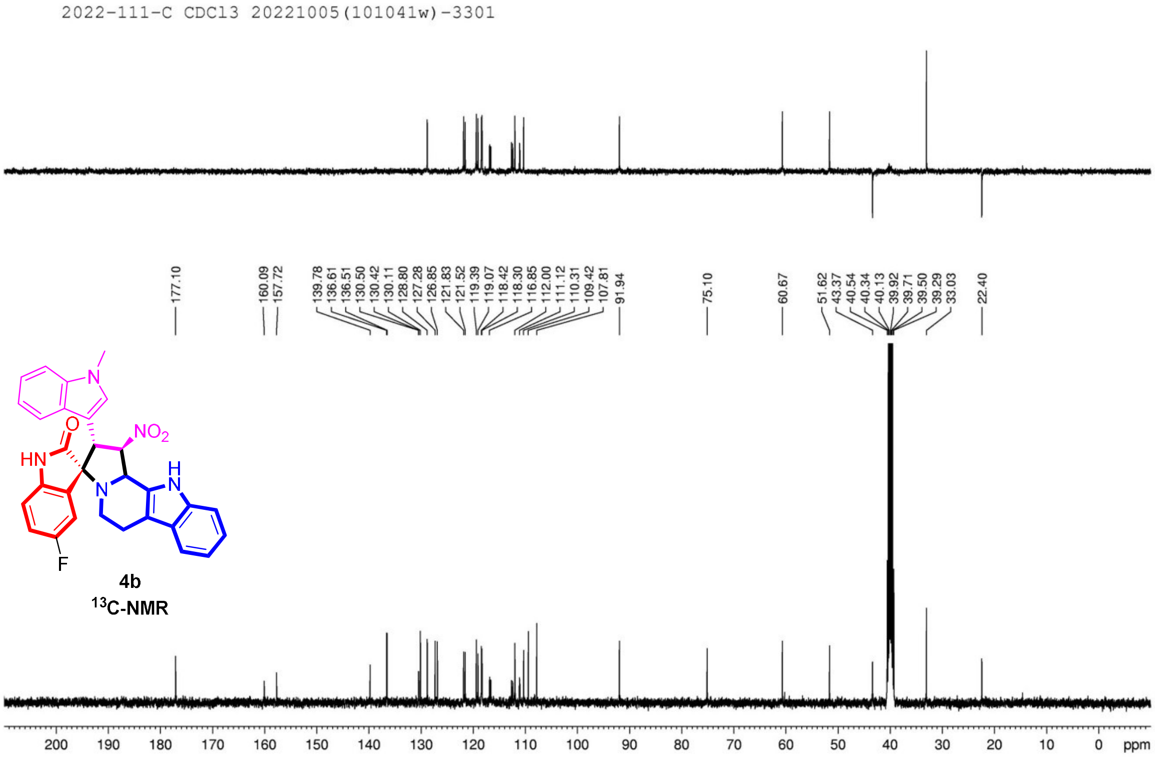
2022-111-C CDCl3 20221005(101041w)-3301
177.10
160.09
157.72
139.78
136.61
136.51
130.50
130.42
130.11
128.80
127.28
126.85
121.83
121.52
119.39
119.07
118.42
118.30
116.85
112.00
111.12
110.31
109.42
107.81
91.94
75.10
60.67
51.62
43.37
40.54
40.34
40.13
39.92
39.71
39.50
39.29
33.03
22.40
NO2
HN
O
N
H
N
F
4b
13C-NMR
200
190
180
170
160
150
140
130
120
110
100
90
80
70
60
50
40
30
20
10
0
ppm

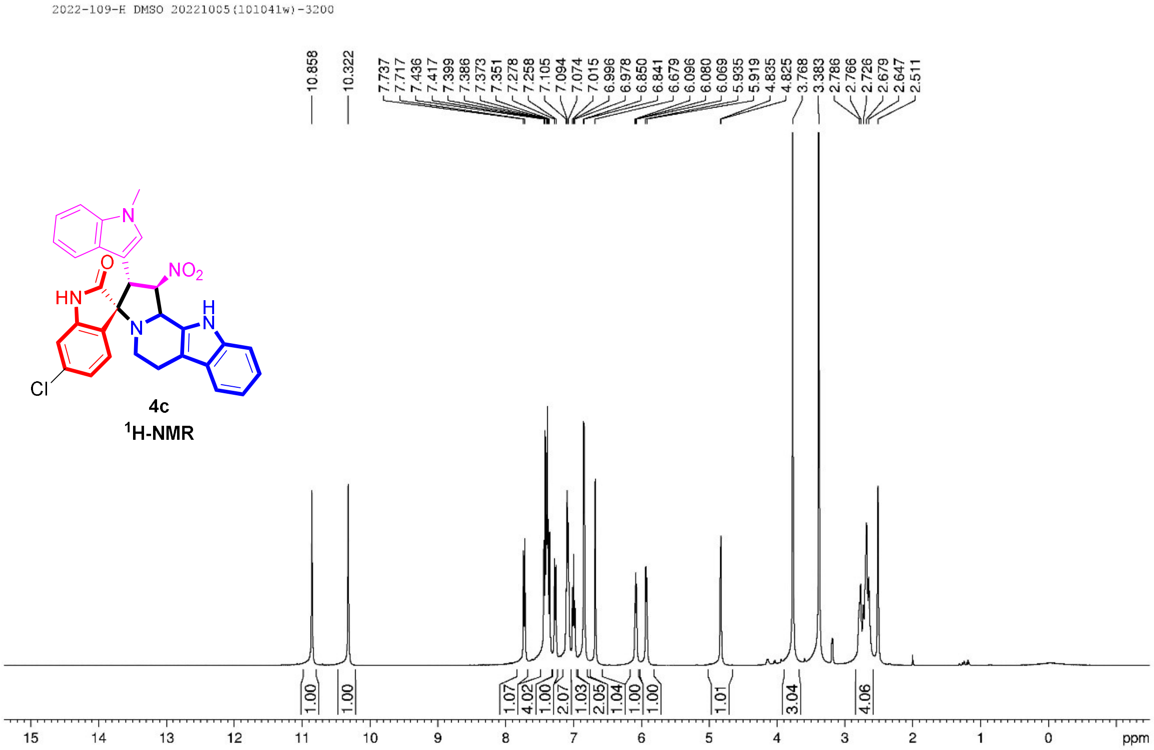

2022-109-E DMSO 20221005(101041w)-3200
10.858
10.322
7.737
7.717
7.436
7.417
7.399
7.386
7.373
7.351
7.278
7.258
7.105
7.094
7.074
7.015
6.996
6.978
6.850
6.841
6.679
6.096
6.080
6.069
5.935
5.919
4.835
4.825
3.768
3.383
2.786
2.766
2.726
2.679
2.647
2.511
NO2
HN
O
N
H
N
Cl
4c
1H-NMR
1.00
1.00
1.07
4.02
1.00
2.07
1.03
2.05
1.04
1.00
1.00
1.01
3.04
4.06
15
14
13
12
11
10
9
8
7
6
5
4
3
2
1
0
ppm

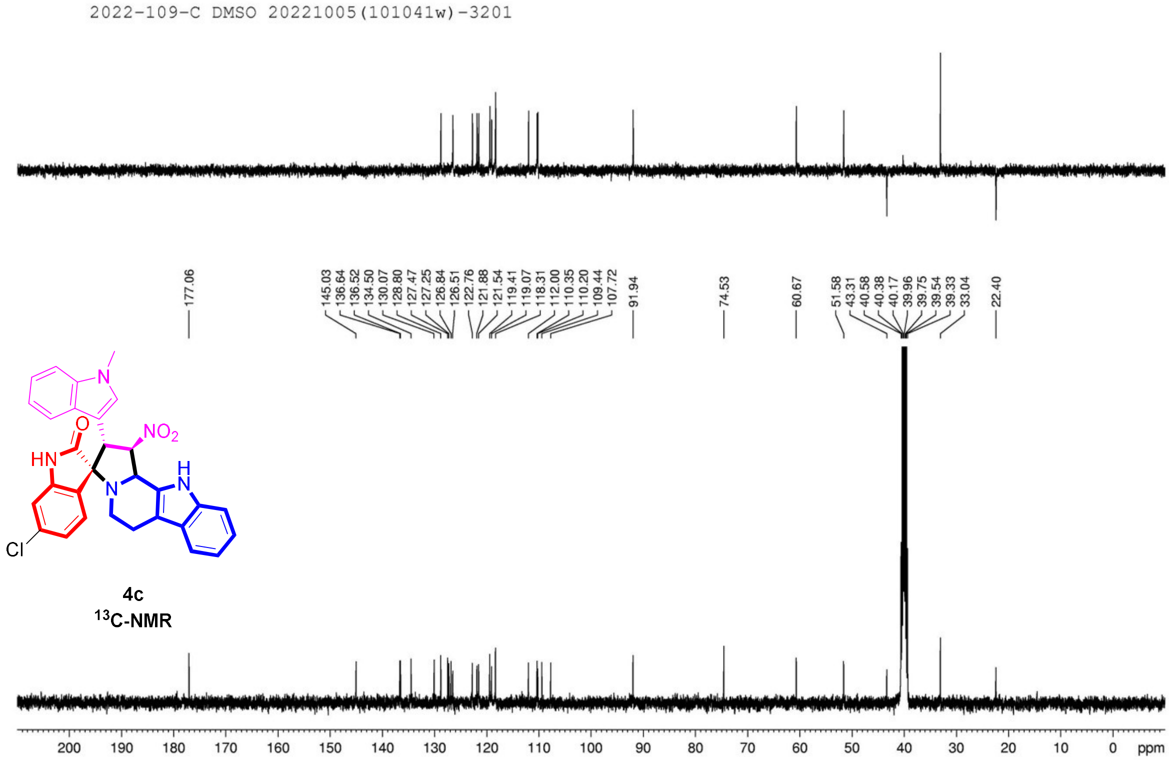

2022-109-C DMSO 20221005(101041w)-3201
177.06
145.03
136.64
136.52
134.50
130.07
128.80
127.47
127.25
126.84
126.51
122.76
121.88
121.54
119.41
119.07
118.31
112.00
110.35
110.20
109.44
107.72
91.94
74.53
60.67
51.58
43.31
40.58
40.38
40.17
39.96
39.75
39.54
39.33
33.04
22.40
NO2
HN
O
N
H
N
Cl
4c
13C-NMR
200
190
180
170
160
150
140
130
120
110
100
90
80
70
60
50
40
30
20
10
0
ppm

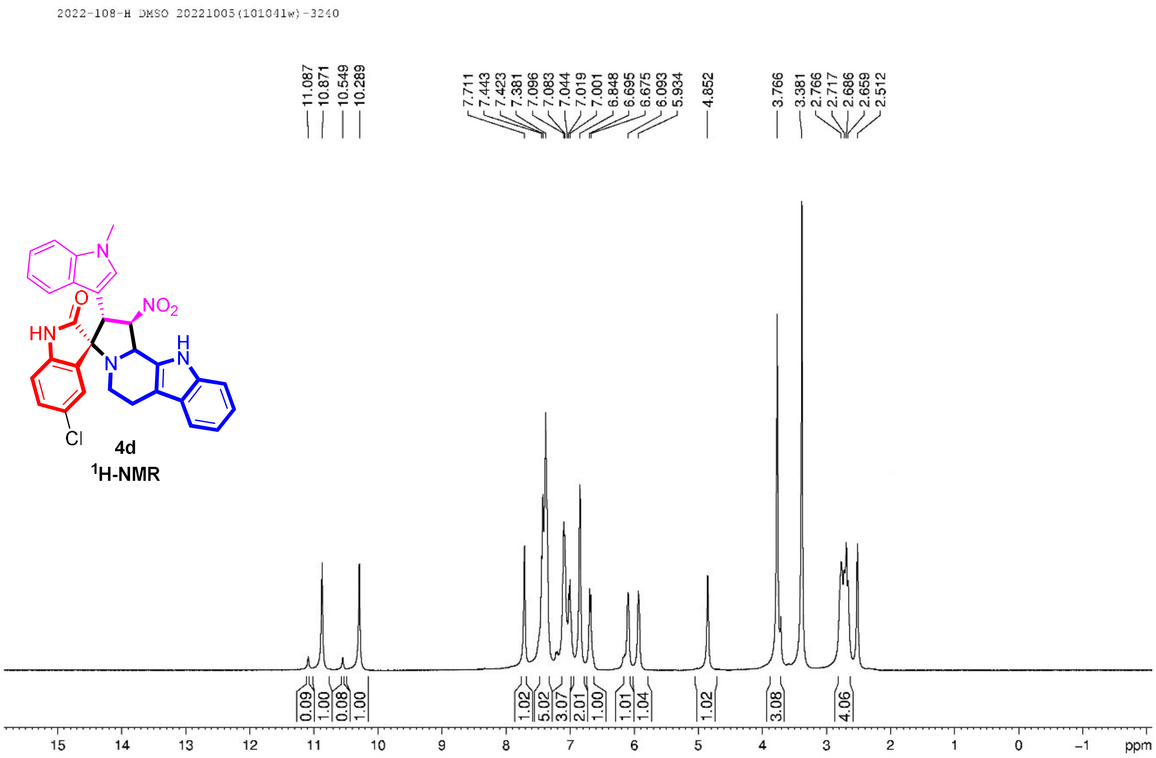
2022-108-H DMSO 20221005(101041w)-3240
11.087
10.871
10.549
10.289
7.711
7.443
7.423
7.381
7.096
7.083
7.044
7.019
7.001
6.848
6.695
6.675
6.093
5.934
4.852
3.766
3.381
2.766
2.717
2.686
2.659
2.512
NO2
HN
O
N
H
N
N
Cl
4d
1H-NMR
0.09
1.00
0.08
1.00
1.02
5.02
3.07
2.01
1.00
1.01
1.04
1.02
3.08
4.06
ppm

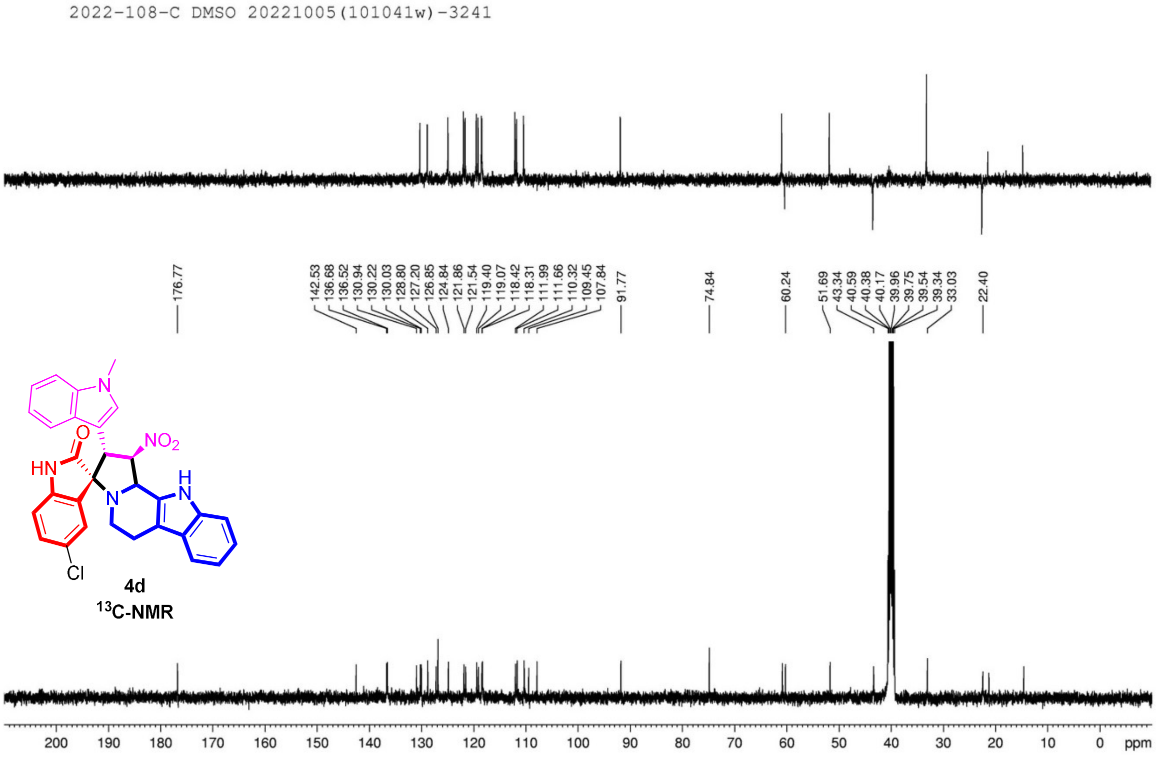
2022-108-C DMSO 20221005(101041w)-3241
176.77
142.53
136.68
136.52
130.94
130.22
130.03
128.80
127.20
126.85
124.84
121.86
121.54
119.40
119.07
118.42
118.31
111.99
111.66
110.32
109.45
107.84
91.77
74.84
60.24
51.69
43.34
40.59
40.38
40.17
39.96
39.75
39.54
39.34
33.03
22.40
NO2
HN
O
N
H
N
N
Cl
4d
13C-NMR
ppm

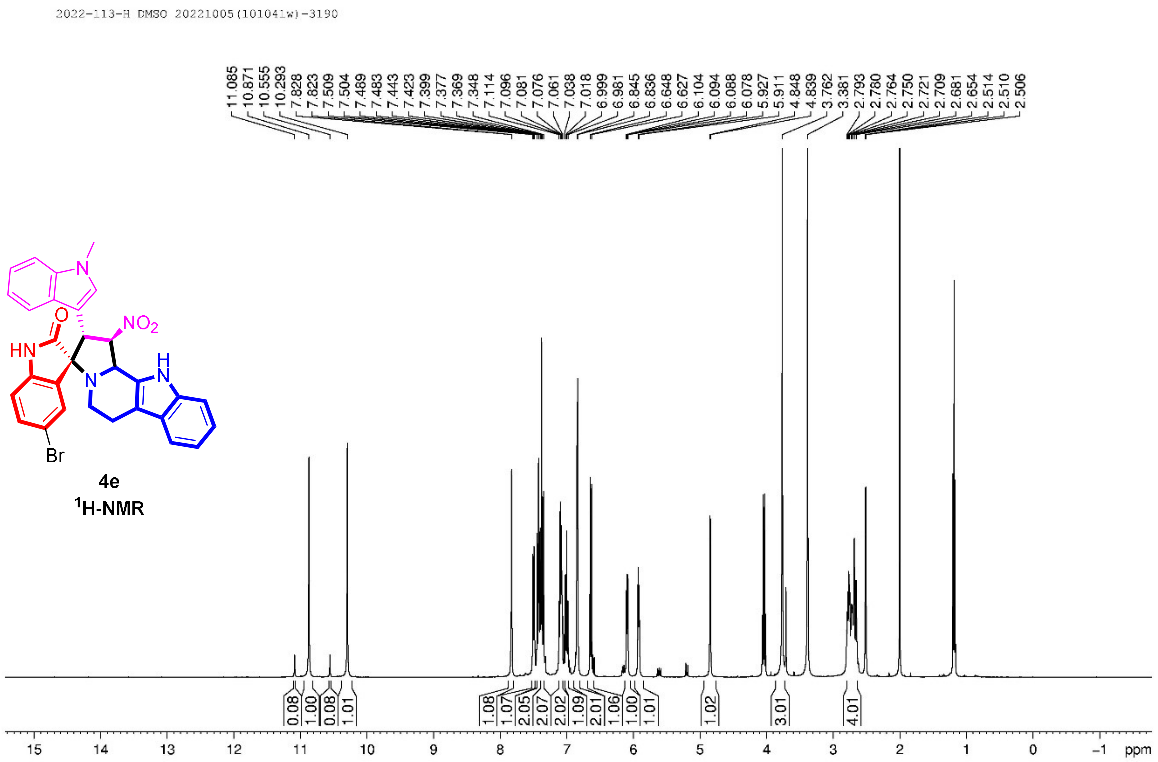
2022-113-H DMSO 20221005(101041w)-3190
11.085
10.871
10.555
10.293
7.828
7.823
7.509
7.504
7.489
7.483
7.443
7.423
7.399
7.377
7.369
7.348
7.114
7.096
7.081
7.076
7.061
7.038
7.018
6.999
6.981
6.845
6.836
6.648
6.627
6.104
6.094
6.088
6.078
5.927
5.911
4.848
4.839
3.762
3.381
2.793
2.780
2.764
2.750
2.721
2.709
2.681
2.654
2.514
2.510
2.506
NO2
HN
O
N
H
N
N
Br
4e
1H-NMR
0.08
1.00
0.08
1.01
1.08
1.07
2.05
2.07
2.02
1.09
2.01
1.06
1.00
1.01
1.02
3.01
4.01
ppm

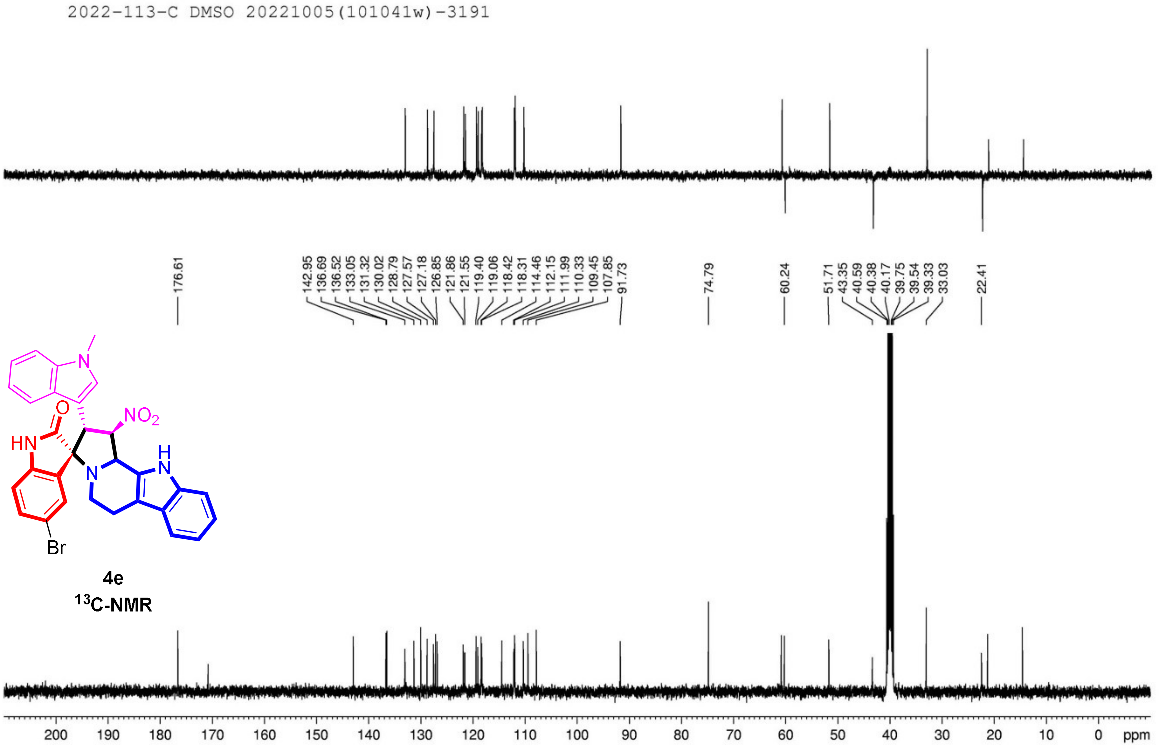
2022-113-C DMSO 20221005(101041w)-3191
176.61
142.95
136.69
136.52
133.05
131.32
130.02
128.79
127.57
127.18
126.85
121.86
121.55
119.40
119.06
118.42
118.31
114.46
112.15
111.99
110.33
109.45
107.85
91.73
74.79
60.24
51.71
43.35
40.59
40.38
40.17
39.75
39.54
39.33
33.03
22.41
NO2
HN
O
N
H
N
N
Br
4e
13C-NMR
ppm

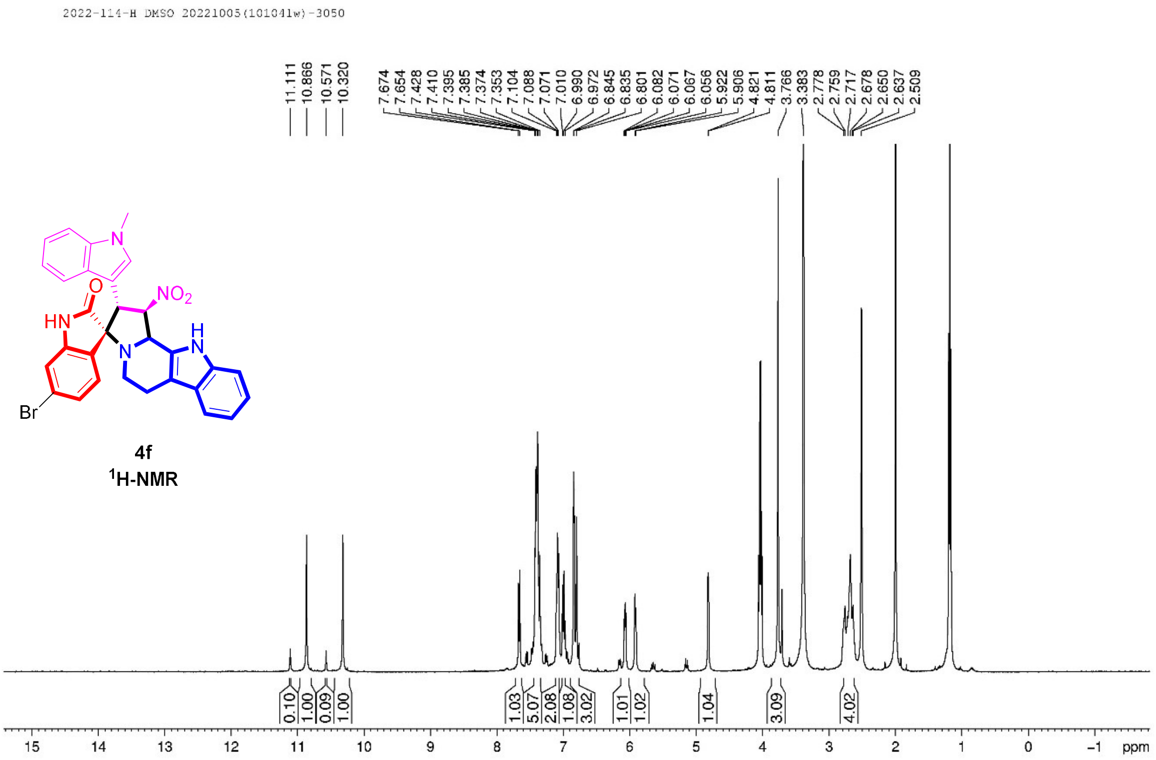

2022-114-H DMSO 20221005(101041w)-3050
11.111
10.866
10.571
10.320
7.674
7.654
7.428
7.410
7.395
7.385
7.374
7.353
7.104
7.088
7.071
7.010
6.990
6.972
6.845
6.835
6.801
6.082
6.071
6.067
6.056
5.922
5.906
4.821
4.811
3.766
3.383
2.778
2.759
2.717
2.678
2.650
2.637
2.509
NO2
HN
O
N
H
N
N
Br
4f
1H-NMR
0.10
1.00
0.09
1.00
1.03
5.07
2.08
1.08
3.02
1.01
1.02
1.04
3.09
4.02
15 14 13 12 11 10 9 8 7 6 5 4 3 2 1 0 -1 ppm

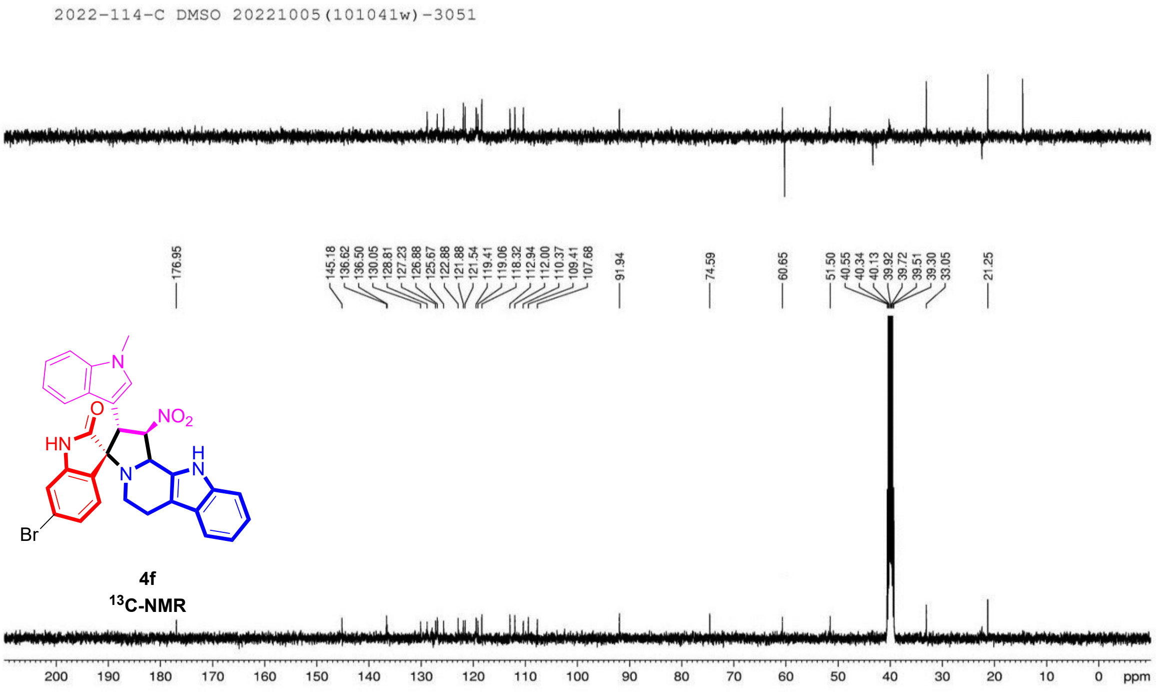

2022-114-C DMSO 20221005(101041w)-3051
176.95
145.18
136.62
136.50
130.05
128.81
127.23
126.88
125.67
122.88
121.88
121.54
119.41
119.06
118.32
112.94
112.00
110.37
109.41
107.68
91.94
74.59
60.65
51.50
40.55
40.34
40.13
39.92
39.72
39.51
39.30
33.05
21.25
NO2
HN
O
N
H
N
N
Br
4f
13C-NMR
200 190 180 170 160 150 140 130 120 110 100 90 80 70 60 50 40 30 20 10 0 ppm

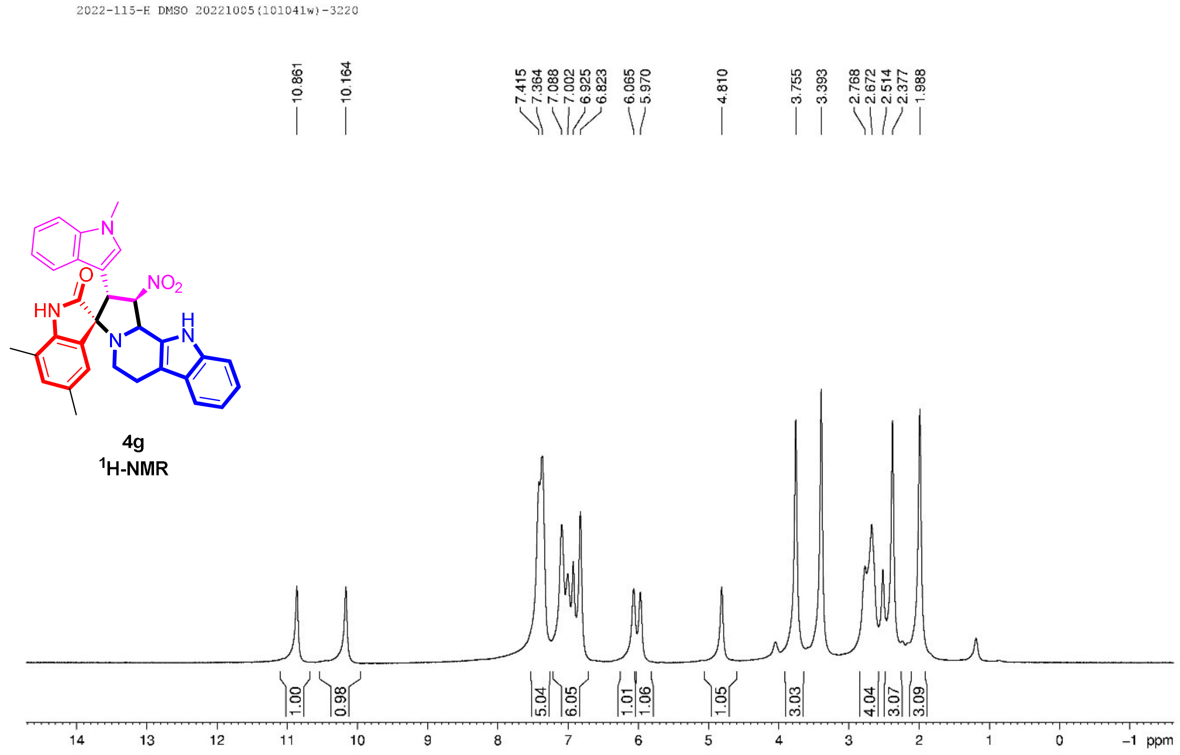

2022-115-H DMSO 20221005(101041w)-3220
10.861
10.164
7.415
7.364
7.088
7.002
6.925
6.823
6.065
5.970
4.810
3.755
3.393
2.768
2.672
2.514
2.377
1.988
NO2
HN
O
N
H
N
4g
1H-NMR
1.00
0.98
5.04
6.05
1.01
1.06
1.05
3.03
4.04
3.07
3.09
14 13 12 11 10 9 8 7 6 5 4 3 2 1 0 −1 ppm

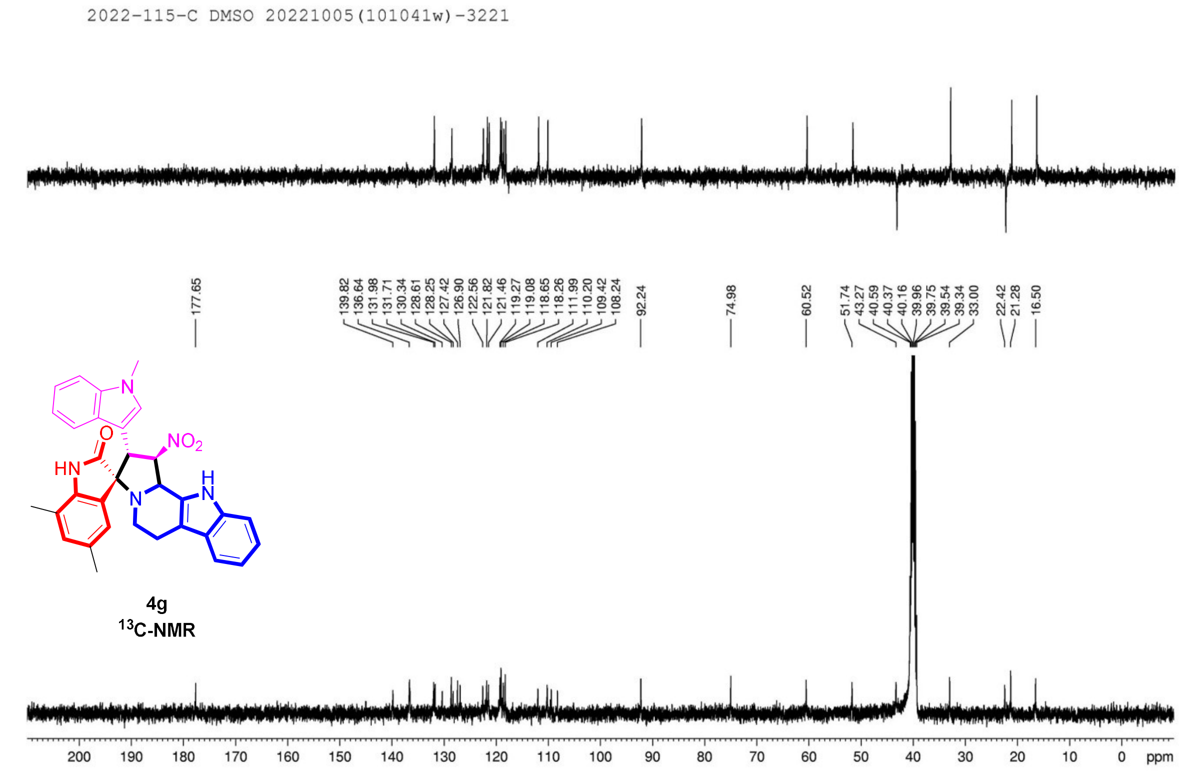

2022-115-C DMSO 20221005(101041w)-3221
177.65
139.82
136.64
131.98
131.71
130.34
128.61
128.25
127.42
126.90
122.56
121.82
121.46
119.27
119.08
118.65
118.26
111.99
110.20
109.42
108.24
92.24
74.98
60.52
51.74
43.27
40.59
40.37
40.16
39.96
39.75
39.54
39.34
33.00
22.42
21.28
16.50
NO2
HN
O
N
H
N
4g
13C-NMR
200 190 180 170 160 150 140 130 120 110 100 90 80 70 60 50 40 30 20 10 0 ppm

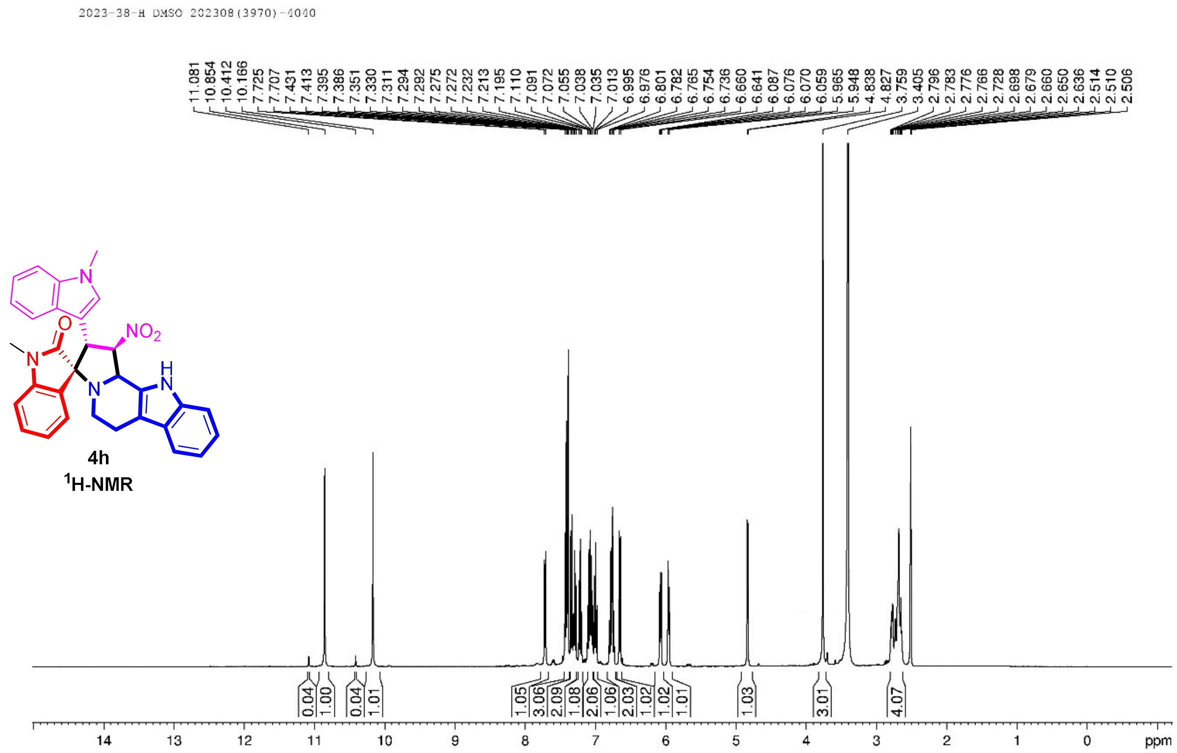

4h

^{1}H-NMR

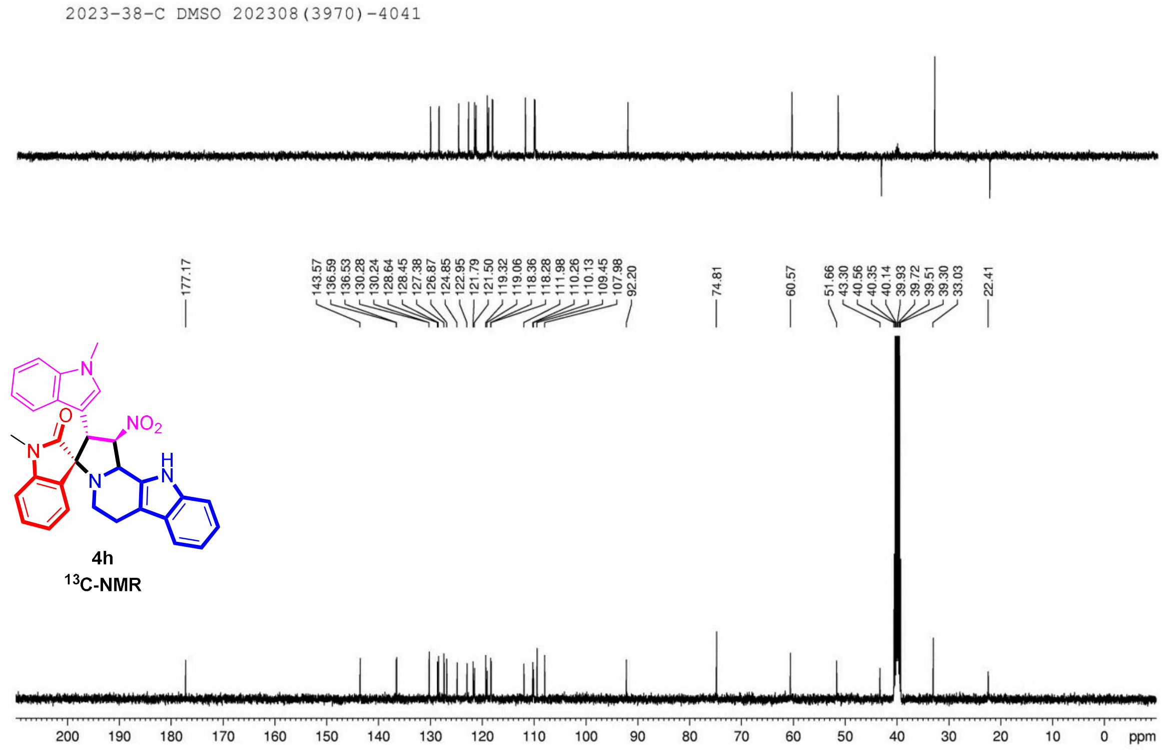

4h

^{13}C-NMR

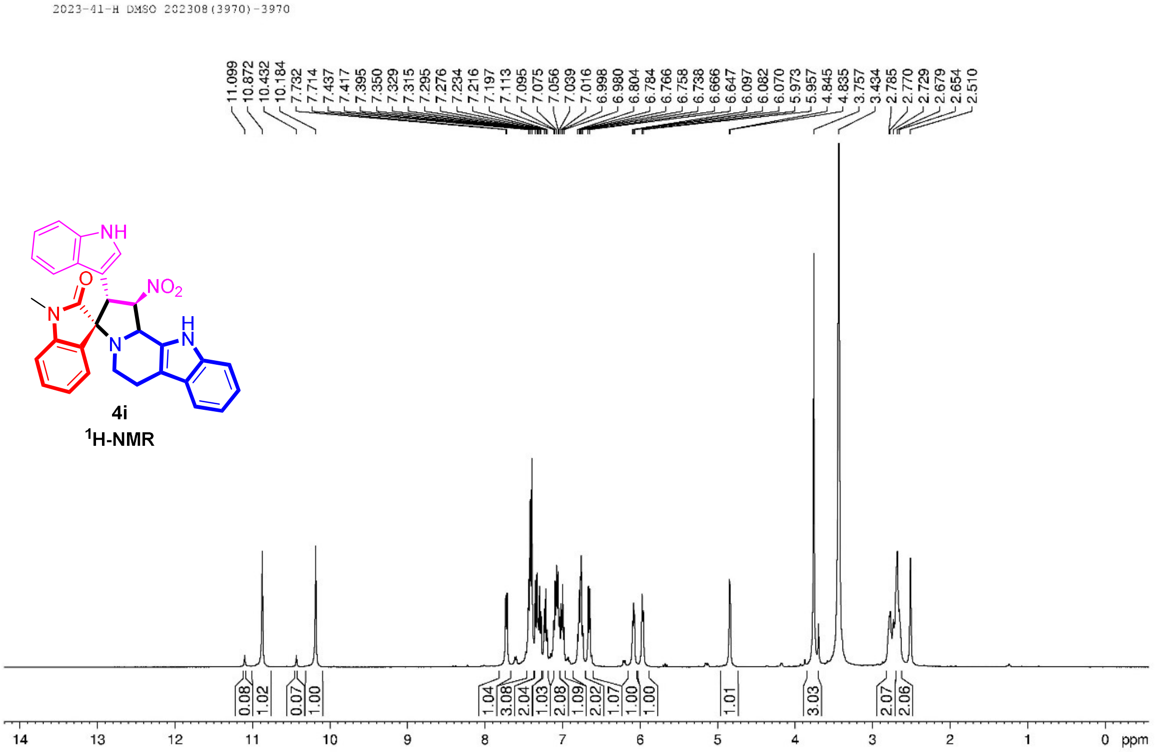
2023-41-H DMSO 202308(3970)-3970
11.099 10.872 10.432 10.184 7.732 7.714 7.437 7.417 7.395 7.350 7.329 7.315 7.295 7.276 7.234 7.216 7.197 7.113 7.095 7.075 7.056 7.039 7.016 6.998 6.980 6.804 6.784 6.766 6.758 6.738 6.666 6.647 6.097 6.082 6.070 5.973 5.957 4.845 4.835 3.757 3.434 2.785 2.770 2.729 2.679 2.654 2.510
NH
O
NO_2
N
H
4i
^{1}H-NMR
0.08 1.02 0.07 1.00 1.04 3.08 2.04 1.03 2.08 1.09 2.02 1.07 1.00 1.00 1.01 3.03 2.07 2.06
14 13 12 11 10 9 8 7 6 5 4 3 2 1 0 ppm

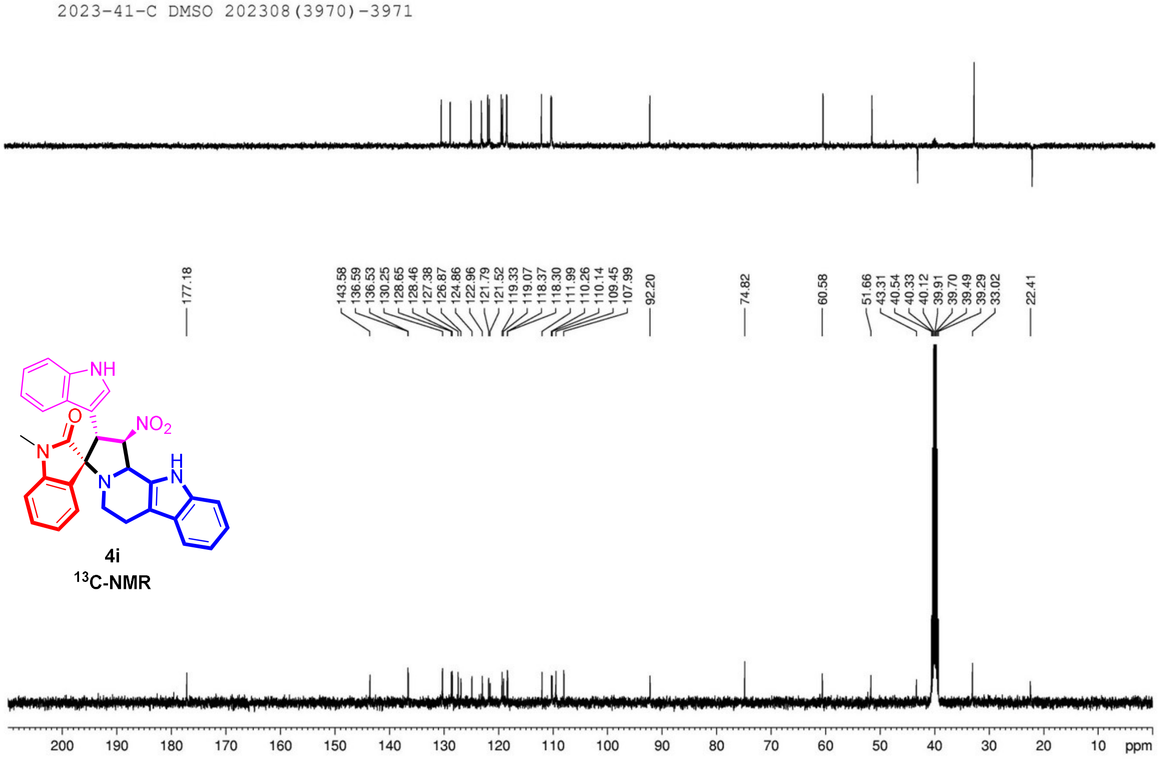
2023-41-C DMSO 202308(3970)-3971
177.18
143.58 136.59 136.53 130.25 128.65 128.46 127.38 126.87 124.86 122.96 121.79 121.52 119.33 119.07 118.37 118.30 111.99 110.26 110.14 109.45 107.99 92.20
74.82
60.58
51.66 43.31 40.54 40.33 40.12 39.91 39.70 39.49 39.29 33.02
22.41
NH
O
NO_2
N
H
4i
^{13}C-NMR
200 190 180 170 160 150 140 130 120 110 100 90 80 70 60 50 40 30 20 10 ppm

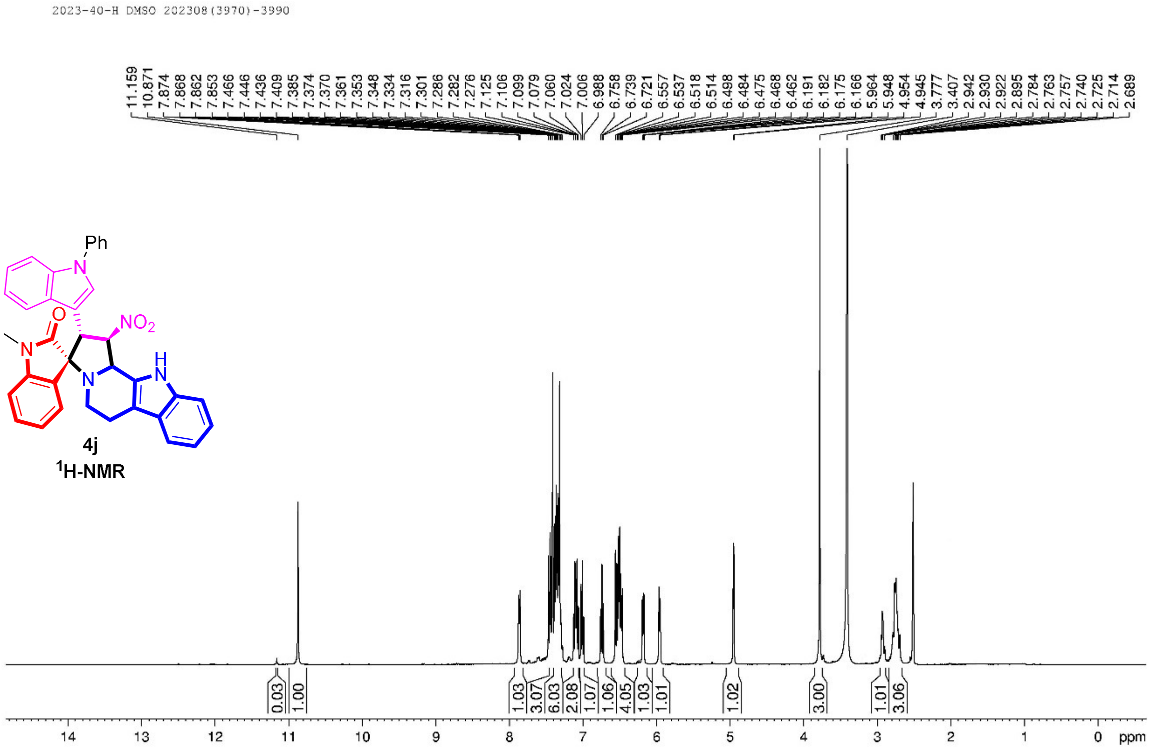
2023-40-H DMSO 202308(3970)-3990
4j
1H-NMR
ppm

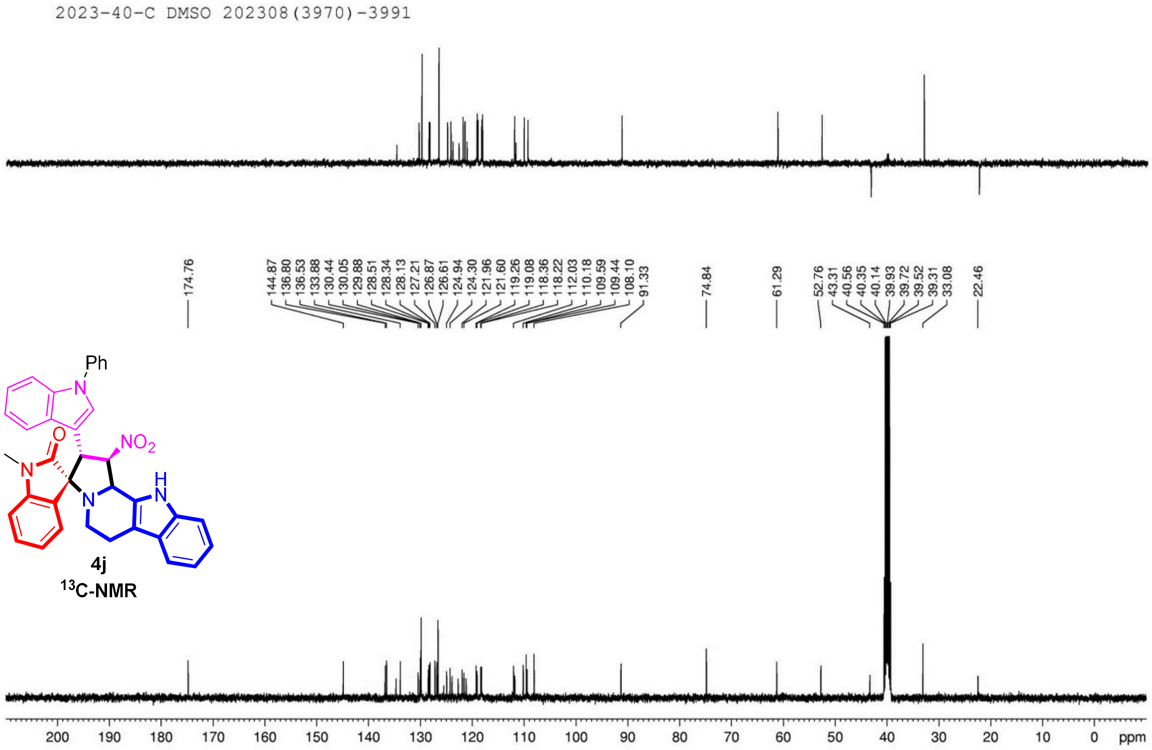
2023-40-C DMSO 202308(3970)-3991
4j
13C-NMR
ppm

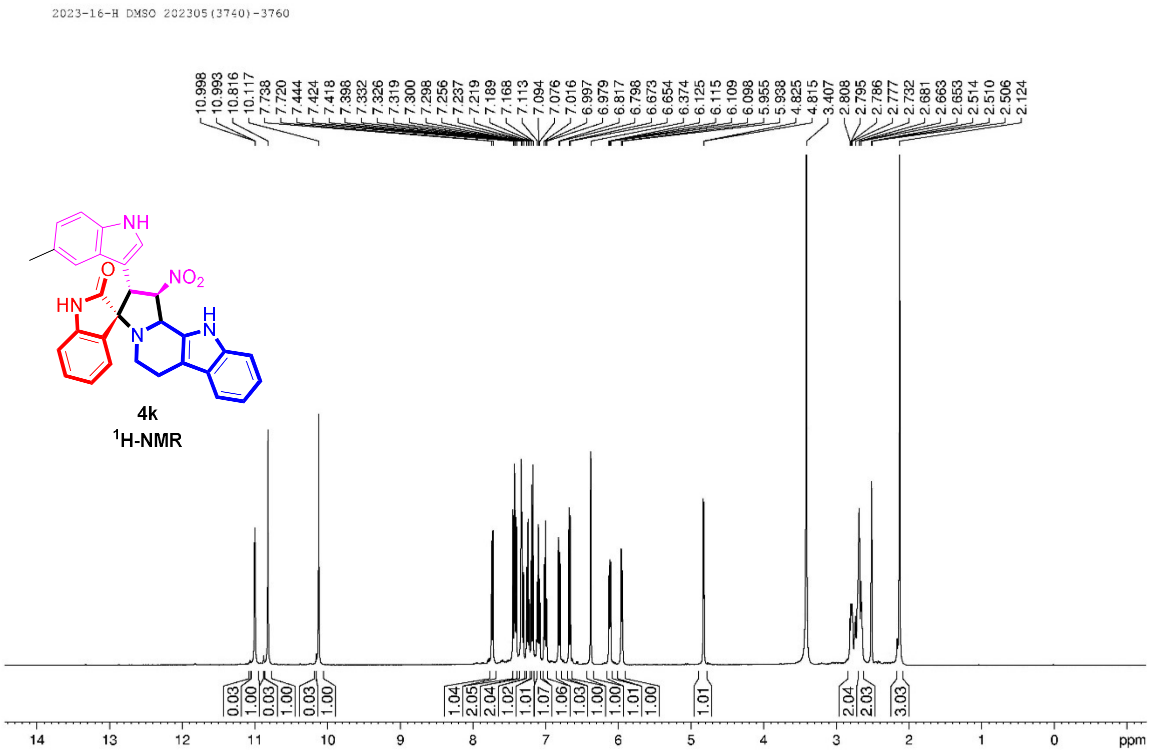
2023-16-H DMSO 202305(3740)-3760
10.998 10.993 10.816 10.117 7.738 7.720 7.444 7.424 7.418 7.398 7.332 7.326 7.319 7.300 7.298 7.256 7.237 7.219 7.189 7.168 7.113 7.094 7.076 7.016 6.997 6.979 6.817 6.798 6.673 6.654 6.374 6.125 6.115 6.109 6.098 5.955 5.938 4.825 4.815 3.407 2.808 2.795 2.786 2.777 2.732 2.681 2.663 2.653 2.514 2.510 2.506 2.124
NH
NO2
HN
O
N
H
N
4k
1H-NMR
0.03 1.00 0.03 1.00 0.03 1.00 1.04 2.05 2.04 1.02 1.01 1.07 1.06 1.03 1.00 1.00 1.01 1.00 1.01 2.04 2.03 3.03
14 13 12 11 10 9 8 7 6 5 4 3 2 1 0 ppm

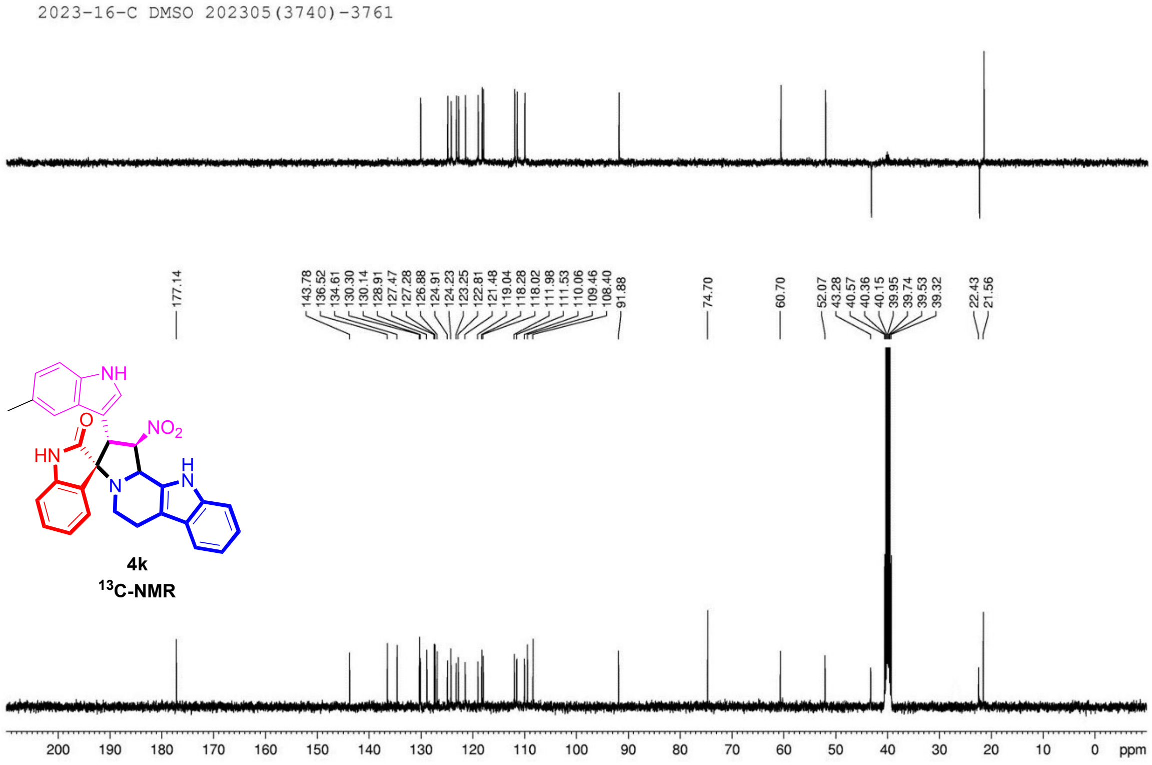
2023-16-C DMSO 202305(3740)-3761
177.14
143.78 136.52 134.61 130.30 130.14 128.91 127.47 127.28 126.88 124.91 124.23 123.25 122.81 121.48 119.04 118.28 118.02 111.98 111.53 110.06 109.46 108.40 91.88
74.70
60.70
52.07 43.28 40.57 40.36 40.15 39.95 39.74 39.53 39.32
22.43 21.56
NH
NO2
HN
O
N
H
N
4k
13C-NMR
200 190 180 170 160 150 140 130 120 110 100 90 80 70 60 50 40 30 20 10 0 ppm

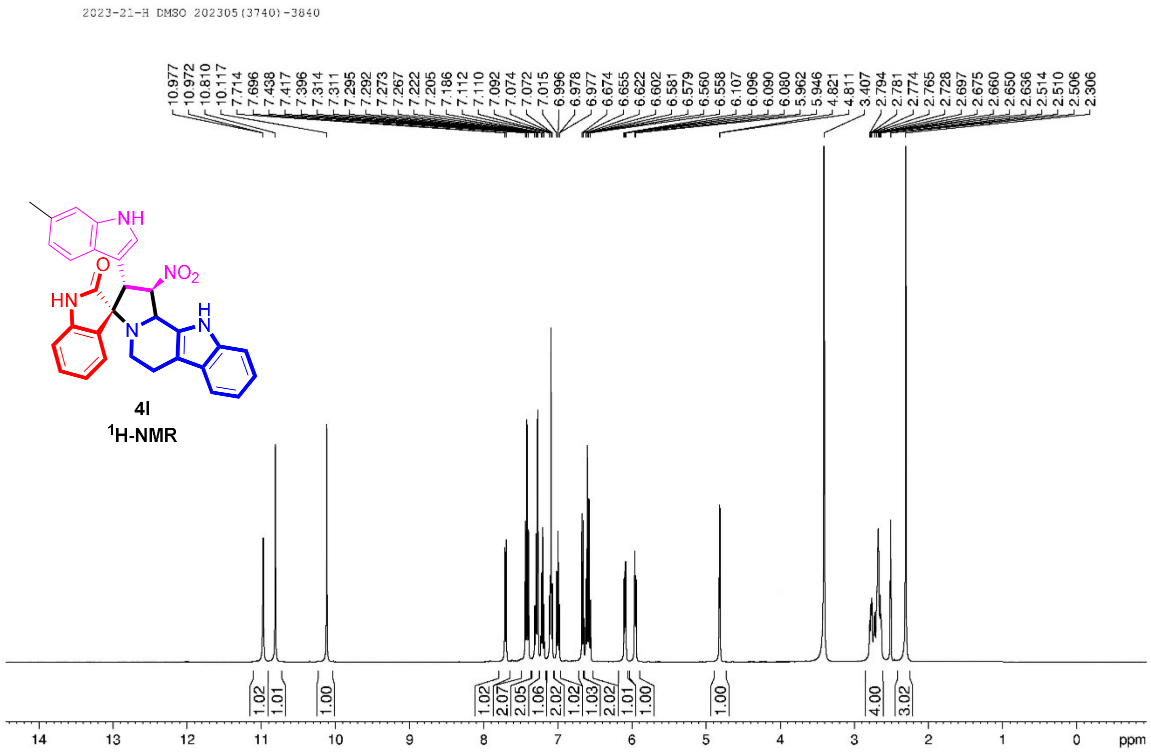

2023-21-H DMSO 202305(3740)-3840
10.977
10.972
10.810
10.117
7.714
7.696
7.438
7.417
7.396
7.314
7.311
7.295
7.292
7.273
7.267
7.222
7.205
7.186
7.112
7.110
7.092
7.074
7.072
7.015
6.996
6.978
6.977
6.674
6.655
6.622
6.602
6.581
6.579
6.560
6.558
6.107
6.096
6.090
6.080
5.962
5.946
4.821
4.811
3.407
2.794
2.781
2.774
2.765
2.728
2.697
2.675
2.660
2.650
2.636
2.514
2.510
2.506
2.306
NH
NO2
O
HN
N
H
N
4l
1H-NMR
1.02
1.01
1.00
1.02
2.07
2.05
1.06
2.02
1.02
1.03
2.02
1.01
1.00
1.00
4.00
3.02
14
13
12
11
10
9
8
7
6
5
4
3
2
1
0
ppm

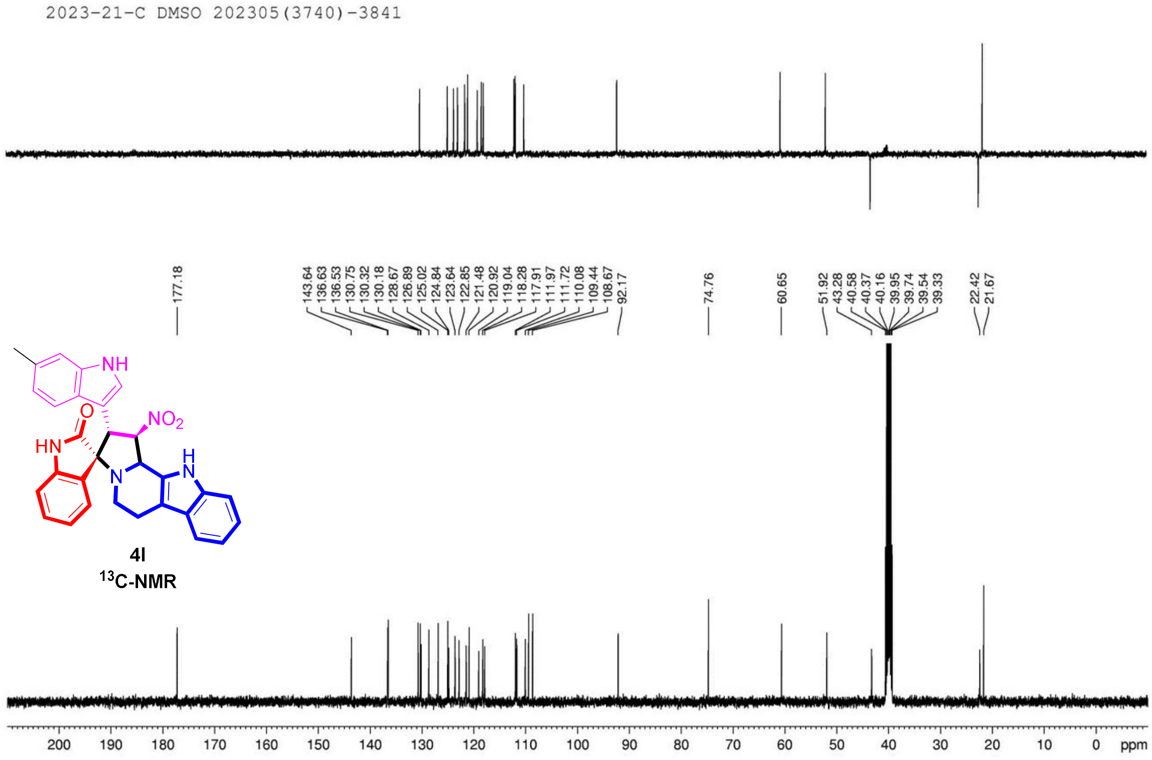

2023-21-C DMSO 202305(3740)-3841
177.18
143.64
136.63
136.53
130.75
130.32
130.18
128.67
126.89
125.02
124.84
123.64
122.85
121.48
120.92
119.04
118.28
117.91
111.97
111.72
110.08
109.44
108.67
92.17
74.76
60.65
51.92
43.28
40.58
40.37
40.16
39.95
39.74
39.54
39.33
22.42
21.67
NH
NO2
O
HN
N
H
N
4l
13C-NMR
200
190
180
170
160
150
140
130
120
110
100
90
80
70
60
50
40
30
20
10
0
ppm

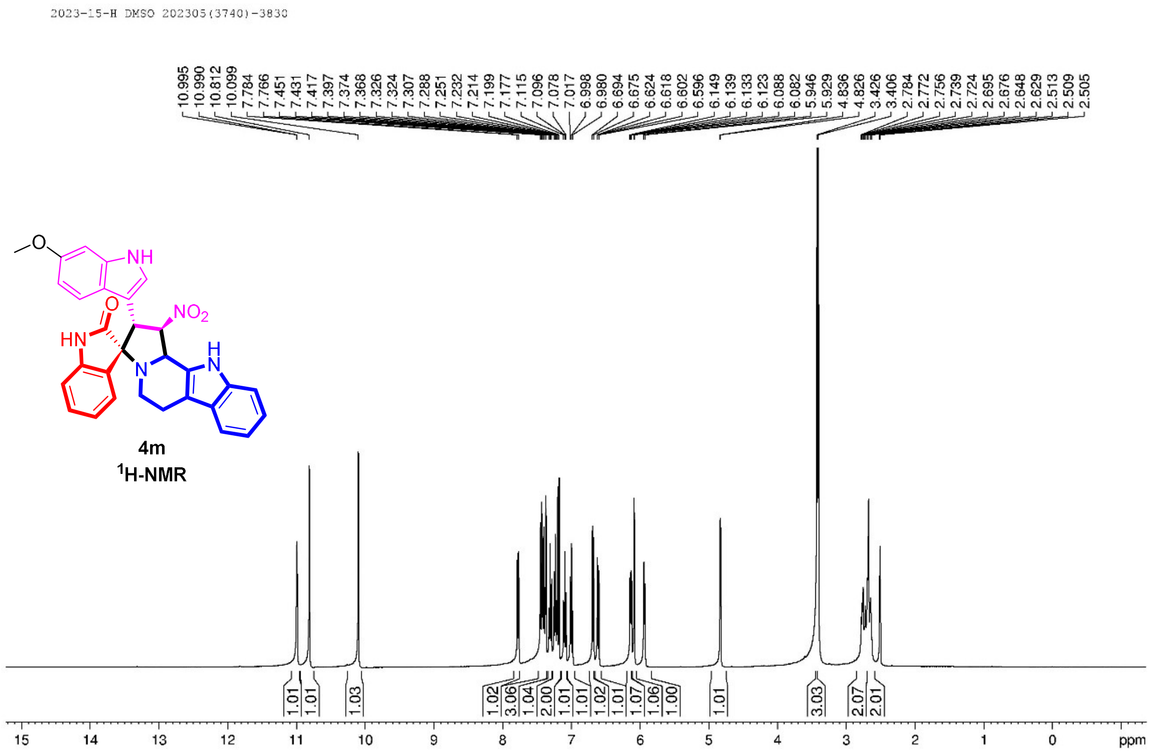
2023-15-H DMSO 202305(3740)-3830
4m
1H-NMR
ppm

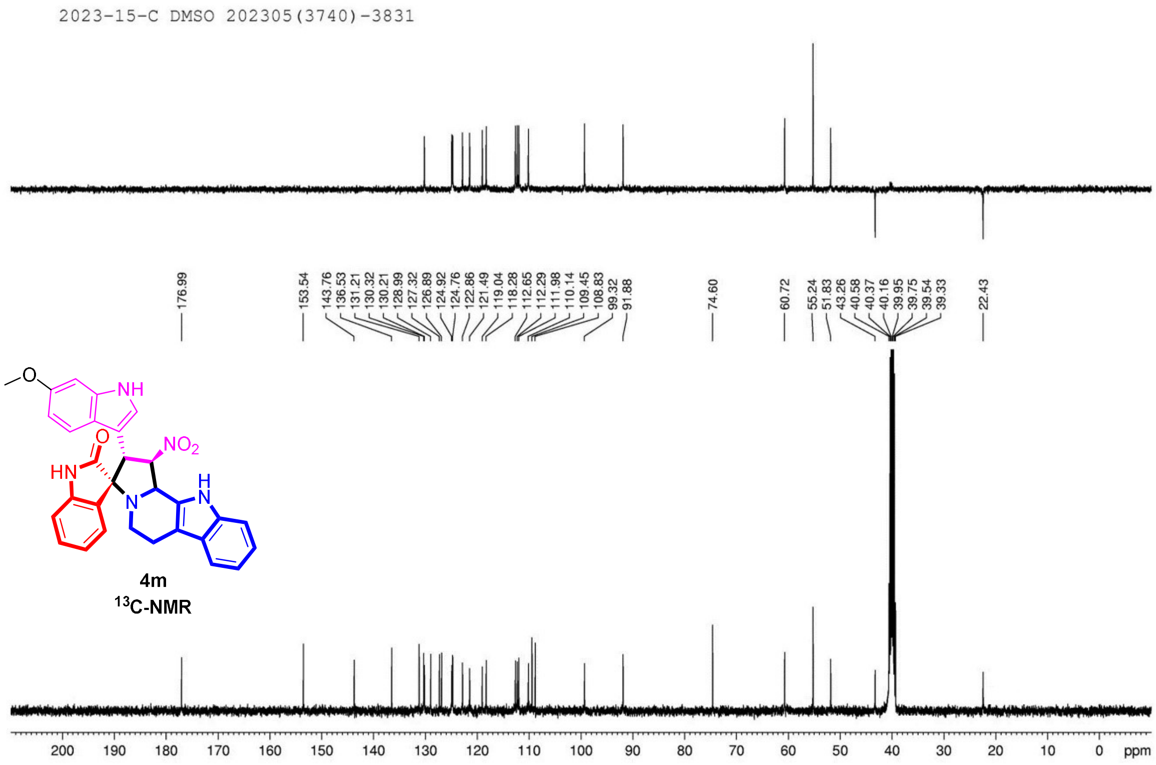
2023-15-C DMSO 202305(3740)-3831
4m
13C-NMR
ppm

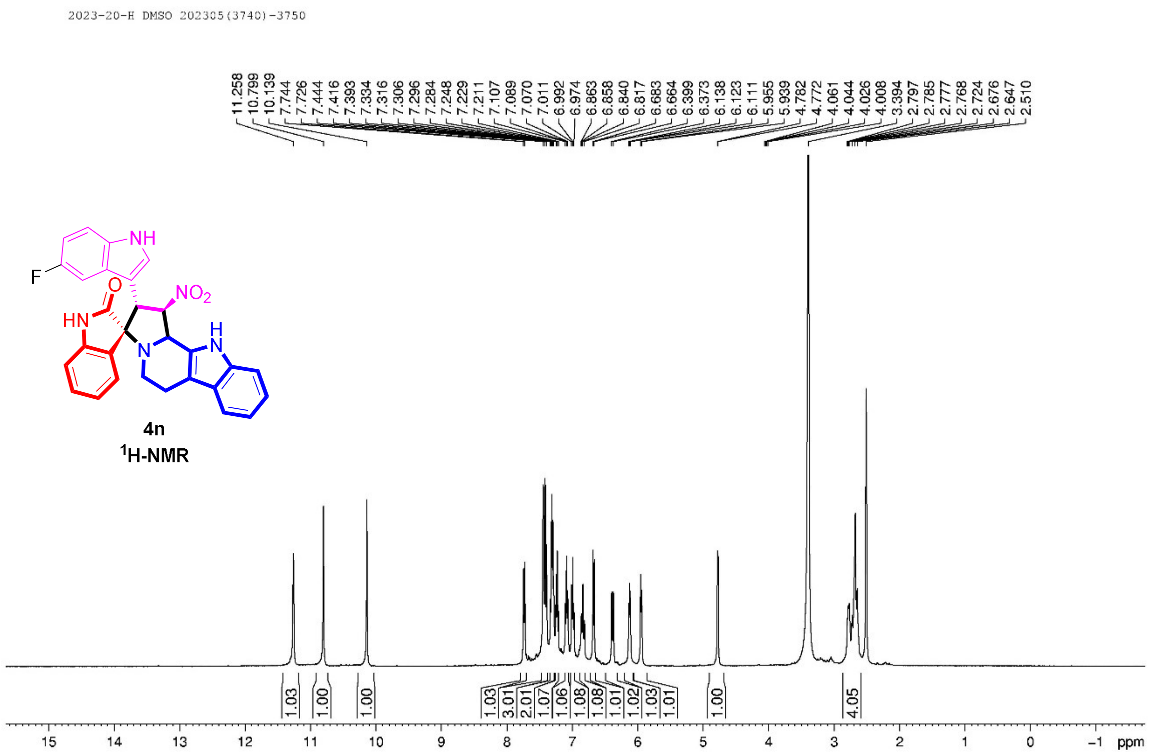

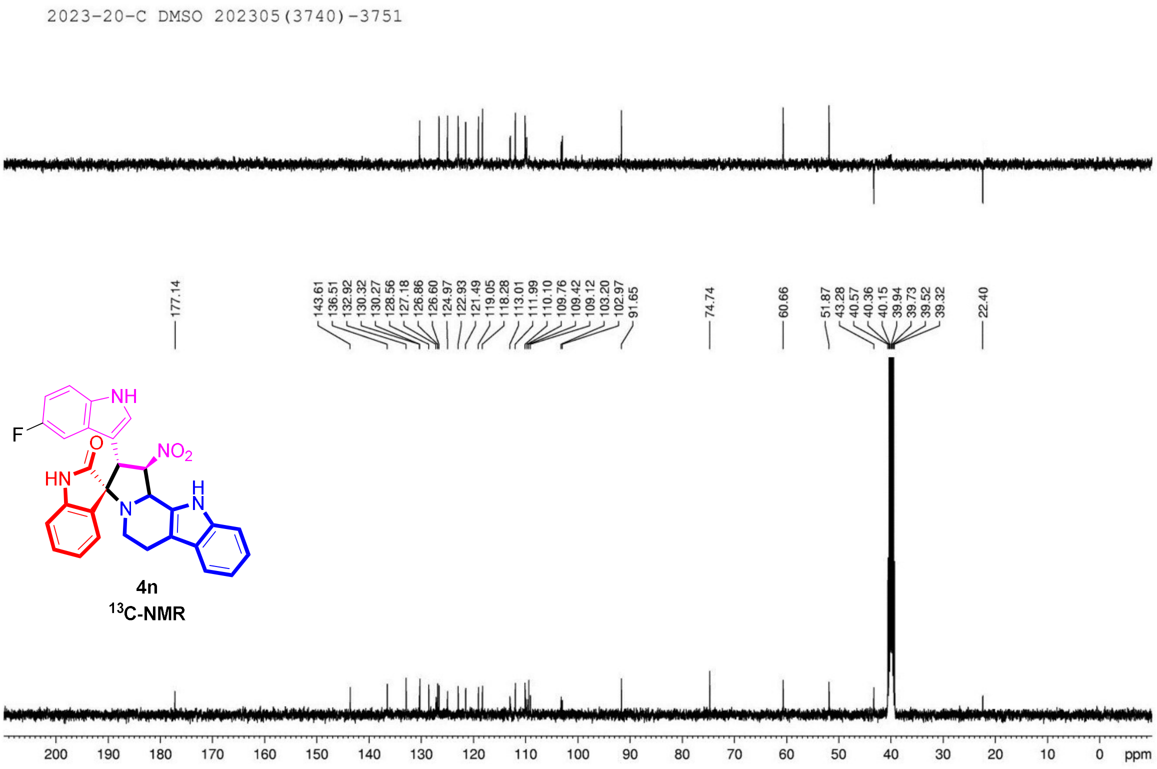

2022-102-H CDCl3 20221005(101041w)-3370

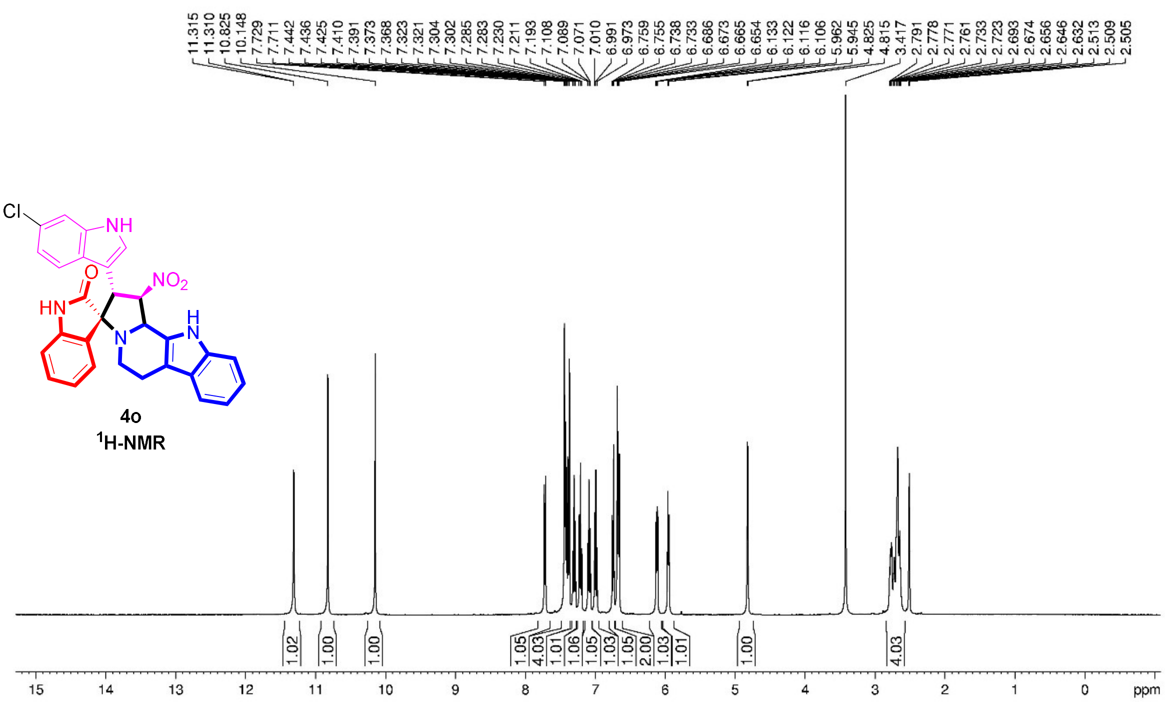

2022-102-C CDCl3 20221005(101041w)-3371

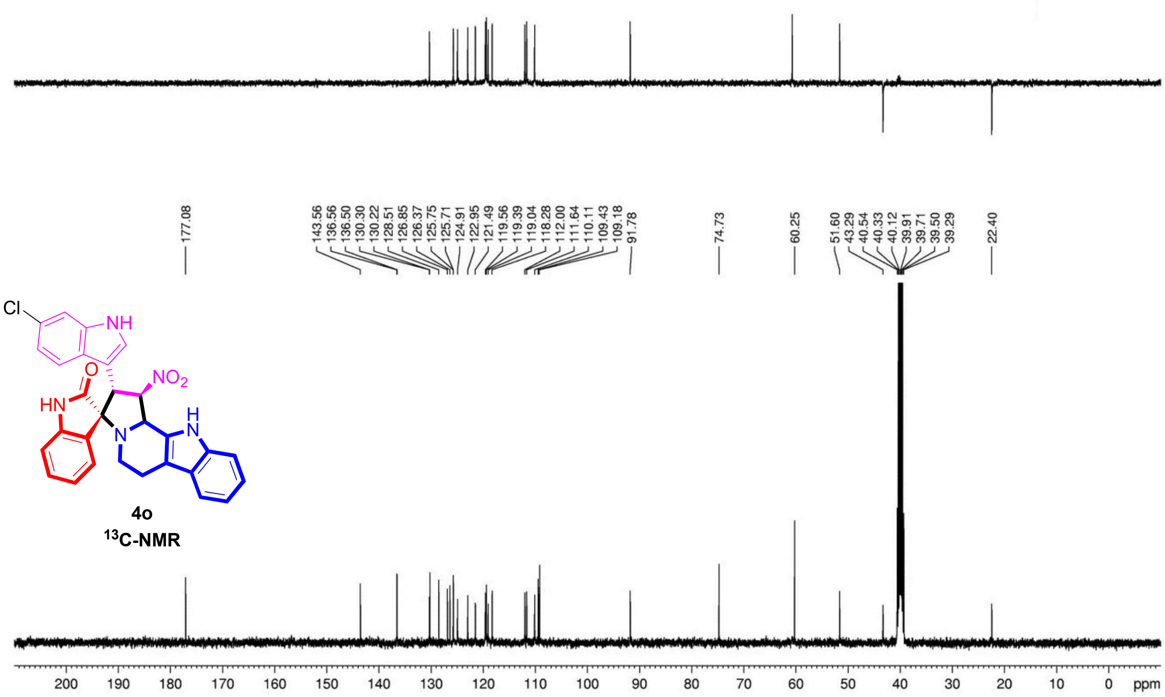

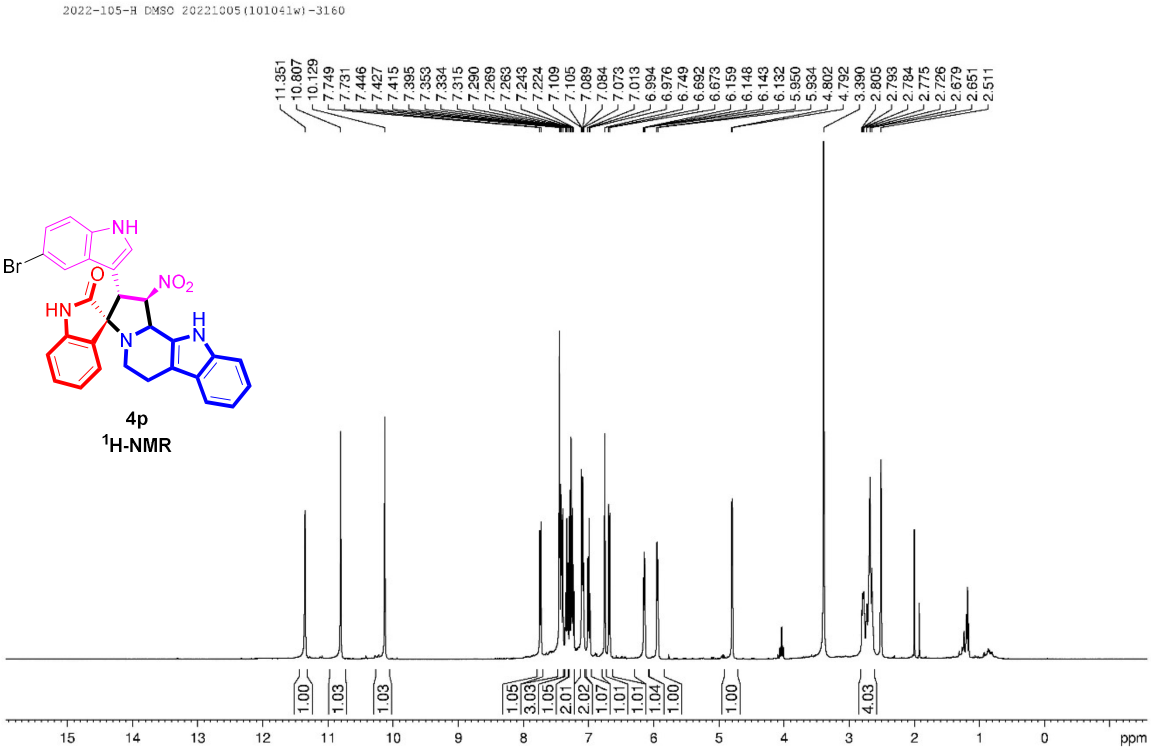
2022-105-H DMSO 20221005(101041w)-3160
11.351
10.807
10.129
7.749
7.731
7.446
7.427
7.415
7.395
7.353
7.334
7.315
7.290
7.269
7.263
7.243
7.224
7.109
7.105
7.089
7.084
7.073
7.013
6.994
6.976
6.749
6.692
6.673
6.159
6.148
6.143
6.132
5.950
5.934
4.802
4.792
3.390
2.805
2.793
2.784
2.775
2.726
2.679
2.651
2.511
Br
NH
O
NO2
HN
N
H
N
4p
1H-NMR
1.00
1.03
1.03
1.05
3.03
1.05
2.01
2.02
1.07
1.01
1.01
1.04
1.00
1.00
4.03
ppm

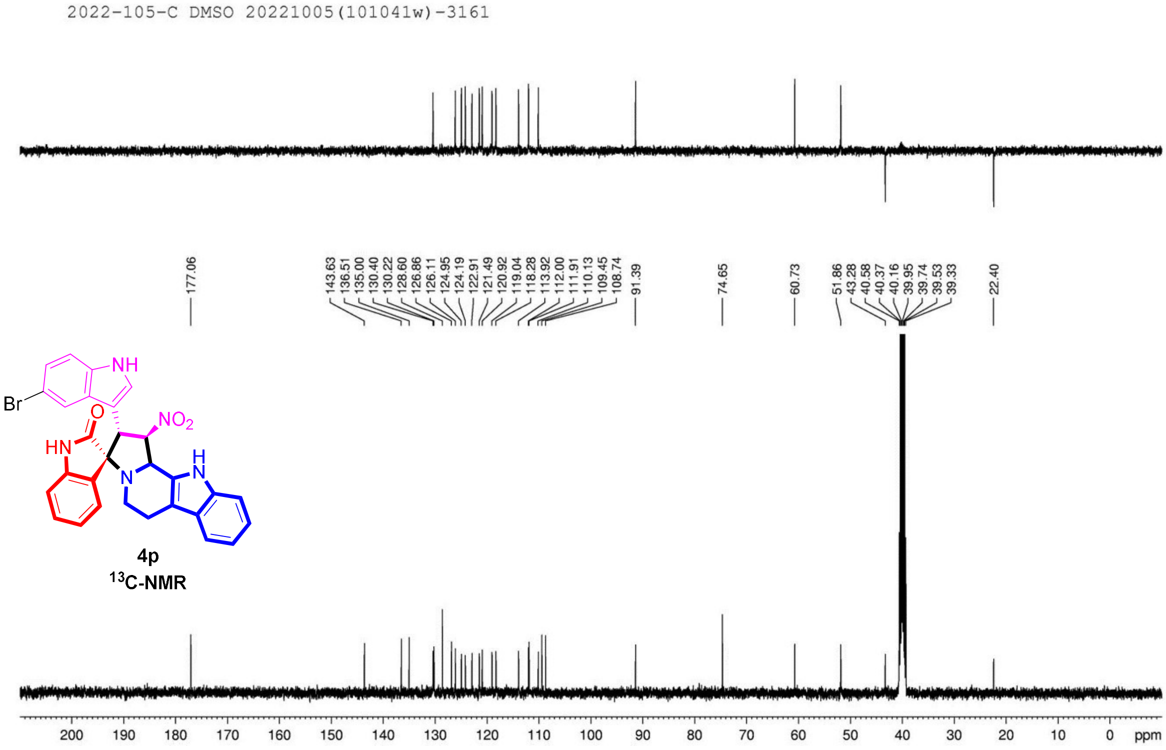
2022-105-C DMSO 20221005(101041w)-3161
177.06
143.63
136.51
135.00
130.40
130.22
128.60
126.86
126.11
124.95
124.19
122.91
121.49
120.92
119.04
118.28
113.92
112.00
111.91
110.13
109.45
108.74
91.39
74.65
60.73
51.86
43.28
40.58
40.37
40.16
39.95
39.74
39.53
39.33
22.40
Br
NH
O
NO2
HN
N
H
N
4p
13C-NMR
ppm

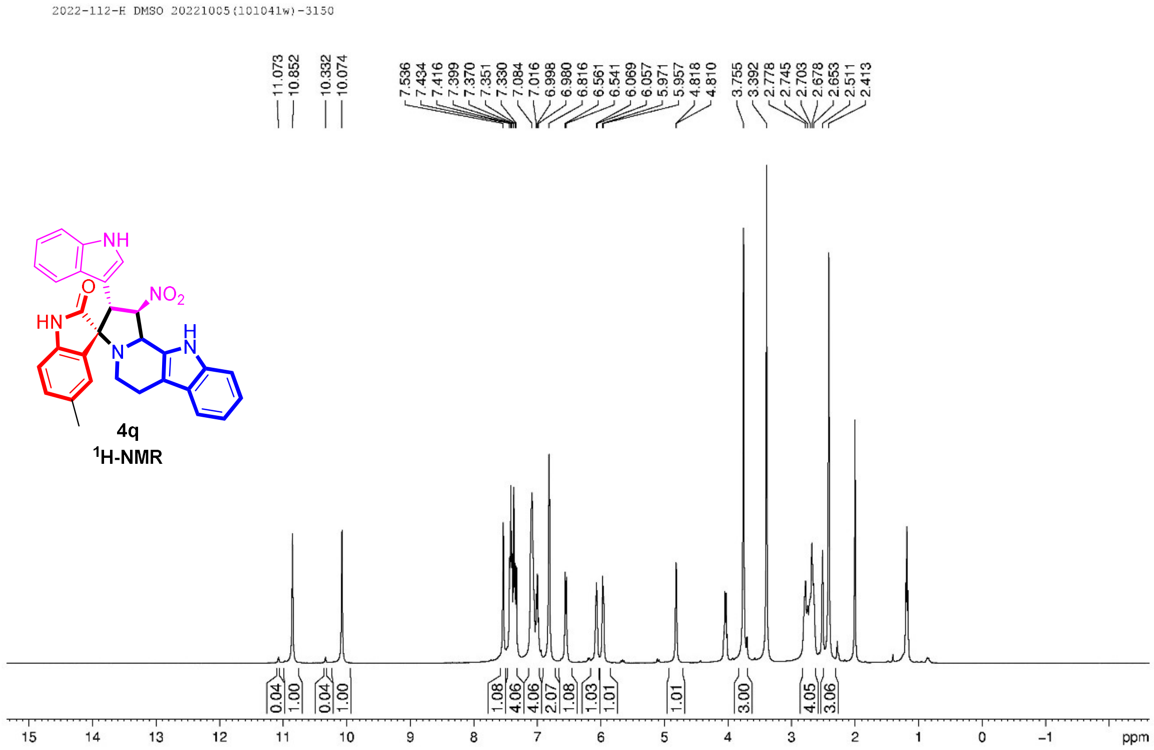
2022-112-E DMSO 20221005(101041w)-3150
11.073
10.852
10.332
10.074
7.536
7.434
7.416
7.399
7.370
7.351
7.330
7.084
7.016
6.998
6.980
6.816
6.561
6.541
6.069
6.057
5.971
5.957
4.818
4.810
3.755
3.392
2.778
2.745
2.703
2.678
2.653
2.511
2.413
NH
NO_2
O
HN
N
H
N
4q
^{1}H-NMR
0.04
1.00
0.04
1.00
1.08
4.06
4.06
2.07
1.08
1.03
1.01
1.01
3.00
4.05
3.06
15 14 13 12 11 10 9 8 7 6 5 4 3 2 1 0 −1 ppm

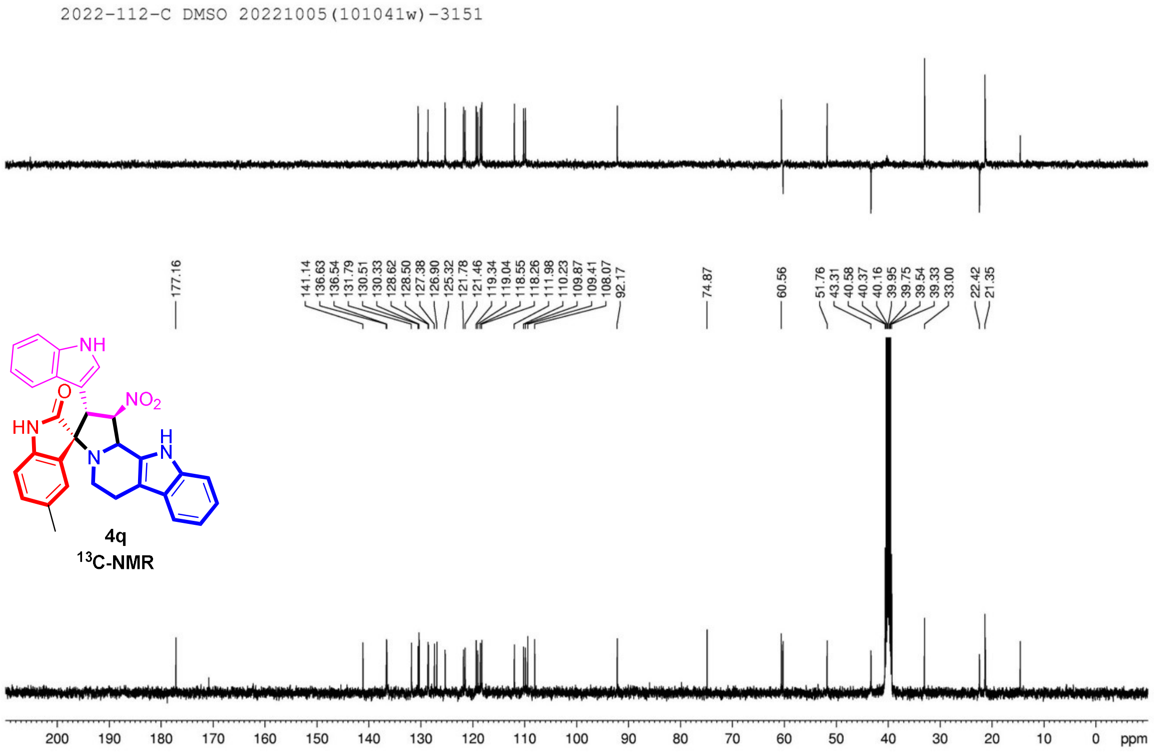
2022-112-C DMSO 20221005(101041w)-3151
177.16
141.14
136.63
136.54
131.79
130.51
130.33
128.62
128.50
127.38
126.90
125.32
121.78
121.46
119.34
119.04
118.55
118.26
111.98
110.23
109.87
109.41
108.07
92.17
74.87
60.56
51.76
43.31
40.58
40.37
40.16
39.95
39.75
39.54
39.33
33.00
22.42
21.35
NH
NO_2
O
HN
N
H
N
4q
^{13}C-NMR
200 190 180 170 160 150 140 130 120 110 100 90 80 70 60 50 40 30 20 10 0 ppm

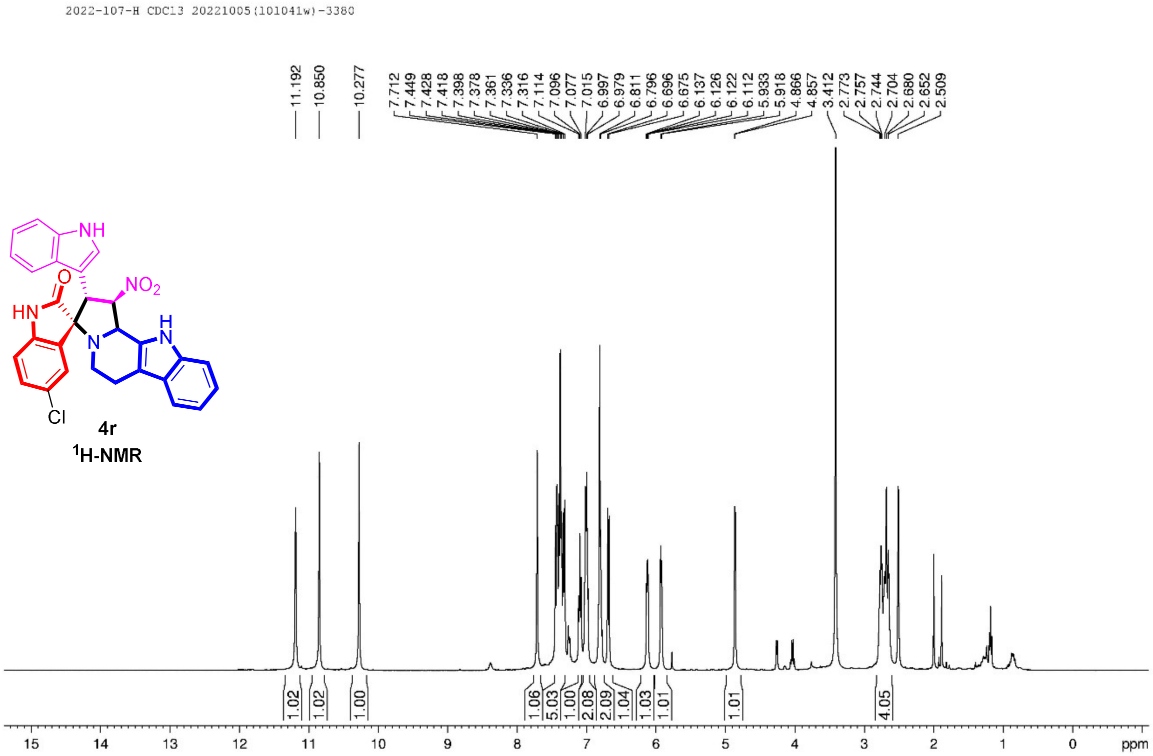
2022-107-H CDCl3 20221005(101041w)-3380
11.192
10.850
10.277
7.712
7.449
7.428
7.418
7.398
7.378
7.361
7.336
7.316
7.114
7.096
7.077
7.015
6.997
6.979
6.811
6.796
6.696
6.675
6.137
6.126
6.122
6.112
5.933
5.918
4.866
4.857
3.412
2.773
2.757
2.744
2.704
2.680
2.652
2.509
NH
O
NO_2
HN
N
H
N
Cl
4r
^{1}H-NMR
1.02
1.02
1.00
1.06
5.03
1.00
2.08
2.09
1.04
1.03
1.01
1.01
4.05
ppm

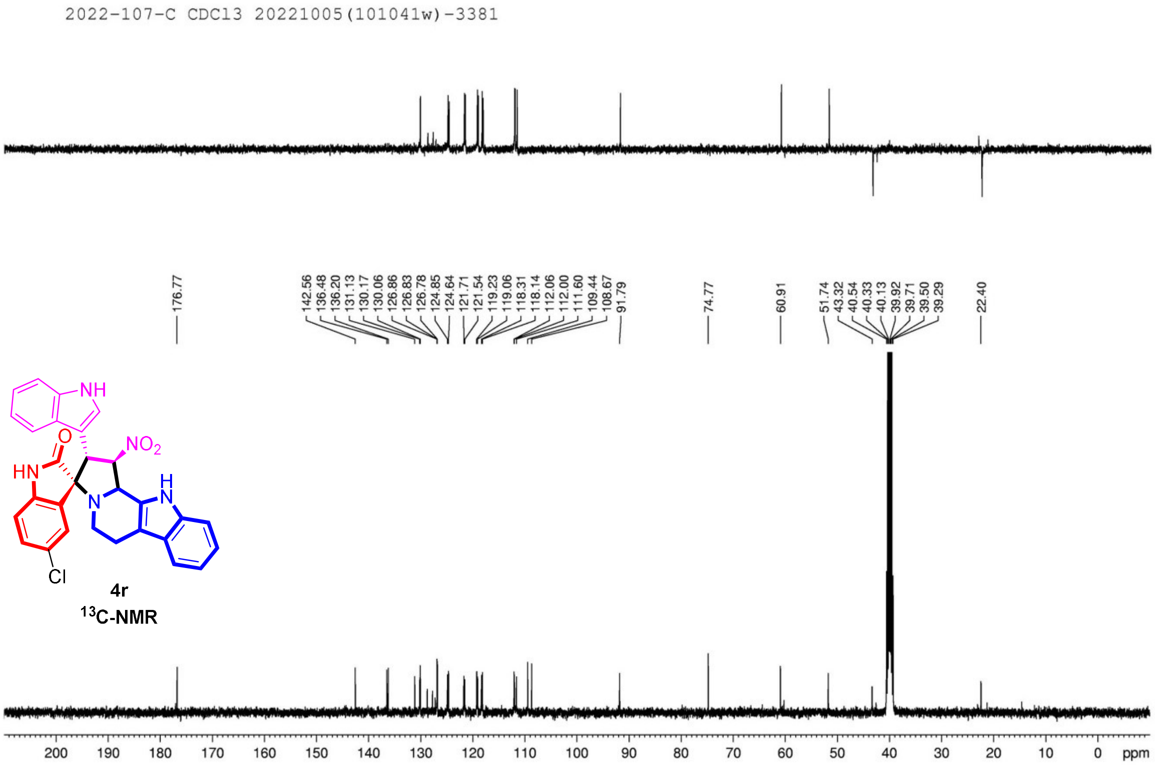
2022-107-C CDCl3 20221005(101041w)-3381
176.77
142.56
136.48
136.20
131.13
130.17
130.06
126.86
126.83
126.78
124.85
124.64
121.71
121.54
119.23
119.06
118.31
118.14
112.06
112.00
111.60
109.44
108.67
91.79
74.77
60.91
51.74
43.32
40.54
40.33
40.13
39.92
39.71
39.50
39.29
22.40
NH
O
NO_2
HN
N
H
N
Cl
4r
^{13}C-NMR
ppm

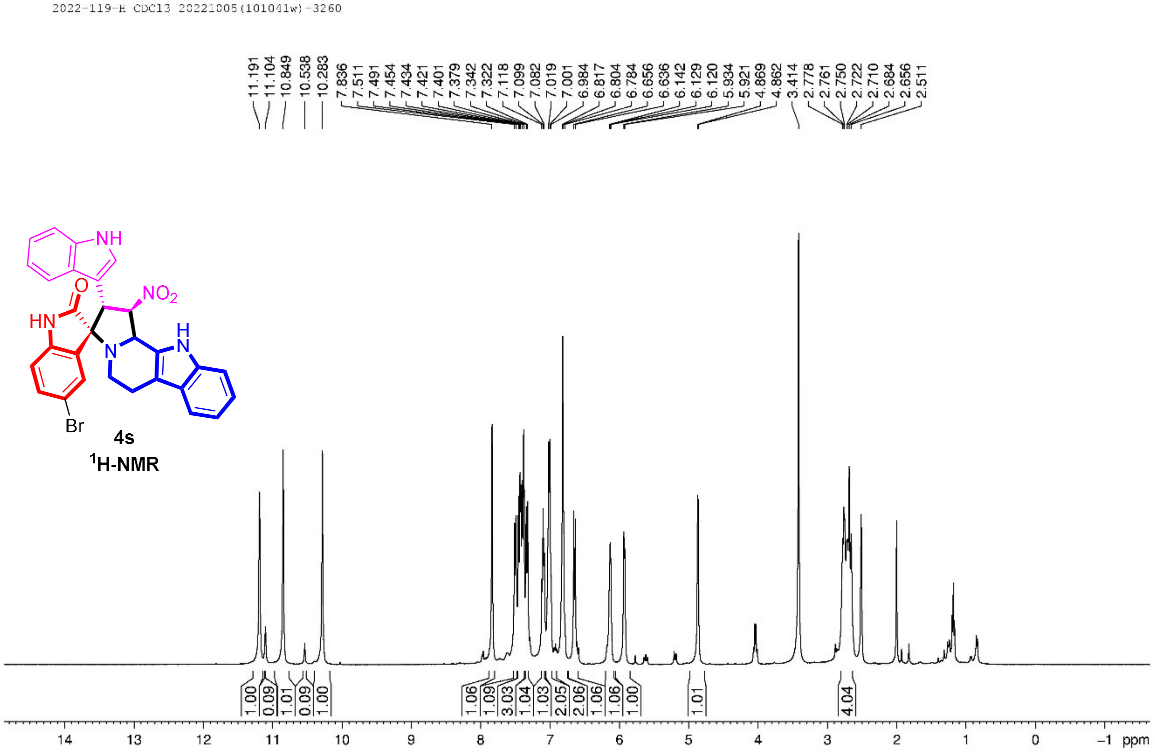
2022-119-H CDCl3 20221005(101041w)-3260
11.191 11.104 10.849 10.538 10.283 7.836 7.511 7.491 7.454 7.434 7.421 7.401 7.379 7.342 7.322 7.118 7.099 7.082 7.019 7.001 6.984 6.817 6.804 6.784 6.656 6.636 6.142 6.129 6.120 5.934 5.921 4.869 4.862 3.414 2.778 2.761 2.750 2.722 2.710 2.684 2.656 2.511
NH
NO2
HN
O
H
N
N
Br
4s
1H-NMR
1.00 0.09 1.01 0.09 1.00 1.06 1.09 3.03 1.04 1.03 2.05 2.06 1.06 1.00 1.01 4.04
14 13 12 11 10 9 8 7 6 5 4 3 2 1 0 –1 ppm

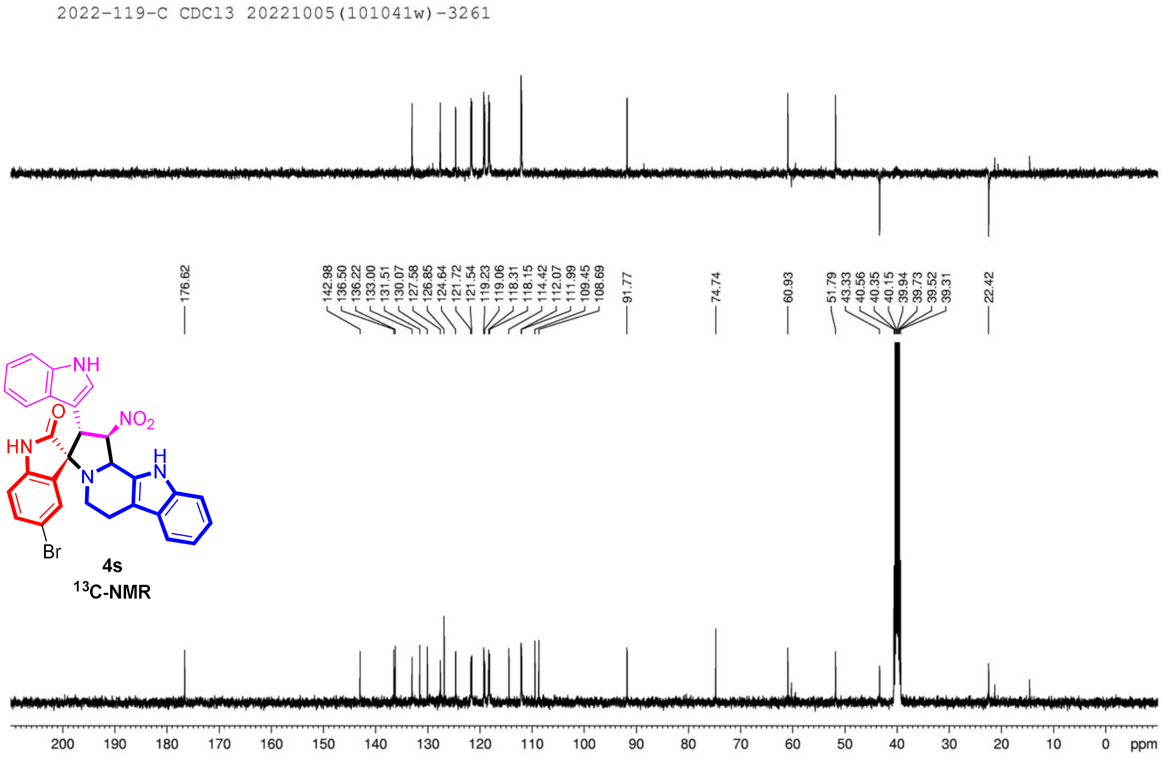
2022-119-C CDCl3 20221005(101041w)-3261
176.62
142.98 136.50 136.22 133.00 131.51 130.07 127.58 126.85 124.64 121.72 121.54 119.23 119.06 118.31 118.15 114.42 112.07 111.99 109.45 108.69
91.77
74.74
60.93
51.79 43.33 40.56 40.35 40.15 39.94 39.73 39.52 39.31
22.42
NH
NO2
HN
O
H
N
N
Br
4s
13C-NMR
200 190 180 170 160 150 140 130 120 110 100 90 80 70 60 50 40 30 20 10 0 ppm

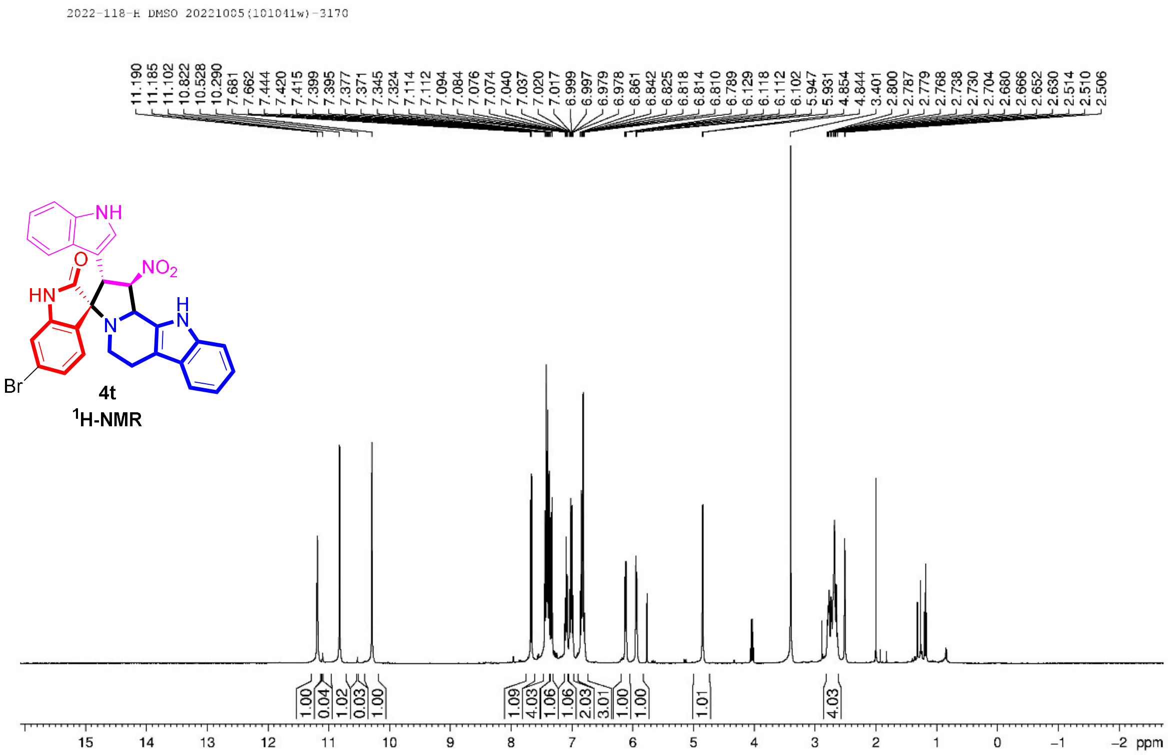
2022-118-H DMSO 20221005(101041w)-3170
11.190 11.185 11.102 10.822 10.528 10.290 7.681 7.662 7.444 7.420 7.415 7.399 7.395 7.377 7.371 7.345 7.324 7.114 7.112 7.094 7.084 7.076 7.074 7.040 7.037 7.020 7.017 6.999 6.997 6.979 6.978 6.861 6.842 6.825 6.818 6.814 6.810 6.789 6.129 6.118 6.112 6.102 5.947 5.931 4.854 4.844 3.401 2.800 2.787 2.779 2.768 2.738 2.730 2.704 2.680 2.666 2.652 2.630 2.514 2.510 2.506
NH
NO2
O
HN
H
N
N
Br
4t
1H-NMR
1.00 0.04 1.02 0.03 1.00 1.09 4.03 1.06 1.06 2.03 3.01 1.00 1.00 1.01 4.03
15 14 13 12 11 10 9 8 7 6 5 4 3 2 1 0 −1 −2 ppm

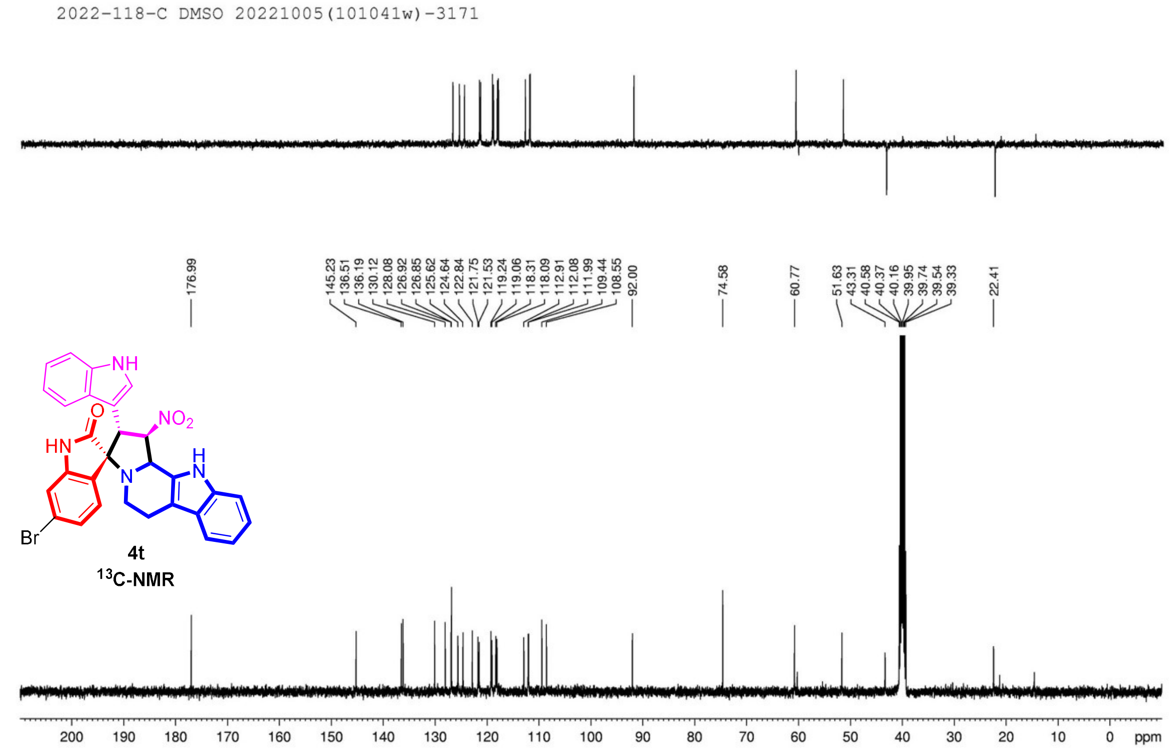
2022-118-C DMSO 20221005(101041w)-3171
176.99
145.23 136.51 136.19 130.12 128.08 126.92 126.85 125.62 124.64 122.84 121.75 121.53 119.24 119.06 118.31 118.09 112.91 112.08 111.99 109.44 108.55 92.00
74.58
60.77
51.63 43.31 40.58 40.37 40.16 39.95 39.74 39.54 39.33
22.41
NH
NO2
O
HN
H
N
N
Br
4t
13C-NMR
200 190 180 170 160 150 140 130 120 110 100 90 80 70 60 50 40 30 20 10 0 ppm

2. ^{1}H and ^{13}C NMR spectras for compounds **6**

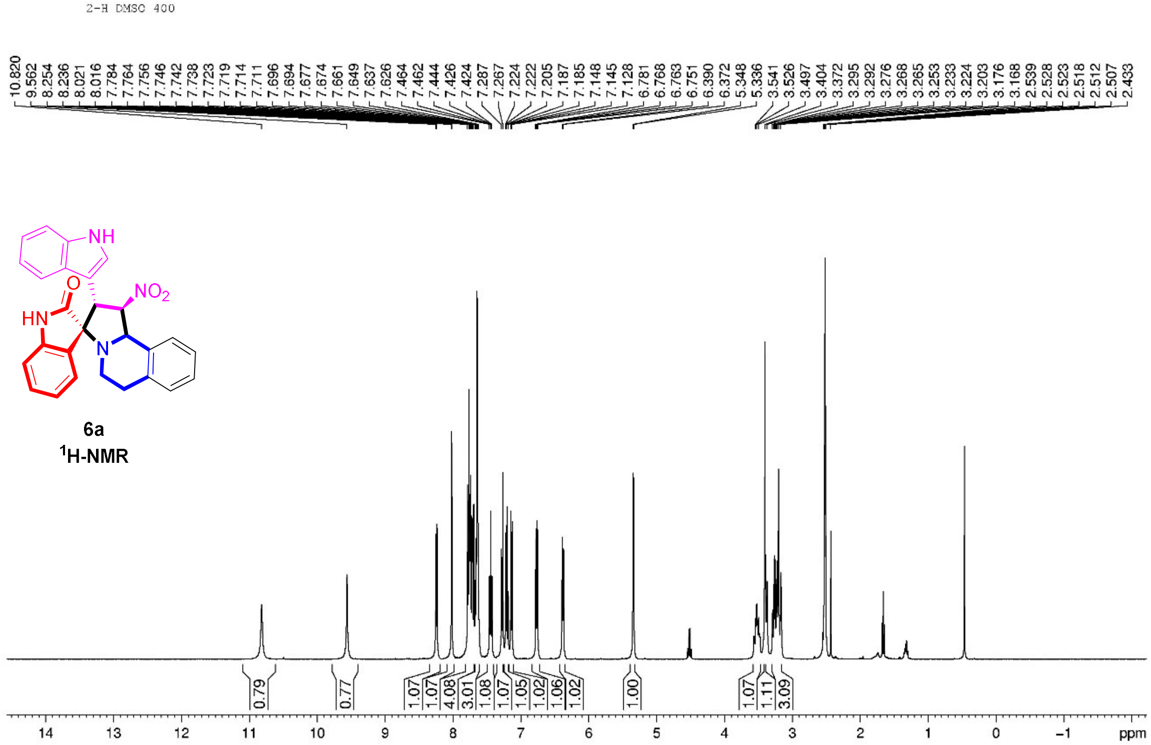

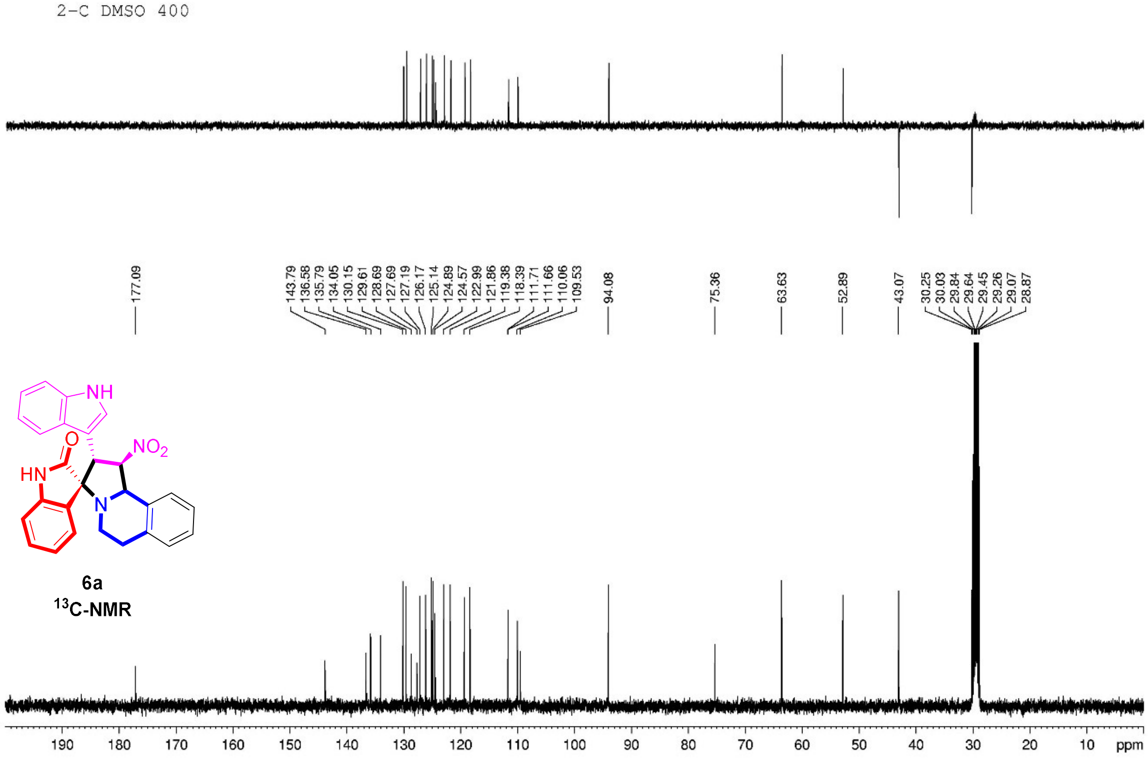

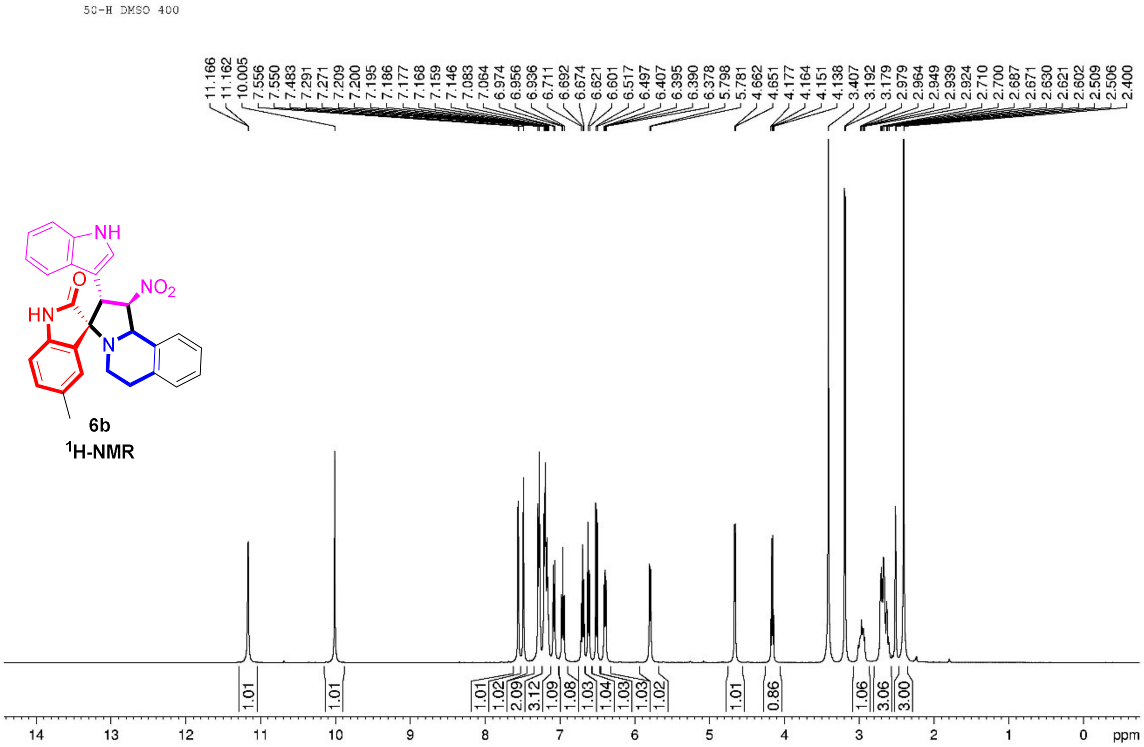
50-H DMSO 400
11.166
11.162
10.005
7.556
7.550
7.483
7.291
7.271
7.209
7.200
7.195
7.186
7.177
7.168
7.159
7.146
7.083
7.064
6.974
6.956
6.936
6.711
6.692
6.674
6.621
6.601
6.517
6.497
6.407
6.395
6.390
6.378
5.798
5.781
4.662
4.651
4.177
4.164
4.151
4.138
3.407
3.192
3.179
2.979
2.964
2.949
2.939
2.924
2.710
2.700
2.687
2.671
2.630
2.621
2.602
2.509
2.506
2.400
NH
O
NO2
HN
N
6b
1H-NMR
1.01
1.01
1.01
1.02
2.09
3.12
1.09
1.08
1.03
1.04
1.03
1.03
1.02
1.01
0.86
1.06
3.06
3.00
ppm

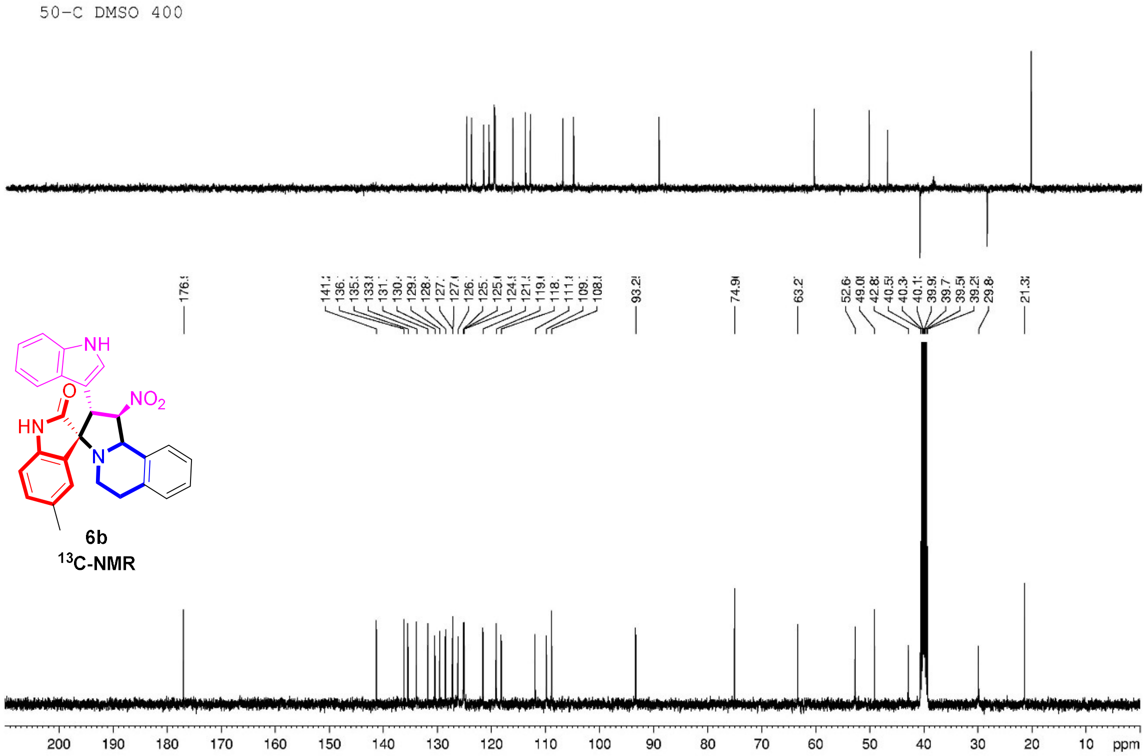
50-C DMSO 400
NH
O
NO2
HN
N
6b
13C-NMR
ppm

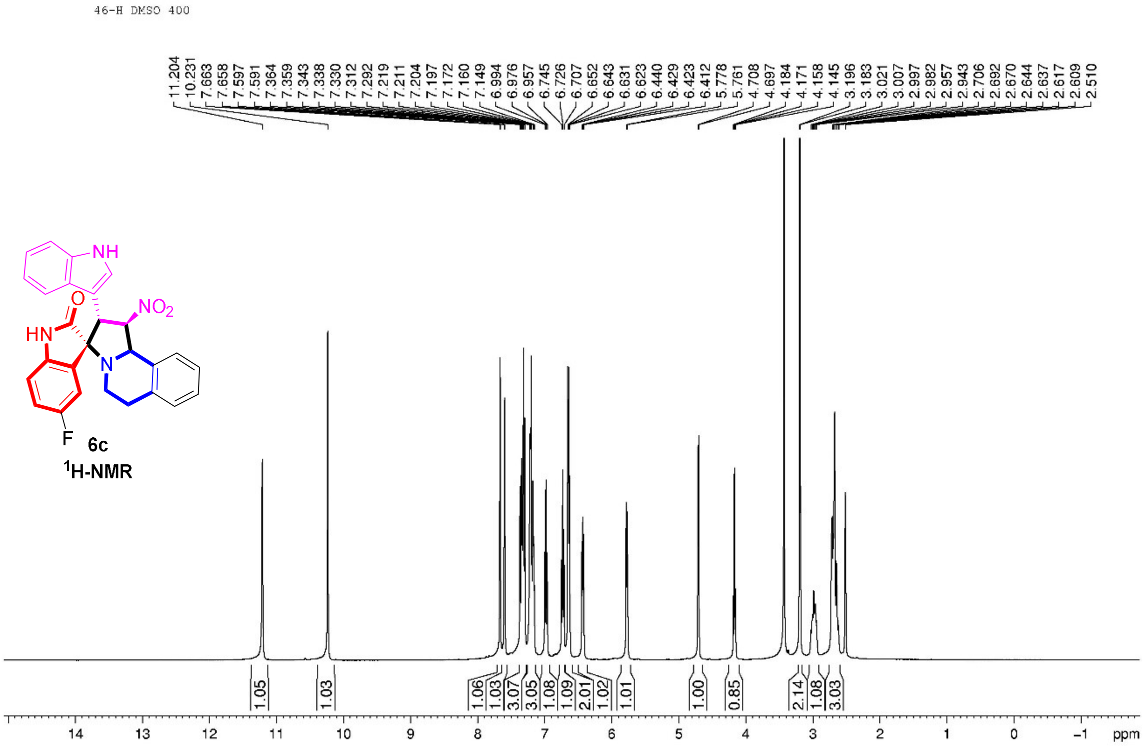
46-H DMSO 400
11.204
10.231
7.663
7.658
7.597
7.591
7.364
7.359
7.343
7.338
7.330
7.312
7.292
7.219
7.211
7.204
7.197
7.172
7.160
7.149
6.994
6.976
6.957
6.745
6.726
6.707
6.652
6.643
6.631
6.623
6.440
6.429
6.423
6.412
5.778
5.761
4.708
4.697
4.184
4.171
4.158
4.145
3.196
3.183
3.021
3.007
2.997
2.982
2.957
2.943
2.706
2.692
2.670
2.644
2.637
2.617
2.609
2.510
NH
NO2
O
HN
N
F
6c
1H-NMR
1.05
1.03
1.06
1.03
3.07
3.05
1.08
1.09
2.01
1.02
1.01
1.00
0.85
2.14
1.08
3.03
14 13 12 11 10 9 8 7 6 5 4 3 2 1 0 -1 ppm

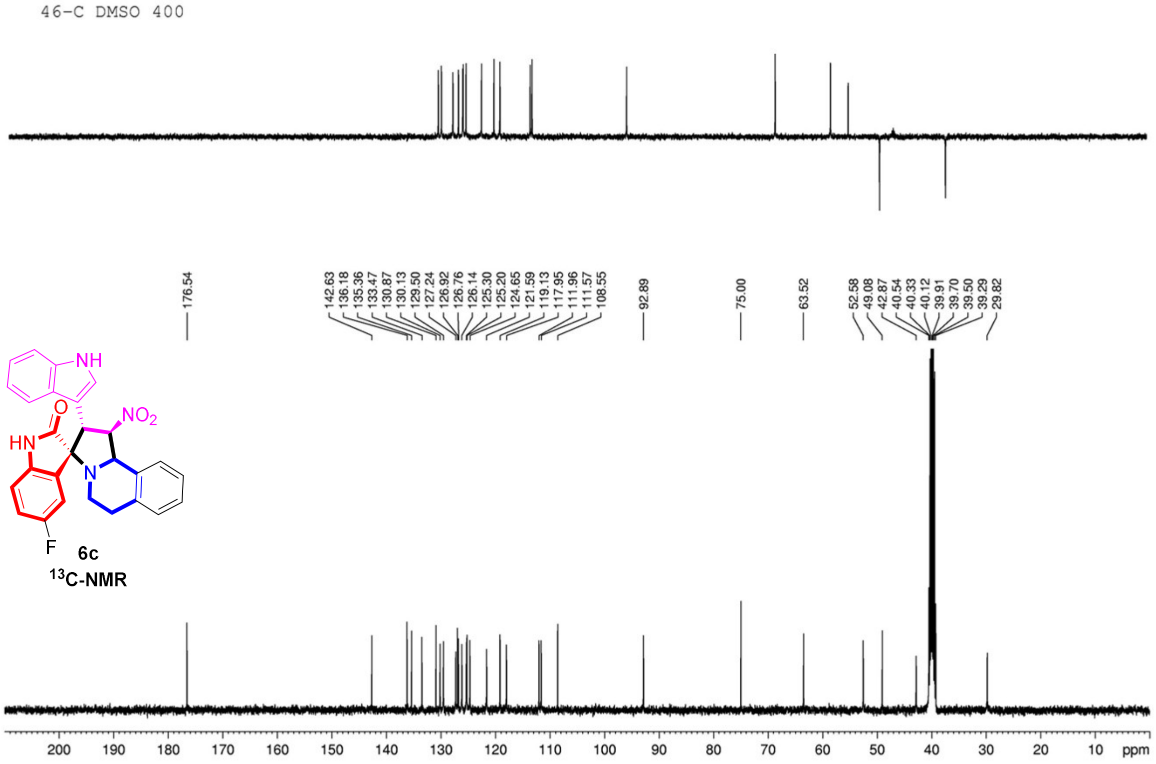
46-C DMSO 400
176.54
142.63
136.18
135.36
133.47
130.87
130.13
129.50
127.24
126.92
126.76
126.14
125.30
125.20
124.65
121.59
119.13
117.95
111.96
111.57
108.55
92.89
75.00
63.52
52.58
49.08
42.87
40.54
40.33
40.12
39.91
39.70
39.50
39.29
29.82
NH
NO2
O
HN
N
F
6c
13C-NMR
200 190 180 170 160 150 140 130 120 110 100 90 80 70 60 50 40 30 20 10 ppm

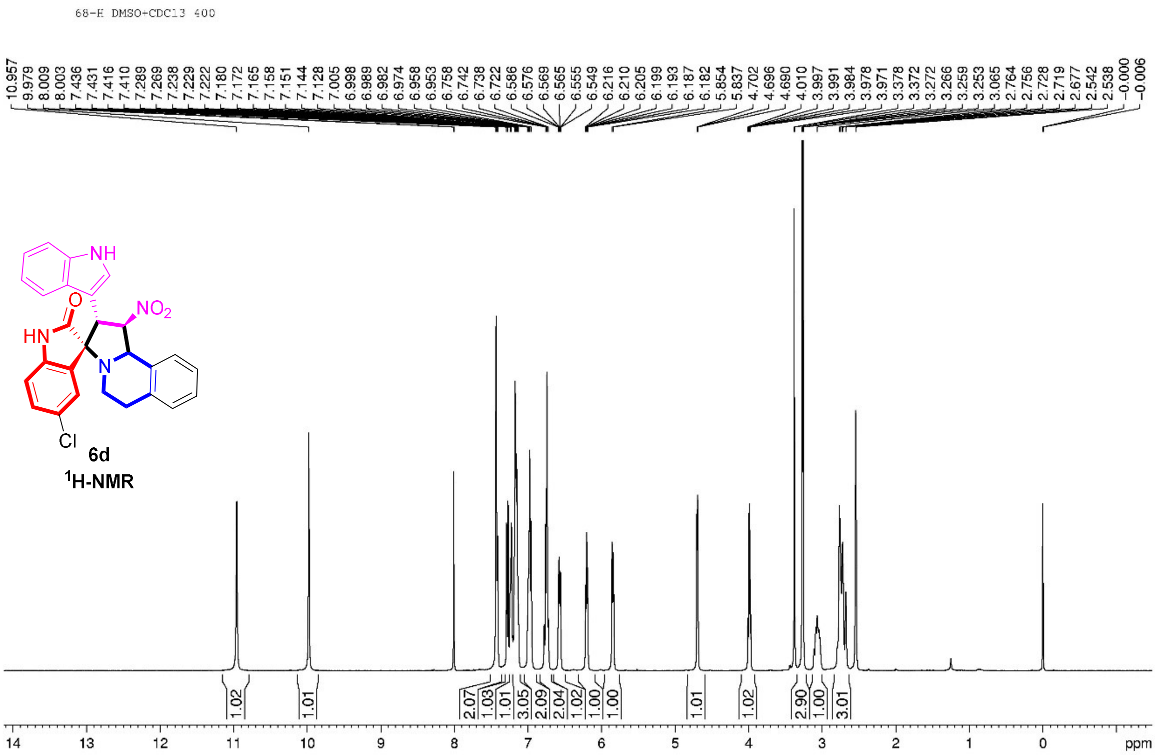
68-H DMSO-CDCl3 400
10.957
9.979
8.009
8.003
7.436
7.431
7.416
7.410
7.289
7.269
7.238
7.229
7.222
7.180
7.172
7.165
7.158
7.151
7.144
7.128
7.005
6.998
6.989
6.982
6.974
6.958
6.953
6.758
6.742
6.738
6.722
6.586
6.576
6.569
6.565
6.555
6.549
6.216
6.210
6.205
6.199
6.193
6.187
6.182
5.854
5.837
4.702
4.696
4.690
4.010
3.997
3.991
3.984
3.978
3.971
3.378
3.372
3.272
3.266
3.259
3.253
3.065
2.764
2.756
2.728
2.719
2.677
2.542
2.538
-0.000
-0.006
NH
NO2
O
HN
N
Cl
6d
1H-NMR
1.02
1.01
2.07
1.03
1.01
3.05
2.09
2.04
1.02
1.00
1.00
1.01
1.02
2.90
1.00
3.01
14
13
12
11
10
9
8
7
6
5
4
3
2
1
0
ppm

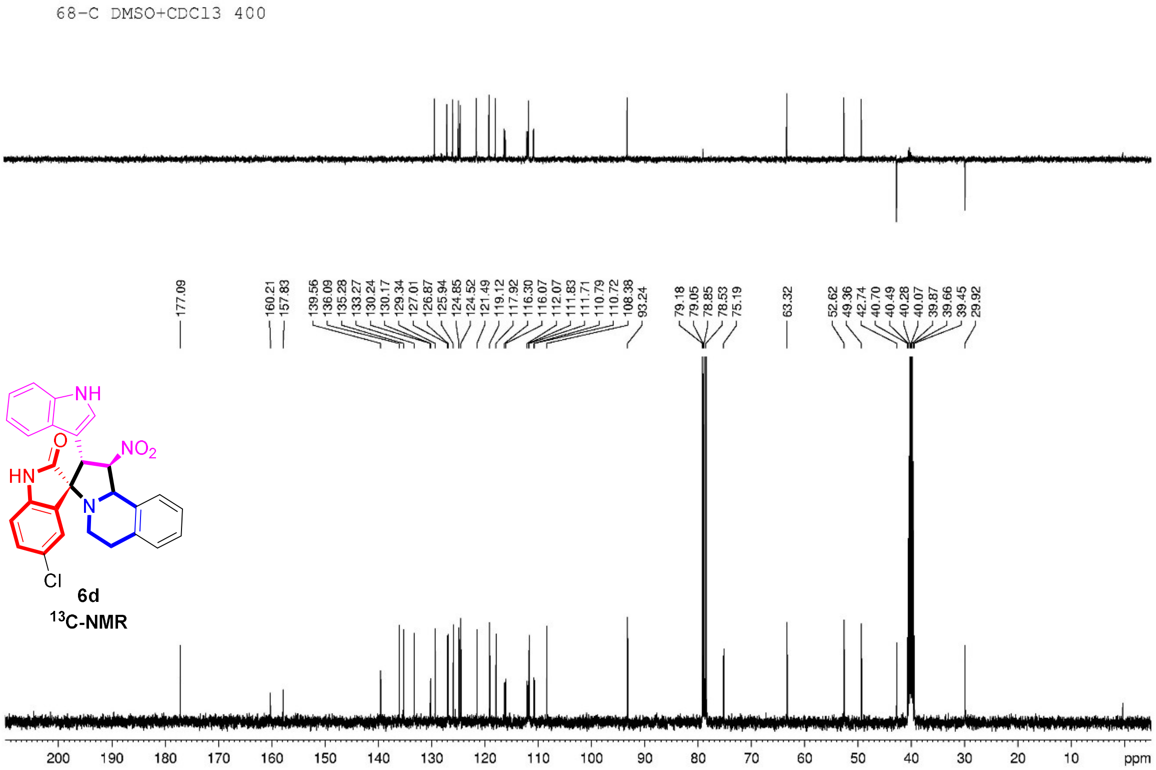
68-C DMSO+CDCl3 400
177.09
160.21
157.83
139.56
136.09
135.28
133.27
130.24
130.17
129.34
127.01
126.87
125.94
124.85
124.52
121.49
119.12
117.92
116.30
116.07
112.07
111.83
111.71
110.79
110.72
108.38
93.24
79.18
79.05
78.85
78.53
75.19
63.32
52.62
49.36
42.74
40.70
40.49
40.28
40.07
39.87
39.66
39.45
29.92
NH
NO2
O
HN
N
Cl
6d
13C-NMR
200
190
180
170
160
150
140
130
120
110
100
90
80
70
60
50
40
30
20
10
ppm

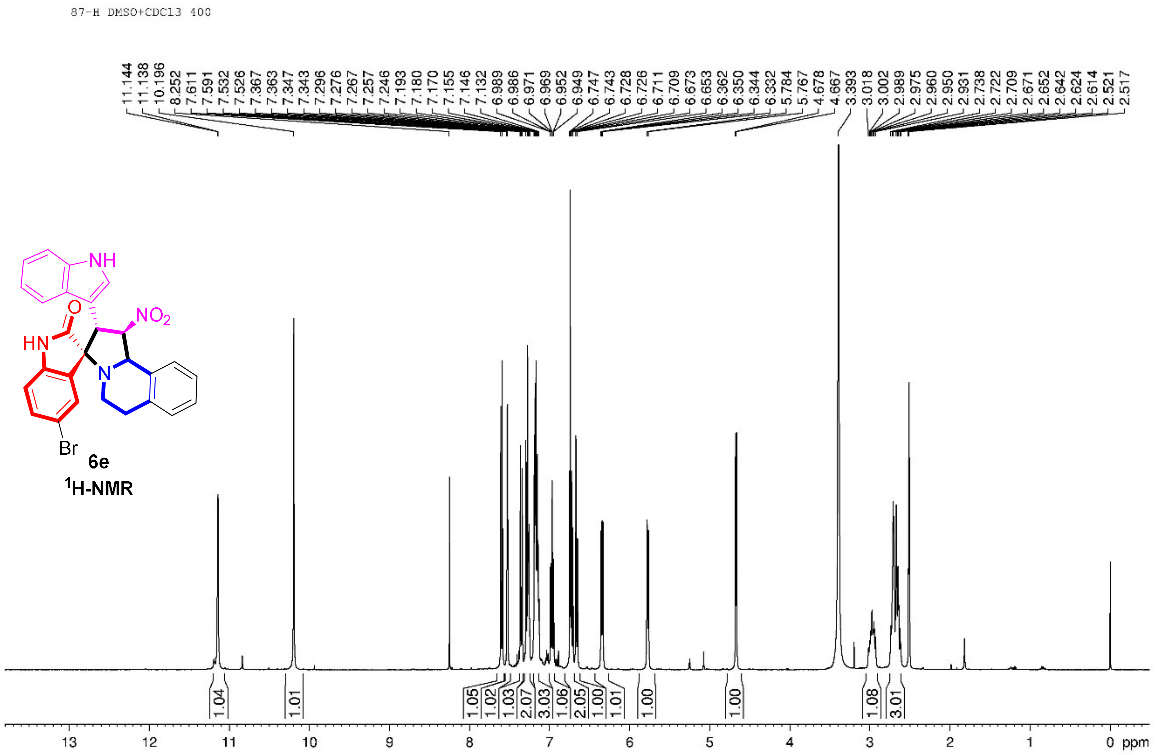
87-H DMSO+CDCl3 400
11.144
11.138
10.196
8.252
7.611
7.591
7.532
7.526
7.367
7.363
7.347
7.343
7.296
7.276
7.267
7.257
7.246
7.193
7.180
7.170
7.155
7.146
7.132
6.989
6.986
6.971
6.969
6.952
6.949
6.747
6.743
6.728
6.726
6.711
6.709
6.673
6.653
6.362
6.350
6.344
6.332
5.784
5.767
4.678
4.667
3.393
3.018
3.002
2.989
2.975
2.960
2.950
2.931
2.738
2.722
2.709
2.671
2.652
2.642
2.624
2.614
2.521
2.517
NH
NO2
HN
O
N
Br
6e
1H-NMR
1.04
1.01
1.05
1.02
1.03
2.07
3.03
1.06
2.05
1.00
1.01
1.00
1.00
1.08
3.01
13
12
11
10
9
8
7
6
5
4
3
2
1
0 ppm

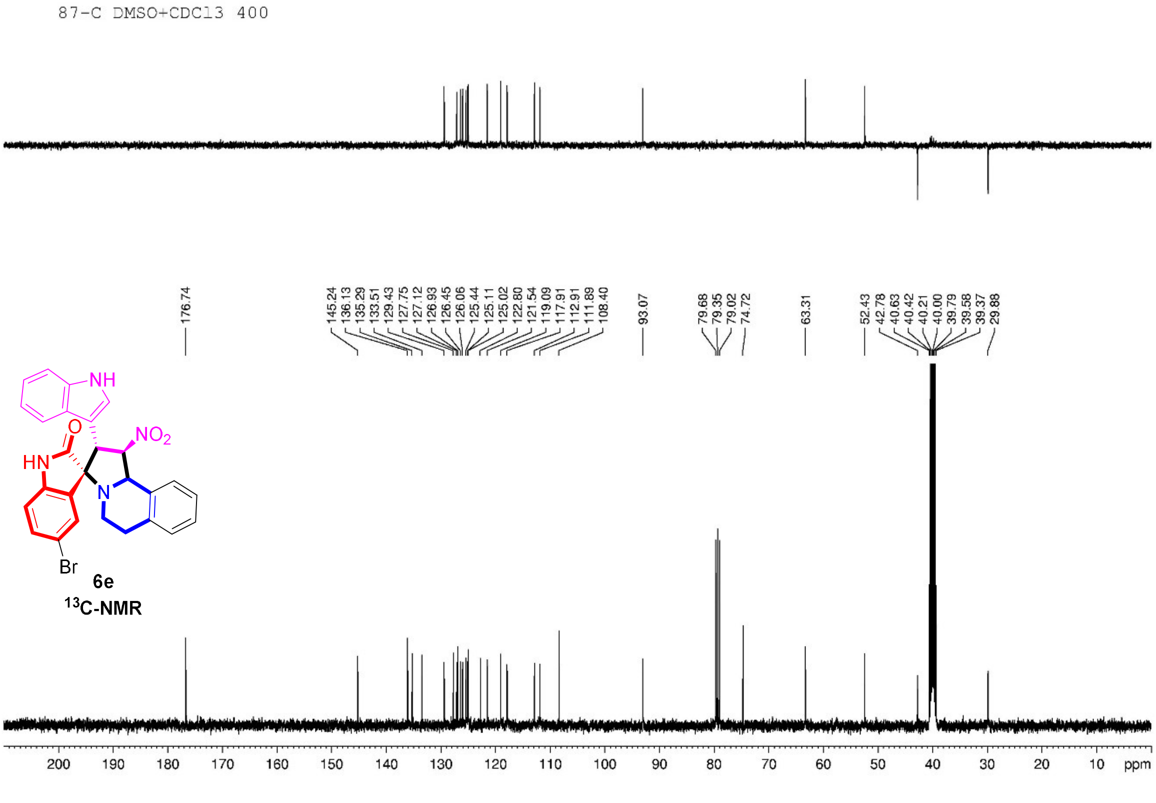
87-C DMSO+CDCl3 400
176.74
145.24
136.13
135.29
133.51
129.43
127.75
127.12
126.93
126.45
126.06
125.44
125.11
125.02
122.80
121.54
119.09
117.91
112.91
111.89
108.40
93.07
79.68
79.35
79.02
74.72
63.31
52.43
42.78
40.63
40.42
40.21
40.00
39.79
39.58
39.37
29.88
NH
NO2
HN
O
N
Br
6e
13C-NMR
200
190
180
170
160
150
140
130
120
110
100
90
80
70
60
50
40
30
20
10
ppm

73-H DMSO+CDCl3 400

6f
^{1}H-NMR

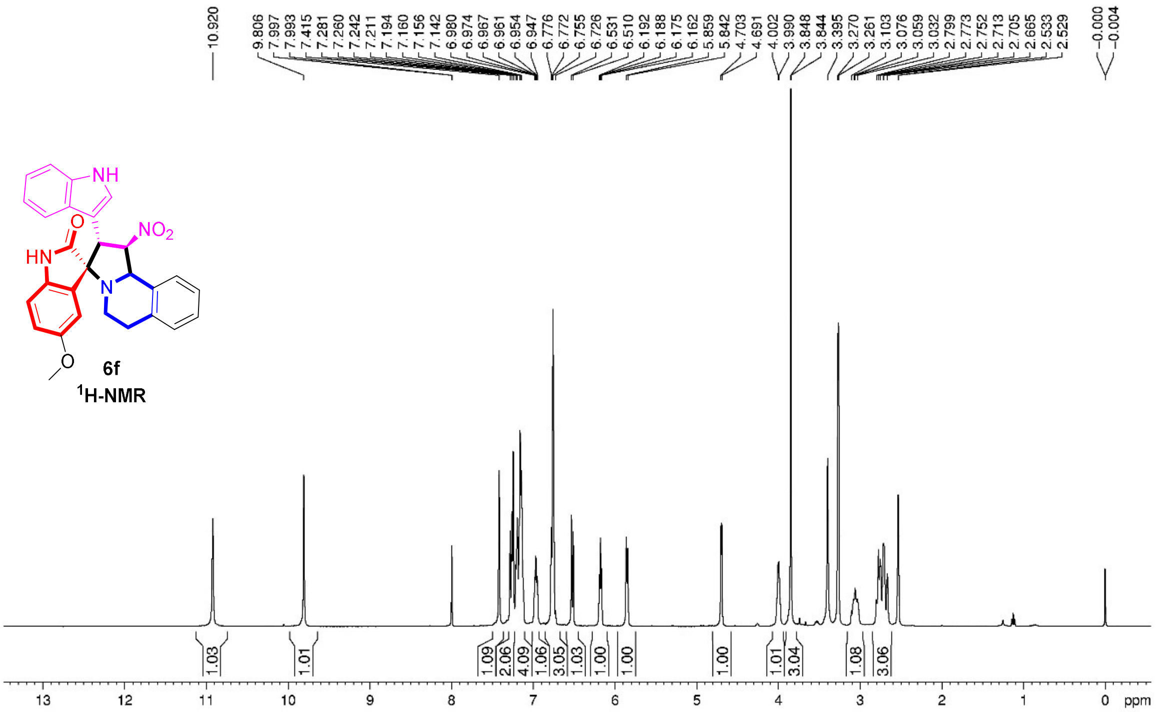

73-C DMSO+CDCl3 400

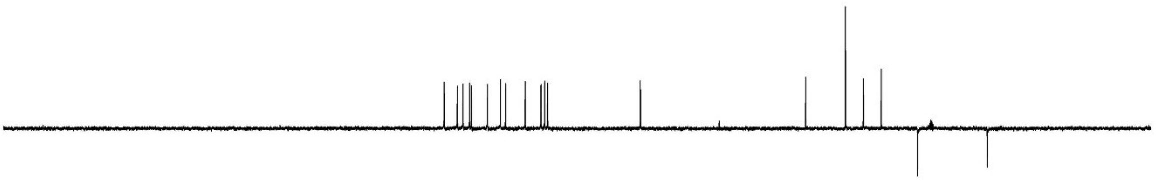

6f
^{13}C-NMR

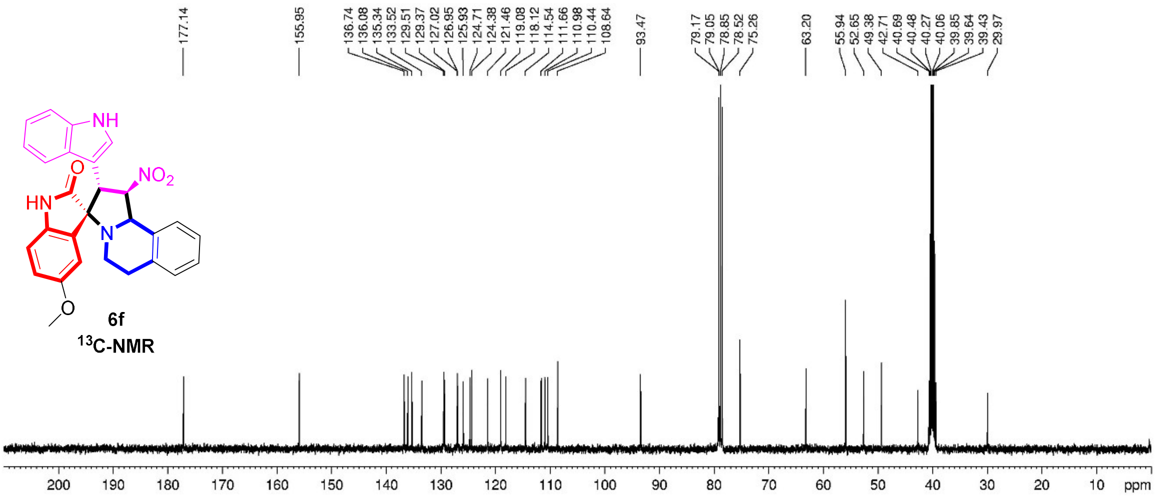

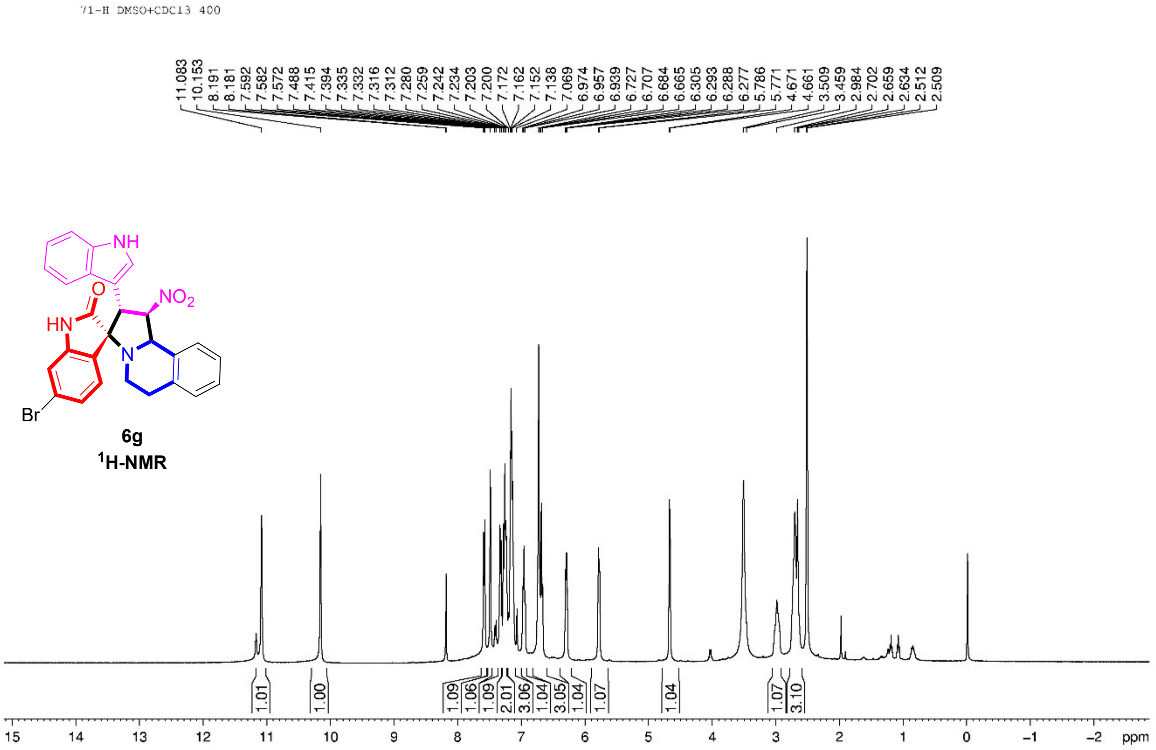
71-H DMSO+CDCl3 400
11.083 10.153 8.191 8.181 7.592 7.582 7.572 7.488 7.415 7.394 7.335 7.332 7.316 7.312 7.280 7.259 7.242 7.234 7.203 7.200 7.172 7.162 7.152 7.138 7.069 6.974 6.957 6.939 6.727 6.707 6.684 6.665 6.305 6.293 6.288 6.277 5.786 5.771 4.671 4.661 3.509 3.459 2.984 2.702 2.659 2.634 2.512 2.509
NH
NO2
HN
O
N
Br
6g
1H-NMR
1.01 1.00 1.09 1.06 1.09 2.01 3.06 1.04 3.05 1.04 1.07 1.04 1.07 3.10
15 14 13 12 11 10 9 8 7 6 5 4 3 2 1 0 –1 –2 ppm

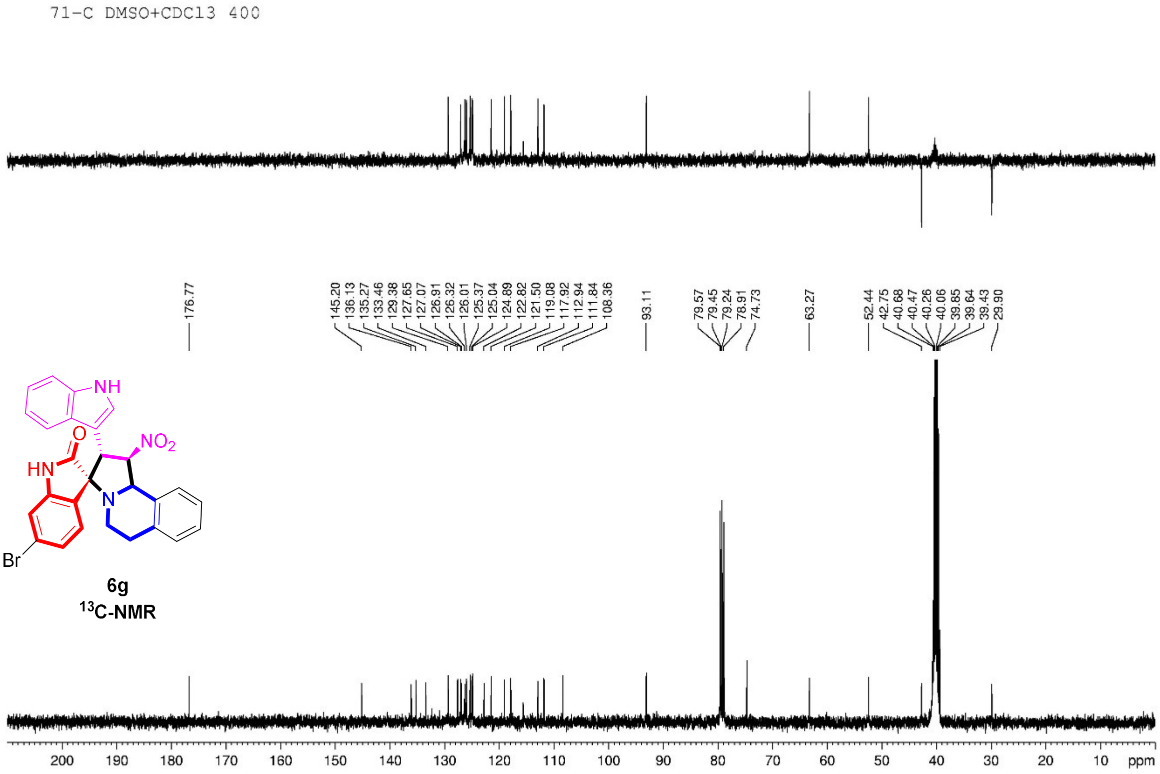
71-C DMSO+CDCl3 400
176.77
145.20 136.13 135.27 133.46 129.38 127.65 127.07 126.91 126.32 126.01 125.37 125.04 124.89 122.82 121.50 119.08 117.92 112.94 111.84 108.36
93.11
79.57 79.45 79.24 78.91 74.73
63.27
52.44 42.75 40.68 40.47 40.26 40.06 39.85 39.64 39.43 29.90
NH
NO2
HN
O
N
Br
6g
13C-NMR
200 190 180 170 160 150 140 130 120 110 100 90 80 70 60 50 40 30 20 10 ppm

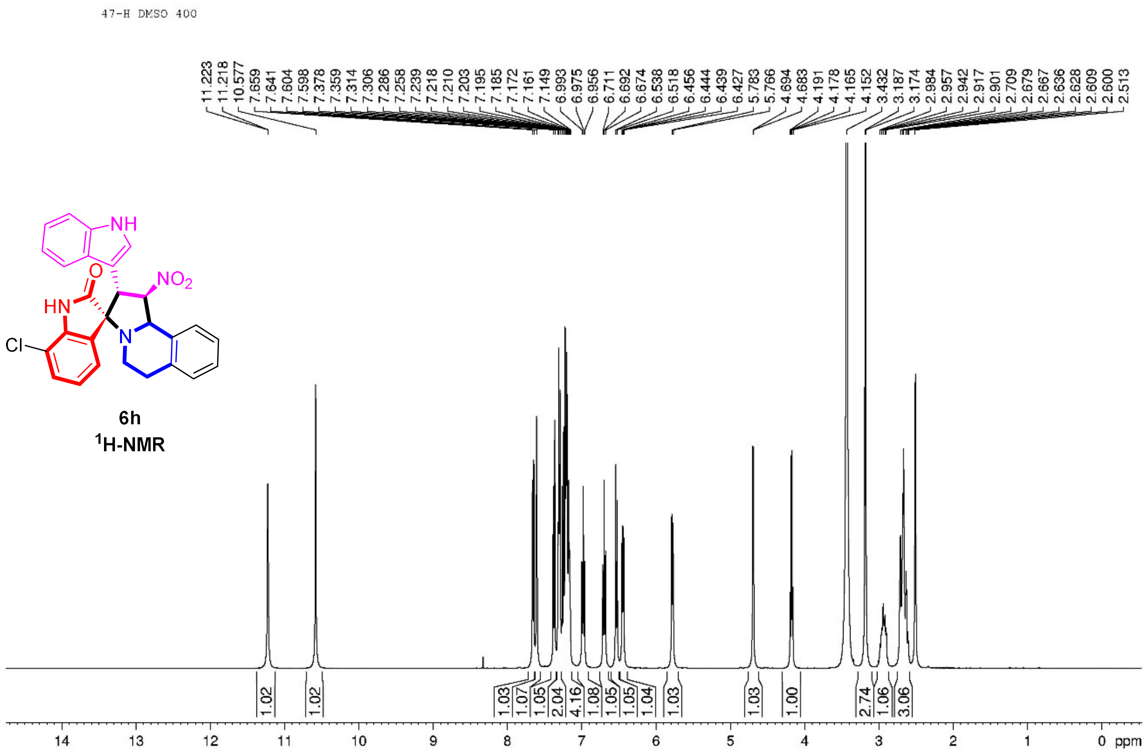
47-H DMSO 400
6h
1H-NMR
ppm

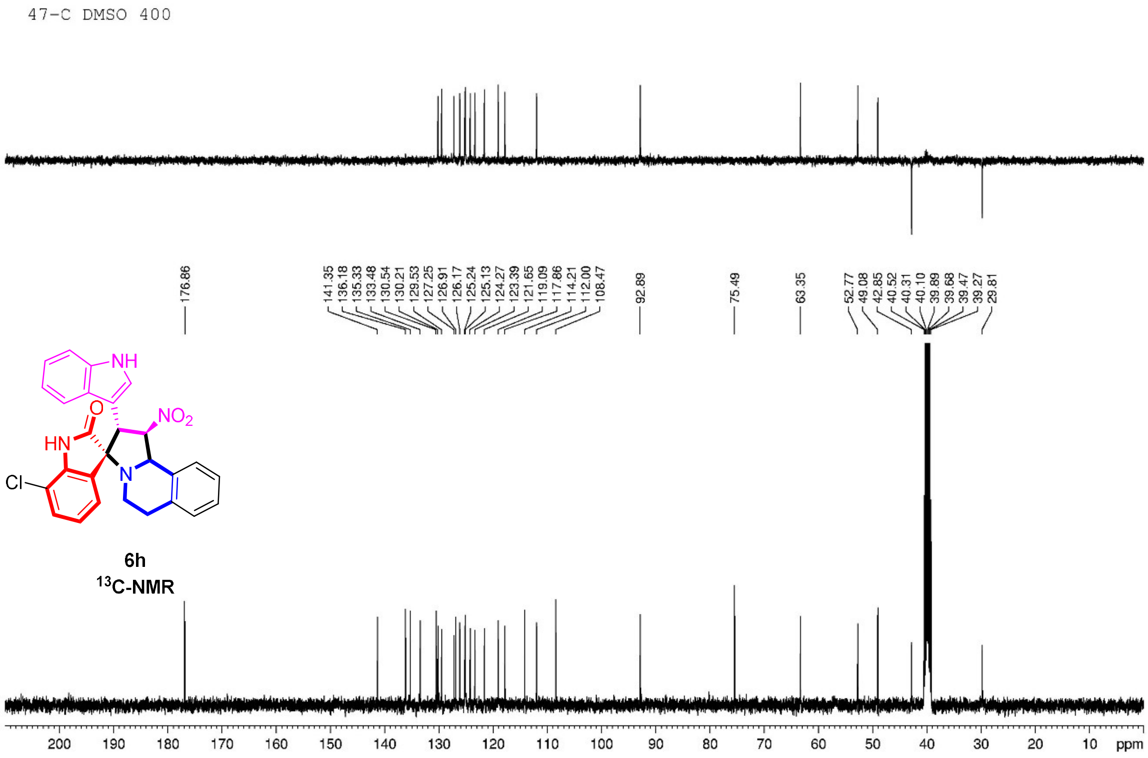
47-C DMSO 400
6h
13C-NMR
ppm

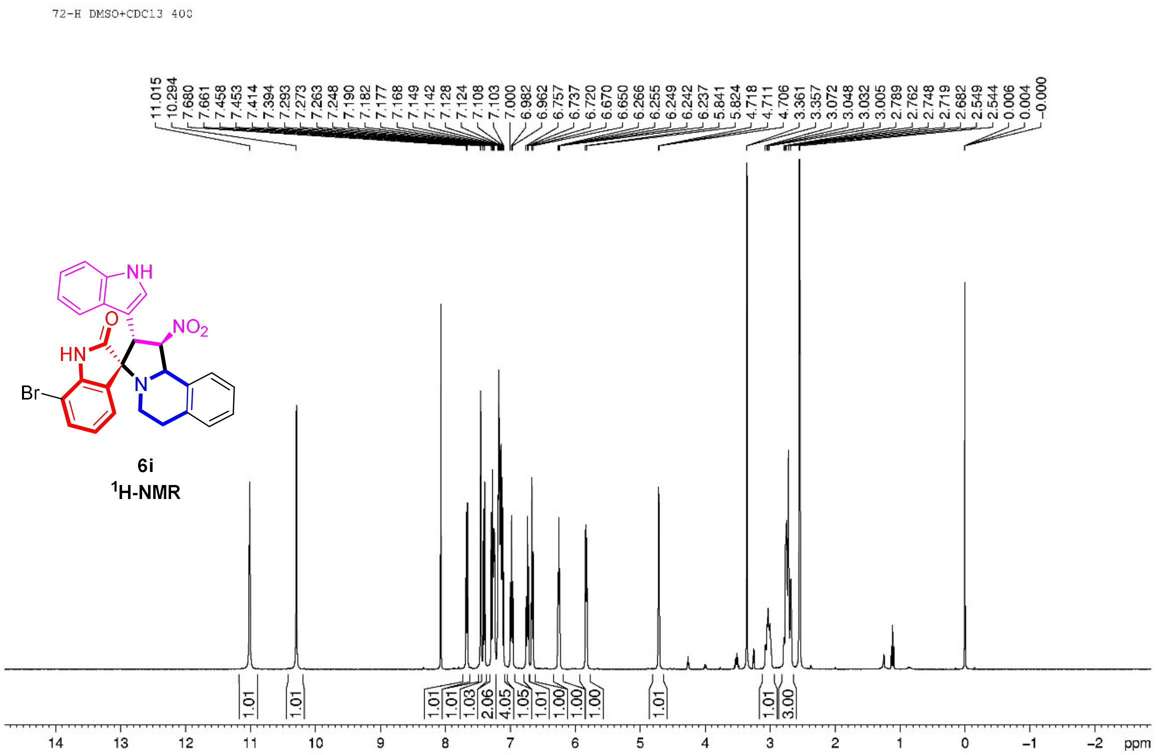
72-H DMSO+CDCl3 400
11.015
10.294
7.680
7.661
7.458
7.453
7.414
7.394
7.293
7.273
7.263
7.248
7.190
7.182
7.177
7.168
7.149
7.142
7.128
7.124
7.108
7.103
7.000
6.982
6.962
6.757
6.737
6.720
6.670
6.650
6.266
6.255
6.249
6.242
6.237
5.841
5.824
4.718
4.711
4.706
3.361
3.357
3.072
3.048
3.032
3.005
2.789
2.762
2.748
2.719
2.682
2.549
2.544
0.006
0.004
-0.000
NH
NO2
O
HN
N
Br
6i
1H-NMR
1.01
1.01
1.01
1.01
1.03
2.06
4.05
1.05
1.01
1.00
1.00
1.00
1.01
1.01
3.00
14 13 12 11 10 9 8 7 6 5 4 3 2 1 0 -1 -2 ppm

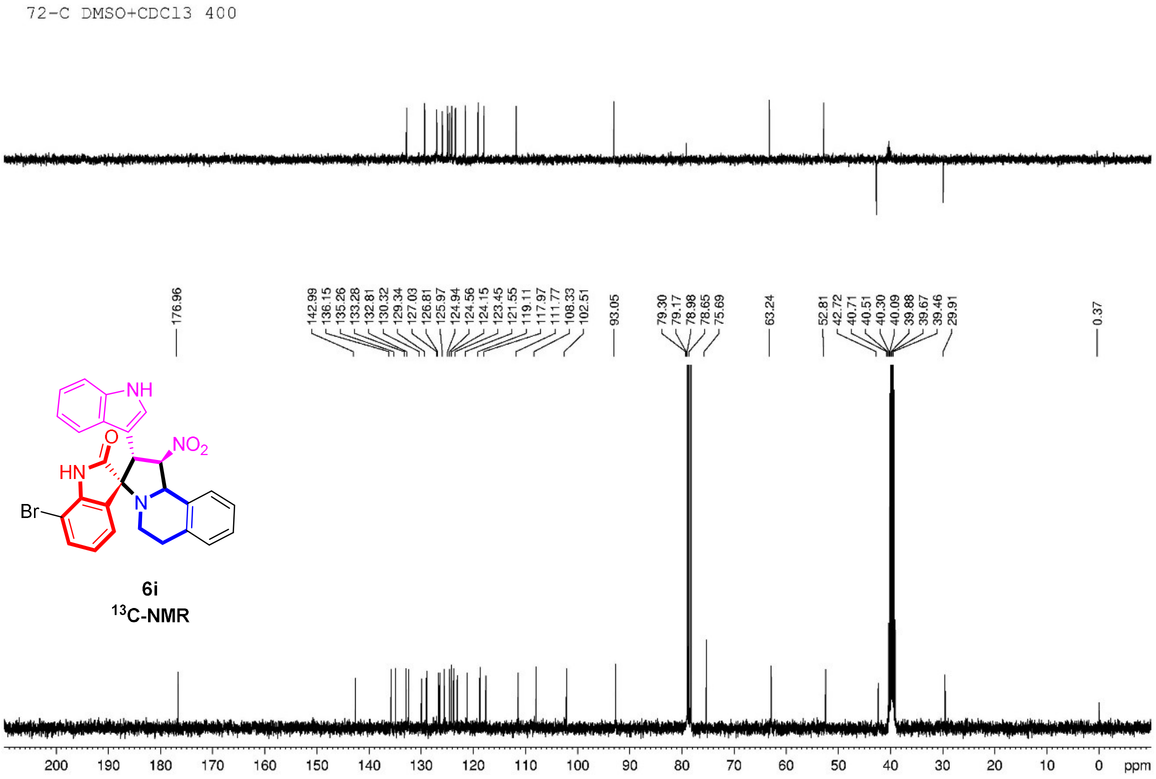
72-C DMSO+CDCl3 400
176.96
142.99
136.15
135.26
133.28
132.81
130.32
129.34
127.03
126.81
125.97
124.94
124.56
124.15
123.45
121.55
119.11
117.97
111.77
108.33
102.51
93.05
79.30
79.17
78.98
78.65
75.69
63.24
52.81
42.72
40.71
40.51
40.30
40.09
39.88
39.67
39.46
29.91
0.37
NH
NO2
O
HN
N
Br
6i
13C-NMR
200 190 180 170 160 150 140 130 120 110 100 90 80 70 60 50 40 30 20 10 0 ppm

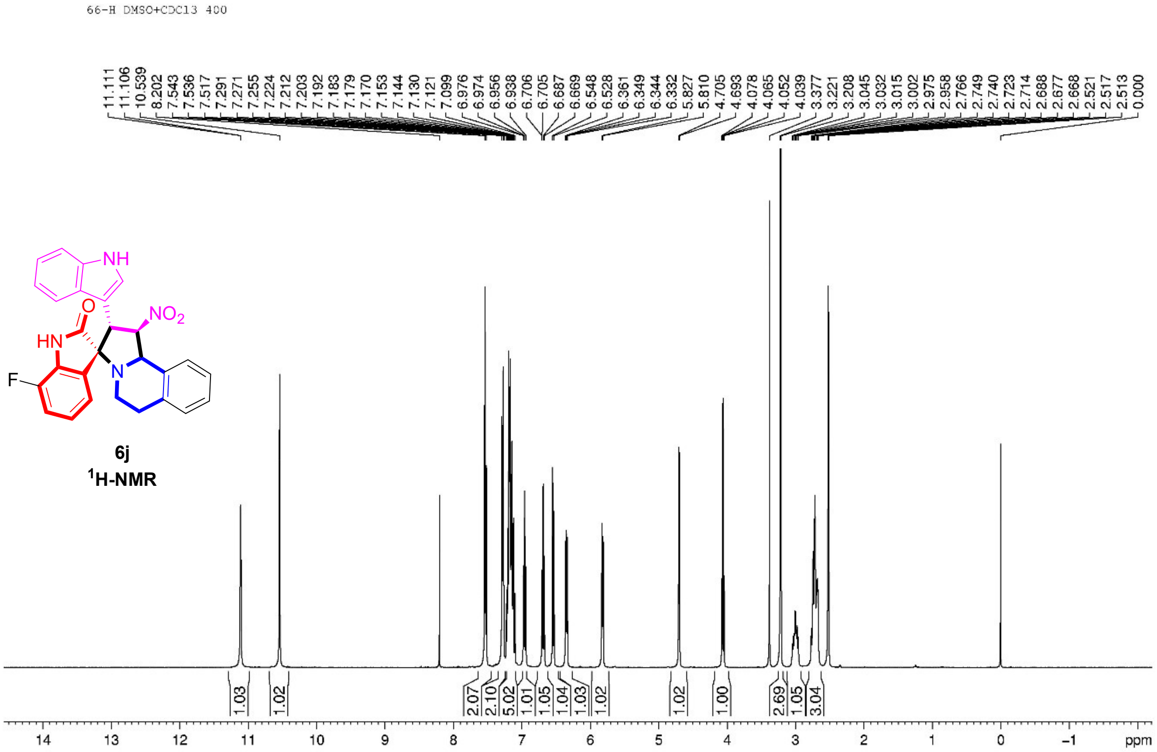

6j
^{1}H-NMR

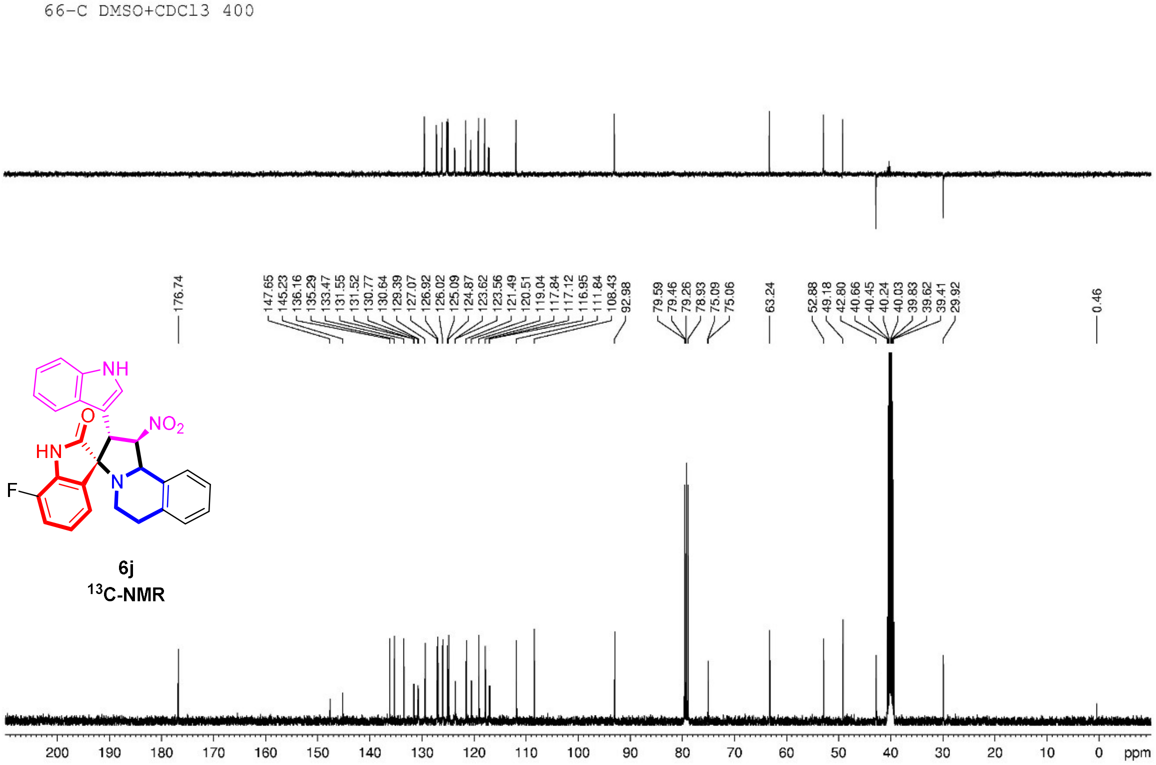

6j
^{13}C-NMR

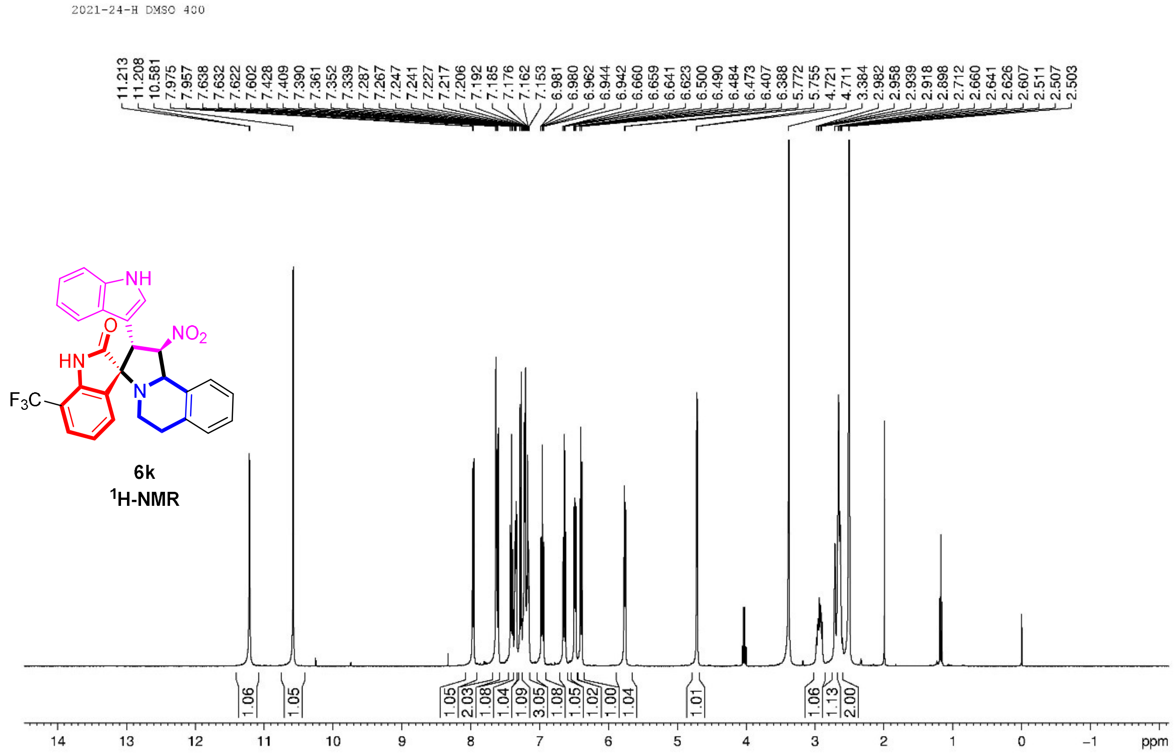
2021-24-H DMSO 400
11.213 11.208 10.581 7.975 7.957 7.638 7.632 7.622 7.602 7.428 7.409 7.390 7.361 7.352 7.339 7.287 7.267 7.247 7.241 7.227 7.217 7.206 7.192 7.185 7.176 7.162 7.153 6.981 6.980 6.962 6.944 6.942 6.660 6.659 6.641 6.623 6.500 6.490 6.484 6.473 6.407 6.388 5.772 5.755 4.721 4.711 3.384 2.982 2.958 2.939 2.918 2.898 2.712 2.660 2.641 2.626 2.607 2.511 2.507 2.503
NH
NO2
O
HN
F3C
N
6k
1H-NMR
1.06 1.05 1.05 2.03 1.08 1.04 1.09 3.05 1.08 1.05 1.02 1.00 1.04 1.01 1.06 1.13 2.00
14 13 12 11 10 9 8 7 6 5 4 3 2 1 0 –1 ppm

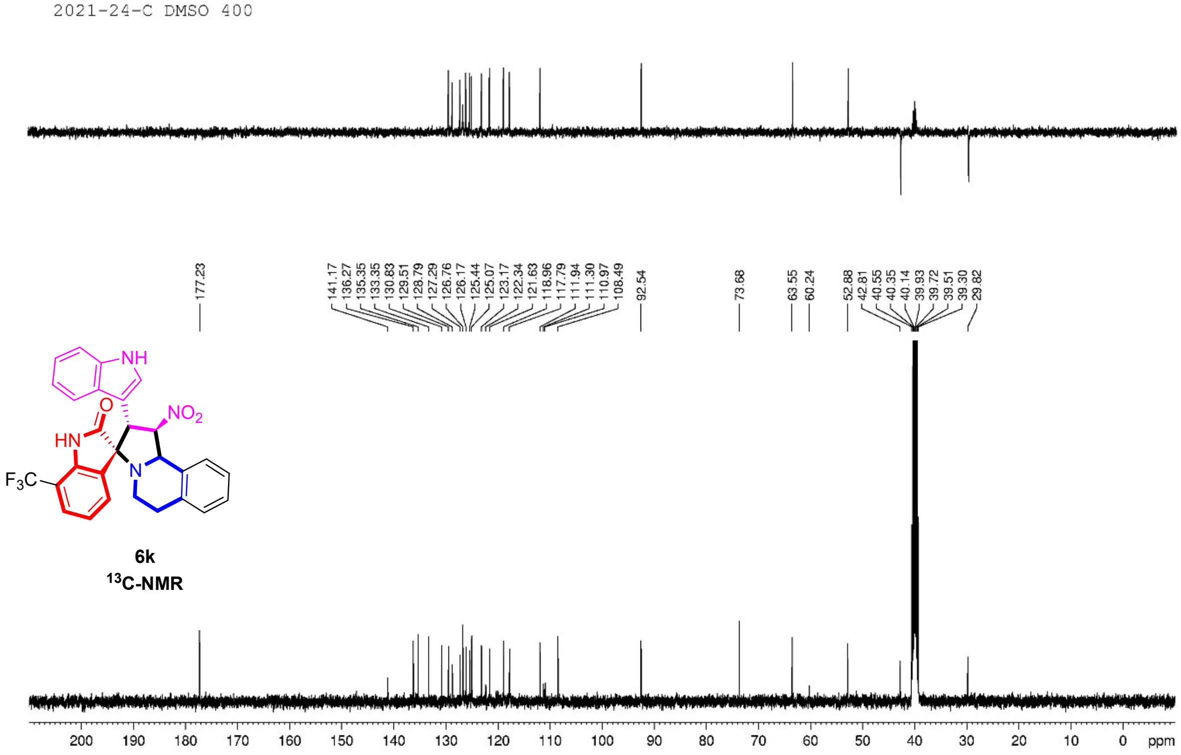
2021-24-C DMSO 400
177.23
141.17 136.27 135.35 133.35 130.83 129.51 128.79 127.29 126.76 126.17 125.44 125.07 123.17 122.34 121.63 118.96 117.79 111.94 111.30 110.97 108.49
92.54
73.68
63.55 60.24
52.88 42.81 40.55 40.35 40.14 39.93 39.72 39.51 39.30 29.82
NH
NO2
O
HN
F3C
N
6k
13C-NMR
200 190 180 170 160 150 140 130 120 110 100 90 80 70 60 50 40 30 20 10 0 ppm

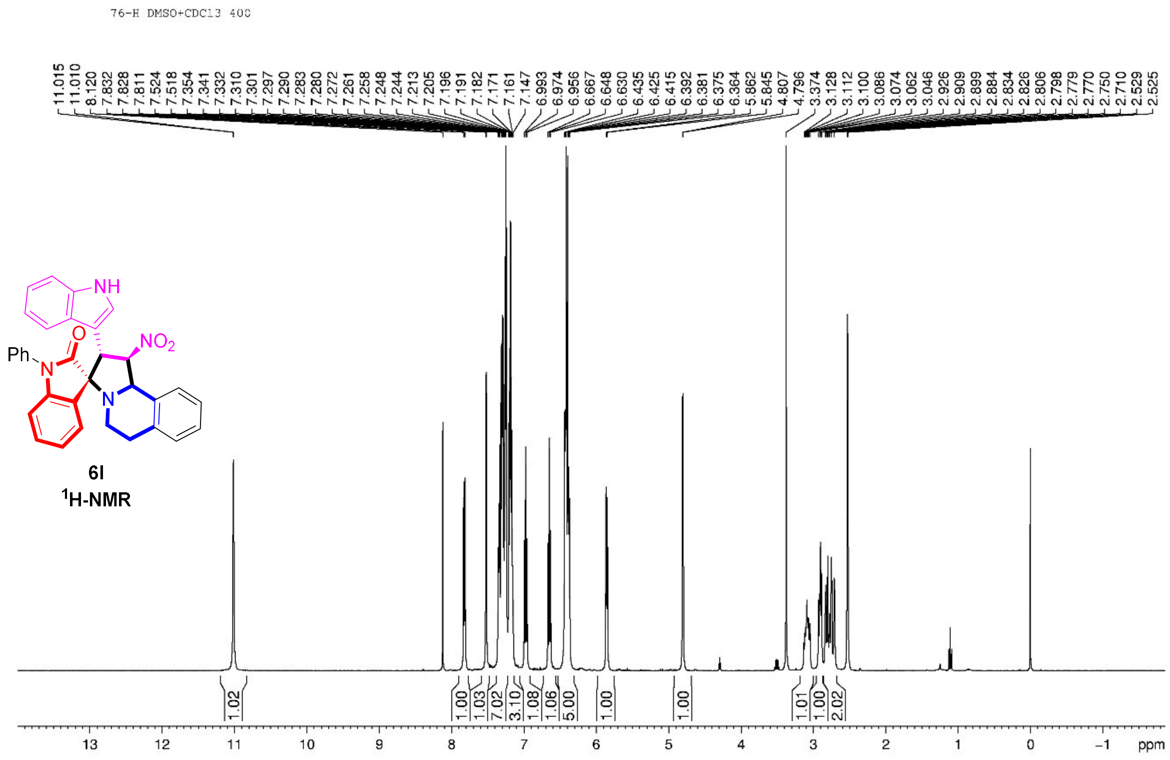
76-H DMSO+CDCl3 400
11.015
11.010
8.120
7.832
7.828
7.811
7.524
7.518
7.354
7.341
7.332
7.310
7.301
7.297
7.290
7.283
7.280
7.272
7.261
7.258
7.248
7.244
7.213
7.205
7.196
7.191
7.182
7.171
7.161
7.147
6.993
6.974
6.956
6.667
6.648
6.630
6.435
6.425
6.415
6.392
6.381
6.375
6.364
5.862
5.845
4.807
4.796
3.374
3.128
3.112
3.100
3.086
3.074
3.062
3.046
2.926
2.909
2.899
2.884
2.834
2.826
2.806
2.798
2.779
2.770
2.750
2.710
2.529
2.525
NH
Ph
N
O
NO2
N
6l
1H-NMR
1.02
1.00
1.03
7.02
3.10
1.08
1.06
5.00
1.00
1.00
1.01
1.00
2.02
13
12
11
10
9
8
7
6
5
4
3
2
1
0
−1
ppm

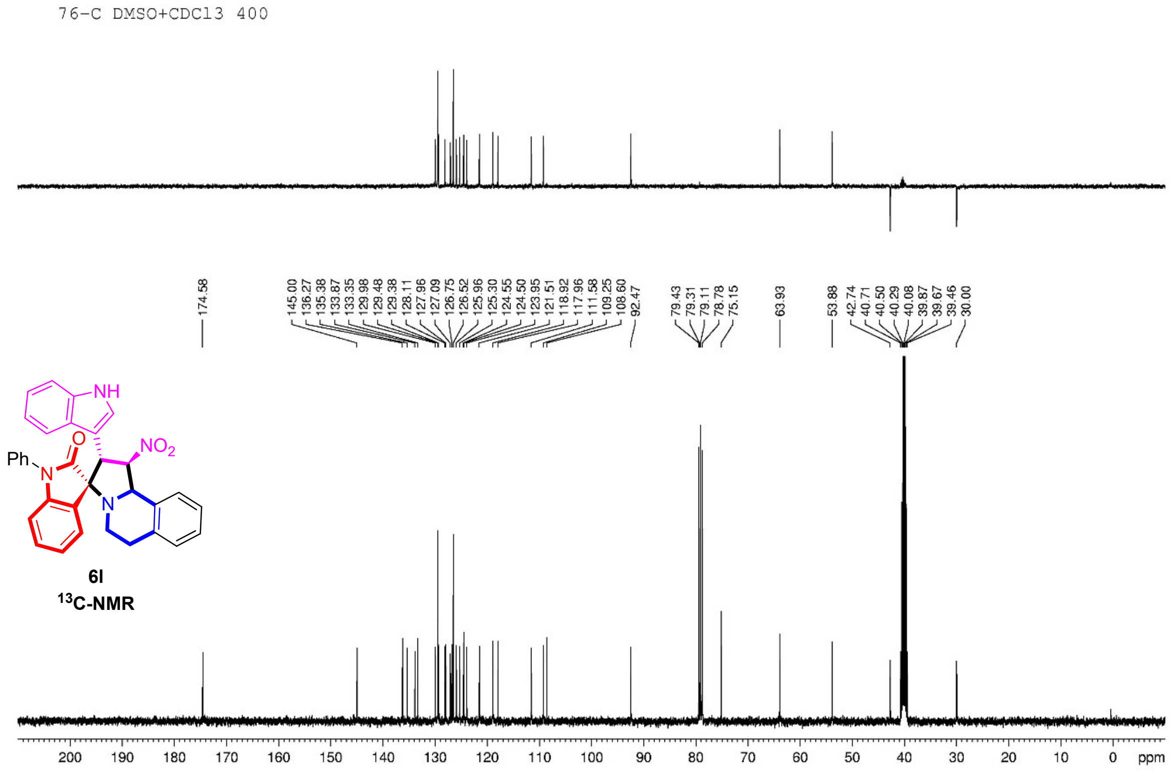
76-C DMSO+CDCl3 400
174.58
145.00
136.27
135.38
133.87
133.35
129.98
129.48
129.38
128.11
127.96
127.09
126.75
126.52
125.96
125.30
124.55
124.50
123.95
121.51
118.92
117.96
111.58
109.25
108.60
92.47
79.43
79.31
79.11
78.78
75.15
63.93
53.88
42.74
40.71
40.50
40.29
40.08
39.87
39.67
39.46
30.00
NH
Ph
N
O
NO2
N
6l
13C-NMR
200
190
180
170
160
150
140
130
120
110
100
90
80
70
60
50
40
30
20
10
0
ppm

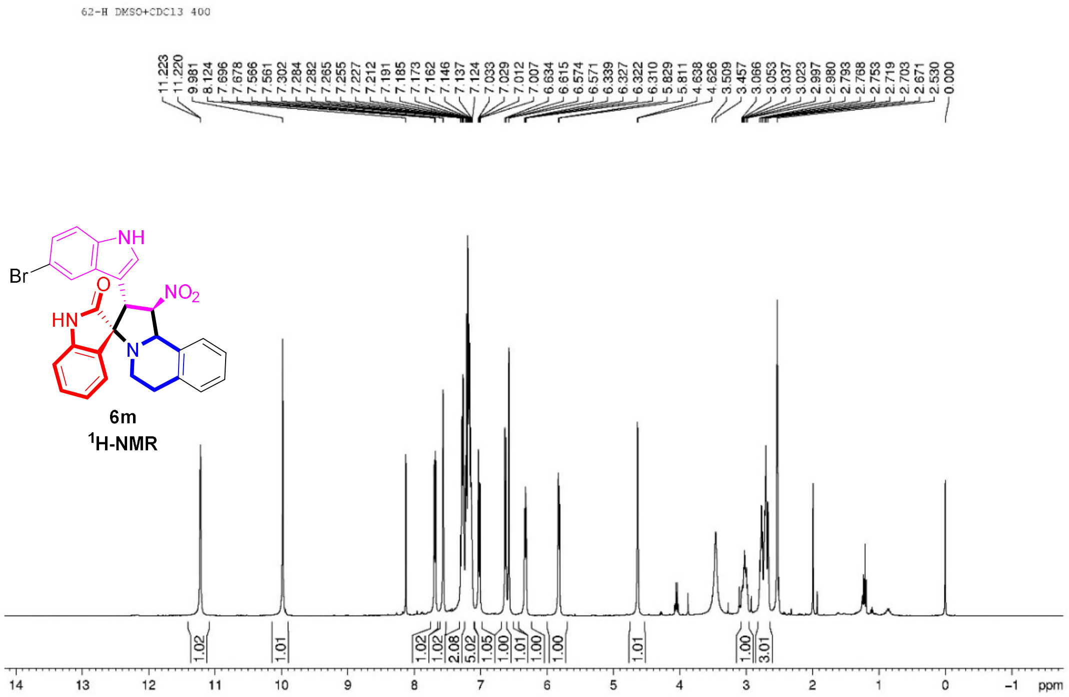
62-H DMSO+CDCl3 400
11.223
11.220
9.981
8.124
7.696
7.678
7.566
7.561
7.302
7.284
7.282
7.265
7.255
7.227
7.212
7.191
7.185
7.173
7.162
7.146
7.137
7.124
7.033
7.029
7.012
7.007
6.634
6.615
6.574
6.571
6.339
6.327
6.322
6.310
5.829
5.811
4.638
4.626
3.509
3.457
3.066
3.053
3.037
3.023
2.997
2.980
2.793
2.768
2.753
2.719
2.703
2.671
2.530
0.000
Br
NH
O
HN
NO2
N
6m
1H-NMR
1.02
1.01
1.02
1.02
2.08
5.02
1.05
1.00
1.01
1.00
1.00
1.01
1.00
3.01
14 13 12 11 10 9 8 7 6 5 4 3 2 1 0 −1 ppm

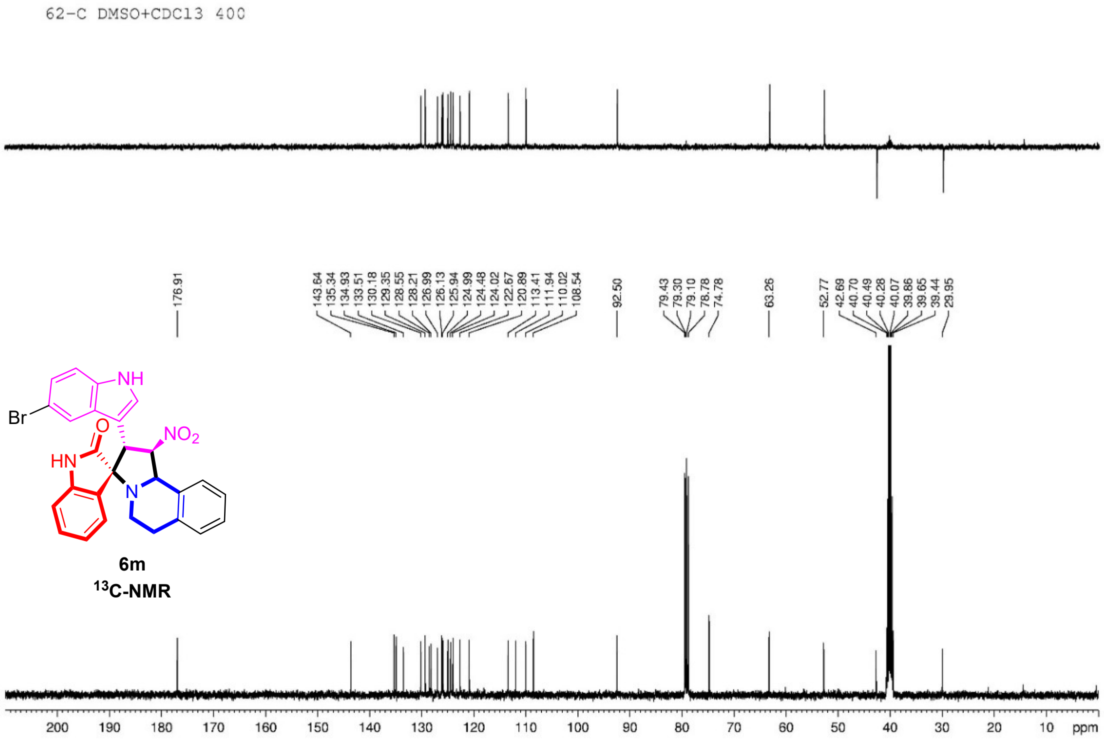
62-C DMSO+CDCl3 400
176.91
143.64
135.34
134.93
133.51
130.18
129.35
128.55
128.21
126.99
126.13
125.94
124.99
124.48
124.02
122.67
120.89
113.41
111.94
110.02
108.54
92.50
79.43
79.30
79.10
78.78
74.78
63.26
52.77
42.69
40.70
40.49
40.28
40.07
39.86
39.65
39.44
29.95
Br
NH
O
HN
NO2
N
6m
13C-NMR
200 190 180 170 160 150 140 130 120 110 100 90 80 70 60 50 40 30 20 10 ppm

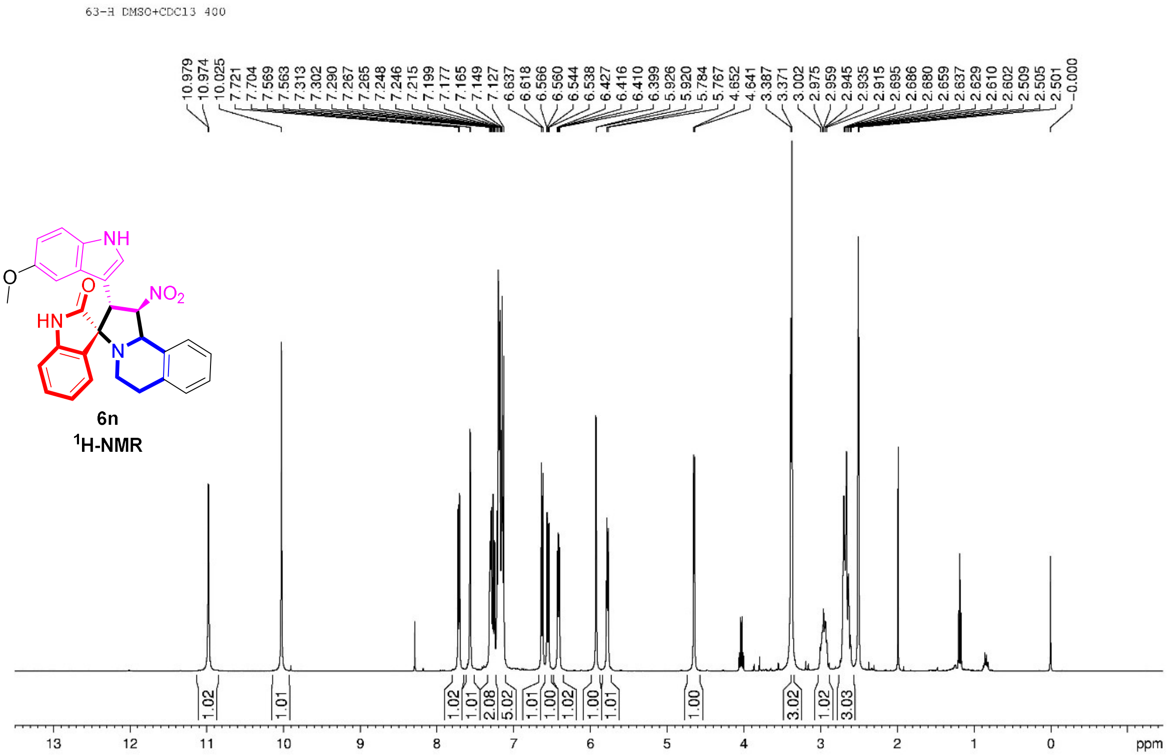
63-H DMSO+CDCl3 400
10.979
10.974
10.025
7.721
7.704
7.569
7.563
7.313
7.302
7.290
7.267
7.265
7.248
7.246
7.215
7.199
7.177
7.165
7.149
7.127
6.637
6.618
6.566
6.560
6.544
6.538
6.427
6.416
6.410
6.399
5.926
5.920
5.784
5.767
4.652
4.641
3.387
3.371
3.002
2.975
2.959
2.945
2.935
2.915
2.695
2.686
2.680
2.659
2.637
2.629
2.610
2.602
2.509
2.505
2.501
-0.000
NH
O
NO2
HN
O
N
6n
1H-NMR
1.02
1.01
1.02
1.01
2.08
5.02
1.01
1.00
1.02
1.00
1.01
1.00
3.02
1.02
3.03
13 12 11 10 9 8 7 6 5 4 3 2 1 0 ppm

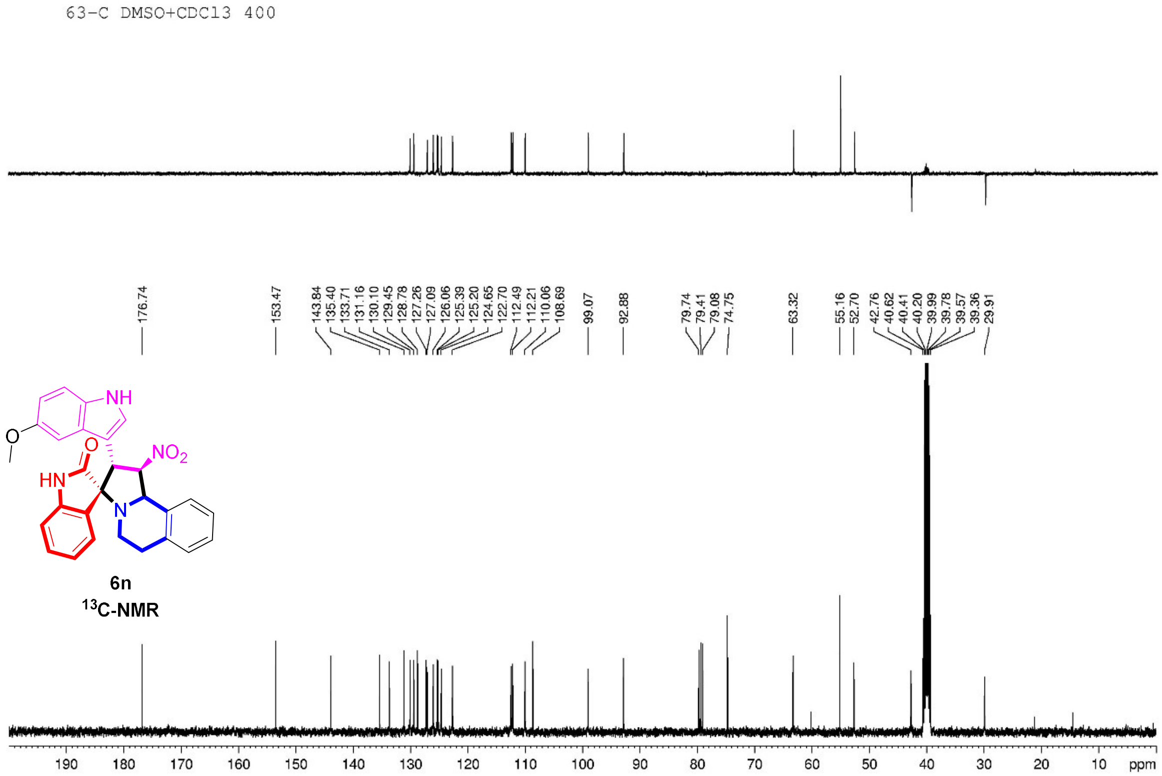
63-C DMSO+CDCl3 400
176.74
153.47
143.84
135.40
133.71
131.16
130.10
129.45
128.78
127.26
127.09
126.06
125.39
125.20
124.65
122.70
112.49
112.21
110.06
108.69
99.07
92.88
79.74
79.41
79.08
74.75
63.32
55.16
52.70
42.76
40.62
40.41
40.20
39.99
39.78
39.57
39.36
29.91
NH
O
NO2
HN
O
N
6n
13C-NMR
190 180 170 160 150 140 130 120 110 100 90 80 70 60 50 40 30 20 10 ppm

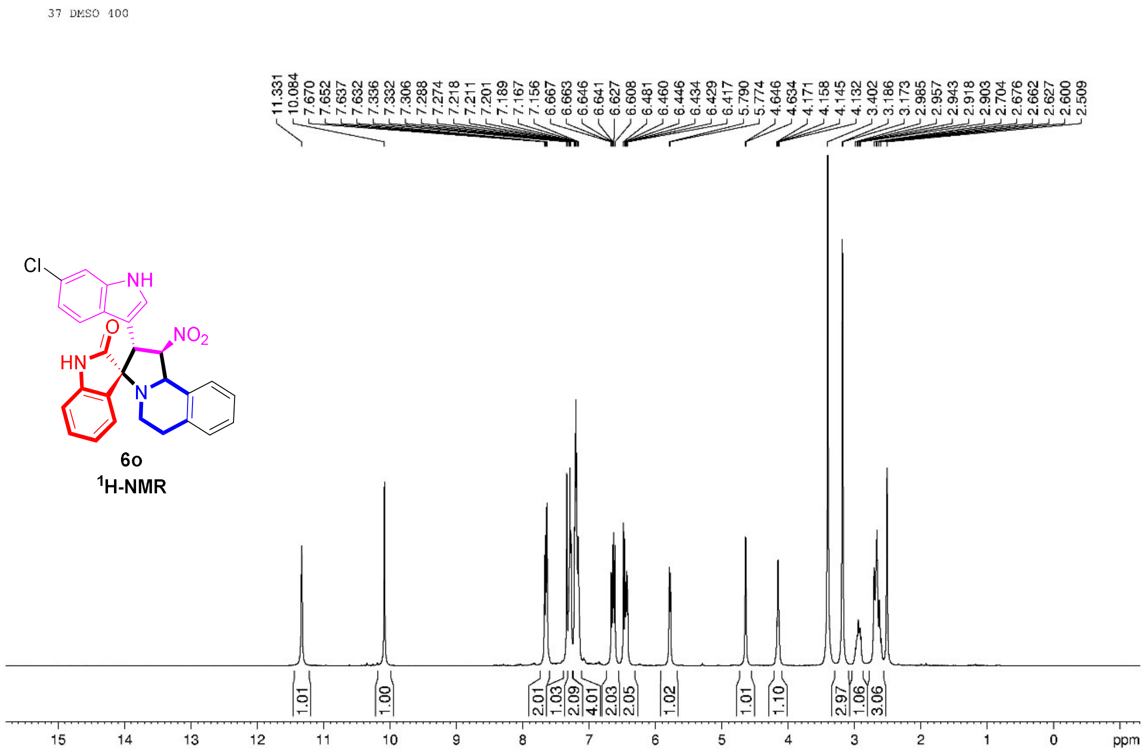
37 DMSO 400
11.331
10.084
7.670
7.652
7.637
7.632
7.336
7.332
7.306
7.288
7.274
7.218
7.211
7.201
7.189
7.167
7.156
6.667
6.663
6.646
6.641
6.627
6.608
6.481
6.460
6.446
6.434
6.429
6.417
5.790
5.774
4.646
4.634
4.171
4.158
4.145
4.132
3.402
3.186
3.173
2.985
2.957
2.943
2.918
2.903
2.704
2.676
2.662
2.627
2.600
2.509
Cl
NH
O
NO2
HN
N
6o
1H-NMR
1.01
1.00
2.01
1.03
2.09
4.01
2.03
2.05
1.02
1.01
1.10
2.97
1.06
3.06
15 14 13 12 11 10 9 8 7 6 5 4 3 2 1 0 ppm

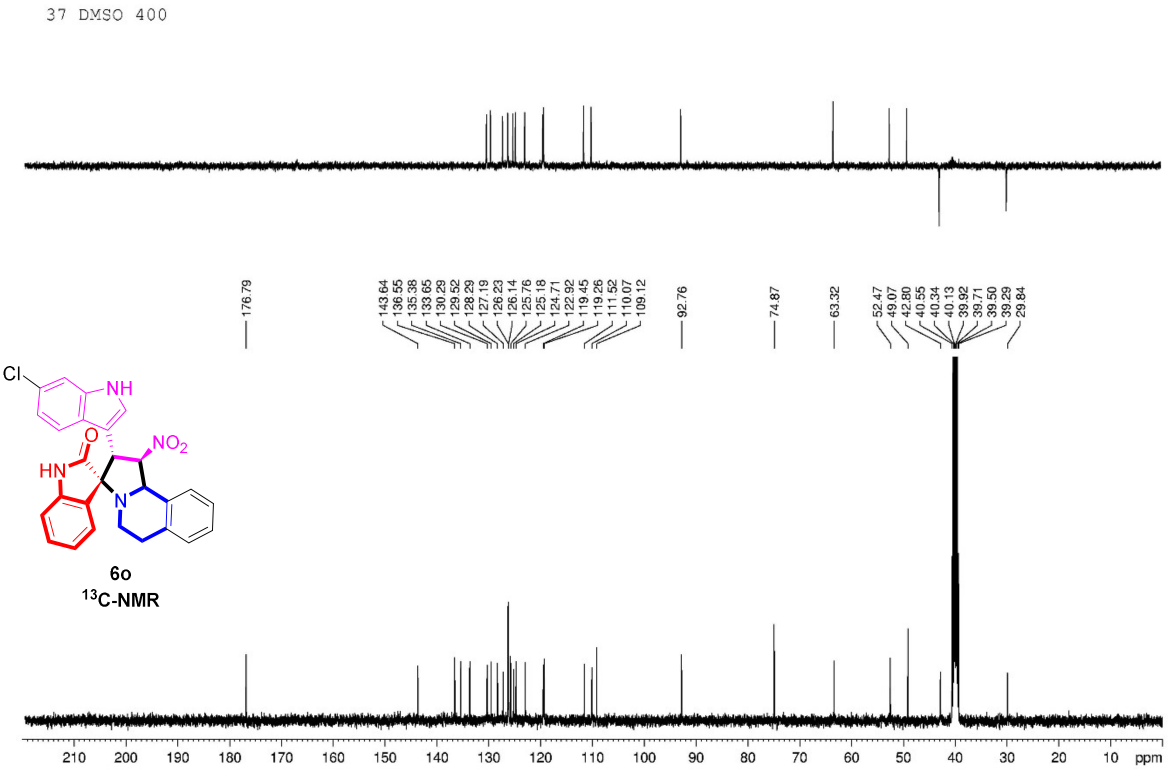
37 DMSO 400
176.79
143.64
136.55
135.38
133.65
130.29
129.52
128.29
127.19
126.23
126.14
125.76
125.18
124.71
122.92
119.45
119.26
111.52
110.07
109.12
92.76
74.87
63.32
52.47
49.07
42.80
40.55
40.34
40.13
39.92
39.71
39.50
39.29
29.84
Cl
NH
O
NO2
HN
N
6o
13C-NMR
210 200 190 180 170 160 150 140 130 120 110 100 90 80 70 60 50 40 30 20 10 ppm

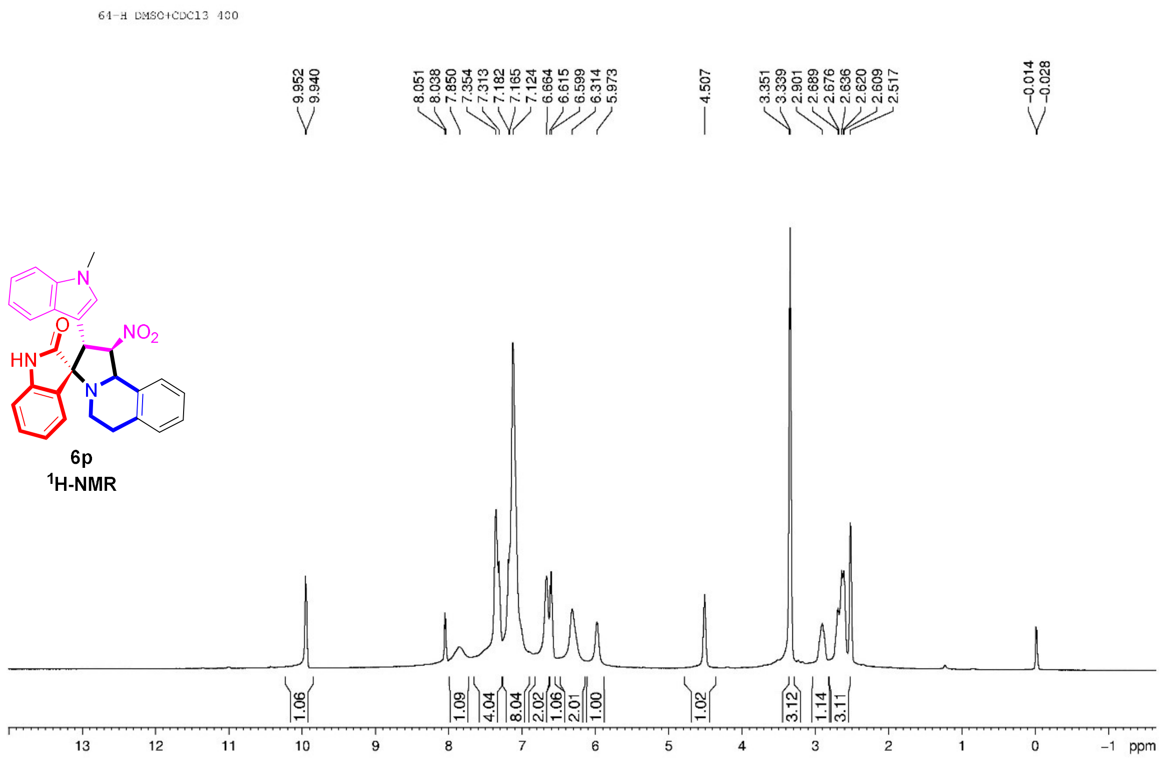
64-H DMSO+CDCl3 400
9.952
9.940
8.051
8.038
7.850
7.354
7.313
7.182
7.165
7.124
6.664
6.615
6.599
6.314
5.973
4.507
3.351
3.339
2.901
2.689
2.676
2.636
2.620
2.609
2.517
-0.014
-0.028
NO2
HN
O
N
6p
1H-NMR
1.06
1.09
4.04
8.04
2.02
1.06
2.01
1.00
1.02
3.12
1.14
3.11
ppm

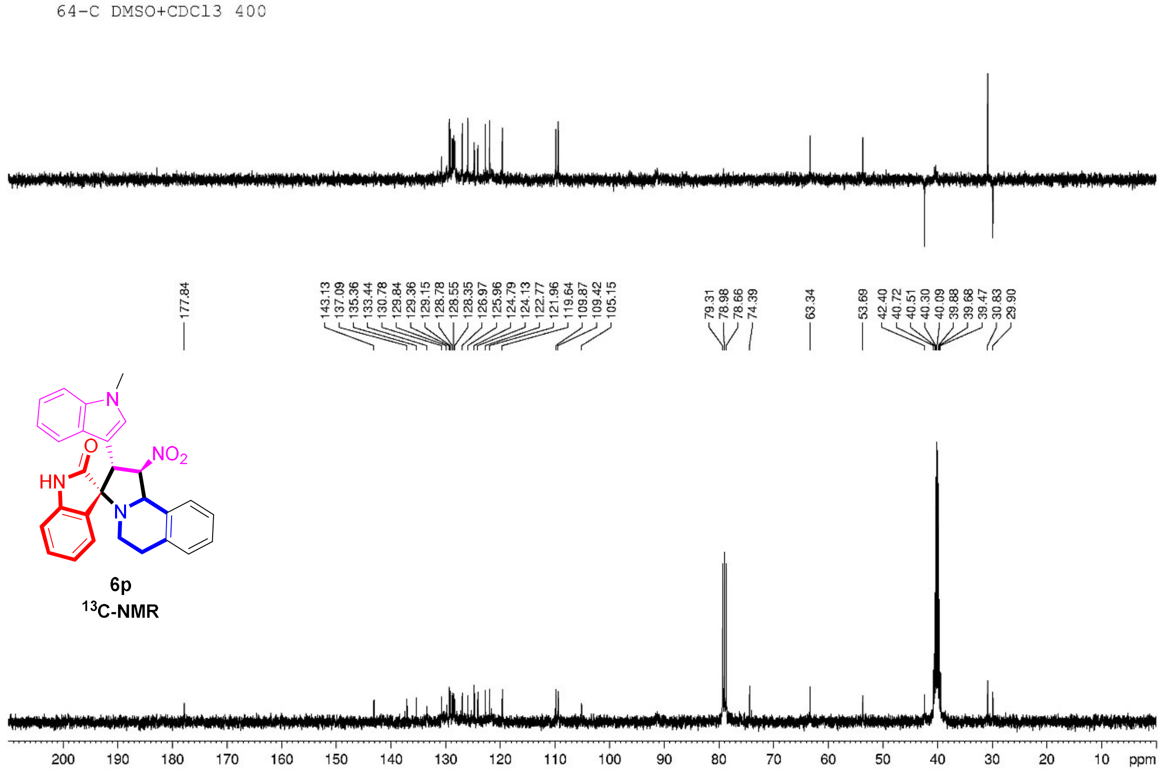
64-C DMSO+CDCl3 400
177.84
143.13
137.09
135.36
133.44
130.78
129.84
129.36
129.15
128.78
128.55
128.35
126.97
125.96
124.79
124.13
122.77
121.96
119.64
109.87
109.42
105.15
79.31
78.98
78.66
74.39
63.34
53.69
42.40
40.72
40.51
40.30
40.09
39.88
39.68
39.47
30.83
29.90
NO2
HN
O
N
6p
13C-NMR
ppm

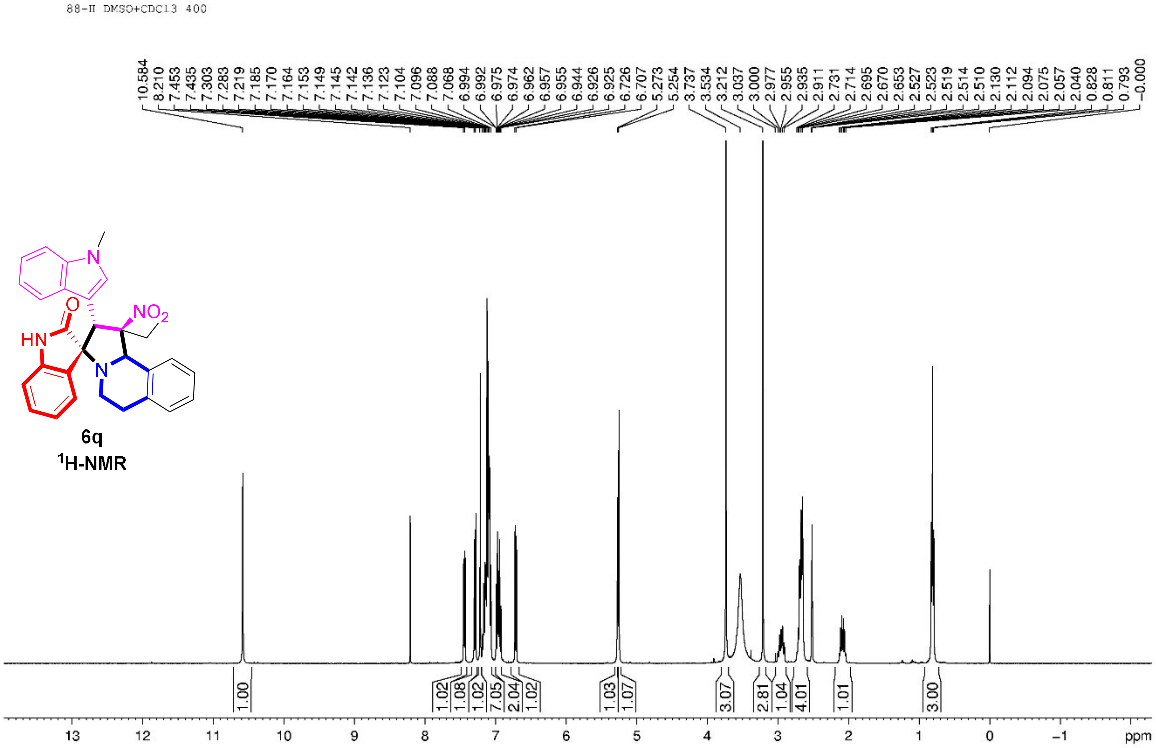
88-H DMSO+CDCl3 400
10.584 8.210 7.453 7.435 7.303 7.283 7.219 7.185 7.170 7.164 7.153 7.149 7.145 7.142 7.136 7.123 7.104 7.096 7.088 7.068 6.994 6.992 6.975 6.974 6.962 6.957 6.955 6.944 6.926 6.925 6.726 6.707 5.273 5.254 3.737 3.534 3.212 3.037 3.000 2.977 2.955 2.935 2.911 2.731 2.714 2.695 2.670 2.653 2.527 2.523 2.519 2.514 2.510 2.130 2.112 2.094 2.075 2.057 2.040 0.828 0.811 0.793 -0.000
1.00 1.02 1.08 1.02 7.05 2.04 1.02 1.03 1.07 3.07 2.81 1.04 4.01 1.01 3.00
ppm
6q
1H-NMR

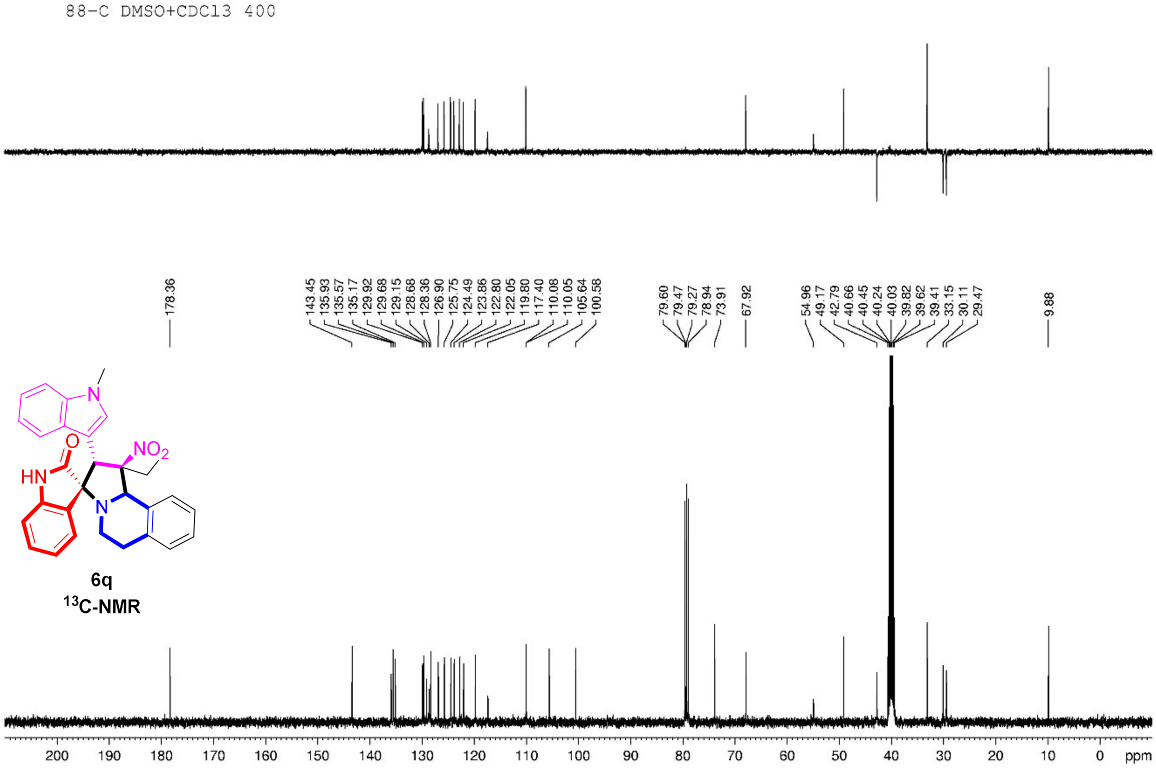
88-C DMSO+CDCl3 400
178.36
143.45 135.93 135.57 135.17 129.92 129.68 129.15 128.68 128.36 126.90 125.75 124.49 123.86 122.80 122.05 119.80 117.40 110.08 110.05 105.64 100.58
79.60 79.47 79.27 78.94 73.91 67.92
54.96 49.17 42.79 40.66 40.45 40.24 40.03 39.82 39.62 39.41 33.15 30.11 29.47
9.88
ppm
6q
13C-NMR

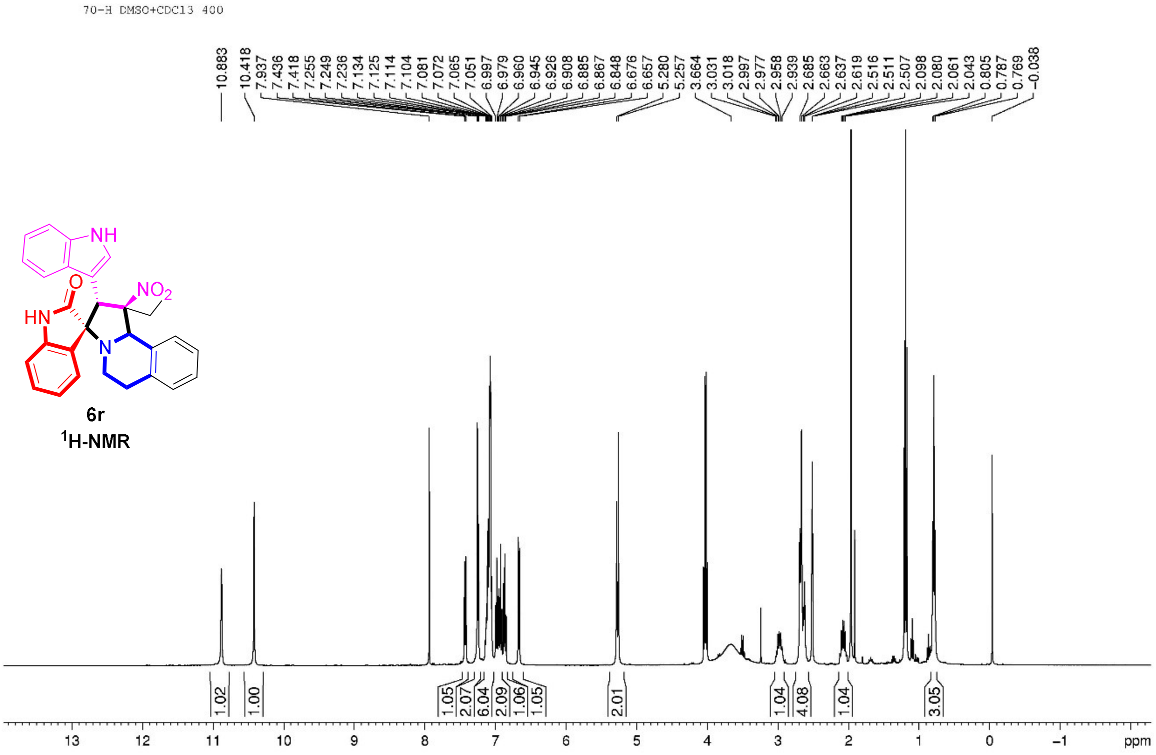
70-H DMSO+CDCl3 400
10.883
10.418
7.937
7.436
7.418
7.255
7.249
7.236
7.134
7.125
7.114
7.104
7.081
7.072
7.065
7.051
6.997
6.979
6.960
6.945
6.926
6.908
6.885
6.867
6.848
6.676
6.657
5.280
5.257
3.664
3.031
3.018
2.997
2.977
2.958
2.939
2.685
2.663
2.637
2.619
2.516
2.511
2.507
2.098
2.080
2.061
2.043
0.805
0.787
0.769
-0.038
NH
NO2
HN
O
N
6r
1H-NMR
1.02
1.00
1.05
2.07
6.04
2.09
1.06
1.05
2.01
1.04
4.08
1.04
3.05
13
12
11
10
9
8
7
6
5
4
3
2
1
0
-1
ppm

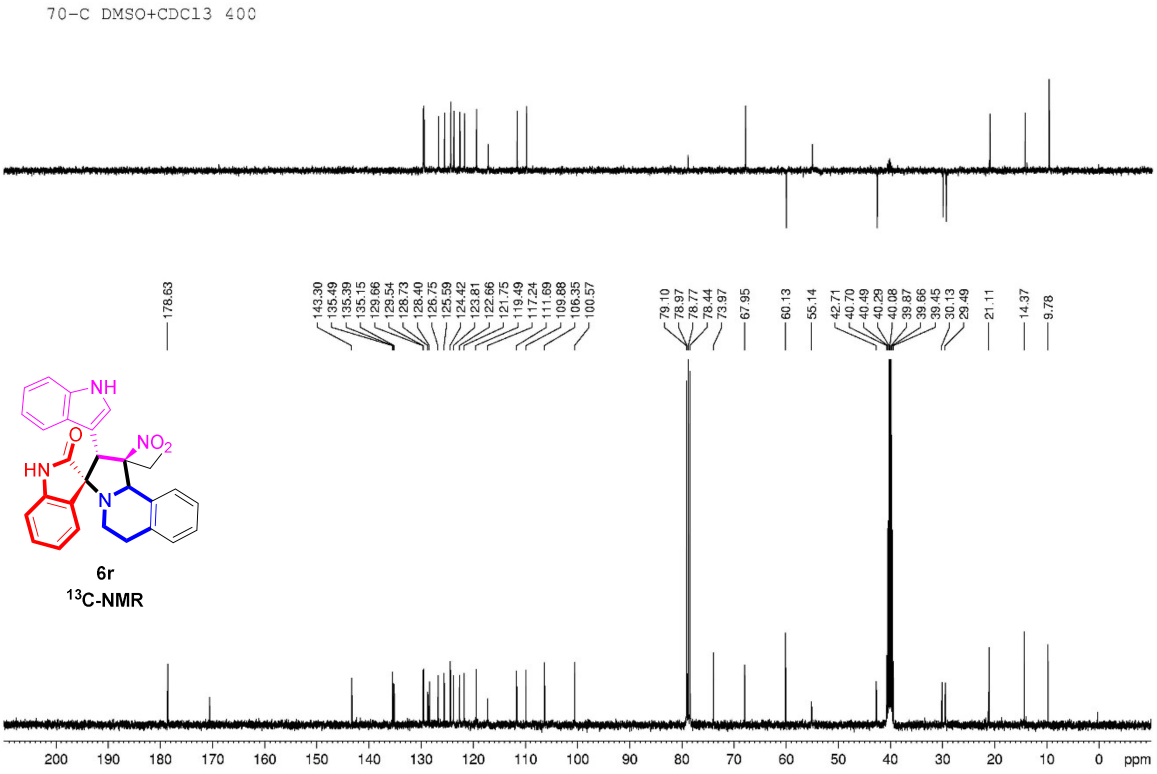
70-C DMSO+CDCl3 400
178.63
143.30
135.49
135.39
135.15
129.66
129.54
128.73
128.40
126.75
125.59
124.42
123.81
122.66
121.75
119.49
117.24
111.69
109.88
106.35
100.57
79.10
78.97
78.77
78.44
73.97
67.95
60.13
55.14
42.71
40.70
40.49
40.29
40.08
39.87
39.66
39.45
30.13
29.49
21.11
14.37
9.78
NH
NO2
HN
O
N
6r
13C-NMR
200
190
180
170
160
150
140
130
120
110
100
90
80
70
60
50
40
30
20
10
0
ppm

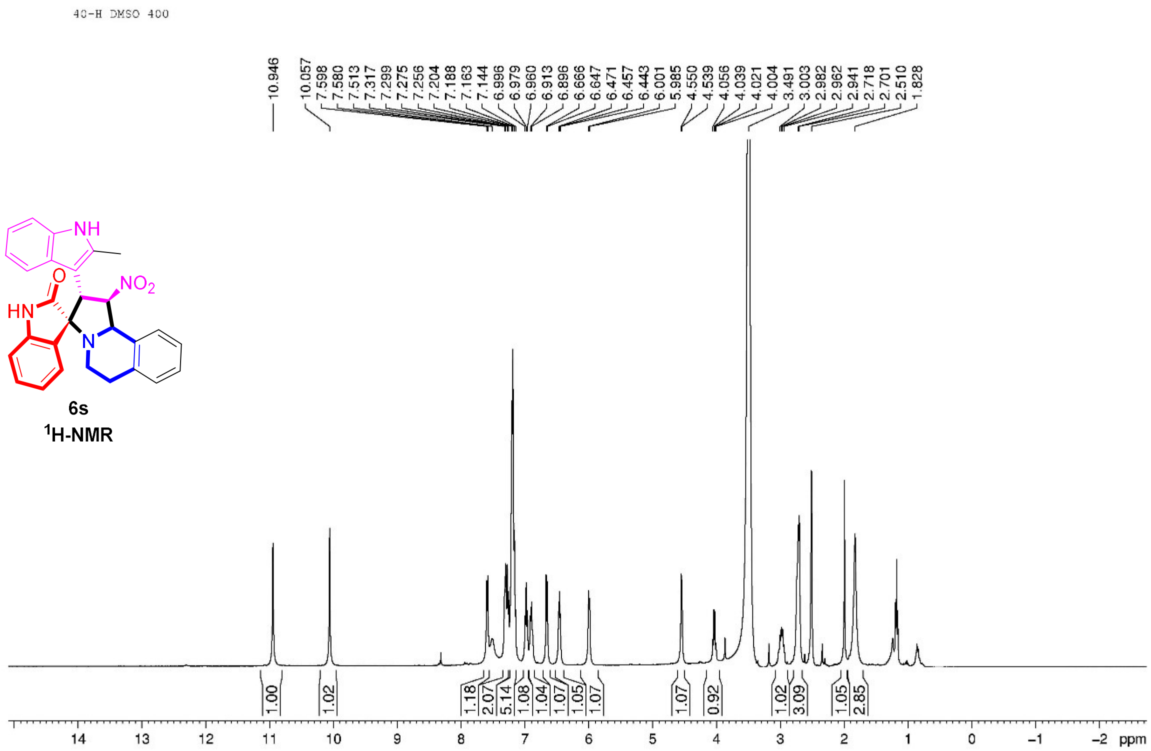
40-H DMSO 400
10.946
10.057
7.598
7.580
7.513
7.317
7.299
7.275
7.256
7.204
7.188
7.163
7.144
6.996
6.979
6.960
6.913
6.896
6.666
6.647
6.471
6.457
6.443
6.001
5.985
4.550
4.539
4.056
4.039
4.021
4.004
3.491
3.003
2.982
2.962
2.941
2.718
2.701
2.510
1.828
NH
O
NO2
HN
N
6s
1H-NMR
1.00
1.02
1.18
2.07
5.14
1.08
1.04
1.07
1.05
1.07
1.07
0.92
1.02
3.09
1.05
2.85
14 13 12 11 10 9 8 7 6 5 4 3 2 1 0 -1 -2 ppm

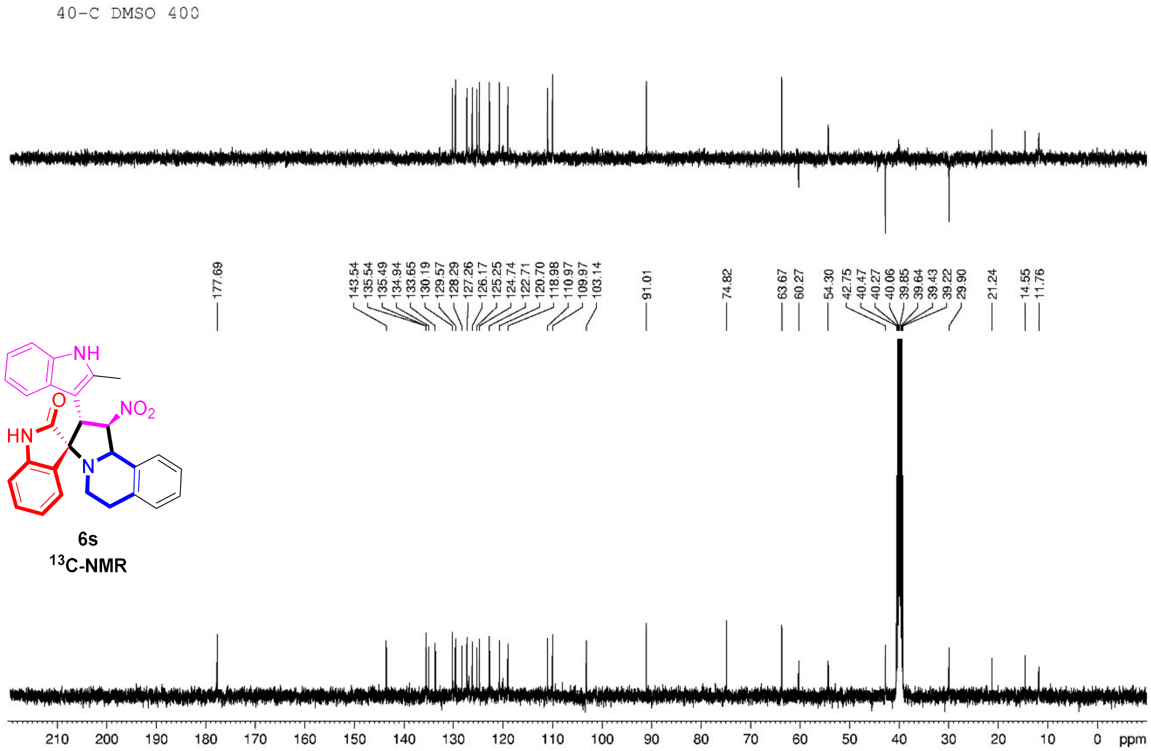
40-C DMSO 400
177.69
143.54
135.54
135.49
134.94
133.65
130.19
129.57
128.29
127.26
126.17
125.25
124.74
122.71
120.70
118.98
110.97
109.97
103.14
91.01
74.82
63.67
60.27
54.30
42.75
40.47
40.27
40.06
39.85
39.64
39.43
39.22
29.90
21.24
14.55
11.76
NH
O
NO2
HN
N
6s
13C-NMR
210 200 190 180 170 160 150 140 130 120 110 100 90 80 70 60 50 40 30 20 10 0 ppm

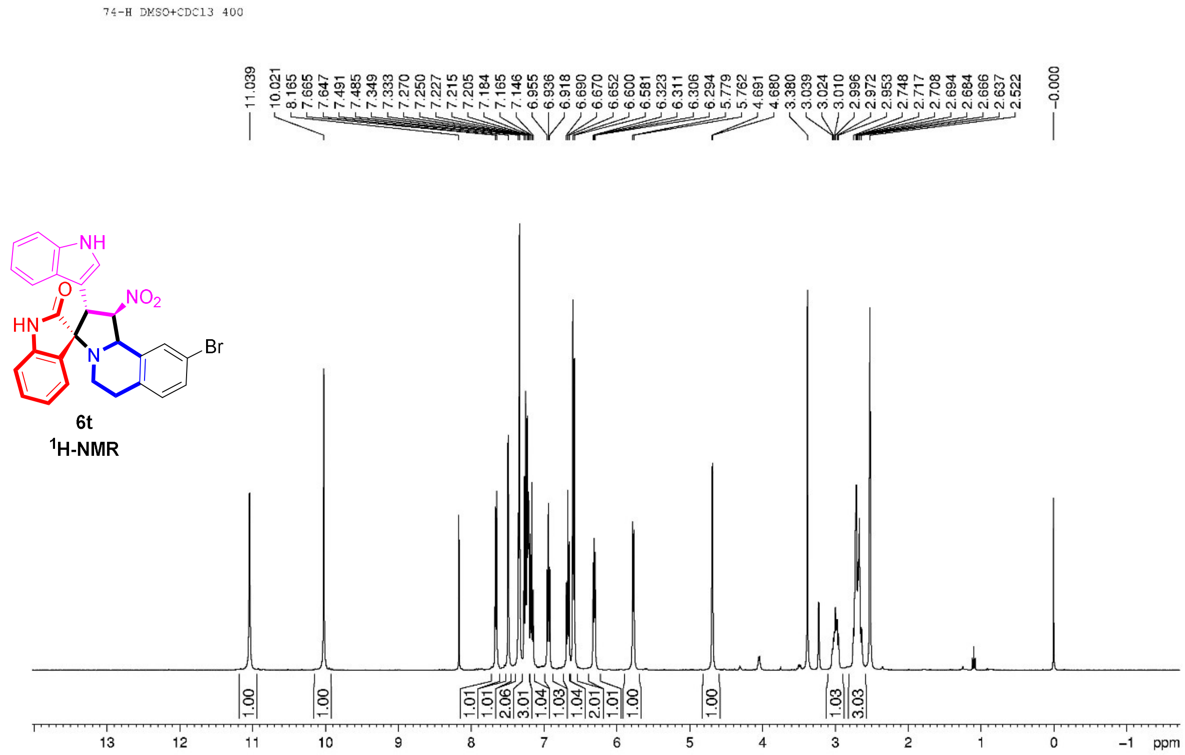

74-H DMSO+CDCl3 400
11.039
10.021
8.165
7.665
7.647
7.491
7.485
7.349
7.333
7.270
7.250
7.227
7.215
7.205
7.184
7.165
7.146
6.955
6.936
6.918
6.690
6.670
6.652
6.600
6.581
6.323
6.311
6.306
6.294
5.779
5.762
4.691
4.680
3.380
3.039
3.024
3.010
2.996
2.972
2.953
2.748
2.717
2.708
2.694
2.684
2.666
2.637
2.522
-0.000
NH
O
NO2
HN
N
Br
6t
1H-NMR
1.00
1.00
1.01
1.01
2.06
3.01
1.04
1.03
1.04
2.01
1.01
1.00
1.00
1.03
3.03
13
12
11
10
9
8
7
6
5
4
3
2
1
0
-1
ppm

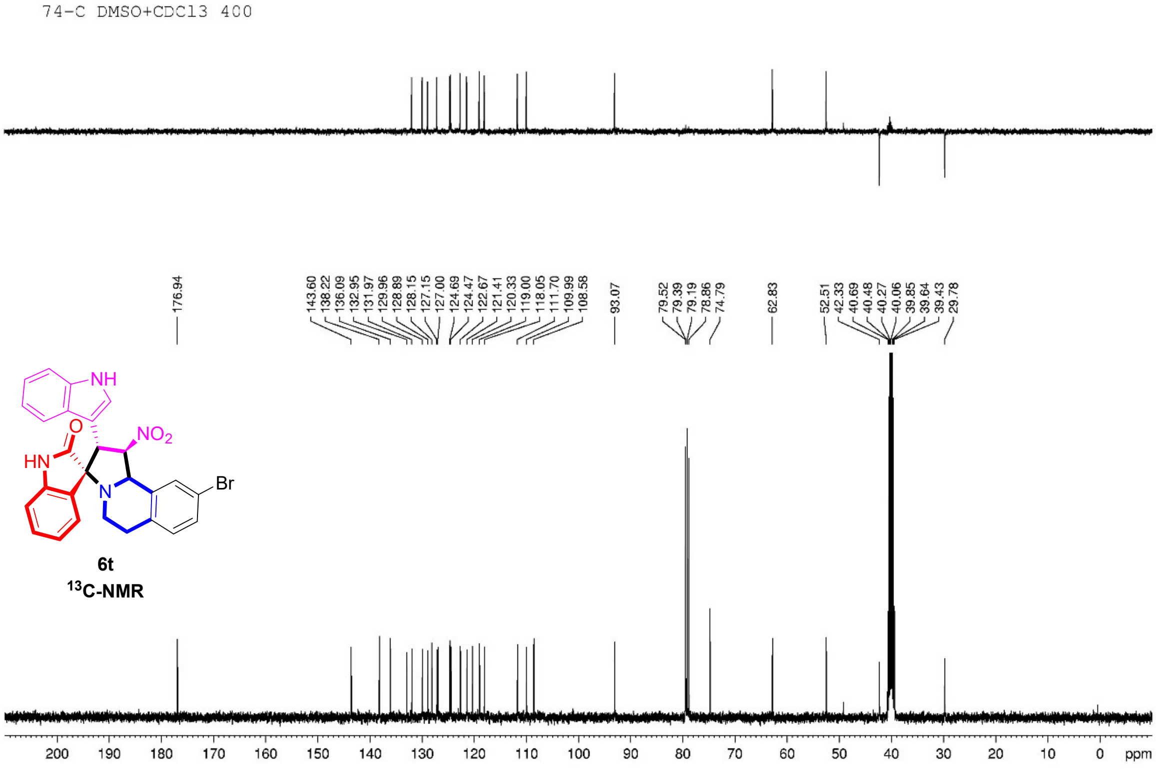

74-C DMSO+CDCl3 400
176.94
143.60
138.22
136.09
132.95
131.97
129.96
128.89
128.15
127.15
127.00
124.69
124.47
122.67
121.41
120.33
119.00
118.05
111.70
109.99
108.58
93.07
79.52
79.39
79.19
78.86
74.79
62.83
52.51
42.33
40.69
40.48
40.27
40.06
39.85
39.64
39.43
29.78
NH
O
NO2
HN
N
Br
6t
13C-NMR
200
190
180
170
160
150
140
130
120
110
100
90
80
70
60
50
40
30
20
10
0
ppm

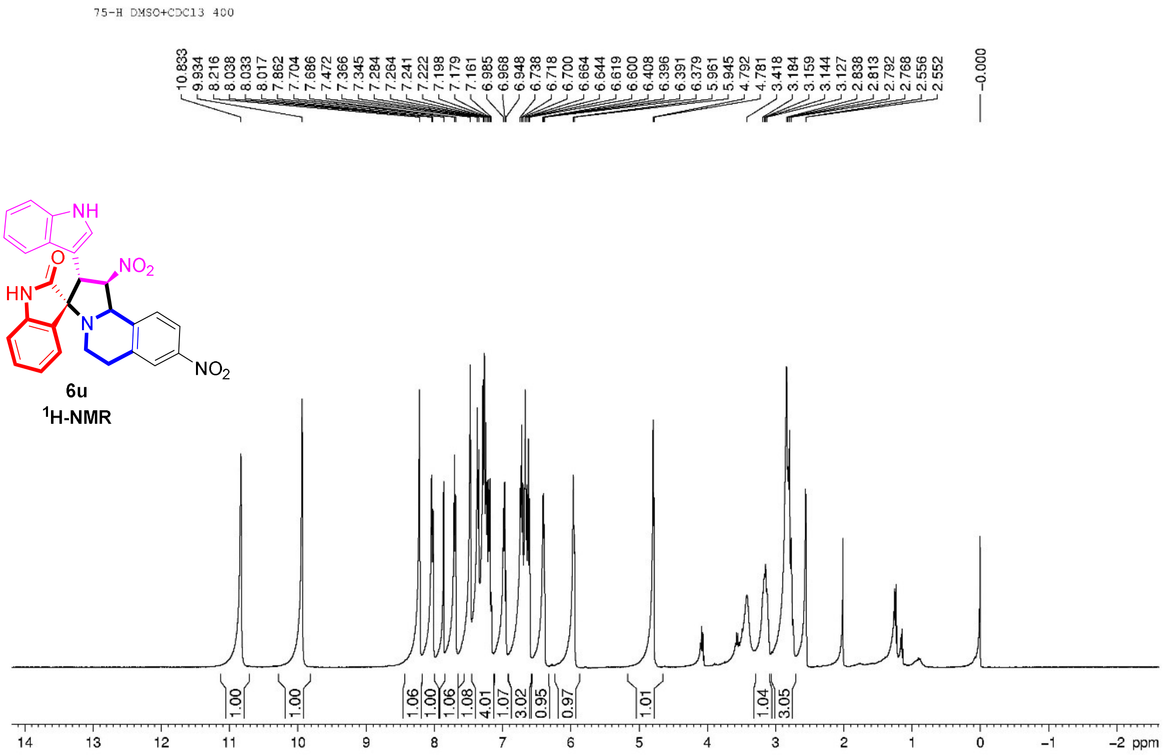
75-H DMSO+CDCl3 400
10.833
9.934
8.216
8.038
8.033
8.017
7.862
7.704
7.686
7.472
7.366
7.345
7.284
7.264
7.241
7.222
7.198
7.179
7.161
6.985
6.968
6.948
6.738
6.718
6.700
6.664
6.644
6.619
6.600
6.408
6.396
6.391
6.379
5.961
5.945
4.792
4.781
3.418
3.184
3.159
3.144
3.127
2.838
2.813
2.792
2.768
2.556
2.552
0.000
NH
NO2
O
HN
N
NO2
6u
1H-NMR
1.00
1.00
1.06
1.00
1.06
1.08
4.01
1.07
3.02
0.95
0.97
1.01
1.04
3.05
14
13
12
11
10
9
8
7
6
5
4
3
2
1
0
−1
−2 ppm

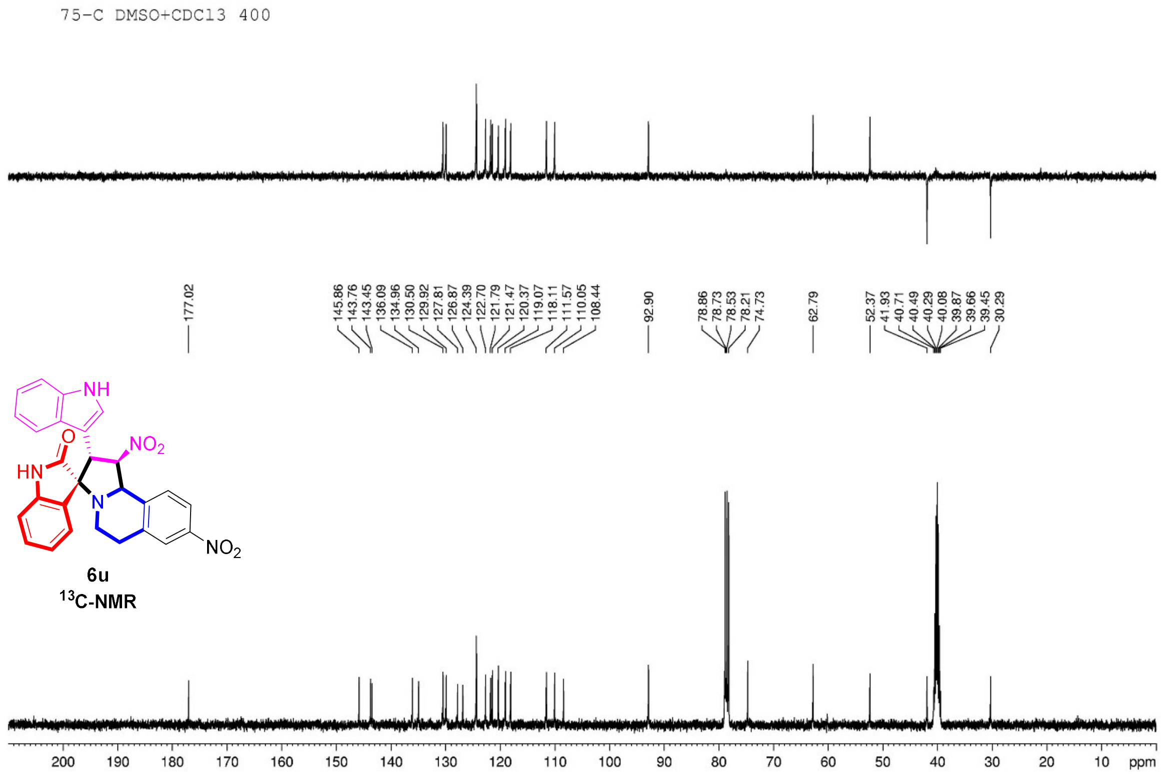
75-C DMSO+CDCl3 400
177.02
145.86
143.76
143.45
136.09
134.96
130.50
129.92
127.81
126.87
124.39
122.70
121.79
121.47
120.37
119.07
118.11
111.57
110.05
108.44
92.90
78.86
78.73
78.53
78.21
74.73
62.79
52.37
41.93
40.71
40.49
40.29
40.08
39.87
39.66
39.45
30.29
NH
O
NO2
HN
N
NO2
6u
13C-NMR
200
190
180
170
160
150
140
130
120
110
100
90
80
70
60
50
40
30
20
10
ppm

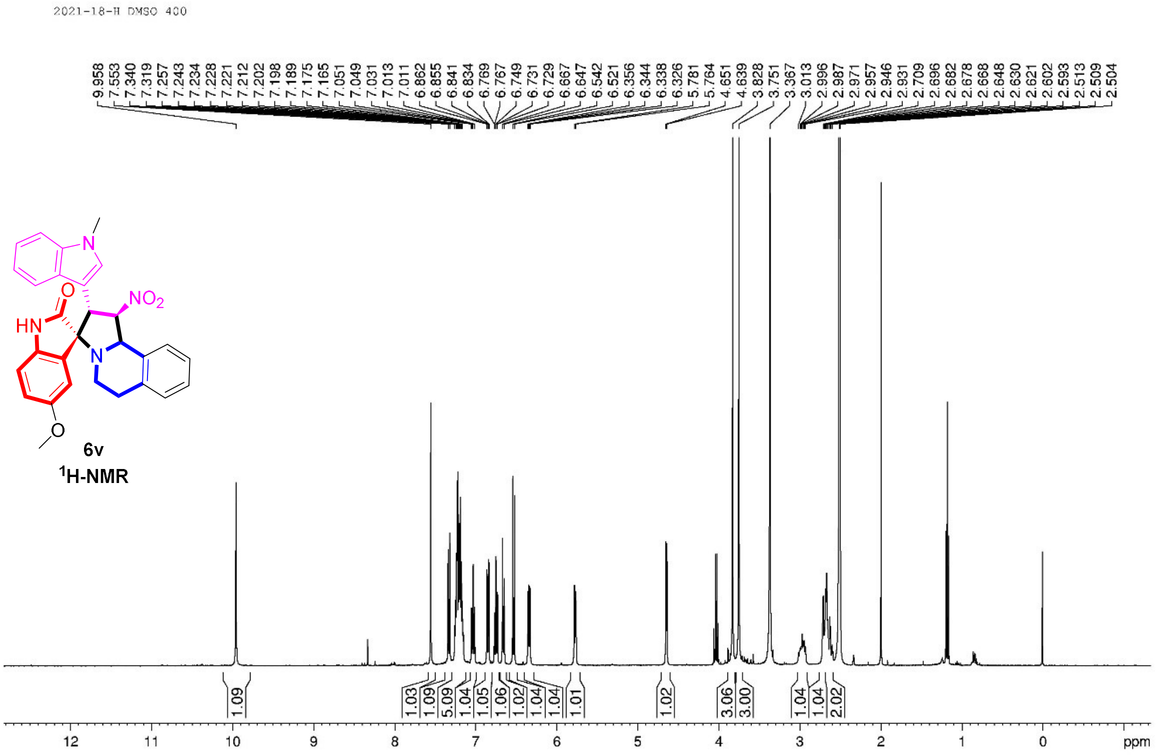
2021-18-II DMSO 400
9.958
7.553
7.340
7.319
7.257
7.243
7.234
7.228
7.221
7.212
7.202
7.198
7.189
7.175
7.165
7.051
7.049
7.031
7.013
7.011
6.862
6.855
6.841
6.834
6.769
6.767
6.749
6.731
6.729
6.667
6.647
6.542
6.521
6.356
6.344
6.338
6.326
5.781
5.764
4.651
4.639
3.828
3.751
3.367
3.013
2.996
2.987
2.971
2.957
2.946
2.931
2.709
2.696
2.682
2.678
2.668
2.648
2.630
2.621
2.602
2.593
2.513
2.509
2.504
NO2
HN
O
N
N
O
6v
1H-NMR
1.09
1.03
1.09
5.09
1.04
1.05
1.06
1.02
1.04
1.04
1.01
1.02
3.06
3.00
1.04
1.04
2.02
12
11
10
9
8
7
6
5
4
3
2
1
0
ppm

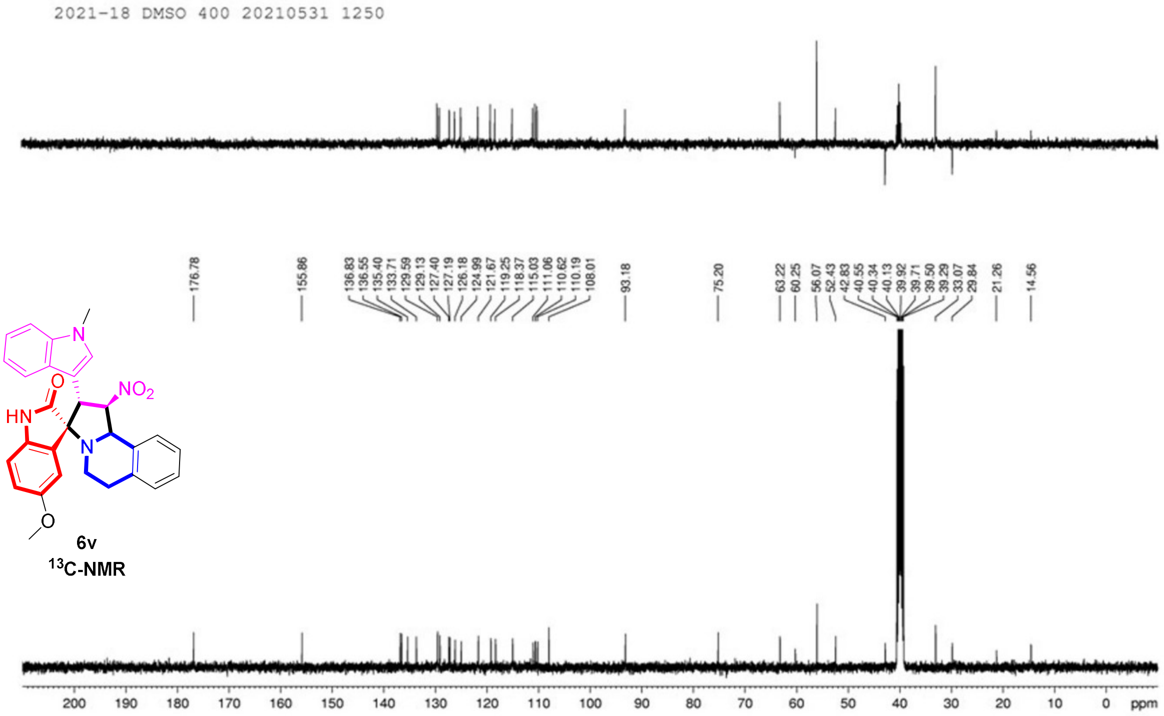
2021-18 DMSO 400 20210531 1250
176.78
155.86
136.83
136.55
135.40
133.71
129.59
129.13
127.40
127.19
126.18
124.99
121.67
119.25
118.37
115.03
111.06
110.62
110.19
108.01
93.18
75.20
63.22
60.25
56.07
52.43
42.83
40.55
40.34
40.13
39.92
39.71
39.50
39.29
33.07
29.84
21.26
14.56
NO2
HN
O
N
N
O
6v
13C-NMR
200
190
180
170
160
150
140
130
120
110
100
90
80
70
60
50
40
30
20
10
0
ppm

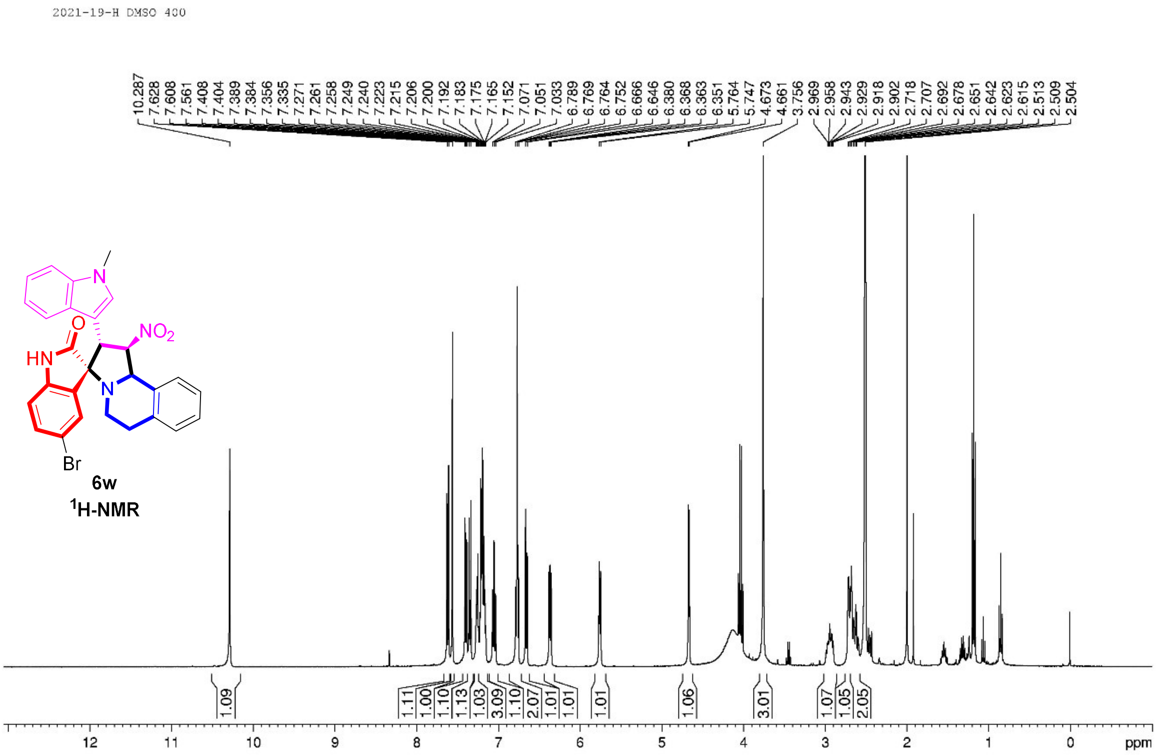
2021-19-H DMSO 400
10.287 7.628 7.608 7.561 7.408 7.404 7.389 7.384 7.356 7.335 7.271 7.261 7.258 7.249 7.240 7.223 7.215 7.206 7.200 7.192 7.183 7.175 7.165 7.152 7.071 7.051 7.033 6.789 6.769 6.764 6.752 6.666 6.646 6.380 6.368 6.363 6.351 5.764 5.747 4.673 4.661 3.756 2.969 2.958 2.943 2.929 2.918 2.902 2.718 2.707 2.692 2.678 2.651 2.642 2.623 2.615 2.513 2.509 2.504
N
O
NO_2
HN
N
Br
6w
^{1}H-NMR
1.09 1.11 1.00 1.10 1.13 1.03 3.09 1.10 2.07 1.01 1.01 1.01 1.06 3.01 1.07 1.05 2.05
12 11 10 9 8 7 6 5 4 3 2 1 0 ppm

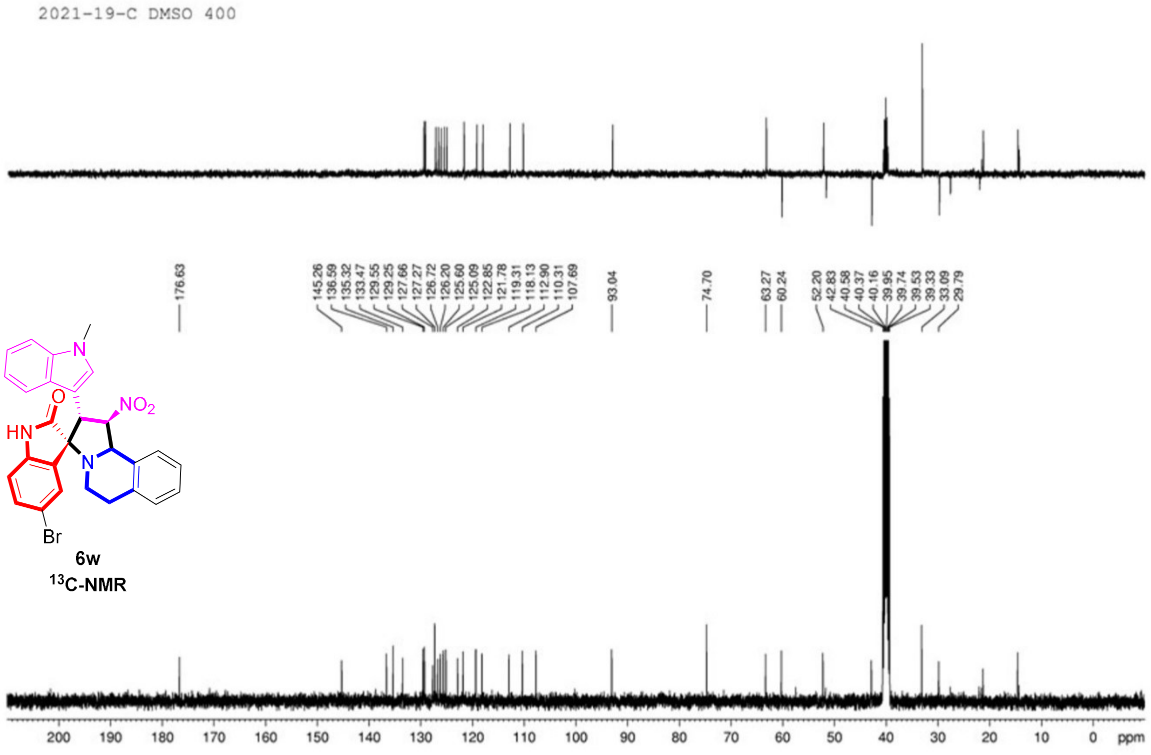
2021-19-C DMSO 400
176.63
145.26 136.59 135.32 133.47 129.55 129.25 127.66 127.27 126.72 126.20 125.60 125.09 122.85 121.78 119.31 118.13 112.90 110.31 107.69
93.04
74.70
63.27 60.24
52.20 42.83 40.58 40.37 40.16 39.95 39.74 39.53 39.33 33.09 29.79
N
O
NO_2
HN
N
Br
6w
^{13}C-NMR
200 190 180 170 160 150 140 130 120 110 100 90 80 70 60 50 40 30 20 10 0 ppm

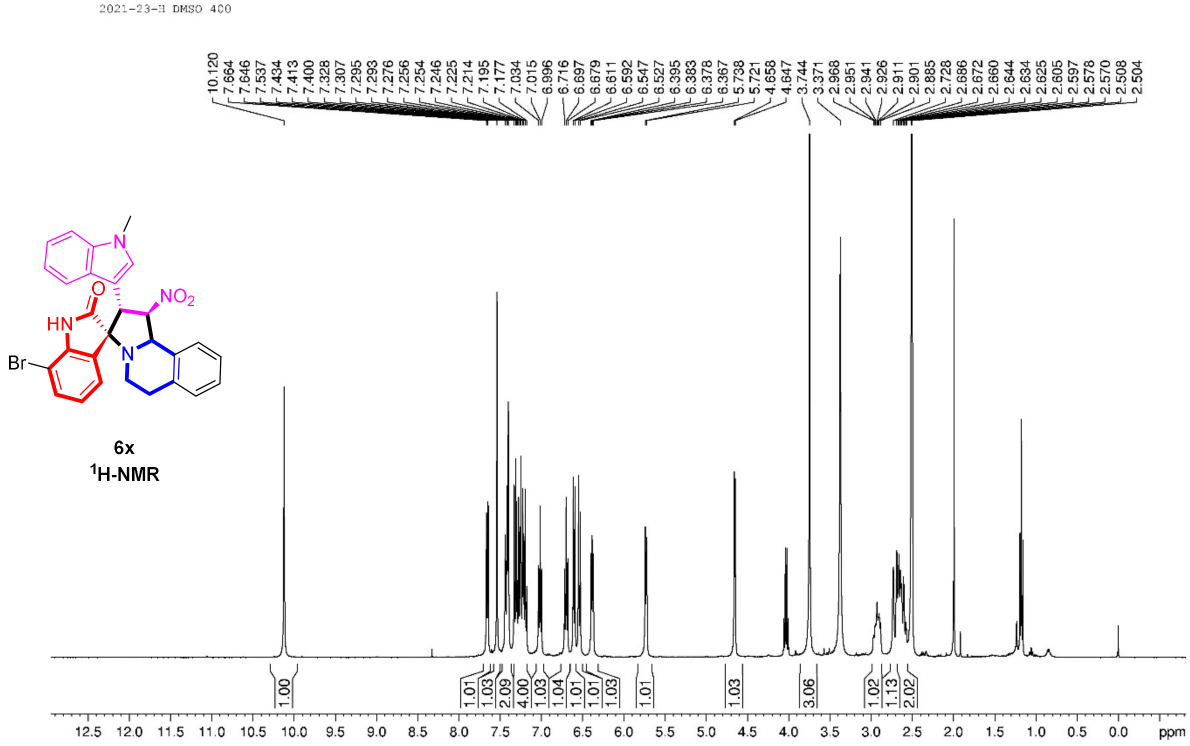
2021-23-H DMSO 400
10.120
7.664
7.646
7.537
7.434
7.413
7.400
7.328
7.307
7.295
7.293
7.276
7.256
7.254
7.246
7.225
7.214
7.195
7.177
7.034
7.015
6.996
6.716
6.697
6.679
6.611
6.592
6.547
6.527
6.395
6.383
6.378
6.367
5.738
5.721
4.658
4.647
3.744
3.371
2.968
2.951
2.941
2.926
2.911
2.901
2.885
2.728
2.686
2.672
2.660
2.644
2.634
2.625
2.605
2.597
2.578
2.570
2.508
2.504
HN
O
N
NO2
N
Br
6x
1H-NMR
1.00
1.01
1.03
2.09
4.00
1.03
1.04
1.01
1.01
1.03
1.01
1.03
3.06
1.02
1.13
2.02
12.5 12.0 11.5 11.0 10.5 10.0 9.5 9.0 8.5 8.0 7.5 7.0 6.5 6.0 5.5 5.0 4.5 4.0 3.5 3.0 2.5 2.0 1.5 1.0 0.5 0.0 ppm

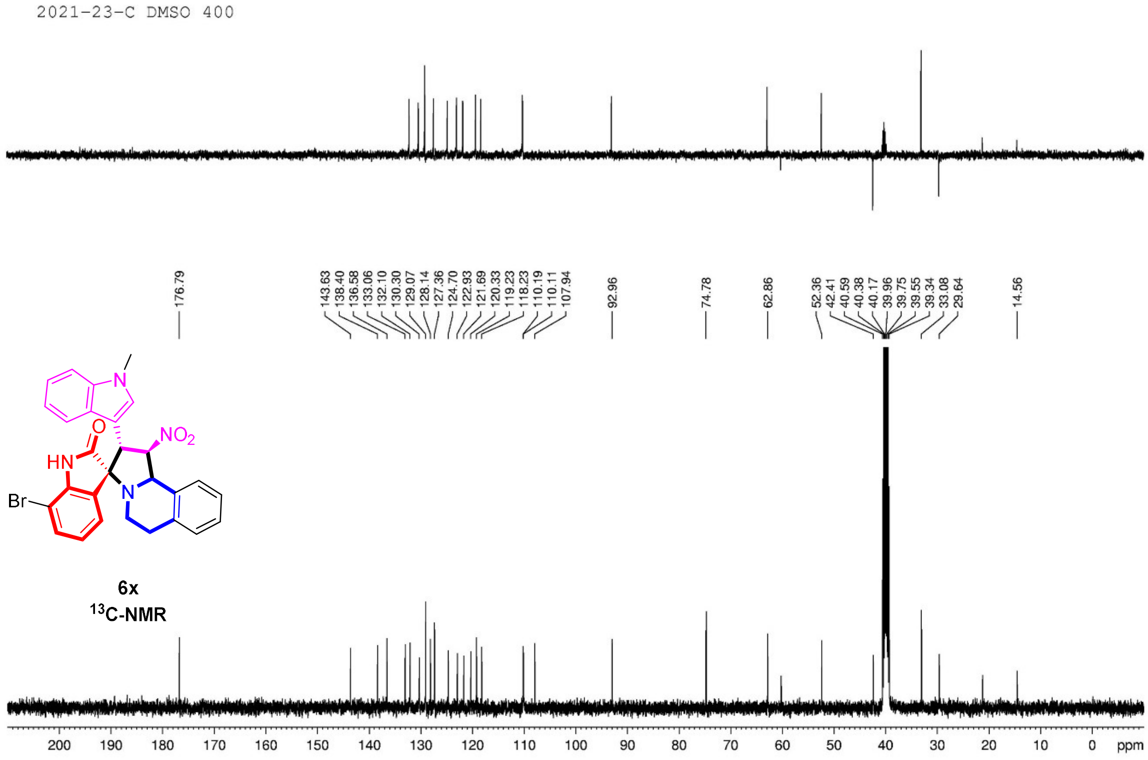
2021-23-C DMSO 400
176.79
143.63
138.40
136.58
133.06
132.10
130.30
129.07
128.14
127.36
124.70
122.93
121.69
120.33
119.23
118.23
110.19
110.11
107.94
92.96
74.78
62.86
52.36
42.41
40.59
40.38
40.17
39.96
39.75
39.55
39.34
33.08
29.64
14.56
HN
O
N
NO2
N
Br
6x
13C-NMR
200 190 180 170 160 150 140 130 120 110 100 90 80 70 60 50 40 30 20 10 0 ppm

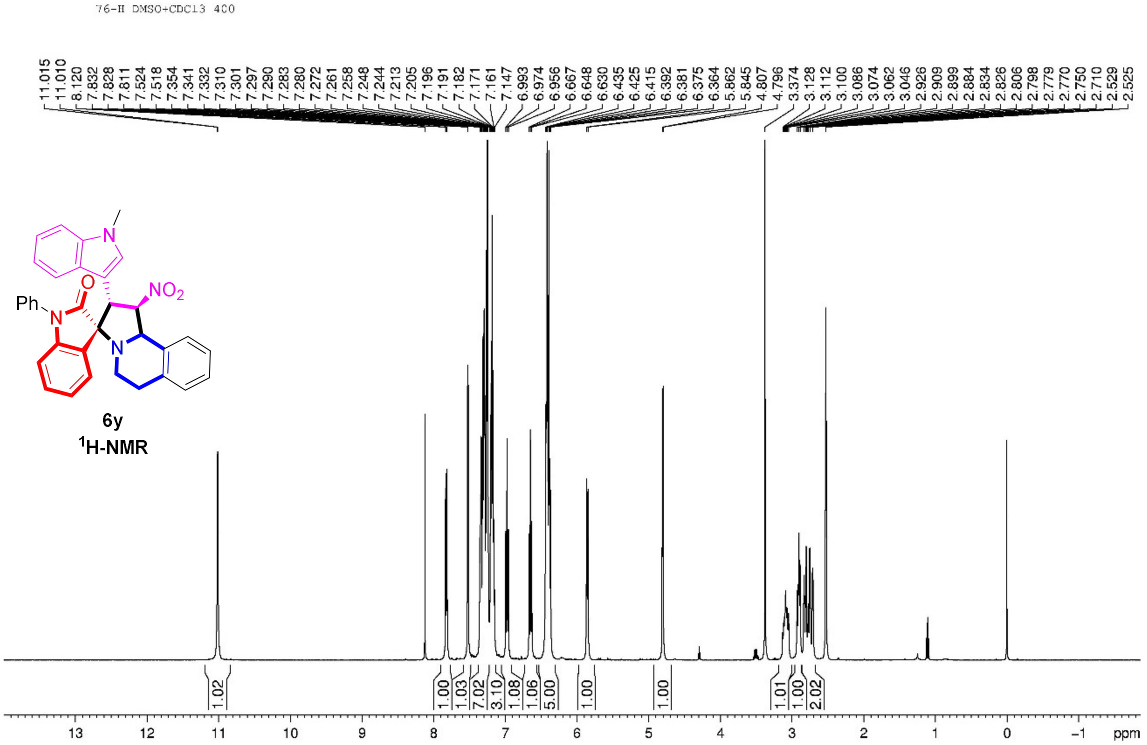
76-H DMSO+CDCl3 400
11.015
11.010
8.120
7.832
7.828
7.811
7.524
7.518
7.354
7.341
7.332
7.310
7.301
7.297
7.290
7.283
7.280
7.272
7.261
7.258
7.248
7.244
7.213
7.205
7.196
7.191
7.182
7.171
7.161
7.147
6.993
6.974
6.956
6.667
6.648
6.630
6.435
6.425
6.415
6.392
6.381
6.375
6.364
5.862
5.845
4.807
4.796
3.374
3.128
3.112
3.100
3.086
3.074
3.062
3.046
2.926
2.909
2.899
2.884
2.834
2.826
2.806
2.798
2.779
2.770
2.750
2.710
2.529
2.525
Ph
N
O
N
NO2
N
6y
1H-NMR
1.02
1.00
1.03
7.02
3.10
1.08
1.06
5.00
1.00
1.00
1.01
1.00
2.02
13
12
11
10
9
8
7
6
5
4
3
2
1
0
−1
ppm

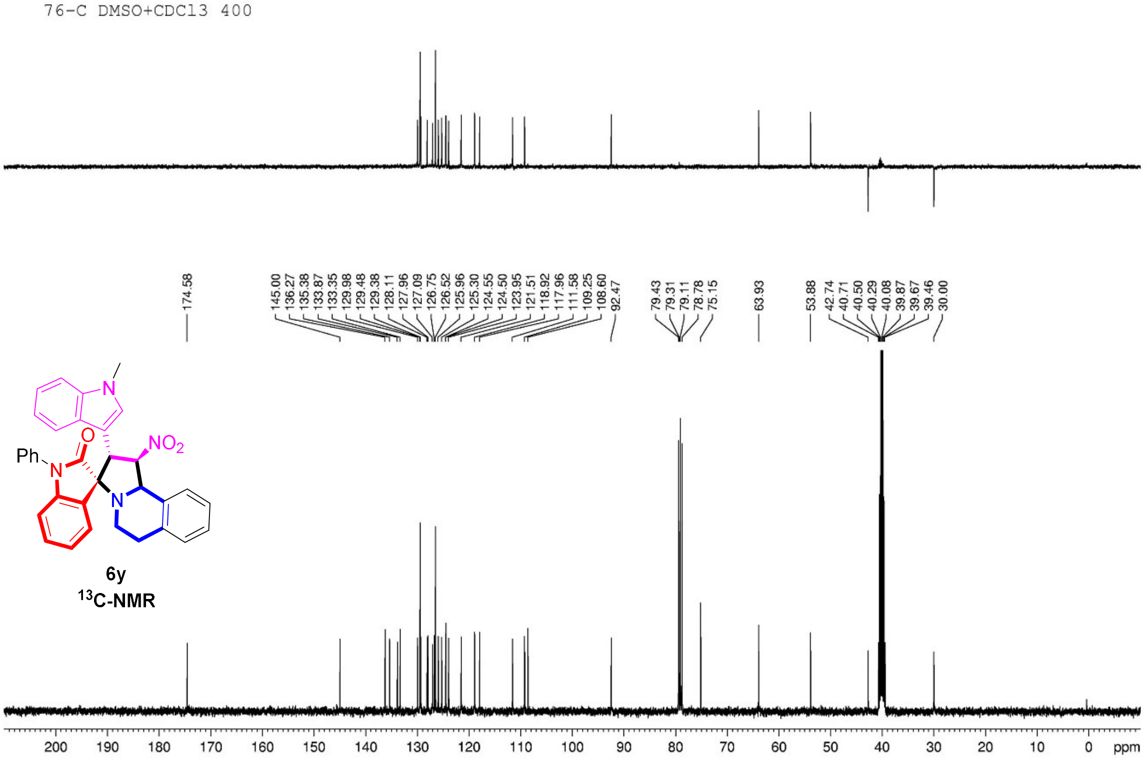
76-C DMSO+CDCl3 400
174.58
145.00
136.27
135.38
133.87
133.35
129.98
129.48
129.38
128.11
127.96
127.09
126.75
126.52
125.96
125.30
124.55
124.50
123.95
121.51
118.92
117.96
111.58
109.25
108.60
92.47
79.43
79.31
79.11
78.78
75.15
63.93
53.88
42.74
40.71
40.50
40.29
40.08
39.87
39.67
39.46
30.00
Ph
N
O
N
NO2
N
6y
13C-NMR
200
190
180
170
160
150
140
130
120
110
100
90
80
70
60
50
40
30
20
10
0
ppm

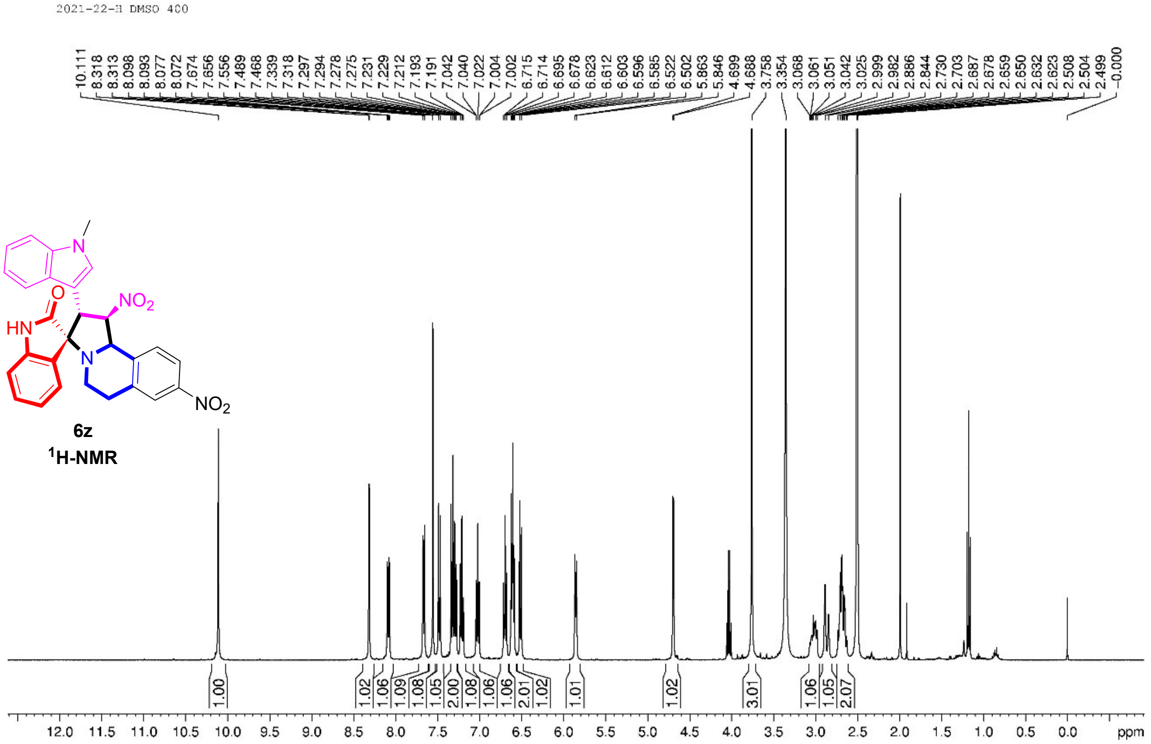
2021-22-H DMSO 400
10.111 8.318 8.313 8.098 8.093 8.077 8.072 7.674 7.656 7.556 7.489 7.468 7.339 7.318 7.297 7.294 7.278 7.275 7.231 7.229 7.212 7.193 7.191 7.042 7.040 7.022 7.004 7.002 6.715 6.714 6.695 6.678 6.623 6.612 6.603 6.596 6.585 6.522 6.502 5.863 5.846 4.699 4.688 3.758 3.354 3.068 3.061 3.051 3.042 3.025 2.999 2.982 2.886 2.844 2.730 2.703 2.687 2.678 2.659 2.650 2.632 2.623 2.508 2.504 2.499 -0.000
NO2
HN
O
N
NO2
6z
1H-NMR
1.00 1.02 1.06 1.09 1.08 1.05 2.00 1.08 1.06 1.06 2.01 1.02 1.01 1.02 3.01 1.06 1.05 2.07
12.0 11.5 11.0 10.5 10.0 9.5 9.0 8.5 8.0 7.5 7.0 6.5 6.0 5.5 5.0 4.5 4.0 3.5 3.0 2.5 2.0 1.5 1.0 0.5 0.0 ppm

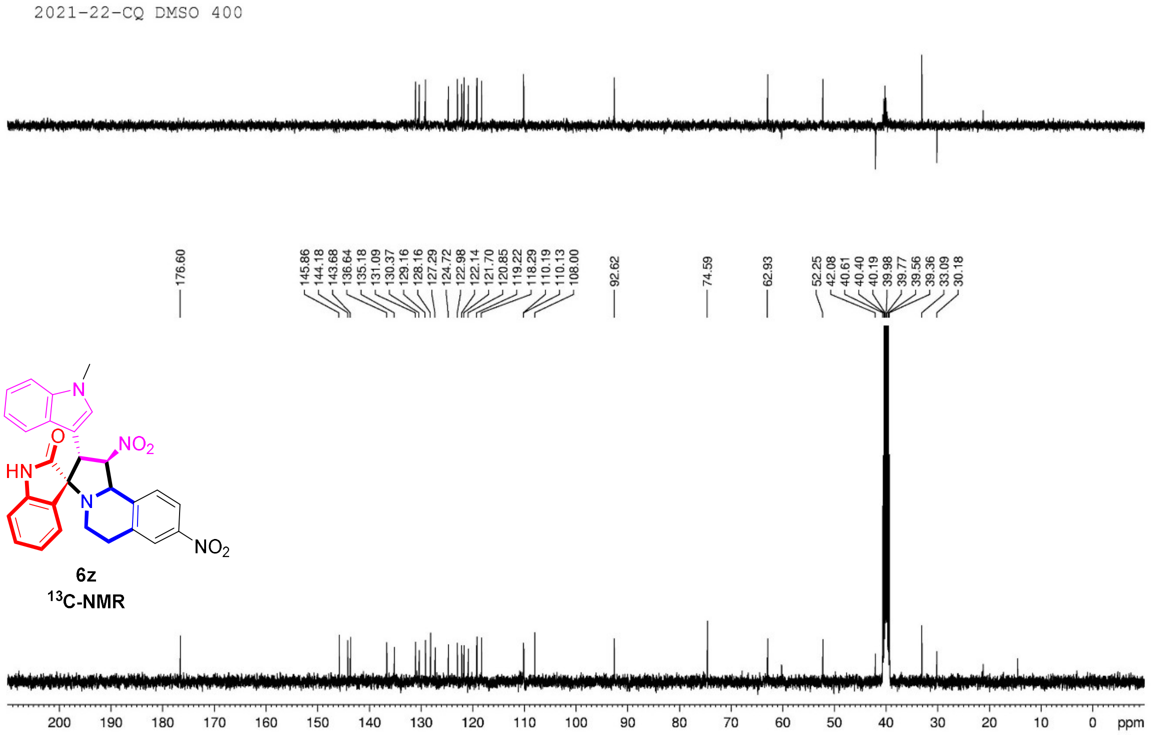
2021-22-CQ DMSO 400
176.60
145.86 144.18 143.68 136.64 135.18 131.09 130.37 129.16 128.16 127.29 124.72 122.98 122.14 121.70 120.85 119.22 118.29 110.19 110.13 108.00
92.62
74.59
62.93
52.25 42.08 40.61 40.40 40.19 39.98 39.77 39.56 39.36 33.09 30.18
NO2
HN
O
N
NO2
6z
13C-NMR
200 190 180 170 160 150 140 130 120 110 100 90 80 70 60 50 40 30 20 10 0 ppm

3. ^{1}H and ^{13}C NMR spectras for compounds **8**

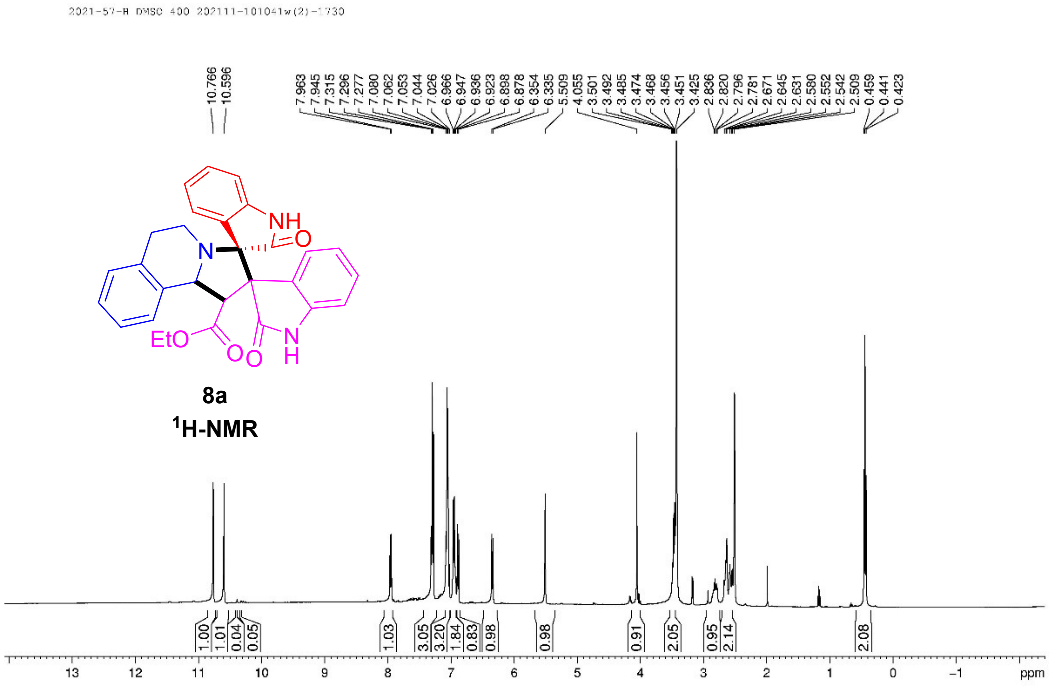

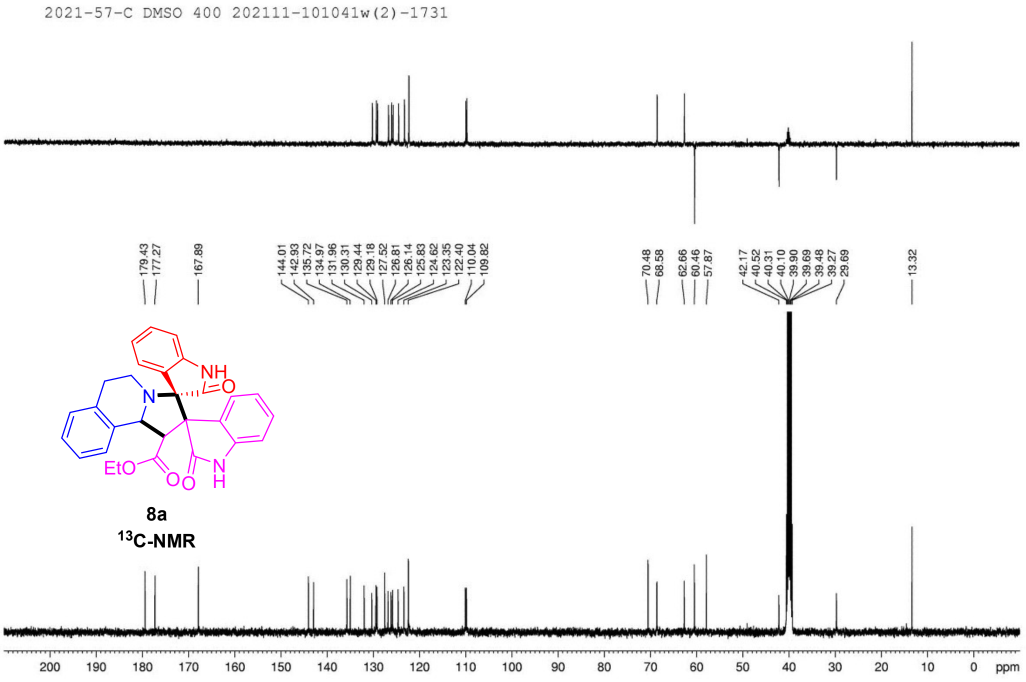

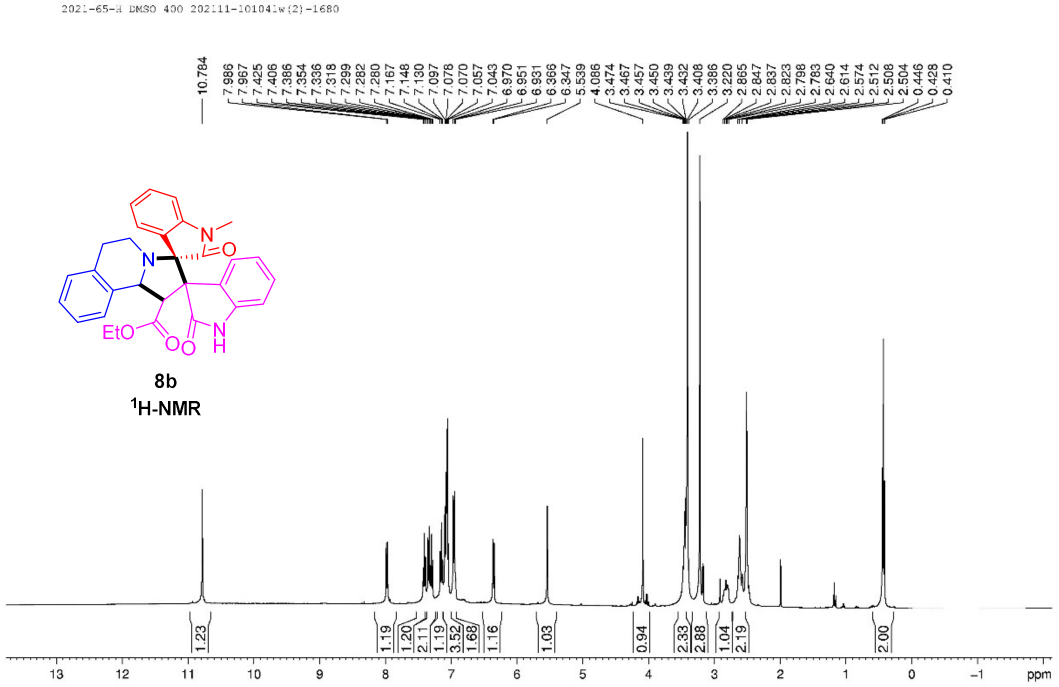
2021-65-H DMSO 400 202111-101041w(2)-1680
8b
1H-NMR
ppm

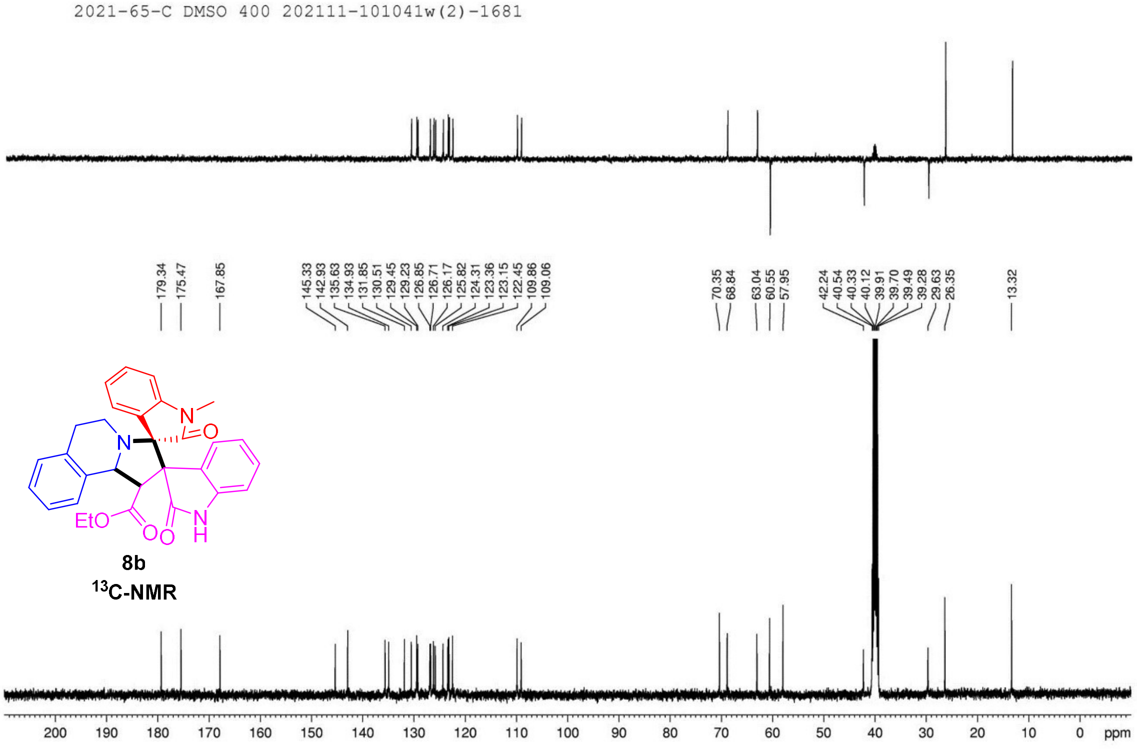
2021-65-C DMSO 400 202111-101041w(2)-1681
8b
13C-NMR
ppm

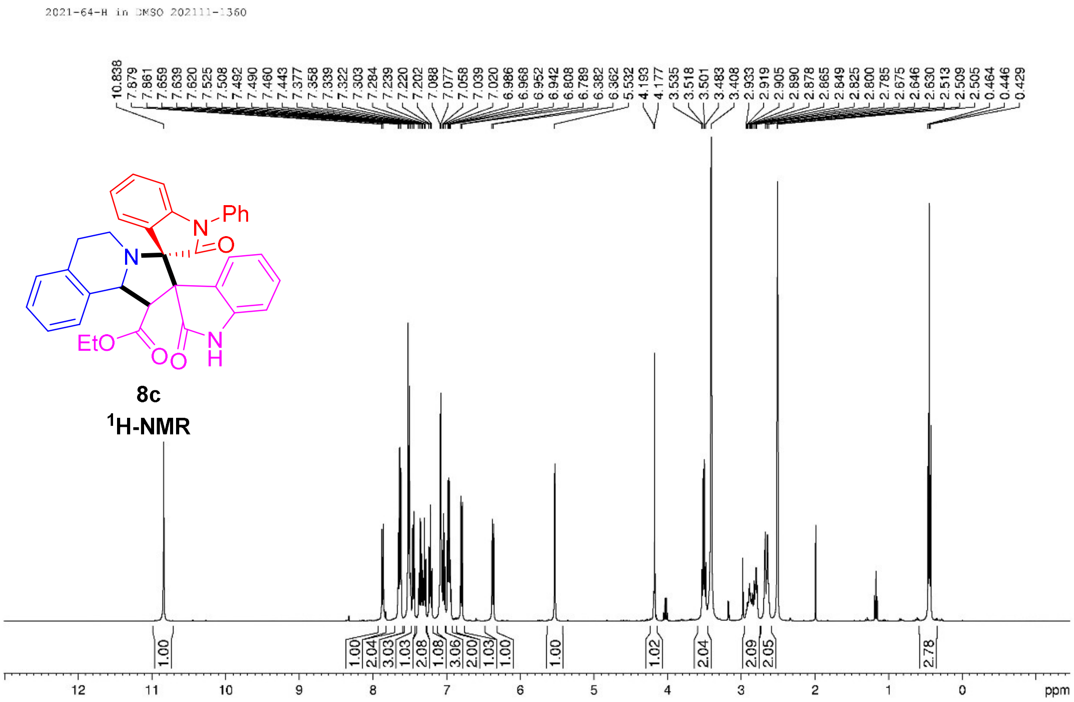
2021-64-H in DMSO 202111-1360
8c
1H-NMR
ppm

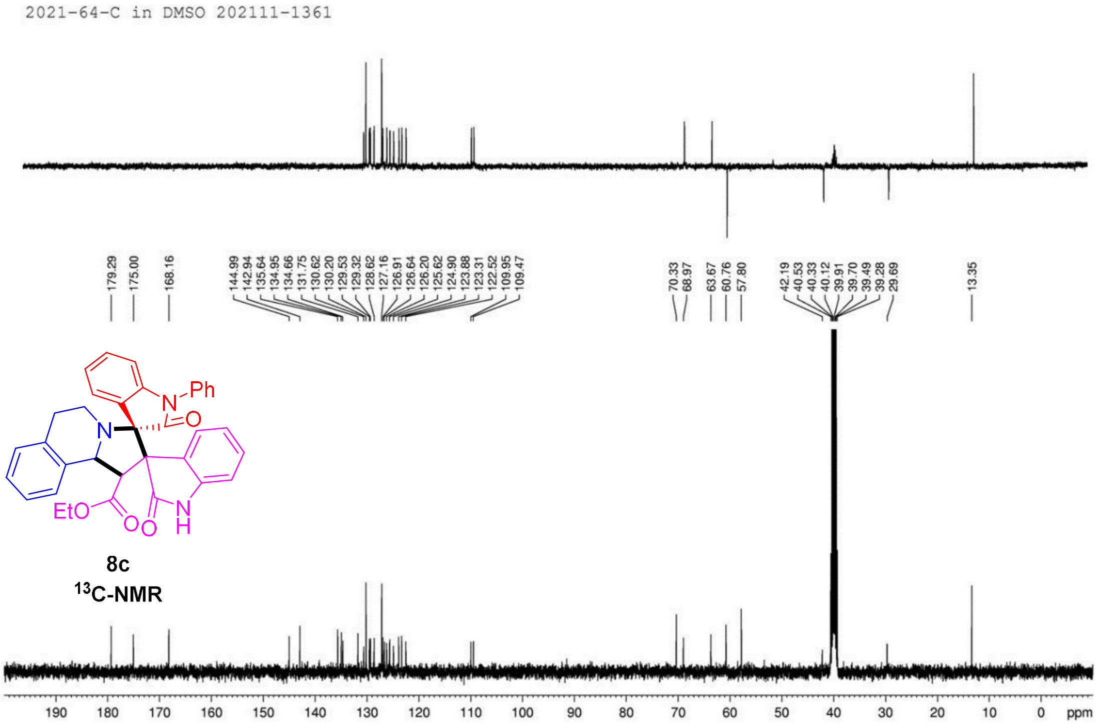
2021-64-C in DMSO 202111-1361
8c
13C-NMR
ppm

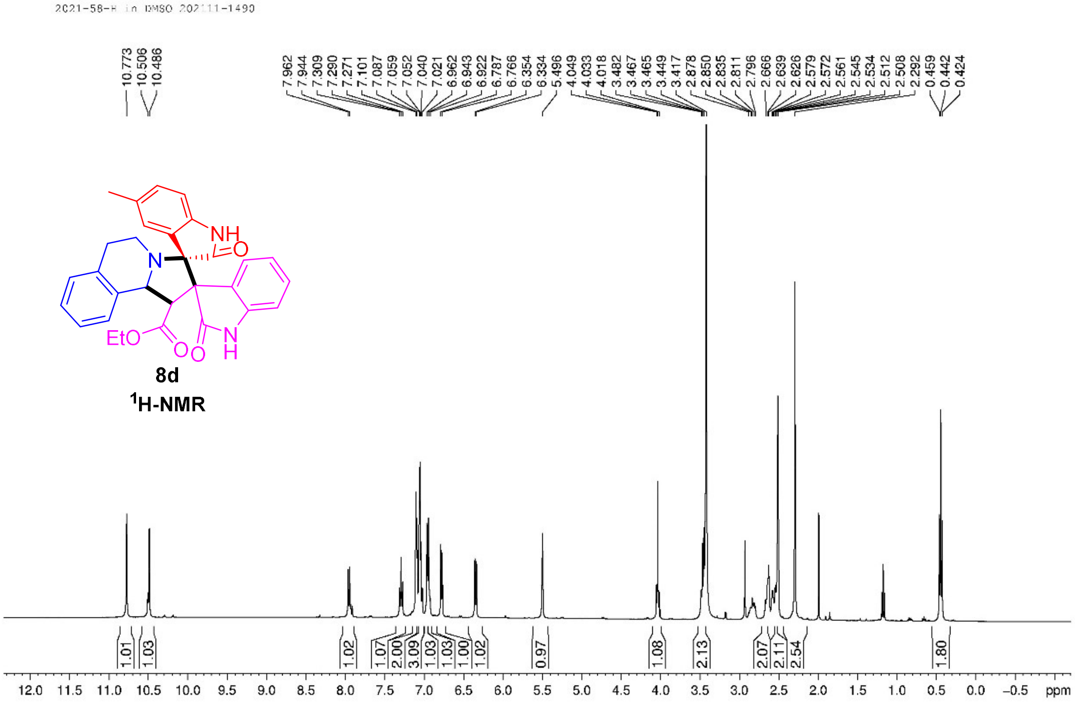
2021-58-H in DMSO 202111-1490
8d
¹H-NMR
EtO
NH
N
O
ppm

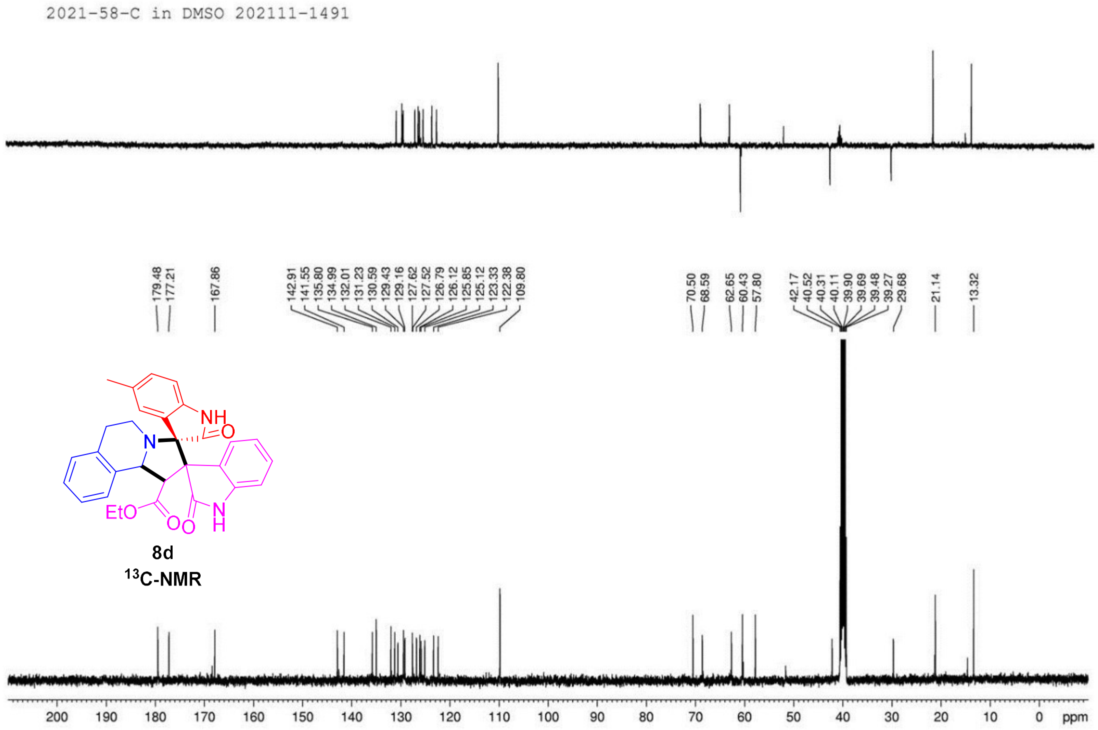
2021-58-C in DMSO 202111-1491
8d
¹³C-NMR
EtO
NH
N
O
ppm

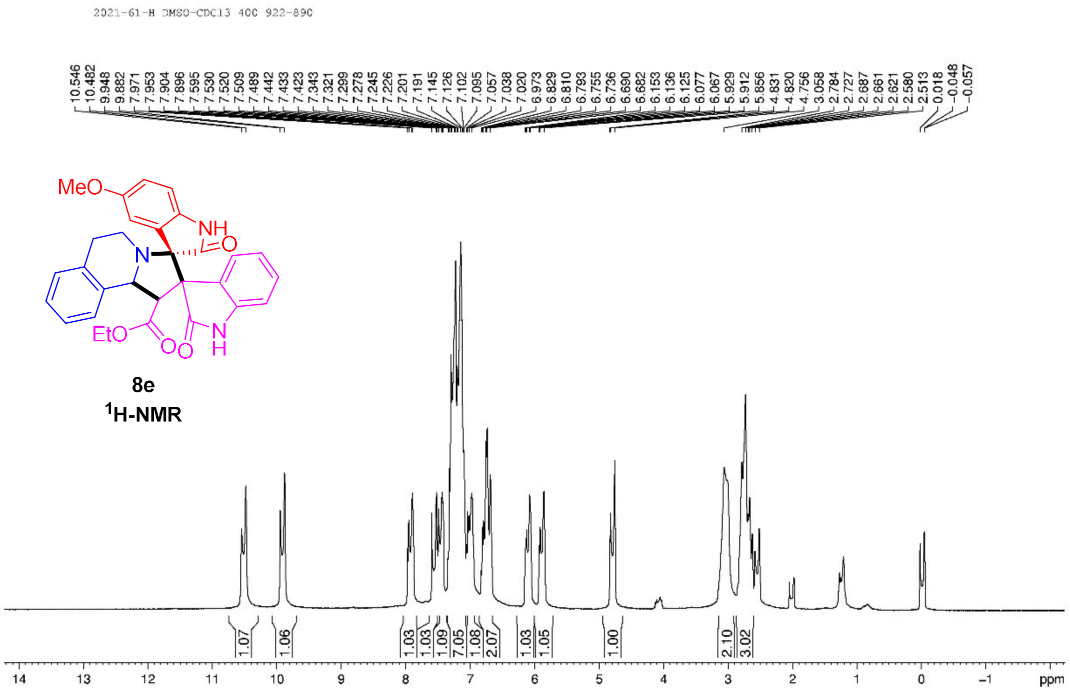
2021-61-H DMSO-CDCl3 400 922-890
10.546 10.482 9.948 9.882 7.971 7.953 7.904 7.896 7.595 7.530 7.520 7.509 7.489 7.442 7.433 7.423 7.343 7.321 7.299 7.278 7.245 7.226 7.201 7.191 7.145 7.126 7.102 7.095 7.057 7.038 7.020 6.973 6.829 6.810 6.793 6.755 6.736 6.690 6.682 6.153 6.136 6.125 6.077 6.067 5.929 5.912 5.856 4.831 4.820 4.756 3.058 2.784 2.727 2.687 2.661 2.621 2.580 2.513 0.018 -0.048 -0.057
MeO
NH
O
N
EtO
O
O
N
H
8e
1H-NMR
1.07
1.06
1.03
1.03
1.09
7.05
1.08
2.07
1.03
1.05
1.00
2.10
3.02
ppm

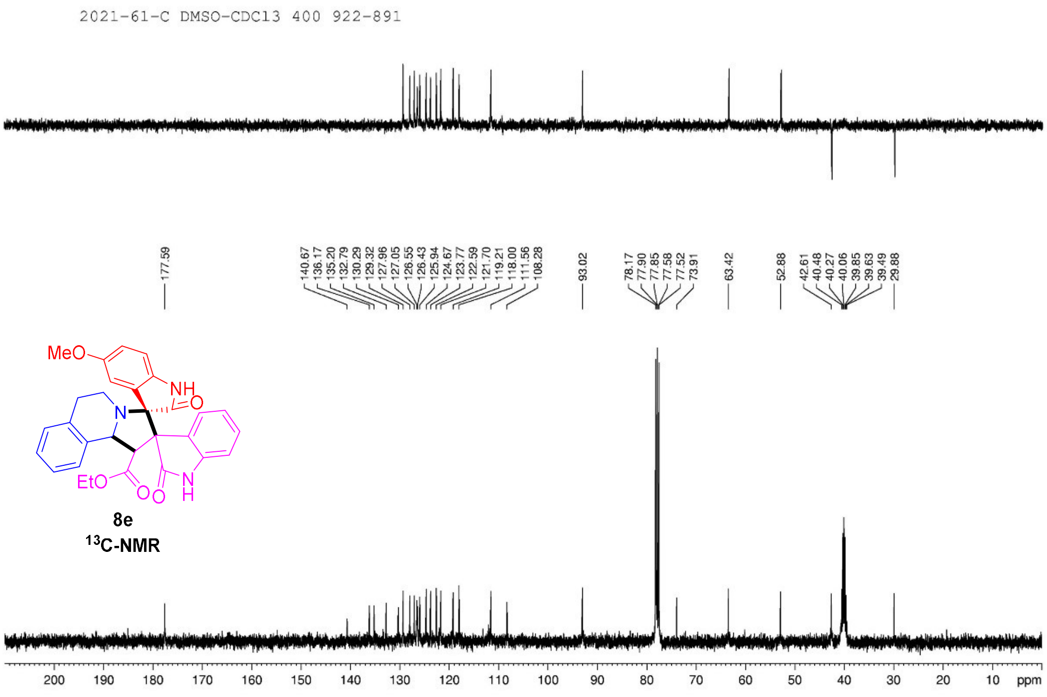
2021-61-C DMSO-CDCl3 400 922-891
177.59
140.67 136.17 135.20 132.79 130.29 129.32 127.96 127.05 126.55 126.43 125.94 124.67 123.77 122.59 121.70 119.21 118.00 111.56 108.28
93.02
78.17 77.90 77.85 77.58 77.52 73.91
63.42
52.88
42.61 40.48 40.27 40.06 39.85 39.63 39.49 29.88
MeO
NH
O
N
EtO
O
O
N
H
8e
13C-NMR
ppm

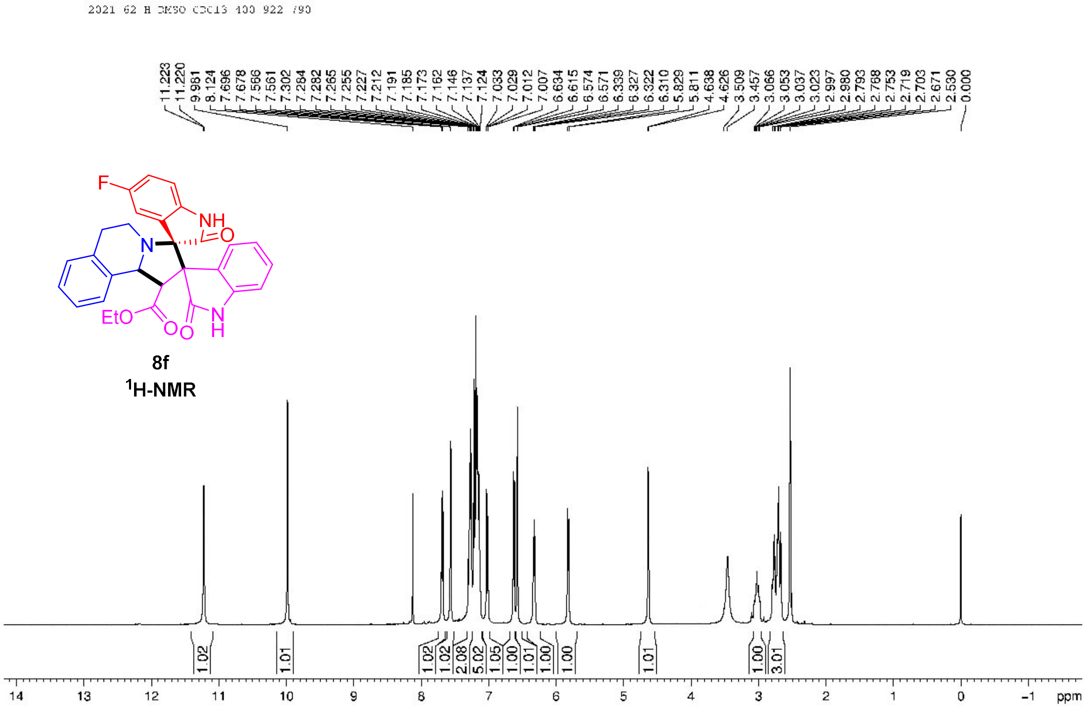
2021 62 H DMSO CDCl3 400 922 790
8f
^{1}H-NMR
ppm

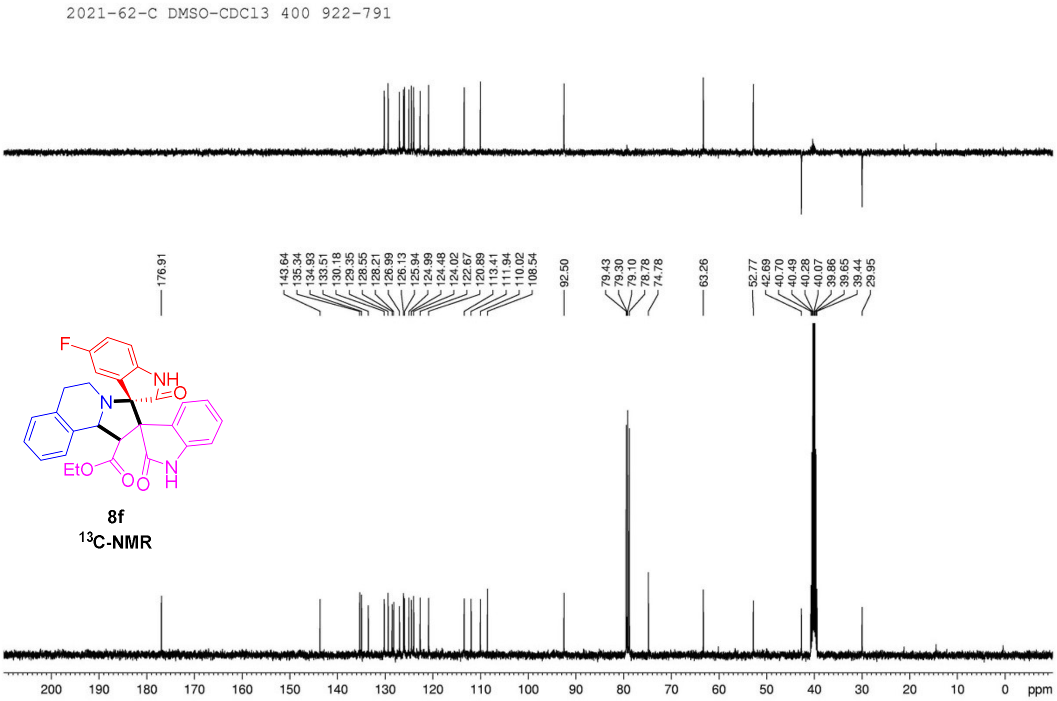
2021-62-C DMSO-CDCl3 400 922-791
8f
^{13}C-NMR
ppm

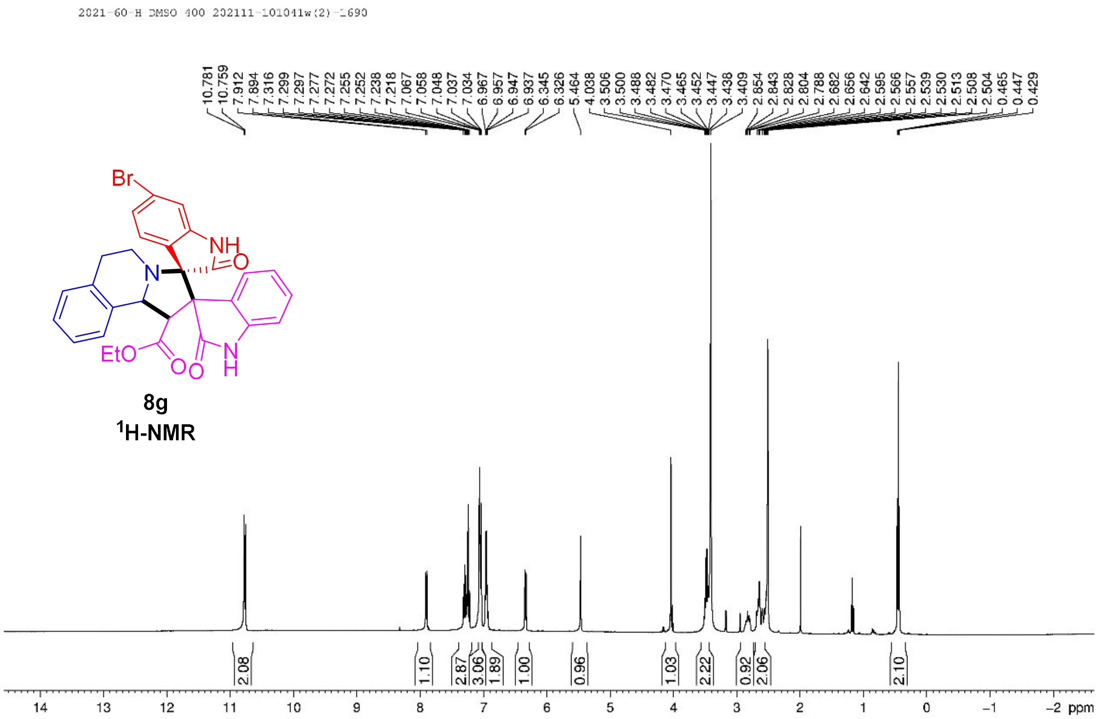
2021-60-H DMSO 400 202111-101041w(2)-1690
10.781
10.759
7.912
7.894
7.316
7.299
7.297
7.277
7.272
7.255
7.252
7.238
7.218
7.067
7.058
7.048
7.037
7.034
6.967
6.957
6.947
6.937
6.345
6.326
5.464
4.038
3.506
3.500
3.488
3.482
3.470
3.465
3.452
3.447
3.438
3.409
2.854
2.843
2.828
2.804
2.788
2.682
2.656
2.642
2.595
2.566
2.557
2.539
2.530
2.513
2.508
2.504
0.465
0.447
0.429
Br
NH
O
N
EtO
O
O
N
H
8g
1H-NMR
2.08
1.10
2.87
3.06
1.89
1.00
0.96
1.03
2.22
0.92
2.06
2.10
14
13
12
11
10
9
8
7
6
5
4
3
2
1
0
–1
–2 ppm

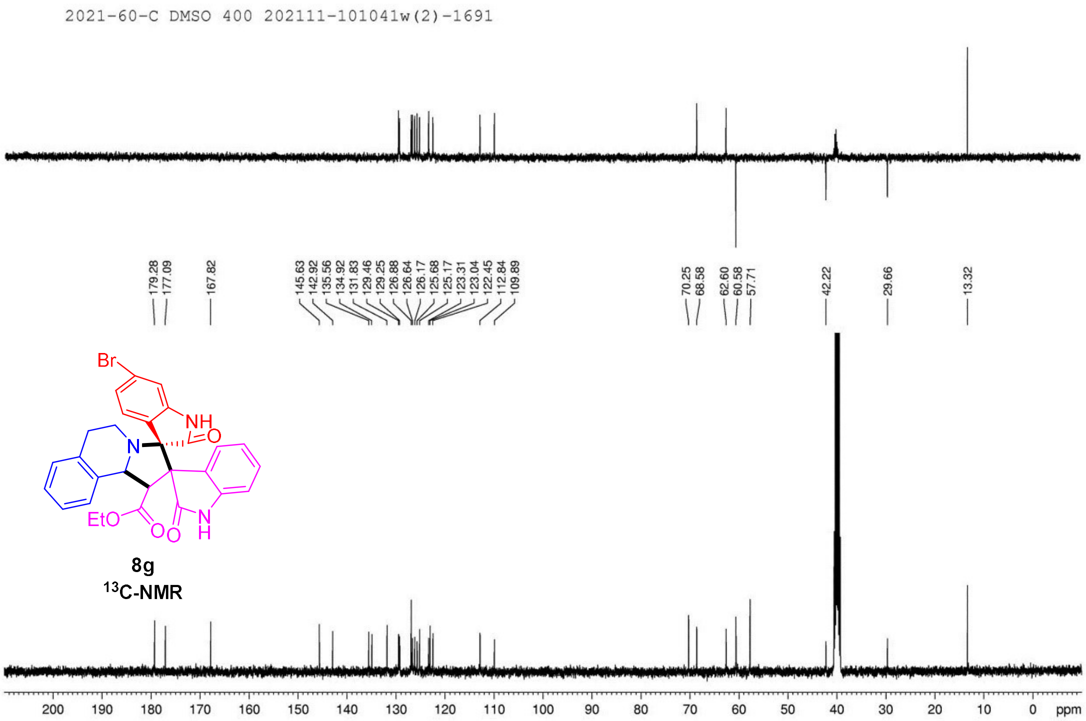
2021-60-C DMSO 400 202111-101041w(2)-1691
179.28
177.09
167.82
145.63
142.92
135.56
134.92
131.83
129.46
129.25
126.88
126.64
126.17
125.68
125.17
123.31
123.04
122.45
112.84
109.89
70.25
68.58
62.60
60.58
57.71
42.22
29.66
13.32
Br
NH
O
N
EtO
O
O
N
H
8g
13C-NMR
200
190
180
170
160
150
140
130
120
110
100
90
80
70
60
50
40
30
20
10
0
ppm

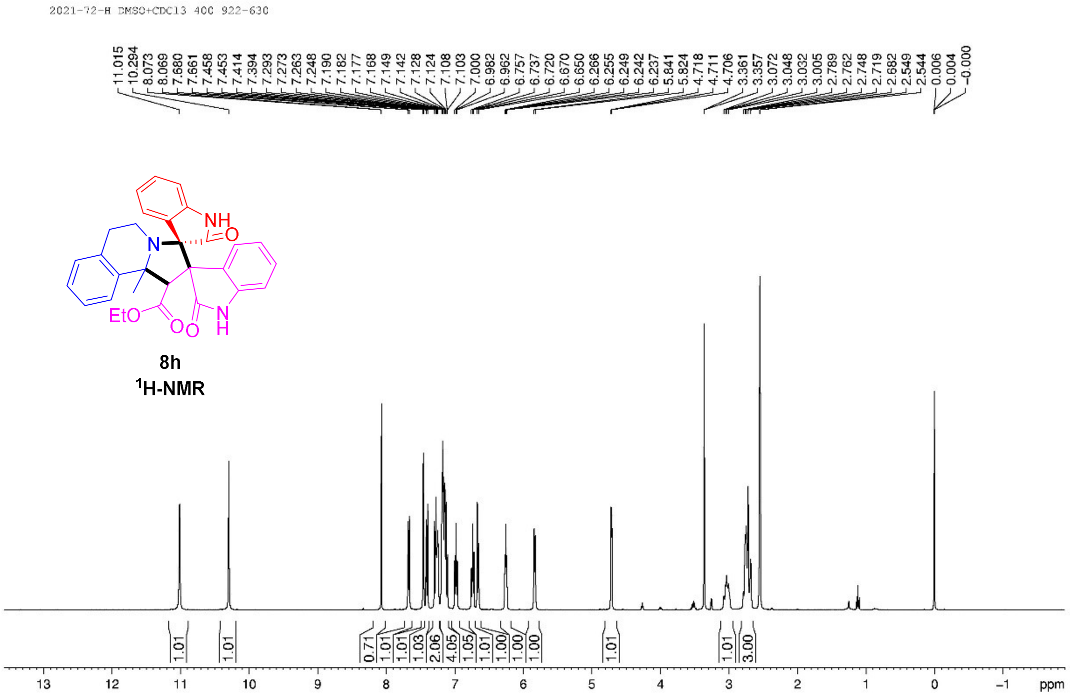
2021-72-H DMSO+CDCl3 400 922-630
11.015 10.294 8.073 8.069 7.680 7.661 7.458 7.453 7.414 7.394 7.293 7.273 7.263 7.248 7.190 7.182 7.177 7.168 7.149 7.142 7.128 7.124 7.108 7.103 7.000 6.982 6.962 6.757 6.737 6.720 6.670 6.650 6.266 6.255 6.249 6.242 6.237 5.841 5.824 4.718 4.711 4.706 3.361 3.357 3.072 3.048 3.032 3.005 2.789 2.762 2.748 2.719 2.682 2.549 2.544 0.006 0.004 -0.000
NH
N
O
EtO
O
O
N
H
8h
1H-NMR
1.01 1.01 0.71 1.01 1.01 1.03 2.06 4.05 1.05 1.01 1.00 1.00 1.00 1.01 1.01 3.00
13 12 11 10 9 8 7 6 5 4 3 2 1 0 -1 ppm

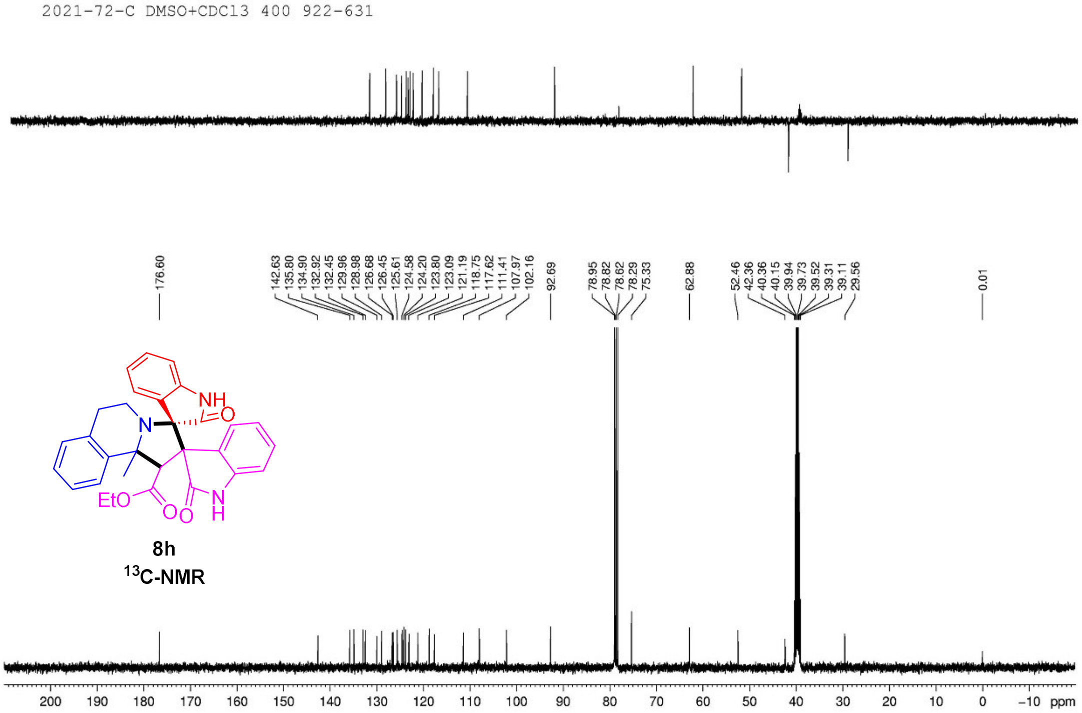
2021-72-C DMSO+CDCl3 400 922-631
176.60
142.63 135.80 134.90 132.92 132.45 129.96 128.98 126.68 126.45 125.61 124.58 124.20 123.80 123.09 121.19 118.75 117.62 111.41 107.97 102.16
92.69
78.95 78.82 78.62 78.29 75.33
62.88
52.46 42.36 40.36 40.15 39.94 39.73 39.52 39.31 39.11 29.56
0.01
NH
N
O
EtO
O
O
N
H
8h
13C-NMR
200 190 180 170 160 150 140 130 120 110 100 90 80 70 60 50 40 30 20 10 0 -10 ppm

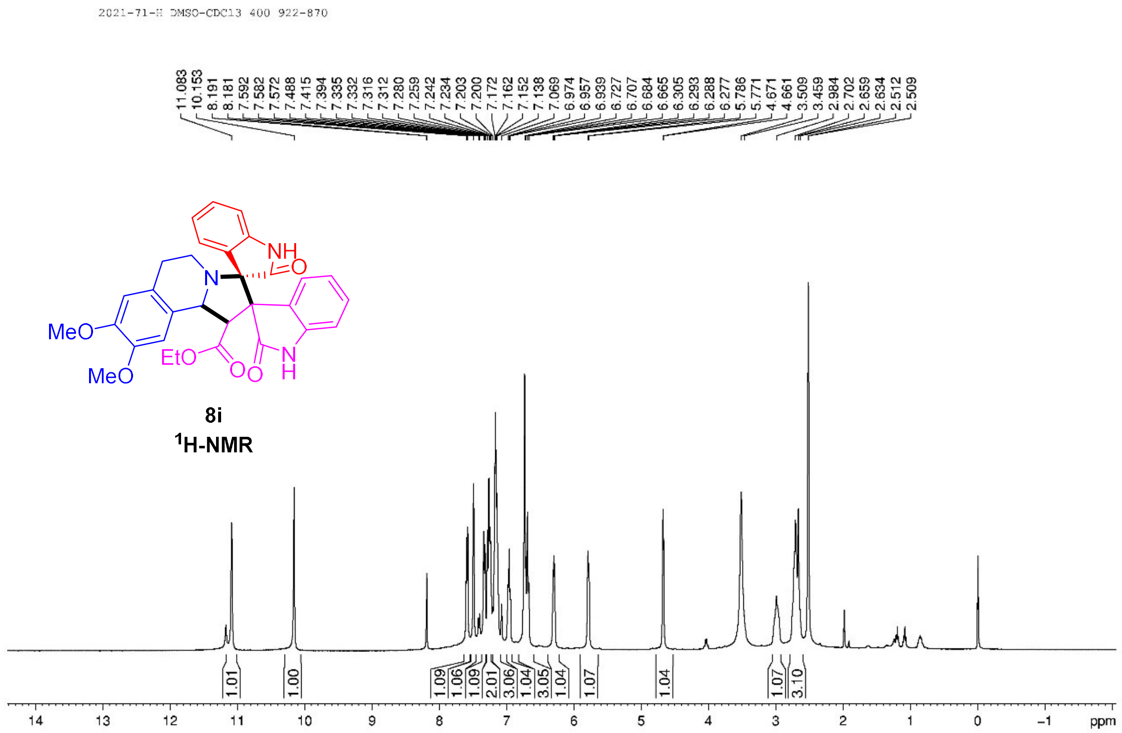
2021-71-H DMSO-CDCl3 400 922-870
11.083 10.153 8.191 8.181 7.592 7.582 7.572 7.488 7.415 7.394 7.335 7.332 7.316 7.312 7.280 7.259 7.242 7.234 7.203 7.200 7.172 7.162 7.152 7.138 7.069 6.974 6.957 6.939 6.727 6.707 6.684 6.665 6.305 6.293 6.288 6.277 5.786 5.771 4.671 4.661 3.509 3.459 2.984 2.702 2.659 2.634 2.512 2.509
MeO
MeO
EtO
NH
O
N
H
8i
1H-NMR
1.01 1.00 1.09 1.06 1.09 2.01 3.06 1.04 3.05 1.04 1.07 1.04 1.07 3.10
ppm

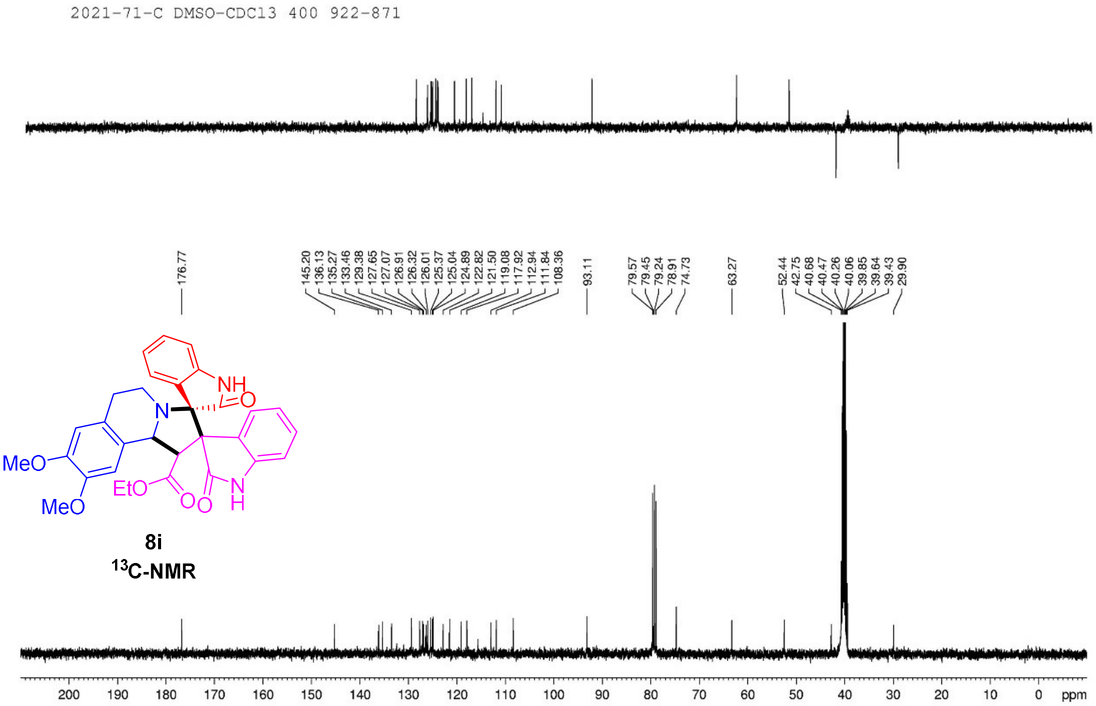
2021-71-C DMSO-CDCl3 400 922-871
176.77
145.20 136.13 135.27 133.46 129.38 127.65 127.07 126.91 126.32 126.01 125.37 125.04 124.89 122.82 121.50 119.08 117.92 112.94 111.84 108.36
93.11
79.57 79.45 79.24 78.91 74.73
63.27
52.44 42.75 40.68 40.47 40.26 40.06 39.85 39.64 39.43 29.90
MeO
MeO
EtO
NH
O
N
H
8i
13C-NMR
ppm

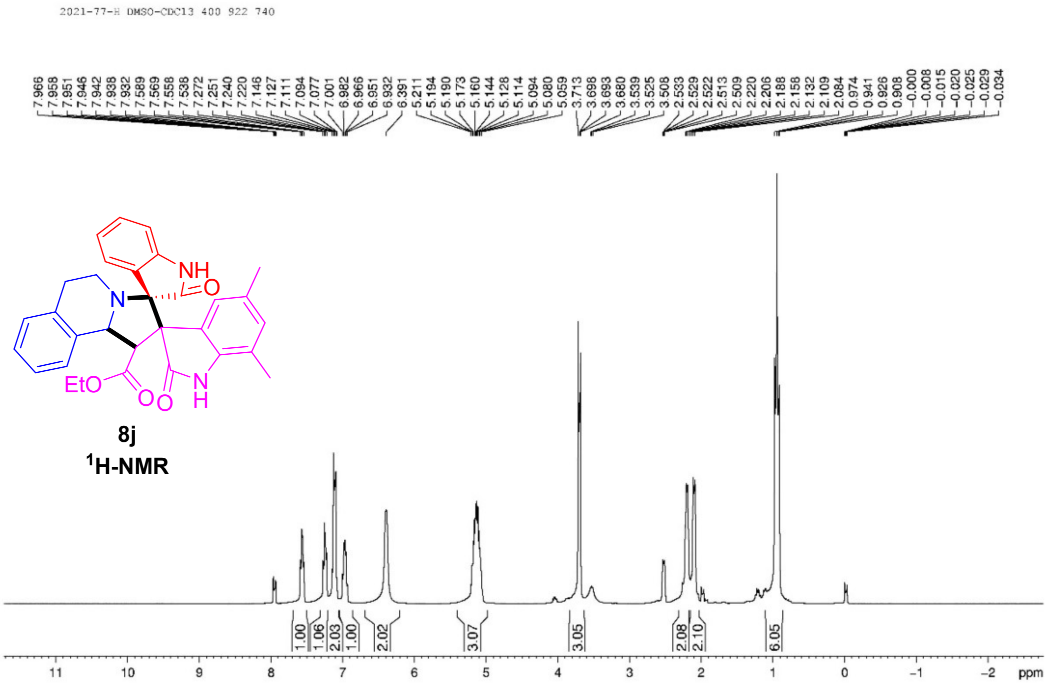
2021-77-H DMSO-CDCl3 400 922 740
NH
O
N
EtO
O
O
N
H
8j
1H-NMR
ppm

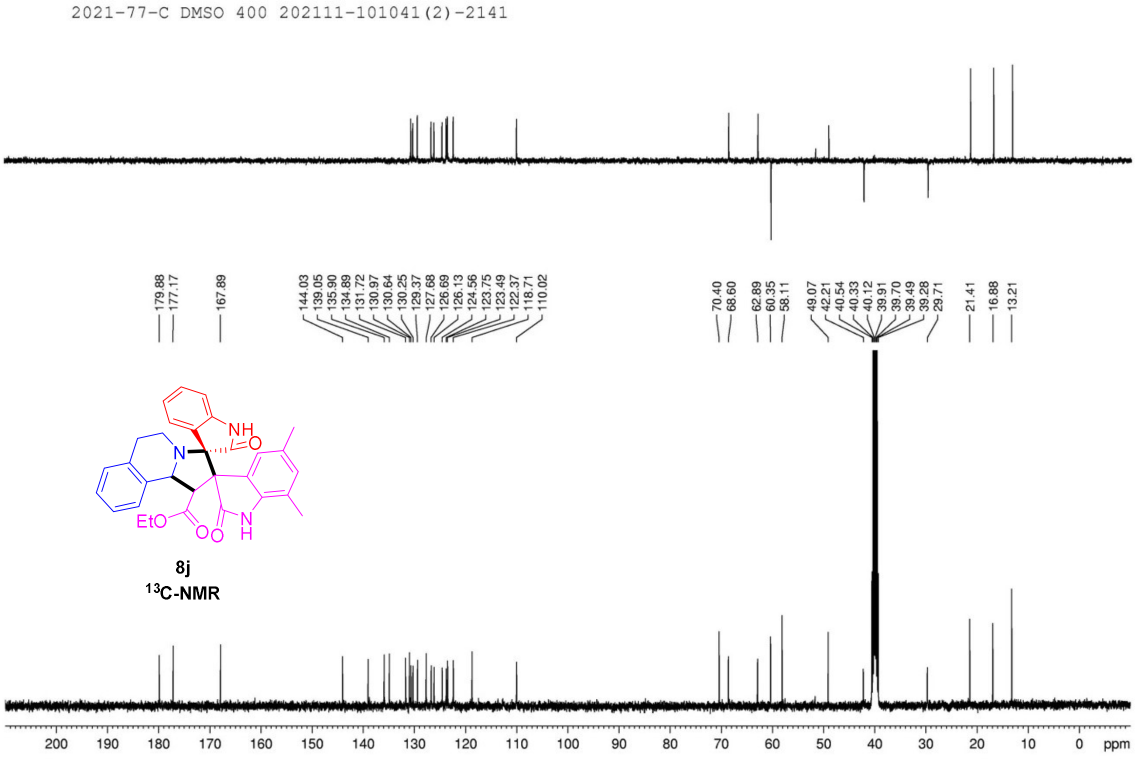
2021-77-C DMSO 400 202111-101041(2)-2141
NH
O
N
EtO
O
O
N
H
8j
13C-NMR
ppm

3. Single crystal X-ray diffraction study data of compound 4c

checkCIF/PLATON report

Structure factors have been supplied for datablock(s) exp_2483_auto

THIS REPORT IS FOR GUIDANCE ONLY. IF USED AS PART OF A REVIEW PROCEDURE FOR PUBLICATION, IT SHOULD NOT REPLACE THE EXPERTISE OF AN EXPERIENCED CRYSTALLOGRAPHIC REFEREE.

No syntax errors found.   CIF dictionary   Interpreting this report

Datablock: exp_2483_auto

Bond precision:	C-C = 0.0029 A		Wavelength=1.54184
Cell:	a=11.1104(2)	b=7.3378(2)	c=30.7872(7)
	alpha=90	beta=94.451(2)	gamma=90
Temperature:	100 K		

	Calculated	Reported
Volume	2502.38(10)	2502.38(10)
Space group	P 21/n	P 1 21/n 1
Hall group	-P 2yn	-P 2yn
Moiety formula	C30 H24 Cl N5 O3	C30 H24 Cl N5 O3
Sum formula	C30 H24 Cl N5 O3	C30 H24 Cl N5 O3
Mr	537.99	537.99
Dx,g cm-3	1.428	1.428
Z	4	4
Mu (mm-1)	1.715	1.715
F000	1120.0	1120.0
F000'	1124.64	
h,k,lmax	13,8,36	13,8,36
Nref	4412	4394
Tmin,Tmax	0.872,0.887	0.782,1.000
Tmin'	0.872	

Correction method= # Reported T Limits: Tmin=0.782 Tmax=1.000
AbsCorr = MULTI-SCAN

Data completeness= 0.996   Theta(max)= 66.600

R(reflections)= 0.0411(3907)   wR2(reflections)= 0.1095(4394)

S = 1.020   Npar= 353

```
The following ALERTS were generated. Each ALERT has the format
       test-name_ALERT_alert-type_alert-level.
Click on the hyperlinks for more details of the test.
```

Alert level C

```
PLAT420_ALERT_2_C D-H Bond Without Acceptor  N009      --H009      .      Please Check
PLAT906_ALERT_3_C Large K Value in the Analysis of Variance ......      2.359 Check
PLAT911_ALERT_3_C Missing FCF Refl Between Thmin & STh/L=    0.595        19 Report
```

Alert level G

```
PLAT007_ALERT_5_G Number of Unrefined Donor-H Atoms ..............          2 Report
PLAT720_ALERT_4_G Number of Unusual/Non-Standard Labels ..........         63 Note
PLAT793_ALERT_4_G Model has Chirality at C00D          (Centro SPGR)        R Verify
PLAT793_ALERT_4_G Model has Chirality at C00E          (Centro SPGR)        R Verify
PLAT793_ALERT_4_G Model has Chirality at C00H          (Centro SPGR)        S Verify
PLAT793_ALERT_4_G Model has Chirality at C00K          (Centro SPGR)        R Verify
PLAT909_ALERT_3_G Percentage of I>2sig(I) Data at Theta(Max) Still        84% Note
PLAT933_ALERT_2_G Number of HKL-OMIT Records in Embedded .res File          1 Note
PLAT941_ALERT_3_G Average HKL Measurement Multiplicity ...........        3.6 Low
PLAT978_ALERT_2_G Number C-C Bonds with Positive Residual Density.          9 Info
```

```
   0 ALERT level A = Most likely a serious problem - resolve or explain
   0 ALERT level B = A potentially serious problem, consider carefully
   3 ALERT level C = Check. Ensure it is not caused by an omission or oversight
  10 ALERT level G = General information/check it is not something unexpected

   0 ALERT type 1 CIF construction/syntax error, inconsistent or missing data
   3 ALERT type 2 Indicator that the structure model may be wrong or deficient
   4 ALERT type 3 Indicator that the structure quality may be low
   5 ALERT type 4 Improvement, methodology, query or suggestion
   1 ALERT type 5 Informative message, check
```

It is advisable to attempt to resolve as many as possible of the alerts in all categories. Often the minor alerts point to easily fixed oversights, errors and omissions in your CIF or refinement strategy, so attention to these fine details can be worthwhile. In order to resolve some of the more serious problems it may be necessary to carry out additional measurements or structure refinements. However, the purpose of your study may justify the reported deviations and the more serious of these should normally be commented upon in the discussion or experimental section of a paper or in the "special_details" fields of the CIF. checkCIF was carefully designed to identify outliers and unusual parameters, but every test has its limitations and alerts that are not important in a particular case may appear. Conversely, the absence of alerts does not guarantee there are no aspects of the results needing attention. It is up to the individual to critically assess their own results and, if necessary, seek expert advice.

Publication of your CIF in IUCr journals

A basic structural check has been run on your CIF. These basic checks will be run on all CIFs submitted for publication in IUCr journals (*Acta Crystallographica*, *Journal of Applied Crystallography*, *Journal of Synchrotron Radiation*); however, if you intend to submit to *Acta Crystallographica Section C* or *E* or *IUCrData*, you should make sure that full publication checks are run on the final version of your CIF prior to submission.

Publication of your CIF in other journals

Please refer to the *Notes for Authors* of the relevant journal for any special instructions relating to CIF submission.

PLATON version of 12/09/2022; check.def file version of 09/08/2022

Datablock exp_2483_auto - ellipsoid plot

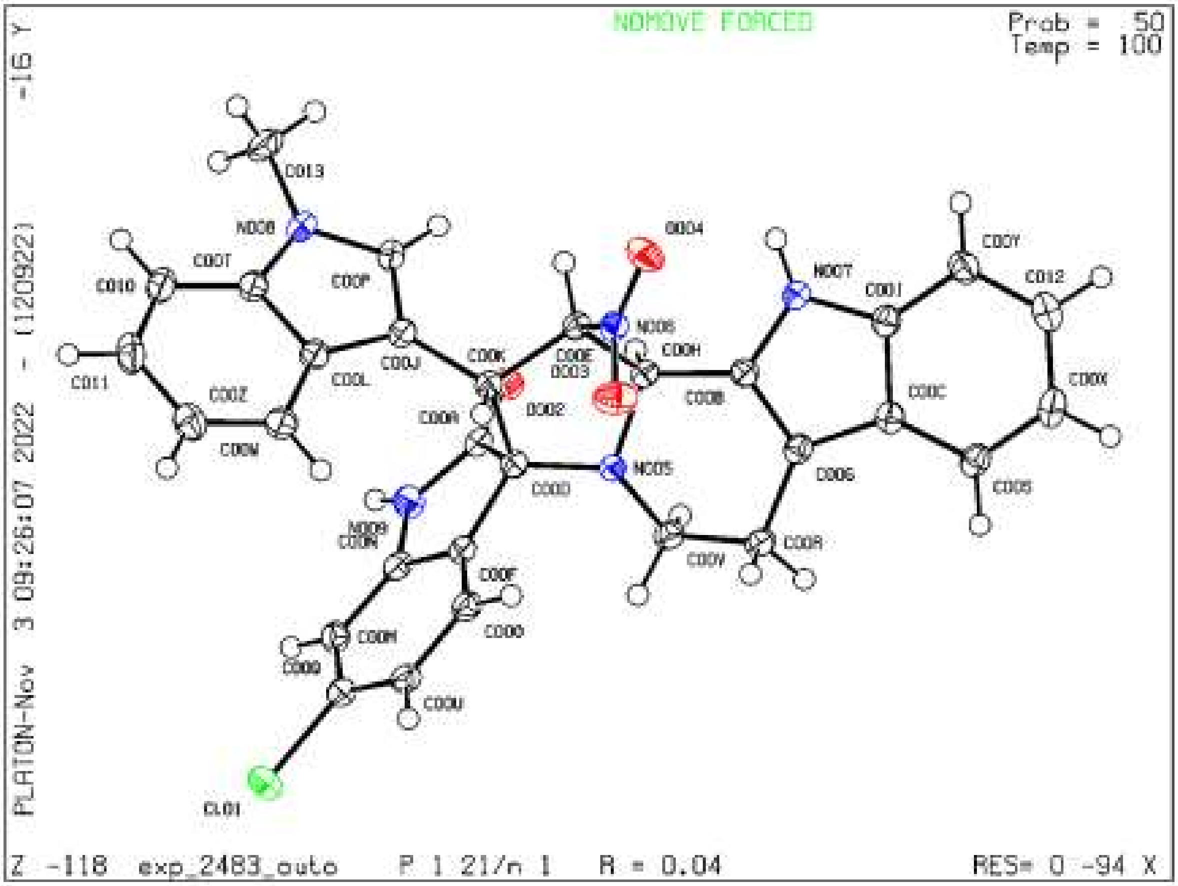

4. Single crystal X-ray diffraction study data of compound 6b

checkCIF/PLATON report

Structure factors have been supplied for datablock(s) exp_2413_auto

THIS REPORT IS FOR GUIDANCE ONLY. IF USED AS PART OF A REVIEW PROCEDURE FOR PUBLICATION, IT SHOULD NOT REPLACE THE EXPERTISE OF AN EXPERIENCED CRYSTALLOGRAPHIC REFEREE.

No syntax errors found. CIF dictionary Interpreting this report

Datablock: exp_2413_auto

```
Bond precision:   C-C = 0.0013 A          Wavelength=0.71073

Cell:            a=9.3321(2)        b=11.1799(2)      c=12.8313(3)
                 alpha=67.455(2)    beta=88.693(2)    gamma=86.534(2)
Temperature:     100 K

                  Calculated                   Reported
Volume            1234.15(5)                   1234.15(5)
Space group       P -1                         P -1
Hall group        -P 1                         -P 1
Moiety formula    C28 H24 N4 O3, C H4 O        C28 H24 N4 O3, C H4 O
Sum formula       C29 H28 N4 O4                C29 H28 N4 O4
Mr                496.55                       496.55
Dx,g cm-3         1.336                        1.336
Z                 2                            2
Mu (mm-1)         0.091                        0.091
F000              524.0                        524.0
F000'             524.23
h,k,lmax          15,18,21                     14,17,20
Nref              11542                        10382
Tmin,Tmax         0.984,0.991                  0.901,1.000
Tmin'             0.973

Correction method= # Reported T Limits: Tmin=0.901 Tmax=1.000
AbsCorr = MULTI-SCAN

Data completeness= 0.899           Theta(max)= 35.812

                                                wR2(reflections)=
R(reflections)= 0.0440( 8251)                   0.1328( 10382)
S = 1.070                   Npar= 337
```

```
The following ALERTS were generated. Each ALERT has the format
       test-name_ALERT_alert-type_alert-level.
Click on the hyperlinks for more details of the test.
```

Alert level C

```
PLAT230_ALERT_2_C Hirshfeld Test Diff for    N007     --C00R     .         6.2 s.u.
```

Alert level G

```
PLAT007_ALERT_5_G Number of Unrefined Donor-H Atoms ..............         3 Report
PLAT154_ALERT_1_G The s.u.'s on the Cell Angles are Equal ..(Note)     0.002 Degree
PLAT720_ALERT_4_G Number of Unusual/Non-Standard Labels ..........        65 Note
PLAT793_ALERT_4_G Model has Chirality at C00G        (Centro SPGR)         R Verify
PLAT793_ALERT_4_G Model has Chirality at C00K        (Centro SPGR)         R Verify
PLAT793_ALERT_4_G Model has Chirality at C00P        (Centro SPGR)         R Verify
PLAT793_ALERT_4_G Model has Chirality at C00R        (Centro SPGR)         R Verify
PLAT910_ALERT_3_G Missing # of FCF Reflection(s) Below Theta(Min).         3 Note
PLAT912_ALERT_4_G Missing # of FCF Reflections Above STh/L=  0.600      1052 Note
PLAT933_ALERT_2_G Number of HKL-OMIT Records in Embedded .res File         2 Note
PLAT941_ALERT_3_G Average HKL Measurement Multiplicity ...........       2.9 Low
PLAT978_ALERT_2_G Number C-C Bonds with Positive Residual Density.        24 Info
```

```
   0 ALERT level A = Most likely a serious problem - resolve or explain
   0 ALERT level B = A potentially serious problem, consider carefully
   1 ALERT level C = Check. Ensure it is not caused by an omission or oversight
  12 ALERT level G = General information/check it is not something unexpected

   1 ALERT type 1 CIF construction/syntax error, inconsistent or missing data
   3 ALERT type 2 Indicator that the structure model may be wrong or deficient
   2 ALERT type 3 Indicator that the structure quality may be low
   6 ALERT type 4 Improvement, methodology, query or suggestion
   1 ALERT type 5 Informative message, check
```

It is advisable to attempt to resolve as many as possible of the alerts in all categories. Often the minor alerts point to easily fixed oversights, errors and omissions in your CIF or refinement strategy, so attention to these fine details can be worthwhile. In order to resolve some of the more serious problems it may be necessary to carry out additional measurements or structure refinements. However, the purpose of your study may justify the reported deviations and the more serious of these should normally be commented upon in the discussion or experimental section of a paper or in the "special_details" fields of the CIF. checkCIF was carefully designed to identify outliers and unusual parameters, but every test has its limitations and alerts that are not important in a particular case may appear. Conversely, the absence of alerts does not guarantee there are no aspects of the results needing attention. It is up to the individual to critically assess their own results and, if necessary, seek expert advice.

Publication of your CIF in IUCr journals

A basic structural check has been run on your CIF. These basic checks will be run on all CIFs submitted for publication in IUCr journals (*Acta Crystallographica*, *Journal of Applied Crystallography*, *Journal of Synchrotron Radiation*); however, if you intend to submit to *Acta Crystallographica Section C* or *E* or *IUCrData*, you should make sure that full publication checks are run on the final version of your CIF prior to submission.

Publication of your CIF in other journals

Please refer to the *Notes for Authors* of the relevant journal for any special instructions relating to CIF submission.

PLATON version of 12/09/2022; check.def file version of 09/08/2022

Datablock exp_2413_auto - ellipsoid plot

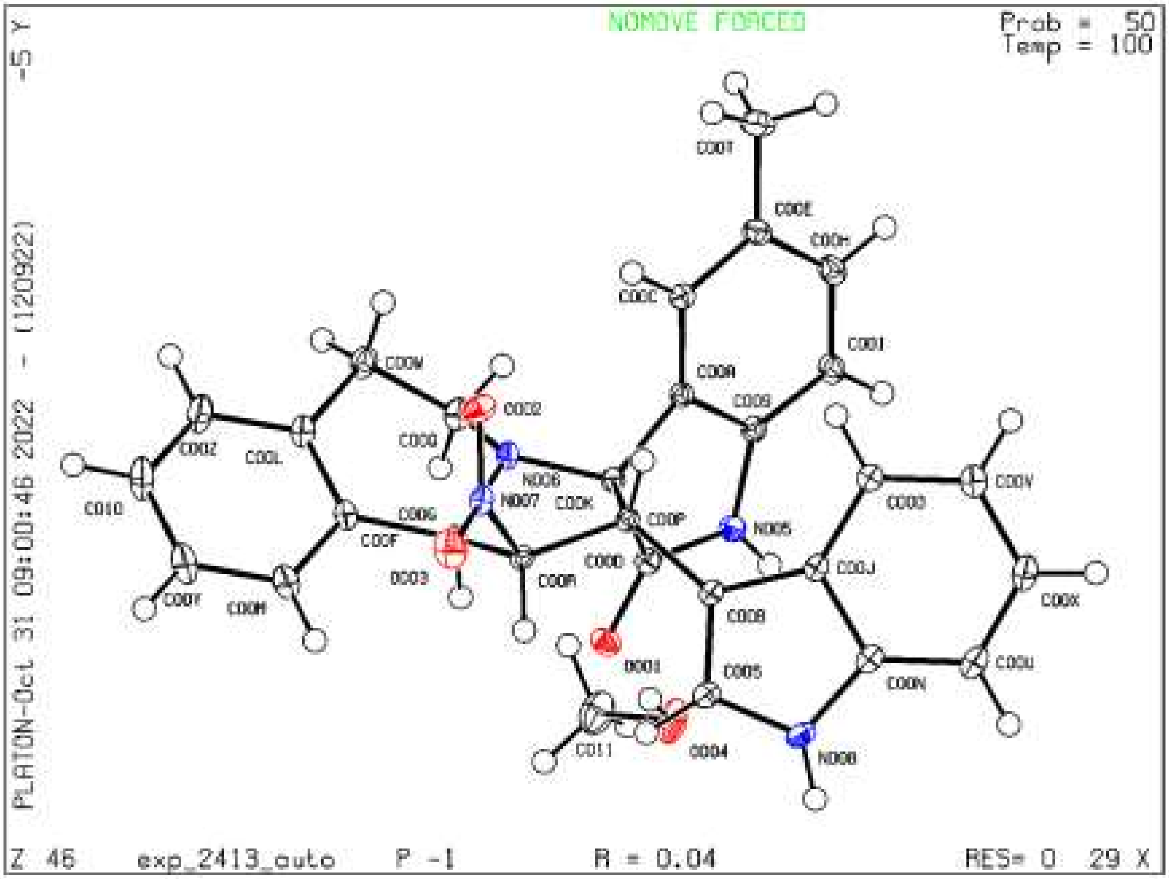

Supplement: Supplementary file 1 [file molecules-29-01790-s001.zip › molecules-2941732-supplementary.pdf]
